# Global, Regional, and National Burden Attributed to Particulate Matter Pollution, 1990–2021: A Systematic Analysis for the Global Burden of Disease Study 2021

**DOI:** 10.5334/aogh.4965

**Published:** 2026-02-24

**Authors:** YangYang Li, Ping Sun, Yiheng Yin, Chang Yu, Dongjie Xie, Zhengwei Wan, Bolin Deng

**Affiliations:** 1 Department of Rehabilitation, The First Affiliated Hospital, and College of Clinical Medicine of Henan University of Science and Technology, Luoyang, China; 2 Department of Health Management Center and Institute of Health Management, Sichuan Provincial People’s Hospital, University of Electronic Science and Technology of China, Chengdu, China; 3 Preventive Medicine Clinic, Sichuan Provincial Center for Disease Control and Prevention, Chengdu, China; 4 The First Affiliated Hospital, and College of Clinical Medicine of Henan University of Science and Technology, Luoyang, China; 5 Department of Ophthalmology, Sichuan Provincial People’s Hospital, University of Electronic Science and Technology of China, Chengdu, China

**Keywords:** ambient particulate matter pollution, household particulate matter pollution, GBD 2021, gender disparities, disease disparities

## Abstract

*Background:* Particulate matter pollution (PMP), both ambient (APMP) and household (HPMP), significantly contributes to global health issues, affecting mortality and disability-adjusted life years (DALYs) across different populations. This study aims to analyze the temporal and spatial trends of deaths and DALYs attributable to APMP and HPMP from 1990 to 2021, stratified by age, sex, and SDI, to understand the evolving global health burden.

*Method:* In this study, data on deaths, DALYs, and population attributable fractions due to overall PMP, APMP, and HPMP from 1990 to 2021 were obtained from the Global Burden of Disease Study 2021. The counts, rates per 100,000 population, and their estimated annual percentage changes, with 95% uncertainty intervals, were reported for each estimate.

*Results:* This study reveals that the global PMP-attributable deaths increased, driven by the doubling of APMP-attributable deaths. Rates attributable to overall PMP and HPMP decreased with rising SDI, while APMP-attributable rates followed an inverted U-shaped pattern, from 1990 to 2021. In 2021, the highest age-specific death and DALY rates occurred in infants and the elderly, with males consistently exhibiting higher rates than females. Regionally, North Africa and the Middle East, and Oceania had the highest rates attributable to APMP and HPMP, respectively, while South Asia showed the largest increase in APMP-attributable rates. The leading PMP-attributable diseases were cardiovascular diseases, maternal and neonatal disorders, and respiratory infections. APMP primarily contributed to chronic obstructive pulmonary disease (COPD), ischemic heart disease, and stroke, while HPMP had the greatest impact on lower respiratory infections, COPD, and neonatal disorders.

*Conclusions:* This study revealed that the burden of different PMP-attributable diseases varied by region, gender, and age. In addition, APMP-attributable deaths and DALYs doubled, with significant regional, gender, and age disparities, underscoring the need for targeted prevention and control strategies.

## Background

There is increasing evidence that ambient and household air pollution leads to various adverse health outcomes, such as respiratory diseases, cardiovascular diseases (CVDs), pulmonary diseases, cancer, low birth weight, diabetes, cognitive impairment, and mental health impacts [1]. The World Health Organization (WHO) estimated that air pollution causes 6.7 million deaths annually, with 4.2 million deaths resulting from outdoor air pollution in 2019 and 3.2 million deaths caused by household air pollution from burning wood and charcoal in 2020 [2, 3]. Ambient particulate matter pollution (APMP) and household particulate matter pollution (HPMP) are the largest contributors to the global burden of air pollution, accounting for over 90% of the air pollution disease burden and harming economic prospects and resilience [4]. Fine particulate matter pollution (PMP), also known as PM_2.5_, refers to airborne particles with a diameter of less than 2.5 microns. Among the major air pollutants currently measured, long-term exposure to PMP is the most consistent and accurate predictor of adverse health outcomes across populations. Its main components include sulfates, nitrates, ammonia, sodium chloride, black carbon, mineral dust, and water. The primary sources are vehicles, residential fuel use, coal-fired power plants, agricultural and industrial activities, waste burning, dust storms, wildfires, and various other anthropogenic emissions and natural factors [5].

Despite a slight decline in global HPMP exposure due to policy and technology advancements and a reversal in overall PMP trends after 2011, APMP exposure has continued to rise [6–8]. Combined with global population growth, aging, and the threats posed by climate change, this increase has significantly impacted human health [9–11]. Data from WHO indicated that nearly all global PMP levels exceed WHO guideline standards, with 99% of the world’s population still living in areas with unhealthy PMP levels [2]. The 2021 Global Burden of Disease (GBD) study on global risk factors indicated that PMP has become the leading cause of global disease burden, accounting for 8.0% of total disability-adjusted life years (DALYs) [4]. Additionally, the study has shown significant regional differences in exposure trends to PMP, with PMP exposure levels in low- and middle-income countries being 1–4 times higher than those in high-income countries [8]. To a large extent, these regional trends are closely related to socioeconomic development and national policy actions.

Given the persistently high burden of global PMP, understanding the detailed and up-to-date burden associated with PMP at global, regional, and national levels is crucial for preventing related diseases and controlling inhalable particulates. The GBD study 2021 is a systematic, up-to-date global epidemiological study aimed at quantifying incidence, death, and DALYs from major diseases, injuries, and risk factors by region, sex, age, and year [4, 12, 13]. This study aims to utilize the latest GBD 2021 dataset to summarize the disease burden attributable to PMP across different regions, genders, and age groups, providing a reference for the formulation of prevention and control strategies for PMP and related diseases.

## Methods

### Data sources

We obtained the number of deaths and DALYs, age-standardized rates (ASRs), and population attributable fractions (PAFs) with 95% uncertainty intervals (UIs) of disease attributable to PMP from the GBD 2021 using the Global Health Data Results Tool (https://vizhub.healthdata.org/gbd-results/). The GBD 2021 provided a comprehensive assessment of 288 causes of death, 371 diseases and injuries, and 88 risk factors in 204 countries and territories from 1990 to 2021. The protocol for GBD 2021 is available on the website of the Institute for Health Metrics and Evaluation (IHME) [14]. An overview of the collection, modeling/analysis, and dissemination of GBD data is available in the supplementary appendix (Appendix 1).

### Definitions

PMP was defined as fine particulate matter under 2.5 μm in aerodynamic diameter, including exposure from outdoor sources (ambient particulate matter pollution, APMP) and indoor pollution from solid fuels used for cooking (household particulate matter pollution, HPMP) [4]. A summary of the input data and modeling descriptions specific to APMP and HPMP in GBD 2021 is provided in the supplementary appendix (Appendix 2–3). In GBD 2021, causes of death and DALYs were classified into four distinct and comprehensive levels [12, 13]. PMP was associated with nine level 2 causes of death and 10 level 2 causes of DALYs across both sexes. Additionally, PMP contributed to 13 causes of death at level 3 and nine causes at level 4, as well as 14 causes of DALYs at level 3 and 10 causes at level 4, in both sexes.

We mainly used five indicators for the burden of disease analysis: total number of deaths, DALYs, ASRs, PAFs, and estimated annual percentage changes (EAPCs). The total number of deaths refers to the number of deaths each year attributable to past exposure to air pollution [13]. DALYs include both years of life lost due to premature death and years lived with disability due to poor health [12]. ASRs represent the number of deaths or DALYs per 100,000 people. Higher PMP-attributable age-standardized disease rates reflect a combination of higher PMP levels and sicker populations [15]. PAFs represent the proportionate changes in health risk that would result if exposure to a risk factor were lowered to the theoretical minimum risk levels. EAPCs were used to quantify the temporal trends of disease burden during 1990–2021 [16].

The socio-demographic index (SDI) serves as a comprehensive indicator of development status and is strongly correlated with health outcomes [12]. In 2021, GBD was calculated as the geometric mean ranging from 0 to 100 of three key indicators: fertility rates among females under 25, average years of education for individuals aged 15 and above, and lag-distributed income per capita. The construction of SDI and the categorization of 204 countries and territories into five quintiles (low, low-middle, middle, high-middle, and high) based on their SDI 2021 estimates were detailed in the GBD 2021 capstone paper [12].

### Statistical analysis

We calculated the EAPCs with 95% confidence intervals (CIs) in ASRs to assess the average trends over a specified period. A 95% CI excluding 0 was statistically significant. The specific method for calculating EAPCs is provided in our previously published article [17]. We plotted the number of deaths or DALYs, ASRs, PAFs, and EAPCs to visualize the burden of disease attributable to overall PMP, AMAP, and HAMP by age, sex, year, and location. We also used Spearman correlation analysis to explore the relationship between SDI and the burden of disease attributable to PMP by location and year [17]. The related codes are available at https://ghdx.healthdata.org/gbd-2021/code. All statistical analyses and graphical representations were performed using R software (version 4.2.2).

## Results

### Overall impact of particulate matter pollution

#### Global trends in PMP-attributable deaths and DALYs

In 2021, the number of global PMP-attributable deaths was 7,833,220.92 (95% UI, 6,479,473.76 to 9,263,395.35), with 4,718,812.24 (3,480,471.83 to 5,795,946.44) attributed to APMP and 3,112,926.41 (1,895,168.20 to 5,188,696.63) to HPMP. The global PMP-attributable DALYs were 231,511,232.94 (194,538,892.20 to 270,855,451.37), with 120,004,672.01 (86,560,331.22 to 149,810,185.34) for APMP and 111,462,958.29 (75,085,852.45 to 163,710,710.53) for HPMP (Table 1). From 1990 to 2021, PMP-attributable deaths increased globally, primarily due to a doubling in APMP-attributable deaths for both females and males (Tables S1–S3, Figure S1-3A). Despite a decrease in global PMP-attributable DALYs, APMP-attributable DALYs still doubled for both sexes (Tables S4–S6, Figure S1–3B). However, ASRs of PMP-attributable deaths and DALYs significantly declined globally from 1990 to 2021, with similar trends for both APMP and HPMP in females and males (Tables S1–S6, Figures S4–S6).

**Table 1:** Global deaths and DALYs attributable to particulate matter pollution in 2021 and estimated annual percentage changes from 1990 to 2021.

CAUSE OF DEATH OR DALYS	DEATHS				DALYS			
	NUMBER OF CASES, 2021	AGE-STANDARDIZED RATES PER 100,000 PEOPLE, 2021	AGE-STANDARDIZED PAF (%), 2021	EAPC IN AGE-STANDARDIZED RATES (%), 1990–2021	NUMBER OF CASES, 2021	AGE-STANDARDIZED RATES PER 100,000 PEOPLE, 2021	AGE-STANDARDIZED PAF (%), 2021	EAPC IN AGE-STANDARDIZED RATES (%), 1990–2021
**All causes**	7,833,220.92 (6,479,473.76 to 9,263,395.35)	96.69 (79.91 to 114.40)	11.57 (9.73 to 13.53)	−2.18 (−2.31 to −2.04)	231,511,232.94 (194,538,892.20 to 270,855,451.37)	2984.47 (2489.63 to 3487.35)	8.25 (6.85 to 9.69)	−2.27 (−2.39 to −2.15)
APMP-attributable causes	4,718,812.24 (3,480,471.83 to 5,795,946.44)	57.62 (42.32 to 70.99)	6.90 (5.13 to 8.41)	−0.34 (−0.48 to −0.20)	120,004,672.01 (86,560,331.22 to 149,810,185.34)	1483.61 (1069.48 to 1869.55)	4.10 (2.98 to 5.13)	−0.28 (−0.43 to −0.12)
HPMP-attributable causes	3,112,926.41 (1,895,168.20 to 5,188,696.63)	39.05 (23.97 to 64.45)	4.67 (2.86 to 7.63)	−3.81 (−4.13 to −3.49)	111,462,958.29 (75,085,852.45 to 163,710,710.53)	1500.29 (1028.38 to 2195.56)	4.15 (2.79 to 5.97)	−3.52 (−3.77 to −3.27)
Female	3,478,614.32 (2,842,903.78 to 4,127,413.84)	78.35 (64.30 to 92.70)	11.51 (9.52 to 13.43)	−2.35 (−2.47 to −2.23)	99,047,039.32 (82,679,826.81 to 117,756,358.69)	2480.23 (2059.40 to 2951.19)	7.60 (6.20 to 9.00)	−2.43 (−2.54 to −2.31)
Male	4,354,606.60 (3,629,597.41 to 5,195,553.86)	119.23 (99.30 to 142.09)	11.70 (9.83 to 13.72)	−2.06 (−2.21 to −1.90)	132,464,193.62 (110,995,515.82 to 154,576,904.15)	3535.80 (2953.30 to 4130.72)	8.83 (7.39 to 10.23)	−2.16 (−2.28 to −2.03)
**Cardiovascular diseases**	4,482,496.23 (3,567,564.40 to 5,392,617.34)	53.62 (42.70 to 64.57)	22.80 (18.84 to 26.90)	−1.92 (−2.06 to −1.78)	99,637,837.09 (80,843,498.77 to 118,292,884.66)	1161.77 (939.61 to 1380.37)	22.98 (19.09 to 26.85)	−1.90 (−2.03 to −1.76)
Ischemic heart disease	2,492,809.91 (1,866,677.65 to 3,103,230.08)	29.88 (22.33 to 37.22)	27.48 (21.21 to 33.85)	−1.37 (−1.47 to −1.28)	54,675,670.12 (41,652,489.01 to 67,418,885.61)	638.48 (486.47 to 787.82)	28.86 (22.37 to 35.37)	−1.31 (−1.41 to −1.22)
Stroke	1,989,686.32 (1,530,479.07 to 2,493,237.87)	23.74 (18.26 to 29.80)	27.15 (21.64 to 33.64)	−2.51 (−2.72 to −2.30)	44,962,166.97 (35,020,338.76 to 55,467,023.53)	523.30 (407.96 to 645.58)	27.75 (22.32 to 34.22)	−2.49 (−2.68 to −2.30)
Intracerebral hemorrhage	995,650.44 (763,171.63 to 1,249,510.29)	11.69 (8.94 to 14.69)	29.91 (23.60 to 36.66)	−2.54 (−2.82 to −2.26)	24,015,341.78 (18,414,608.05 to 29,838,879.10)	276.93 (212.21 to 344.36)	29.98 (23.83 to 36.70)	−2.58 (−2.83 to −2.33)
Ischemic stroke	905,602.35 (694,785.38 to 1,144,793.07)	11.01 (8.44 to 13.96)	24.93 (19.93 to 31.10)	−2.19 (−2.37 to −2.02)	18,295,352.08 (14,324,970.64 to 22,541,397.17)	215.64 (168.84 to 266.03)	25.76 (20.60 to 32.03)	−2.04 (−2.20 to −1.87)
Subarachnoid hemorrhage	88,433.52 (65,255.07 to 116,468.07)	1.04 (0.76 to 1.36)	24.73 (19.35 to 30.95)	−4.64 (−4.92 to −4.35)	2,651,473.11 (1,994,097.80 to 3,505,548.54)	30.73 (23.13 to 40.64)	24.50 (19.35 to 30.48)	−4.20 (−4.43 to −3.97)
**Maternal and neonatal disorders**	496,966.05 (419,486.39 to 580,880.21)	8.03 (6.78 to 9.39)	25.13 (23.34 to 27.04)	−1.53 (−1.63 to −1.43)	44,737,310.78 (37,766,689.50 to 52,293,054.19)	723.06 (610.39 to 845.18)	23.35 (21.54 to 25.21)	−1.53 (−1.63 to −1.43)
Neonatal disorders	496,966.05 (419,486.39 to 580,880.21)	8.03 (6.78 to 9.39)	27.18 (25.28 to 29.26)	−1.53 (−1.63 to −1.43)	44,737,310.78 (37,766,689.50 to 52,293,054.19)	723.06 (610.39 to 845.18)	24.58 (22.65 to 26.55)	−1.53 (−1.63 to −1.43)
Neonatal preterm birth	227,615.56 (189,160.11 to 271,466.91)	3.68 (3.06 to 4.39)	30.84 (28.69 to 33.10)	−1.73 (−1.80 to −1.66)	20,494,977.58 (17,038,342.06 to 24,435,506.76)	331.25 (275.38 to 394.95)	26.41 (24.31 to 28.80)	−1.73 (−1.80 to −1.66)
Neonatal encephalopathy due to birth asphyxia and trauma	155,361.31 (129,104.49 to 187,255.72)	2.51 (2.09 to 3.03)	25.77 (23.38 to 28.09)	−1.15 (−1.32 to −0.98)	13,980,138.89 (11,617,949.83 to 16,850,172.87)	225.97 (187.79 to 272.36)	24.24 (21.91 to 26.44)	−1.15 (−1.32 to −0.98)
Neonatal sepsis and other neonatal infections	54,025.69 (45,371.42 to 64,084.44)	0.87 (0.73 to 1.04)	23.24 (21.62 to 24.81)	−0.96 (−1.09 to −0.84)	4,861,948.25 (4,083,310.36 to 5,767,175.69)	78.57 (65.98 to 93.19)	21.20 (19.59 to 22.79)	−0.96 (−1.09 to −0.84)
Other neonatal disorders	52,435.68 (36,764.50 to 65,587.59)	0.85 (0.59 to 1.06)	23.65 (21.45 to 25.59)	−1.77 (−1.94 to −1.61)	4,720,040.50 (3,309,887.35 to 5,902,580.05)	76.29 (53.49 to 95.40)	23.34 (21.15 to 25.30)	−1.77 (−1.93 to −1.61)
Hemolytic disease and other neonatal jaundice	7527.82 (5818.67 to 9920.66)	0.12 (0.09 to 0.16)	22.40 (20.29 to 24.79)	−3.52 (−3.69 to −3.35)	680,205.56 (526,741.16 to 895,348.64)	10.99 (8.51 to 14.47)	18.82 (16.78 to 20.90)	−3.51 (−3.68 to −3.34)
**Respiratory infections and tuberculosis**	65,1356.39 (12,1714.90 to 1,076,711.75)	8.68 (1.71 to 14.39)	6.34 (1.26 to 10.44)	−3.02 (−3.19 to −2.86)	29,110,366.69 (7,007,734.71 to 48,141,777.96)	420.28 (106.66 to 693.37)	9.63 (2.44 to 15.57)	−3.76 (−3.96 to −3.55)
Lower respiratory infections	651,238.01 (121,605.08 to 1,076,503.21)	8.68 (1.71 to 14.39)	30.21 (6.15 to 48.36)	−3.02 (−3.19 to −2.86)	29,098,330.84 (6,988,264.72 to 48,127,683.27)	420.09 (106.35 to 693.12)	35.84 (9.29 to 56.41)	−3.76 (−3.96 to −3.55)
Upper respiratory infections	116.58 (18.26 to 299.46)	0.00 (0.00 to 0.00)	0.59 (0.28 to 0.85)	−2.44 (−2.52 to −2.37)	11,708.46 (2800.50 to 28,081.79)	0.19 (0.05 to 0.45)	0.23 (0.07 to 0.49)	−2.32 (−2.39 to −2.25)
Otitis media	1.80 (0.55 to 4.62)	0.00 (0.00 to 0.00)	0.41 (0.24 to 0.74)	−6.28 (−6.51 to −6.05)	327.40 (156.27 to 626.36)	0.01 (0.00 to 0.01)	0.02 (0.01 to 0.03)	−4.54 (−4.80 to −4.28)
**Chronic respiratory diseases**	1,535,298.01 (1,214,704.19 to 1,918,255.85)	18.51 (14.64 to 23.13)	34.57 (28.32 to 42.55)	−2.96 (−3.13 to −2.79)	332,38712.44 (26,680,066.05 to 41,336,741.04)	389.50 (312.59 to 484.62)	30.10 (24.54 to 36.88)	−2.87 (−3.01 to −2.74)
Chronic obstructive pulmonary disease	1,535,298.01 (1,214,704.19 to 1,918,255.85)	18.51 (14.64 to 23.13)	40.95 (33.37 to 50.00)	−2.96 (−3.13 to −2.79)	33,238,712.44 (26,680,066.05 to 41,336,741.04)	389.50 (312.59 to 484.62)	41.41 (34.01 to 50.17)	−2.87 (−3.01 to −2.74)
**Diabetes and kidney diseases**	281,908.84 (165,678.17 to 395,527.82)	3.32 (1.95 to 4.66)	8.69 (5.35 to 12.41)	0.03 (−0.05 to 0.10)	12,904,493.55 (7,501,414.23 to 19,485,253.91)	148.92 (86.50 to 224.91)	10.25 (6.18 to 14.71)	0.77 (0.71 to 0.83)
Diabetes mellitus	281,908.84 (165,678.17 to 395,527.82)	3.32 (1.95 to 4.66)	16.94 (10.38 to 24.09)	0.03 (−0.05 to 0.10)	12,904,493.55 (7,501,414.23 to 19,485,253.91)	148.92 (86.50 to 224.91)	16.24 (9.94 to 23.12)	0.77 (0.71 to 0.83)
Diabetes mellitus type 2	281,908.84 (165,678.17 to 395,527.82)	3.32 (1.95 to 4.66)	17.47 (10.69 to 24.84)	0.03 (−0.05 to 0.10)	12,904,493.55 (7,501,414.23 to 19,485,253.91)	148.92 (86.50 to 224.91)	17.07 (10.48 to 24.30)	0.77 (0.71 to 0.83)
**Neoplasms**	374,212.72 (236,358.33 to 520,255.39)	4.34 (2.74 to 6.04)	3.73 (2.42 to 5.17)	−1.32 (−1.49 to −1.16)	8,934,120.30 (5,681,090.30 to 12,409,780.48)	102.08 (64.89 to 141.62)	3.45 (2.24 to 4.78)	−1.64 (−1.80 to −1.48)
Tracheal, bronchus, and lung cancer	374,212.72 (236,358.33 to 520,255.39)	4.34 (2.74 to 6.04)	18.47 (11.87 to 25.52)	−1.32 (−1.49 to −1.16)	8,934,120.30 (5,681,090.30 to 12,409,780.48)	102.08 (64.89 to 141.62)	19.15 (12.35 to 26.33)	−1.64 (−1.80 to −1.48)
**Sense organ diseases**	NA	NA	NA	NA	1,956,323.97 (−612,092.84 to 4,033,705.23)	22.84 (−7.15 to 47.05)	2.54 (−0.60 to 5.17)	−1.51 (−1.72 to −1.29)
Blindness and vision loss	NA	NA	NA	NA	1,956,323.97 (−612,092.84 to 4,033,705.23)	22.84 (−7.15 to 47.05)	6.83 (−1.66 to 14.11)	−1.51 (−1.72 to −1.29)
Cataract	NA	NA	NA	NA	1,956,323.97 (−612,092.84 to 4,033,705.23)	22.84 (−7.15 to 47.05)	29.81 (−7.97 to 57.77)	−1.51 (−1.72 to −1.29)
**Enteric infections**	6558.80 (4931.11 to 9244.79)	0.11 (0.08 to 0.15)	0.60 (0.41 to 0.85)	−6.20 (−6.49 to −5.92)	593,959.88 (446,774.05 to 835,347.37)	9.60 (7.22 to 13.50)	0.94 (0.73 to 1.26)	−6.19 (−6.47 to −5.91)
Diarrheal diseases	6558.80 (4931.11 to 9244.79)	0.11 (0.08 to 0.15)	0.70 (0.46 to 1.02)	−6.20 (−6.49 to −5.92)	593,959.88 (446,774.05 to 835,347.37)	9.60 (7.22 to 13.50)	1.15 (0.88 to 1.49)	−6.19 (−6.47 to −5.91)
**Other infectious diseases**	3715.75 (2867.22 to 4849.19)	0.06 (0.05 to 0.08)	0.74 (0.59 to 0.97)	−2.65 (−3.03 to −2.26)	334,394.98 (258,055.71 to 436,365.86)	5.40 (4.17 to 7.05)	0.91 (0.73 to 1.21)	−2.65 (−3.03 to −2.26)
Meningitis	3434.16 (2619.15 to 4550.79)	0.06 (0.04 to 0.07)	1.89 (1.59 to 2.38)	−2.70 (−3.11 to −2.29)	309,052.23 (235,708.82 to 409,534.00)	4.99 (3.81 to 6.62)	2.40 (2.01 to 3.03)	−2.70 (−3.11 to −2.29)
Encephalitis	281.58 (211.51 to 363.29)	0.00 (0.00 to 0.01)	0.38 (0.31 to 0.46)	−1.85 (−2.03 to −1.68)	25,342.75 (19,039.85 to 32,694.56)	0.41 (0.31 to 0.53)	0.61 (0.51 to 0.72)	−1.85 (−2.03 to −1.68)
**Other non-communicable diseases**	708.14 (371.35 to 1049.21)	0.01 (0.01 to 0.02)	0.07 (0.04 to 0.10)	−3.69 (−3.96 to −3.42)	63,713.25 (33,411.31 to 94,401.21)	1.03 (0.54 to 1.52)	0.05 (0.03 to 0.08)	−3.69 (−3.96 to −3.42)
Sudden infant death syndrome	708.14 (371.35 to 1049.21)	0.01 (0.01 to 0.02)	2.32 (1.84 to 2.83)	−3.69 (−3.96 to −3.42)	63,713.25 (33,411.31 to 94,401.21)	1.03 (0.54 to 1.52)	2.33 (1.84 to 2.83)	−3.69 (−3.96 to −3.42)

Abbreviations: DALYs, disability-adjusted life years. PMP, particulate matter pollution. APMP, ambient particulate matter pollution. HPMP, household particulate matter pollution. EAPCs, estimated annual percentage changes. PAFs, population attributable fractions.

#### Age- and gender-specific patterns in PMP-attributable deaths and DALYs.

**Figure 1:**
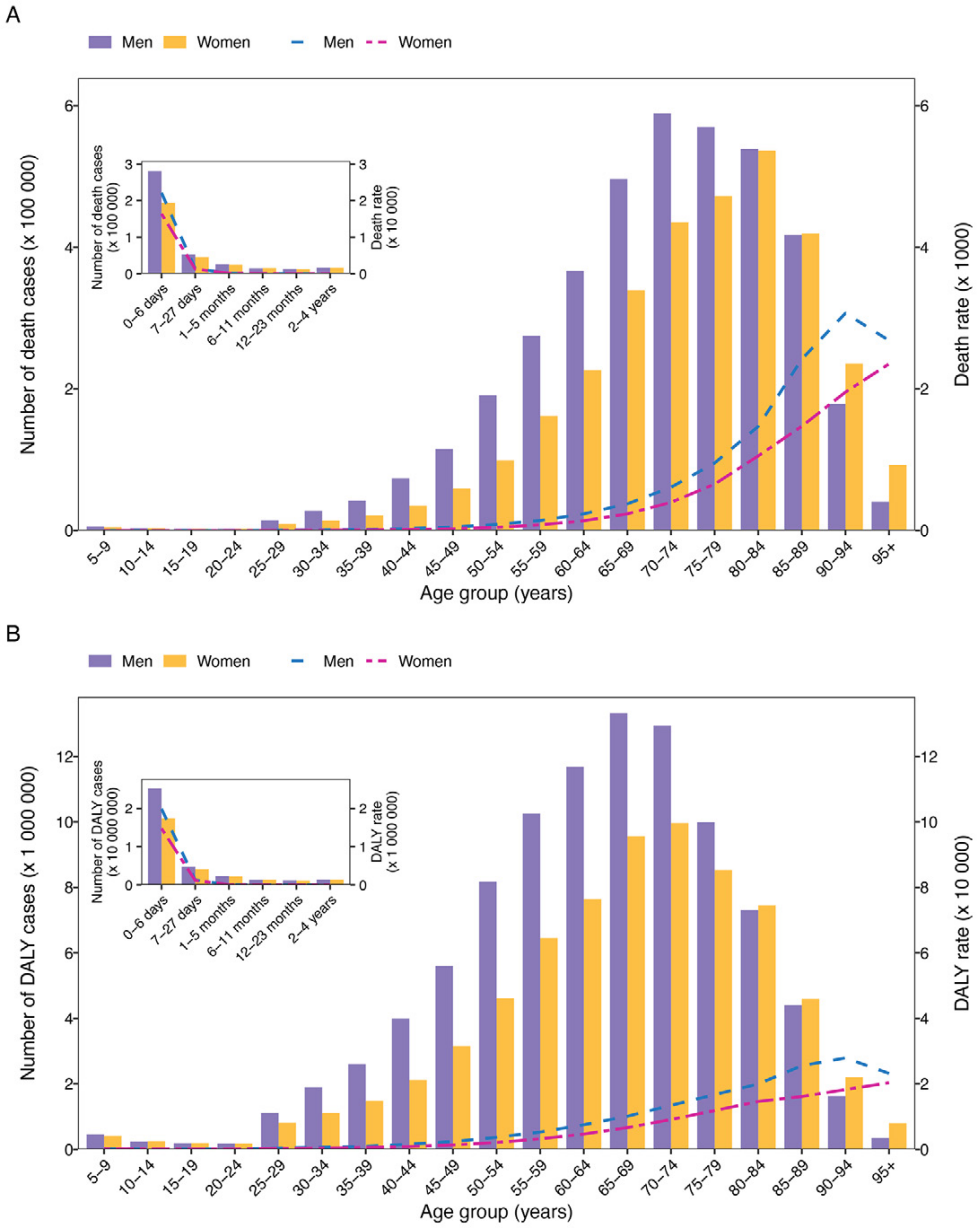
Age-specific numbers and rates of deaths and DALYs attributable to particulate matter pollution by sex in 2021. **(A)** Deaths attributable to particulate matter pollution. **(B)** DALYs attributable to particulate matter pollution. DALYs, disability-adjusted life years.

There were two peaks in PMP-attributable age-specific deaths: one at age 0–6 days for both sexes and the other at age 70–74 in males and 80–84 in females (Figure 1A). The pattern of DALYs was slightly different, with the other peak at age 65–69 in males and age 70–74 in females (Figure 1B). Before ages 85–89 for deaths and 80–84 for DALYs, males had higher numbers than females, but this trend reversed afterwards. Except for the 0–4 years age group, the age-specific rates of deaths and DALYs increased with age before age 90–94 in males, then declined, while they continually increased in females. Additionally, males consistently had higher age-specific rates of deaths and DALYs than females. APMP- and HPMP-attributable age-specific deaths and DALYs showed similar patterns (Figures S7 and S8).

#### Regional variations in PMP-attributable deaths and DALYs

Across the 21 GBD regions in 2021, the highest APMP-attributable ASRs of deaths (102.51 per 100,000 people) and DALYs (2,399.07) were observed in North Africa and the Middle East (Tables S7 and S8). The lowest rates were seen in high-income North America (7.75 deaths per 100,000 people) and Australasia (183.19 DALYs). The highest HPMP-attributable ASRs were in Oceania (219.79 deaths and 5,997.06 DALYs per 100,000 people [Tables S9 and S10]). The lowest HPMP-attributable rates were in high-income North America, Australasia, high-income Asia Pacific, and Western Europe. Overall, PMP burdens showed similar patterns to HPMP (Tables S11 and S12). The highest number of PMP-attributable deaths and DALYs occurred in East Asia and South Asia. From 1990 to 2021, South Asia had the largest increase in ASRs of APMP-attributable deaths (EAPC = 2.39%) and DALYs (1.74%), while high-income North America had the largest decrease (EAPC = −5.77% for deaths and −5.10% for DALYs) (Tables S7 and S8). All 21 GBD regions saw a decrease in ASRs of HPMP- and overall PMP-attributable deaths and DALYs (Tables S9–S12).

#### Country-specific trends in PMP-attributable deaths and DALYs

**Figure 2:**
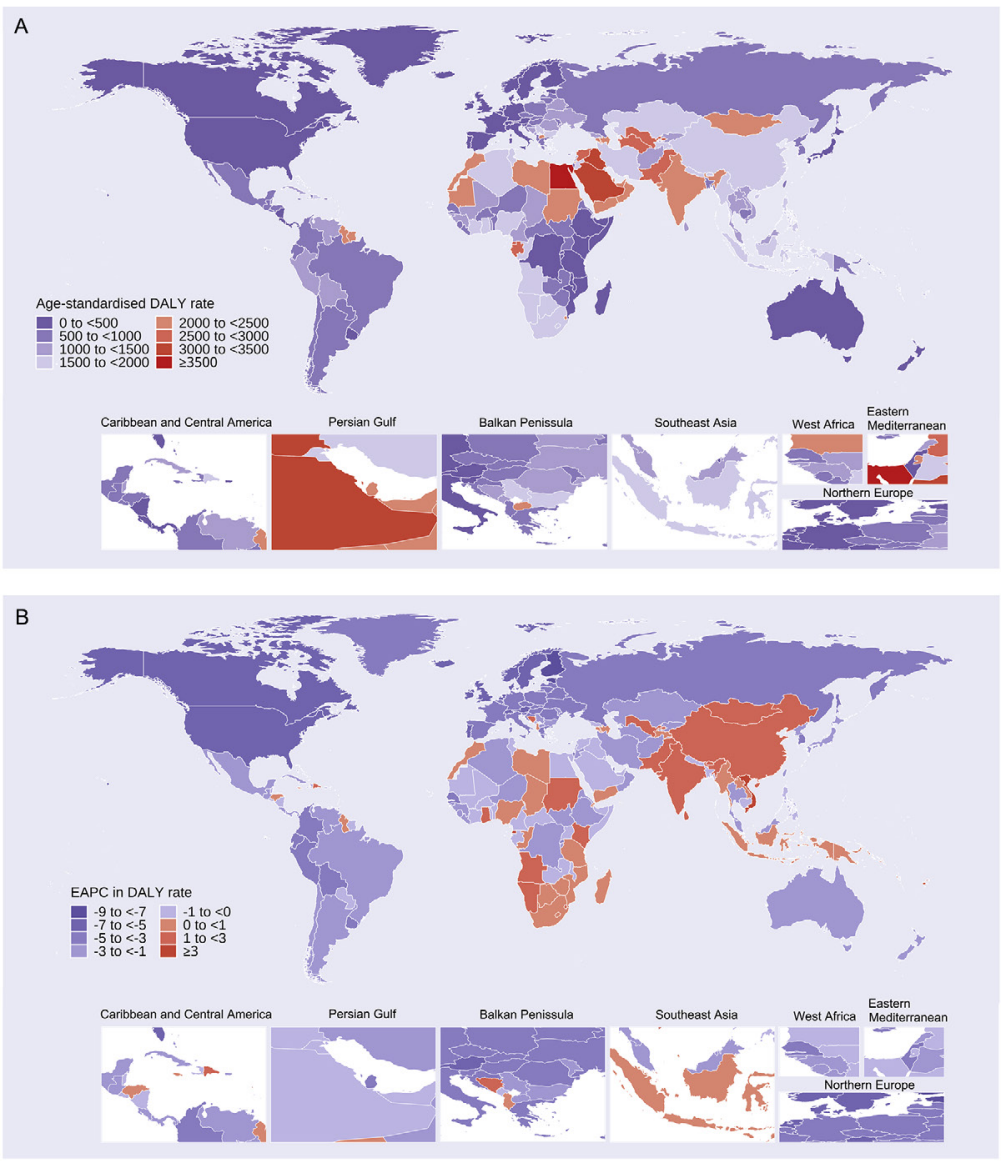
Age-standardized DALY rates and their EAPCs attributable to ambient particulate matter pollution by country. **(A)** Age-standardised DALY rates, in 2021. **(B)** EAPCs in age-standardised DALY rates, 1990–2021. DALYs, disability-adjusted life years. EAPCs, estimated annual percentage changes.

In 2021, Egypt had the highest ASRs of APMP-attributable DALYs and deaths among the world’s 20 most populous countries, with 5,279.18 DALYs and 251.38 deaths per 100,000 people (Tables S13 and S14, Figure 2A, Figure S9A). The United States of America had the lowest rates, with 8.20 deaths and 224.24 DALYs per 100,000 people. For HPMP, the highest rates were in the Solomon Islands (337.76 deaths per 100,000 people) and the Central African Republic (8,596.53 DALYs) (Tables S15 and S16, Figures S10 and S11A). Among populous countries, the Democratic Republic of the Congo had the highest ASRs, with 208.91 deaths and 5,369.44 DALYs per 100,000 people. Overall, PMP burdens mirrored those of HPMP (Tables S17 and S18, Figure S12 and S13). From 1990 to 2021, Viet Nam (one of the world’s 20 most populous countries) saw the largest increases in APMP-attributable deaths (EAPC = 5.13%) and DALYs (4.59%), while Estonia experienced the biggest decline (−8.31% for DALYs and −8.54% for deaths) (Tables S13 and S14, Figure 2B, Figure S9B). The United Kingdom had the largest decline among populous countries, with EAPCs of −5.69% for DALYs and −6.2% for deaths. Only the Northern Mariana Islands, Zimbabwe, and Lesotho saw increases in HPMP-attributable deaths and DALYs from 1990 to 2021 (Tables S15 and S16, Figures S10 and S11B).

### Impact of particulate matter pollution on each disease

#### Leading causes of PMP-attributable deaths and DALYs

In 2021, the leading four level 2 causes of PMP-attributable deaths were CVDs (53.62 per 100,000 people; PAF = 15.15% from APMP and 7.64% from HPMP), chronic respiratory diseases (CRDs) (18.51; 19.09% and 15.48%), respiratory infections and tuberculosis (8.68; 2.76% and 3.57%), and maternal and neonatal disorders (8.03; 7.12% and 18.01%) (Table 1, Tables S2 and S3). The leading causes of PMP-attributable DALYs were CVDs (1161.77; 14.65% and 8.33%), maternal and neonatal disorders (723.06; 6.61% and 16.73%), respiratory infections and tuberculosis (420.28; 3.29% and 6.34%), and CRDs (389.50; 16.09% and 14.01%) (Table 1, Tables S5 and S6). From 1990 to 2021, only diabetes and kidney diseases and neoplasms showed an increasing trend in age-standardized DALY rate attributable to APMP (Table S5). Other GBD level 2 causes of APMP-, HPMP-, and overall PMP-attributable deaths and DALYs are detailed in supplementary tables and figures (Tables S1–S6 and Figures S1–S6).

**Figure 3:**
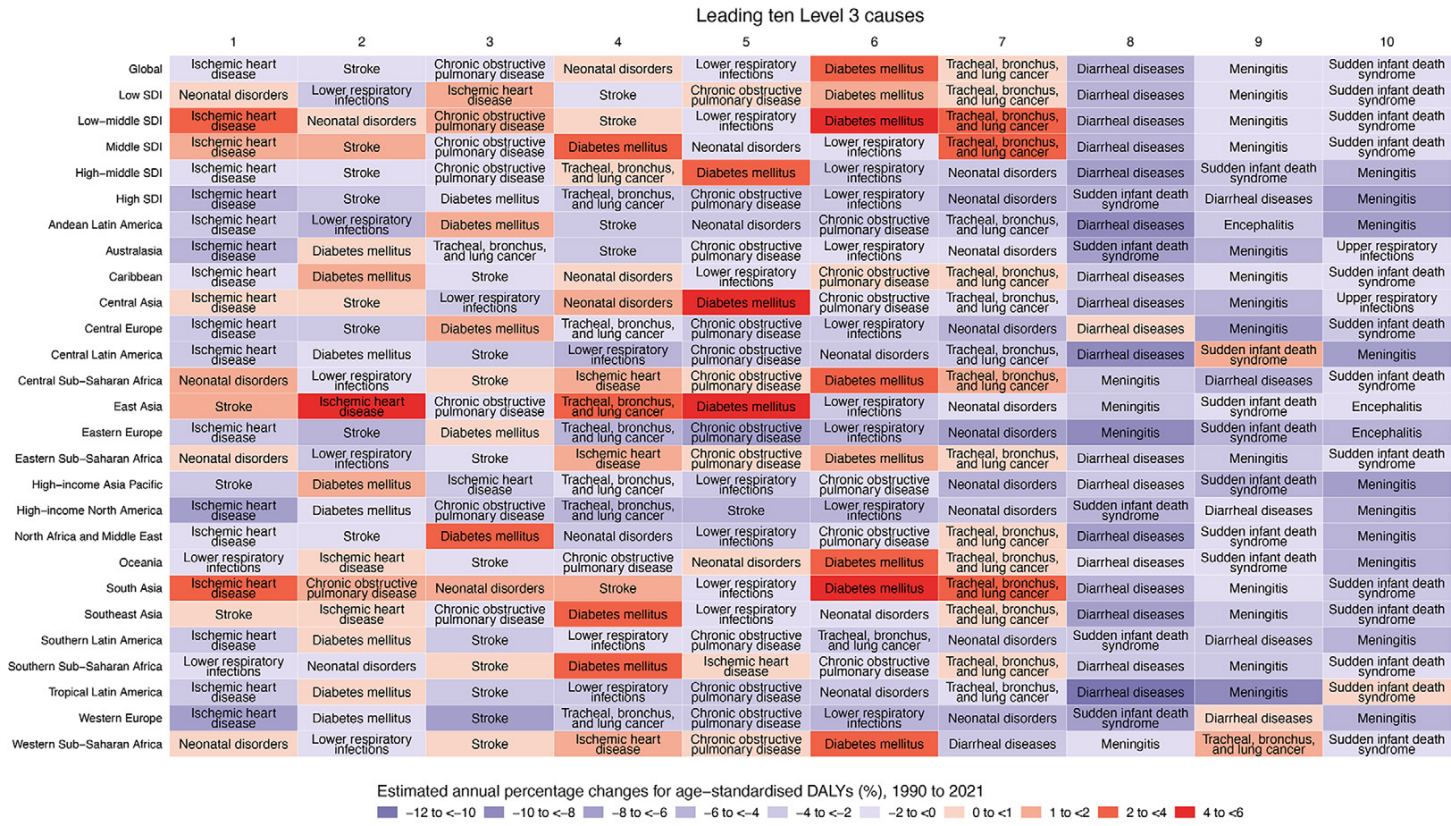
Estimated annual percentage changes in age-standardized DALY rates attributable to ambient particulate matter pollution, 1990–2021, for the leading ten level 3 attributable causes in 2021, by SDI quintile and GBD region. For each region and SDI quintile, level 3 causes are ranked by attributable DALY counts in 2021 from left (first) to right (tenth). Causes are colored by their estimated annual percentage changes in age-standardised DALY rates attributable to ambient particulate matter pollution, from 1990 to 2021. DALYs, disability-adjusted life years. SDI, socio-demographic index. GBD, Global Burden of Diseases, Injuries, and Risk Factors Study.

In 2021, among all GBD level 3 causes, the highest global age-standardized death rates attributable to PMP were for ischemic heart disease (IHD) (29.88 per 100,000 people; PAF = 19.18% from APMP and 8.30% from HPMP), stroke (23.74; 16.90% and 10.24%), and chronic obstructive pulmonary disease (COPD) (18.51; 22.61% and 18.33%) (Tables S1–S3). The highest global age-standardized DALY rates attributable to PMP were for neonatal disorders (723.06; 6.96% and 17.61%), IHD (638.48; 19.34% and 9.52%), stroke (523.30; 16.59% and 11.16%), lower respiratory infections (LRI) (420.09; 12.25% and 23.58%), COPD (389.50; 22.14% and 19.27%), diabetes mellitus (DM) (148.92; 11.23% and 5.01%), and tracheal, bronchus, and lung cancer (TBL) (102.08; 14.94% and 4.21%) (Tables S4–S6). From 1990 to 2021, only DM and TBL showed increases in age-standardized DALY rates attributable to APMP (Figure 3).

#### Regional, gender, and age group variations in PMP-attributable disease burdens

Figure 3 and Figures S14–S18 provide detailed visualization of the leading ten level 3 attributable causes in 2021 and their EAPCs in age-standardized DALY and death rates from 1990 to 2021 for each region. IHD had the highest PMP-attributable deaths and DALYs in high, high-middle, and middle SDI regions, whereas neonatal disorders had the highest burdens in low or low-middle SDI regions. From 1990 to 2021, burdens of DM, TBL, and IHD attributable to APMP significantly increased in middle-, low-middle, and low SDI regions. Only high SDI regions showed decreasing trends in ASRs of deaths and DALYs for the leading 10 causes attributable to PMPs.

**Figure 4:**
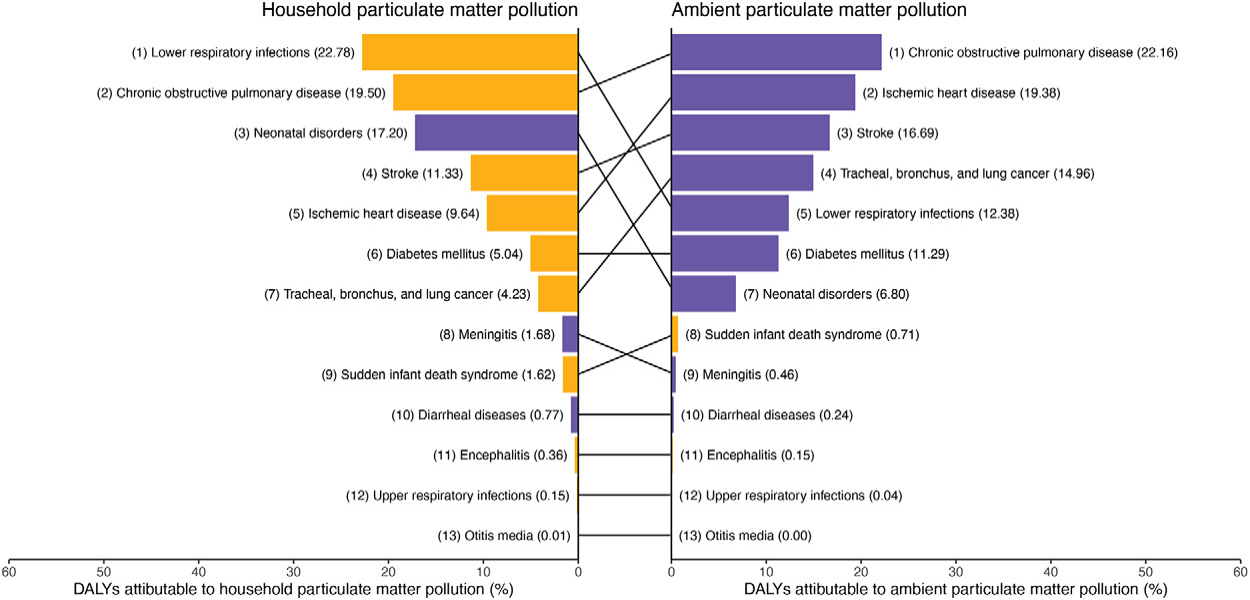
Global rankings of fractions of level 3 causes in age-standardized DALYs attributable to ambient and household particulate matter pollution globally in 2021. The list of causes of disease burden represents the level 3 causes of age-standardised DALYs (per 100,000 population) attributable to APMP and HPMP observed for both sexes globally in 2021. This same list of health conditions was ranked according to their PAFs for both APMP and HPMP globally in 2021. The colors of the bars denote whether the PAFs are higher for females (yellow) or males (purple) as established by whether the 95% uncertainty interval of the absolute difference in PAFs includes zero. DALYs, disability-adjusted life years. APMP, ambient particulate matter pollution. HPMP, household particulate matter pollution. PAFs, population attributable fractions.

The proportional contribution of APMP to age-standardized DALYs was highest in NCDs, with the leading three being COPD, IHD, and stroke (Figure 4). In contrast, HPMP contributed most to DALYs in LRI, COPD, and neonatal disorders. Cataract DALYs were solely attributable to HPMP (Table S6). For most GBD level 3 causes, the proportional contribution of APMP to age-standardized DALYs was higher in males than in females (9 of 13 GBD level 3 causes), except for sudden infant death syndrome (SIDS), encephalitis, upper respiratory infections (URI), and otitis media. Conversely, HPMP’s proportional contribution was higher in females (9 of 14 causes), except for neonatal disorders, meningitis, diarrheal diseases, and otitis media (Figure 4). Global rankings of fractions of level 3 causes in age-standardized deaths attributable to APMP and HPMP across females and males in 2021 were provided in Figure S19.

**Figure 5:**
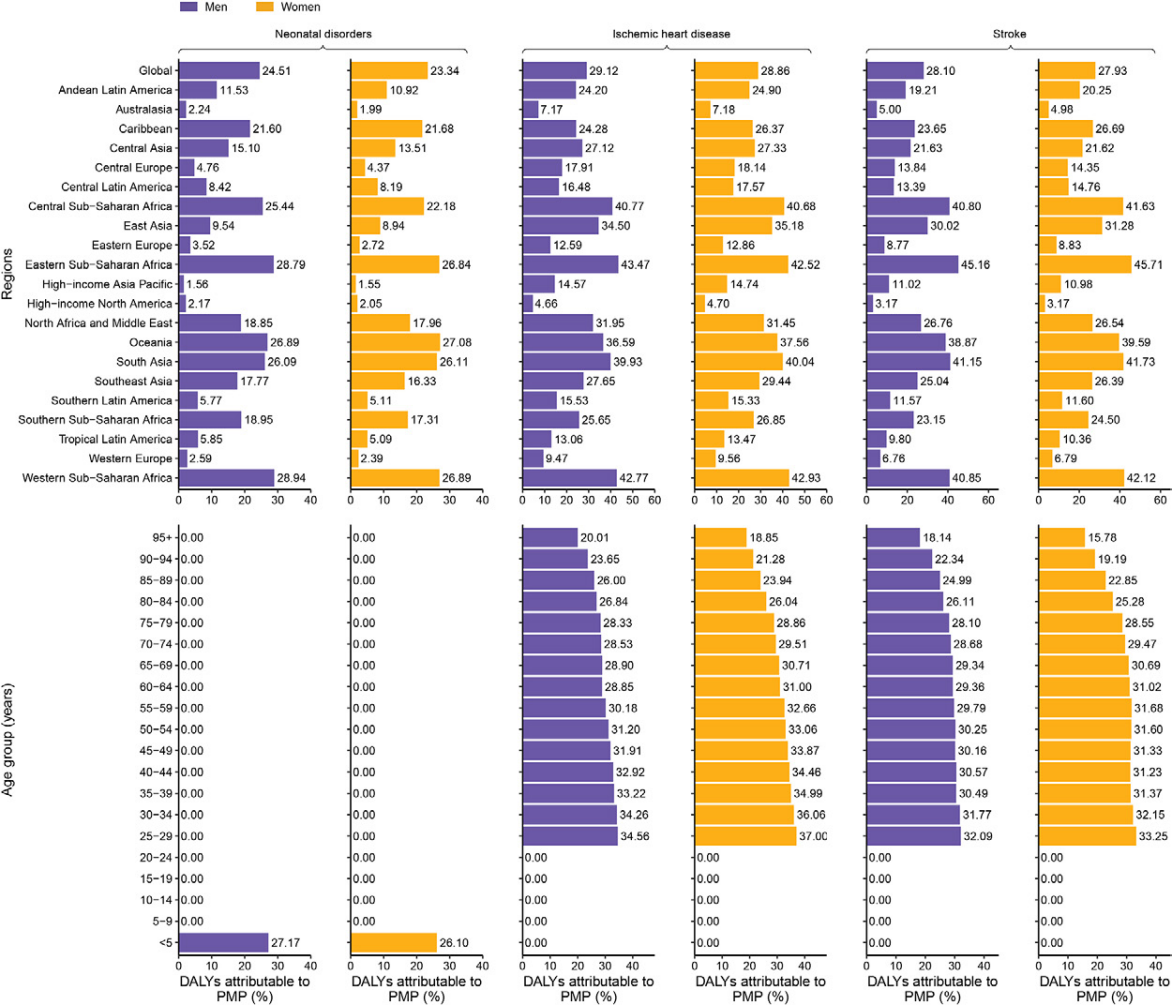
Fraction of neonatal disorders, ischemic heart disease, and stroke in age-standardized DALYs attributable to particulate matter pollution by region and by age group for females and males in 2021. **(A)** By region. **(B)** By age group. The three leading GBD level 3 causes of particulate matter pollution-attributable DALYs are shown. DALYs, disability-adjusted life years. GBD, Global Burden of Disease Study.

There were substantial differences in the attributable proportions of age-standardized DALYs due to PMPs across regions and age groups (Figure 5, Figures S20–S33). In 2021, Eastern and Western Sub-Saharan Africa had the highest attributable proportions of age-standardized DALYs due to PMP for the leading five GBD level 3 causes in both sexes, whereas high-income North America and high-income Asia Pacific had the lowest attributable proportions for the leading eight GBD level 3 causes in both sexes. By age group, notably, PMP was only associated with DALYs for neonatal disorders, diarrheal diseases, meningitis, SIDS, encephalitis, URI, and otitis media among children aged under 5 years.

### Relationship between SDI and the impact of particulate matter pollution on disease burden

**Figure 6:**
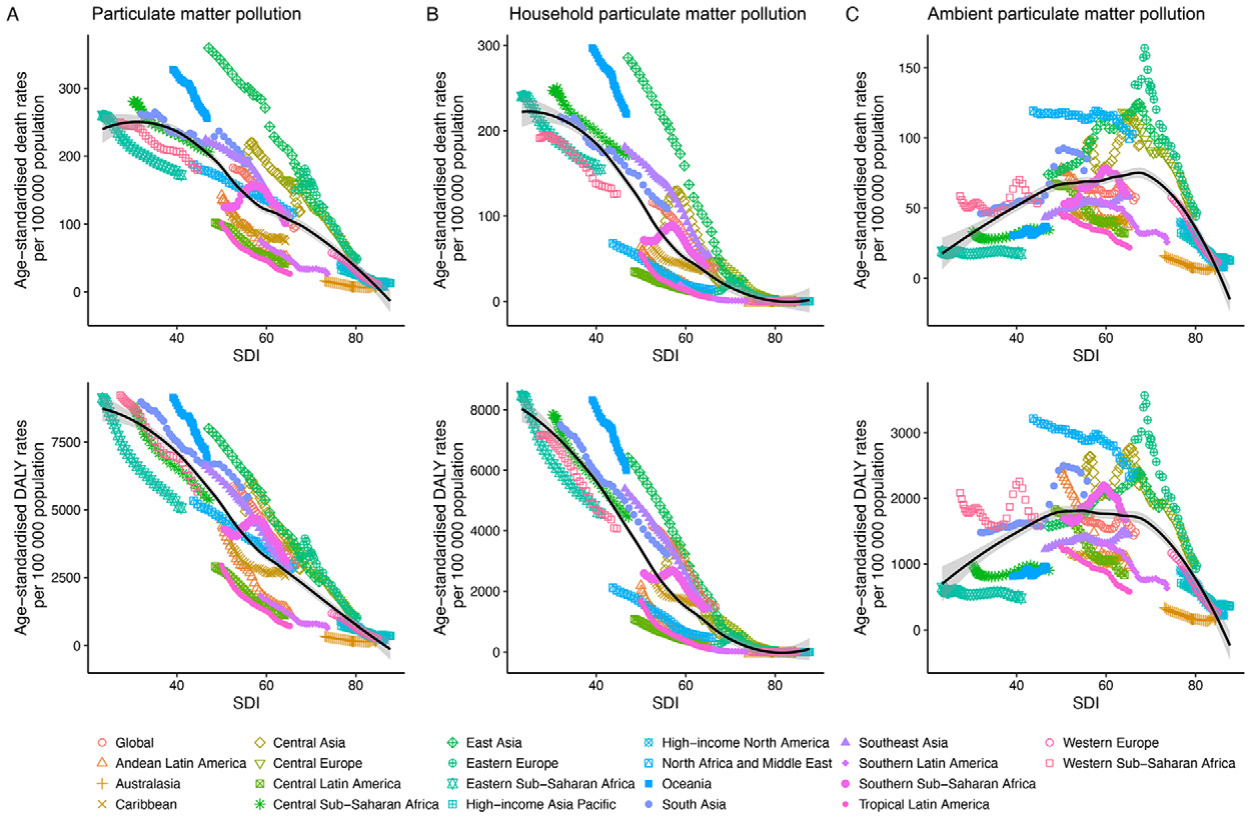
Age-standardized death and DALY rates attributable to particulate matter pollution across 21 GBD regions by socio-demographic index, 1990–2021. **(A)** Age-standardised death and DALY rates attributable to particulate matter pollution by SDI. **(B)** Age-standardised death and DALY rates attributable to household particulate matter pollution by SDI. **(C)** Age-standardised death and DALY rates attributable to ambient particulate matter pollution by SDI. For each region, points from left to right depict estimates from each year from 1990 to 2021. DALYs, disability-adjusted life years. GBD, Global Burden of Diseases, Injuries, and Risk Factors Study. SDI, socio-demographic index.

Figure 6 illustrates the changes in age-standardized death and DALY rates across SDI by region from 1990 to 2021. Both ASRs of overall PMP-attributable deaths and DALYs exhibited significantly negative trends with increasing SDI (Figure 6A). Similar patterns were observed for HPMP, with ASRs of HPMP-attributable deaths and DALYs approaching zero as SDI approached 80 (Figure 6B). However, APMP-attributable ASRs displayed an inverted U-shaped relationship with SDI (Figure 6C). Figures S34–S39 highlight the association between age-standardized death and DALY rates, their EAPCs, and SDI across countries in 2021, mirroring the patterns in Figure 6.

## Discussion

This study provides comprehensive estimates of global deaths and DALYs attributable to PMPs and examines their temporal trends. The key findings are as follows: First, from 1990 to 2021, global PMP-attributable deaths increased, primarily due to the doubling of APMP-attributable deaths. Despite a global decrease in PMP-attributable DALYs, APMP-attributable DALYs also doubled. Second, in 2021, age-specific death and DALY rates were consistently higher in males than in females. These rates peaked at two points: 0–6 days for both sexes, 90–94 years for males, and 95+ years for females. Third, from 1990 to 2021, SDI-related heterogeneity in disease burden was observed. Age-standardized death and DALY rates attributable to overall PMP and HPMP significantly decreased with increasing SDI, while those attributable to APMP showed an inverted U-shaped relationship with SDI. Fourth, in 2021, the leading diseases attributable to PMP were CVDs, maternal and neonatal disorders, respiratory infections and tuberculosis, and CRDs. APMP contributed most to age-standardized DALYs for COPD, IHD, and stroke, while HPMP had the greatest impact on LRI, COPD, and neonatal disorders. Finally, the burden of different GBD level 3 diseases attributable to PMP varied by region, gender, and age.

### Global burden attributable to ambient and household particulate matter pollution

Our study showed that in 2021, PMP posed a significant burden on global health, largely attributable to APMP, which has seen a substantial increase in both deaths and DALYs over the years. According to WHO population data and IHME reports, PMP-related deaths accounted for over one-tenth of global deaths and were a major cause of global DALYs [18, 19]. This aligns with a comprehensive analysis of 88 global risk factors, which identified PMP as the leading level 3 risk factor in 2021 and the primary contributor to the global health burden [4]. Initiatives under the United Nations (UN) Sustainable Development Goals (SDGs) to enhance access to clean household energy, such as grid electricity provision and cleaner cooking methods [20], have significantly reduced deaths and DALYs attributable to HPMP. However, the latest WHO data revealed that high levels of APMP persisted in large regions of the world, with 99% of the global population breathing air that exceeded WHO’s recommended air quality limits [21]. This may be mainly due to rapid industrialization and urbanization, especially in developing countries, leading to higher levels of APMP and increased urban population exposure, thus heightening the health burden [22]. Additionally, according to data from the UN and WHO, continuous global population growth and significant aging trends over the past 30 years have amplified the impact of PMP [9, 10].

Despite the increase in absolute deaths and DALYs caused by APMP, our study revealed that from 1990 to 2021, age-standardized death and DALY rates attributable to global PMP significantly declined. These divergent trends reflected the competing influences of population growth, aging, and increased global environmental exposure on the burden of disease attributable to PMP. Moreover, the decline in age-standardized death and DALY rates attributable to PMP can be attributed to several factors. First, over the past 30 years, significant efforts have been made globally to control PMP. WHO member states have implemented stringent air quality management policies and regulations, such as limiting industrial emissions, setting vehicle emission standards, and controlling emissions from coal-fired power plants [23, 24]. Second, continuous advancements in socioeconomic conditions and scientific research have led to improvements in air quality monitoring systems, the development of clean energy technologies, and advancements in medical technologies and facilities, all contributing to the reduction of PMP and its associated disease burden [25, 26]. Additionally, increased public awareness of PMP pollution and its health risks, coupled with a heightened emphasis on environmental protection, has helped mitigate exposure risks [27]. These demonstrated the global capacity to adapt and improve in the face of public health challenges. However, the persistently high burden of disease attributable to PMP underscored its continued significance not only among environmental risk factors but also in comparison to dietary, lifestyle, and other modifiable risk factors, indicating that global efforts to control PMP remain insufficient.

### Socio-demographic differences in household particulate matter pollution and ambient particulate matter pollution

Our study found that global age-standardized death and DALY rates attributable to HPMP decreased with increasing social development, likely due to differences in household fuel use and economic and sanitary conditions. In 2018, the Solomon Islands government initiated a pilot study to address cultural and developmental barriers in Oceania’s transition to clean energy practices, formulating a visionary energy transition plan that included clean energy for household cooking [27]. In 2017, the Central African Republic’s Ministry of Environment and Sustainable Development collaborated extensively with the Supporting National Planning initiative to establish comprehensive emission inventories and enhance the capacity to mitigate short-lived climate pollutants like black carbon and methane [28]. Similarly, in 2017, the Democratic Republic of Congo joined the Climate and Clean Air Coalition, striving to fulfill its international environmental commitments [29]. Despite these three countries having the highest global disease burden attributable to HPMP, their efforts have led to a significant decline in this burden. Our results indicated that 95% of countries and regions have seen a significant reduction in the global disease burden attributable to HPMP, demonstrating the feasibility of current global policies addressing HPMP. However, the Northern Mariana Islands, Zimbabwe, and Lesotho continued to experience the fastest increase in this burden. In 2021, the WHO provided a household energy policy repository, suggesting that these countries should learn from others and tailor their environmental pollution policies [24]. Additionally, these low SDI countries often have poorer medical technology and healthcare services, also contributing to a higher disease burden [30].

However, countries and regions with middle SDI levels exhibited the highest global age-standardized death and DALY rates attributable to APMP, consistent with the inverted U-shaped relationship in Figure 6C, while those with high SDI showed the lowest rates. Moreover, middle and lower SDI regions showed rising trends in these rates, highlighting disparities in global development. Rapid industrialization, urbanization, and inadequate environmental regulations contribute to severe pollution in these areas [31]. For instance, Eastern and Western sub-Saharan Africa had the highest proportion of age-standardized DALYs attributable to PMP-related diseases. The IHME also reported that PMP exposure rates in Asia, Africa, and the Middle East are nearly double the global average of 57.6 per 100,000 [15]. Nationally, our study identified Egypt as having the highest global disease burden attributable to APMP, with Viet Nam showing the fastest growth. According to a real-time air quality tool, air pollution levels in Cairo, Egypt, exceed acceptable global standards by tenfold [32]. In 2020, Egypt launched the 2030 Sustainable Development Strategy to reduce particulate emissions by 50% as part of efforts to tackle urban pollution [33]. According to the International Trade Administration, Viet Nam ranked 14th among 118 countries with the most severe air pollution in 2022. In the same year, Viet Nam introduced the National Environmental Protection Strategy 2030, aiming to prevent pollution escalation and environmental degradation by 2050 [34]. Additionally, among the top 20 populous countries, the United States of America had the lowest global disease burden attributable to APMP, with the United Kingdom showing the most significant decline. An observational study revealed that these high SDI countries typically benefit from high-quality healthcare systems, underscoring the critical need for research to improve healthcare in low- and middle-income countries [35]. It is imperative for low and middle SDI countries to learn from other countries’ experiences, seek international cooperation and assistance, strengthen environmental regulation and governance, and enhance preventive healthcare services for related diseases.

### Disparate impact of particulate matter pollution on diseases

Our study found significant heterogeneity in the spectrum of diseases attributable to PMP, with the leading level 2 causes being CVDs, maternal and neonatal disorders, respiratory infections and tuberculosis, and CRDs. Regarding CVDs, an exposure−response analysis supported our findings, linking long-term PMP exposure to increased hospital admissions for first-time IHD, cerebrovascular disease, heart failure, arrhythmias, cardiomyopathy, and thoracoabdominal aortic aneurysm, with the first four being particularly sensitive to PMP [36]. A meta-analysis of cohort studies revealed significant associations between several PMP components and CVD mortality, such as black carbon (BC), sulfate (SO_4_^2–^), nitrate (NO_3_^–^), nickel (Ni), and zinc (Zn), with BC having the most substantial impact [37]. These components may affect cardiovascular health through systemic inflammation, vasoconstriction, changes in cardiac electrical activity, and thrombosis [38]. Another prospective cohort study suggested organic matter (OM) and chloride within PMP mostly impact CVDs, with OM potentially driving metabolic syndrome factors like obesity, high triglycerides, and high blood pressure [39]. Additionally, a fixed cohort study that integrated targeted biomarkers and multi-omics results identified novel biomarkers, such as decay-accelerating factor, cholesterol ester transfer protein, and polyprotein-2, suggesting that ultrafine particles under 100 nm have unique mechanisms affecting cardiovascular health and may pose greater health risks than larger particles [40]. These studies implied that controlling specific PMP components and particle sizes could prevent CVDs and that intervening in metabolic risk factors may reduce the incidence of PMP-related CVDs.

### Disparate age group impact of particulate matter pollution

The global disease burden attributable to PMP exhibited age-related differences. Our study showed that one of the global peaks in deaths and DALYs occurs in the infant and young child population. The impact of PMP on infants differs from that on adults. According to the Health Effects Institute Special Report for global air, in 2021, PMP became the second largest risk factor for deaths and DALYs in children under five, including neonates [15]. Systematic reviews indicated that maternal exposure to air pollution increases the risk of miscarriage, preterm birth, and low birth weight [41]. Experimental evidence suggested that PMP exerts cytotoxic effects by mediating stress response pathways associated with inflammation, metabolic changes, and apoptosis, leading to organelle damage and oxidative stress [42, 43]. Numerous studies have shown that inhalation of PMP pollutants has systemic adverse effects on infant health, affecting multiple organs, various cell types, and molecular mediators, thereby impairing their defenses against infections [44–46]. Our study also revealed that DALYs attributable to neonatal diseases, diarrheal diseases, meningitis, SIDS, encephalitis, URI, and otitis media occur only in children under five, with LRI being the leading cause of death in this age group. Moreover, prenatal and early childhood exposure to PMP increases lifelong risks of cancer and other diseases [1], including cardiovascular issues from secondhand smoke [47]. The UNSDGs should advocate substantially reducing under-five mortality, especially in low SDI regions. Healthcare professionals and the public should address air pollution, particularly household air pollution, by adopting clean cooking and heating fuels and lighting technologies to benefit maternal and infant health.

Another global peak in deaths and DALYs occurred in the elderly, who bear the heaviest burden of PMP-attributable NCDs such as COPD, diabetes, stroke, heart disease, lung cancer, and chronic lung diseases. Previous studies have established that COPD is one of the leading causes of morbidity and mortality among the elderly, with air pollution contributing to approximately 50% of COPD risk [48]. Meta-analyses showed that certain PMP components (NO_3_^–^, silicon, and Zn) are significantly associated with respiratory disease mortality, with NO_3_^–^ having the greatest impact [37]. Moreover, our results also indicated a significant impact of PMP on COPD DALYs in younger populations, and recent studies suggested that combined exposure to household air pollution and ozone exacerbates COPD risk in young people [49]. This underscores the importance of early COPD diagnosis in younger individuals to facilitate timely pharmacological interventions and the formulation of preventive and therapeutic measures based on pollutant components to slow disease progression and achieve better outcomes. According to the WHO, IHD, stroke, and COPD are the top three global causes of death, with lung cancer and diabetes among the top ten [50]. Air pollution significantly contributes to the high burden of these chronic NCDs, and individuals with these diseases are, in turn, more susceptible to PMP’s toxic effects [51]. Moreover, our findings revealed that from 1990 to 2021, the burden of NCDs increased, with diabetes and tracheal, bronchial, and lung cancer showing upward trends in APMP-attributable age-standardized DALY rates. Multi-omics studies also highlighted the increasingly significant relationship between PMP and metabolic diseases, including type 2 diabetes mellitus, suggesting that PMP contributes to a growing number of chronic disease cases, leading to productivity loss and an overburdened healthcare system [52]. In 2019, the World Health Assembly included air pollution as a risk factor in the NCD framework [53], which should prompt more public health actions at the societal level, particularly among the elderly. Additionally, health education for individuals and families is crucial to mitigate lifestyle risk factors like poor diet, physical inactivity, smoking, and excessive alcohol consumption on PMP-related disease burden.

### Disparate gender impact of particulate matter pollution

Furthermore, the global disease burden of PMP exhibited gender differences. Our findings showed that overall death and DALY rates are consistently higher in males than females, with APMP contributing a higher proportion of age-standardized DALYs in males for most GBD level 3 causes. A meta-analysis of cohort studies supported our results, indicating that PMP components had a greater impact on males [37]. However, for specific diseases such as diabetes, PMP exposure had a more pronounced association in females [54]. A qualitative evaluation study showed that females, particularly the elderly or those assessed for residential exposure, are more strongly affected [55]. Studies on children indicated that boys were more affected in early life, while girls were more affected in later childhood. It remains unclear whether these observed differences are due to gender-related biological responses (e.g., hormone composition, body size), behavioral patterns (e.g., smoking), or exposure patterns (e.g., high-risk occupations, household cooking). More research should focus on emerging methods for quantitative gender analysis to elucidate the roots of these differences, enabling targeted interventions to control PMP and mitigate its adverse effects, thereby improving population health across different societies and genders.

### Strengths and limitations of the study

This study is the first to comprehensively evaluate the levels and trends of deaths and DALYs attributable to PMP at global, regional, and national levels from 1990 to 2021, distinguishing between disease burdens attributable to APMP and HPMP. It also provided an in-depth analysis of the burden differences across various diseases, genders, and age groups. However, this study has several limitations. First, the GBD study estimated PMP exposure based on population-weighted annual mean concentrations, assuming spatial uniformity of PMP components, which vary by source and season, potentially affecting the effect size of PMP [4]. Second, the GBD study estimated PMP levels based on the proportion of households using solid fuels for cooking, combined with data from household and personal exposure measurement studies, but did not account for exposures from solid fuels used for heating, boiling water, or other residential tasks, nor exposures from burning liquid fuels (e.g., kerosene), potentially underestimating total exposure and disease burden in certain locations [4]. Third, due to a lack of universally accepted case-finding methods and limited clinical healthcare access, particularly in low- and middle-income countries, some diseases may be underdiagnosed [13]. Fourth, the role of PMP in other diseases, such as neurodegenerative diseases (e.g., Alzheimer’s) and chronic kidney disease, is still being researched, and these diseases are not currently included in GBD estimates [56, 57]. Fifth, given the high prevalence of comorbidities in the elderly population, the GBD study may overestimate the overall level of DALYs in this demographic [12].

## Conclusion

This study found that global age-standardized death and DALY rates from overall PMP have declined, but the absolute number of deaths and DALYs from APMP has doubled due to population growth, aging, and increased PMP exposure. The disease burden from PMP varies by region, age, gender, and cause. Our findings highlight the urgent need to improve air quality, especially in low and middle SDI countries. We recommend better monitoring of PMP components and stricter control standards. Protective measures should be prioritized for vulnerable groups, including pregnant women, infants, children, and the elderly. Further research on PMP-related diseases is needed to improve prevention and healthcare strategies.

## Data Availability

Data used for the analyses are publicly available from the Institute of Health Metrics and Evaluation (http://www.healthdata.org/; http://ghdx.healthdata.org/gbd-results-tool).

## Declarations

Ethics approval and consent to participate: The University of Washington Institutional Review Board reviewed and approved a waiver of informed consent because GBD 2021 uses de-identified, aggregated data (https://www.healthdata.org/research-analysis/gbd).

## Additional File

The additional file for this article can be found as follows:

Supplementary Materials. Supplementary appendix.

This finding of multiple local introgression events has at least three key implications.
