# Supplementary material for: Global, Regional, and National Burden Attributed to Particulate Matter Pollution, 1990–2021: A Systematic Analysis for the Global Burden of Disease Study 2021
Source: Ann Glob Health. 2026 Feb 24;92(1):22. doi: 10.5334/aogh.4965 (PMC12947824; doi:10.5334/aogh.4965)
Supplement: Supplementary Materials. — Supplementary appendix. [file agh-92-1-4965-s1.zip › agh-92-1-4965-s1/Supplementary Materials.docx]

**Supplementary appendix**

**Global, regional, and national burden attributed to particulate matter pollution, 1990–2021: a systematic analysis for the Global Burden of Disease Study 2021**

**Contents**

[Appendix 1. Global Burden of Diseases, Injuries, and Risk Factors Study 2021 5](#_Toc174872428)

[Appendix 2. GBD 2021 ambient particulate matter pollution-specific modelling descriptions 5](#_Toc174872429)

[Appendix 3. GBD 2021 household particulate matter pollution-specific modelling descriptions 8](#_Toc174872430)

[Table S1. Global deaths attributable to particulate matter pollution in 1990 and 2021, and estimated annual percentage changes from 1990 to 2021. 11](#_Toc174872431)

[Table S2. Global deaths attributable to ambient particulate matter pollution in 1990 and 2021, and estimated annual percentage changes from 1990 to 2021. 13](#_Toc174872432)

[Table S3. Global deaths attributable to household particulate matter pollution in 1990 and 2021, and estimated annual percentage changes from 1990 to 2021. 16](#_Toc174872433)

[Table S4. Global DALYs attributable to particulate matter pollution in 1990 and 2021, and estimated annual percentage changes from 1990 to 2021. 19](#_Toc174872434)

[Table S5. Global DALYs attributable to ambient particulate matter pollution in 1990 and 2021, and estimated annual percentage changes from 1990 to 2021. 22](#_Toc174872435)

[Table S6. Global DALYs attributable to household particulate matter pollution in 1990 and 2021, and estimated annual percentage changes from 1990 to 2021. 25](#_Toc174872436)

[Table S7. Number and age-standardised rates of deaths attributable to ambient particulate matter pollution in 1990 and 2021, and estimated annual percentage changes from 1990 to 2021, by region. 29](#_Toc174872437)

[Table S8. Number and age-standardised rates of DALYs attributable to ambient particulate matter pollution in 1990 and 2021, and estimated annual percentage changes from 1990 to 2021, by region. 31](#_Toc174872438)

[Table S9. Number and age-standardised rates of deaths attributable to household particulate matter pollution in 1990 and 2021, and estimated annual percentage changes from 1990 to 2021, by region. 33](#_Toc174872439)

[Table S10. Number and age-standardised rates of DALYs attributable to household particulate matter pollution in 1990 and 2021, and estimated annual percentage changes from 1990 to 2021, by region. 35](#_Toc174872440)

[Table S11. Number and age-standardised rates of deaths attributable to particulate matter pollution in 1990 and 2021, and estimated annual percentage changes from 1990 to 2021, by region. 38](#_Toc174872441)

[Table S12. Number and age-standardised rates of DALYs attributable to particulate matter pollution in 1990 and 2021, and estimated annual percentage changes from 1990 to 2021, by region. 40](#_Toc174872442)

[Table S13. Number and age-standardised rates of DALYs attributable to ambient particulate matter pollution in 1990 and 2021, and estimated annual percentage changes from 1990 to 2021, by country. 42](#_Toc174872443)

[Table S14. Number and age-standardised rates of deaths attributable to ambient particulate matter pollution in 1990 and 2021, and estimated annual percentage changes from 1990 to 2021, by country. 56](#_Toc174872444)

[Table S15. Number and age-standardised rates of deaths attributable to household particulate matter pollution in 1990 and 2021, and estimated annual percentage changes from 1990 to 2021, by country. 67](#_Toc174872445)

[Table S16. Number and age-standardised rates of DALYs attributable to household particulate matter pollution in 1990 and 2021, and estimated annual percentage changes from 1990 to 2021, by country. 78](#_Toc174872446)

[Table S17. Number and age-standardised rates of deaths attributable to particulate matter pollution in 1990 and 2021, and estimated annual percentage changes from 1990 to 2021, by country. 91](#_Toc174872447)

[Table S18. Number and age-standardised rates of DALYs attributable to particulate matter pollution in 1990 and 2021, and estimated annual percentage changes from 1990 to 2021, by country. 102](#_Toc174872448)

[Figure S1. Numbers of all-age deaths and DALYs attributable to particulate matter pollution by sex, 1990–2021. 117](#_Toc174872449)

[Figure S2. Numbers of all-age deaths and DALYs attributable to ambient particulate matter pollution by sex, 1990–2021. 118](#_Toc174872450)

[Figure S3. Numbers of all-age deaths and DALYs attributable to household particulate matter pollution by sex, 1990–2021. 119](#_Toc174872451)

[Figure S4. Age-standardised death and DALY rates attributable to particulate matter pollution by sex, 1990–2021. 120](#_Toc174872452)

[Figure S5. Age-standardised death and DALY rates attributable to ambient particulate matter pollution by sex, 1990–2021. 121](#_Toc174872453)

[Figure S6. Age-standardised death and DALY rates attributable to household particulate matter pollution by sex, 1990–2021. 122](#_Toc174872454)

[Figure S7. Age-specific numbers and rates of deaths and DALYs attributable to ambient particulate matter pollution by sex, in 2021. 123](#_Toc174872455)

[Figure S8. Age-specific numbers and rates of deaths and DALYs attributable to household particulate matter pollution by sex, in 2021. 124](#_Toc174872456)

[Figure S9. Age-standardised death rates and their EAPCs attributable to ambient particulate matter pollution by country. 125](#_Toc174872457)

[Figure S10. Age-standardised death rates and their EAPCs attributable to household particulate matter pollution by country. 126](#_Toc174872458)

[Figure S11. Age-standardised DALY rates and their EAPCs attributable to household particulate matter pollution by country. 127](#_Toc174872459)

[Figure S12. Age-standardised death rates and their EAPCs attributable to particulate matter pollution by country. 128](#_Toc174872460)

[Figure S13. Age-standardised DALY rates and their EAPCs attributable to particulate matter pollution by country. 129](#_Toc174872461)

[Figure S14. Estimated annual percentage changes in age-standardised death rate attributable to ambient particulate matter pollution, 1990–2021, for the leading ten level 3 attributable causes in 2021, by SDI quintile and GBD region. 130](#_Toc174872462)

[Figure S15. Estimated annual percentage changes in age-standardised death rate attributable to household particulate matter pollution, 1990–2021, for the leading ten level 3 attributable causes in 2021, by SDI quintile and GBD region. 131](#_Toc174872463)

[Figure S16. Estimated annual percentage changes in age-standardised DALY rate attributable to household particulate matter pollution, 1990–2021, for the leading ten level 3 attributable causes in 2021, by SDI quintile and GBD region. 132](#_Toc174872464)

[Figure S17. Estimated annual percentage changes in age-standardised death rate attributable to particulate matter pollution, 1990–2021, for the leading ten level 3 attributable causes in 2021, by SDI quintile and GBD region. 133](#_Toc174872465)

[Figure S18. Estimated annual percentage changes in age-standardised DALY rate attributable to particulate matter pollution, 1990–2021, for the leading ten level 3 attributable causes in 2021, by SDI quintile and GBD region. 134](#_Toc174872466)

[Figure S19. Global rankings of fractions of level 3 causes in age-standardised deaths attributable to ambient and household particulate matter pollution globally in 2021 135](#_Toc174872467)

[Figure S20. Fraction of lower respiratory infections, chronic obstructive pulmonary disease, and diabetes mellitus in age-standardised DALYs attributable to particulate matter pollution by region and by age group for females and males in 2021. 136](#_Toc174872468)

[Figure S21. Fraction of tracheal, bronchus, and lung cancer, blindness and vision loss, and diarrheal diseases in age-standardised DALYs attributable to particulate matter pollution by region and by age group for females and males in 2021. 137](#_Toc174872469)

[Figure S22. Fraction of meningitis, sudden infant death syndrome, and encephalitis in age-standardised DALYs attributable to particulate matter pollution by region and by age group for females and males in 2021. 138](#_Toc174872470)

[Figure S23. Fraction of upper respiratory infections and otitis media in age-standardised DALYs attributable to particulate matter pollution by region and by age group for females and males in 2021. 139](#_Toc174872471)

[Figure S24. Fraction of ischemic heart disease, stroke, and chronic obstructive pulmonary disease in age-standardised DALYs attributable to ambient particulate matter pollution by region and by age group for females and males in 2021. 140](#_Toc174872472)

[Figure S25. Fraction of neonatal disorders, lower respiratory infections, and diabetes mellitus in age-standardised DALYs attributable to ambient particulate matter pollution by region and by age group for females and males in 2021. 141](#_Toc174872473)

[Figure S26. Fraction of tracheal, bronchus, and lung cancer, diarrheal diseases, and meningitis in age-standardised DALYs attributable to ambient particulate matter pollution by region and by age group for females and males in 2021. 142](#_Toc174872474)

[Figure S27. Fraction of sudden infant death syndrome, encephalitis, and upper respiratory infections in age-standardised DALYs attributable to ambient particulate matter pollution by region and by age group for females and males in 2021. 143](#_Toc174872475)

[Figure S28. Fraction of Otitis media in age-standardised DALYs attributable to ambient particulate matter pollution by region and by age group for females and males in 2021. 144](#_Toc174872476)

[Figure S29. Fraction of neonatal disorders, lower respiratory infections, and ischemic heart disease in age-standardised DALYs attributable to household particulate matter pollution by region and by age group for females and males in 2021. 145](#_Toc174872477)

[Figure S30. Fraction of stroke, chronic obstructive pulmonary disease, and diabetes mellitus in age-standardised DALYs attributable to household particulate matter pollution by region and by age group for females and males in 2021. 146](#_Toc174872478)

[Figure S31. Fraction of blindness and vision loss, tracheal, bronchus, and lung cancer, and diarrheal diseases in age-standardised DALYs attributable to household particulate matter pollution by region and by age group for females and males in 2021. 147](#_Toc174872479)

[Figure S32. Fraction of meningitis, sudden infant death syndrome, and encephalitis in age-standardised DALYs attributable to household particulate matter pollution by region and by age group for females and males in 2021. 148](#_Toc174872480)

[Figure S33. Fraction of upper respiratory infections and otitis media in age-standardised DALYs attributable to household particulate matter pollution by region and by age group for females and males in 2021. 149](#_Toc174872481)

[Fig S34. Age-standardised death and DALY rates attributable to particulate matter pollution across 204 countries and territories by socio-demographic index in 2021. 150](#_Toc174872482)

[Fig S35. Age-standardised death and DALY rates attributable to household particulate matter pollution across 204 countries and territories by socio-demographic index in 2021. 151](#_Toc174872483)

[Fig S36. Age-standardised death and DALY rates attributable to ambient particulate matter pollution across 204 countries and territories by socio-demographic index in 2021. 152](#_Toc174872484)

[Fig S37. Estimated annual percentage changes in age-standardised death and DALY rates attributable to particulate matter pollution across 204 countries and territories by socio-demographic index in 2021. 153](#_Toc174872485)

[Fig S38. Estimated annual percentage changes in age-standardised death and DALY rates attributable to household particulate matter pollution across 204 countries and territories by socio-demographic index in 2021. 154](#_Toc174872486)

[Fig S39. Estimated annual percentage changes in age-standardised death and DALY rates attributable to ambient particulate matter pollution across 204 countries and territories by socio-demographic index in 2021. 155](#_Toc174872487)

Appendix 1. Global Burden of Diseases, Injuries, and Risk Factors Study 2021

The Global Burden of Diseases, Injuries, and Risk Factors Study (GBD) is a comprehensive research initiative aimed at systematically assessing the health status and disease burden of populations worldwide. An international network comprising over 11,500 collaborators from 164 countries and territories contributed to GBD 2021, which includes more than 607 billion results derived from over 300,000 data sources, covering 288 causes of death, 371 diseases and injuries, and 88 risk factors in 204 countries and territories. GBD data collection involves diverse sources, including epidemiological surveys, hospital records, vital registration systems, disease surveillance systems, and additional sources such as academic papers and policy reports (https://ghdx.healthdata.org/gbd-2021/sources). The data is standardised using the International Classification of Diseases (ICD) codes to ensure accuracy and comparability (https://ghdx.healthdata.org/record/ihme-data/gbd-2021-cause-icd-code-mappings). Sophisticated modeling tools, such as DisMod-MR and Spatiotemporal Gaussian Process Regression (ST-GPR), are employed to estimate prevalence, incidence, and mortality rates. Data processing includes corrections for heterogeneity and biases, as well as uncertainty analysis through Monte Carlo simulations (https://www.healthdata.org/gbd/methods-appendices-2021). Dissemination of GBD findings is achieved through scientific publications (https://www.healthdata.org/research-analysis/gbd-publications), and interactive tools like GBD Compare and Viz Hub (https://www.healthdata.org/research-analysis/gbd-data). These tools facilitate the exploration and comparison of health data across regions and time periods. The primary goal of GBD findings is to provide a comprehensive framework for understanding global and local health trends, thereby supporting evidence-based health decision-making and resource allocation.

Appendix 2. GBD 2021 ambient particulate matter pollution-specific modelling descriptions

*Definition*

Exposure to ambient particulate matter pollution (APMP) is defined as the population-weighted annual average mass concentration of particles with an aerodynamic diameter less than 2.5 micrometers (PM_2.5_) in a cubic meter of air. This measurement is reported in ug/m^3^.

*Input data*

Ambient air pollution exposure estimates use input data from multiple sources. These include satellite observations of aerosols in the atmosphere, ground monitor measurements, chemical transport model simulations, population estimates, and land-use data.

*Modelling strategy*

The following is a summary of the modelling approach, known as the Data Integration Model for Air Quality (DIMAQ) used in GBD 2015, 2016, 2017, 2019, and 2020^1,2^.

Before the implementation of DIMAQ in GBD 2010 and 2013, exposure estimates were obtained using single global function to calibrate available ground measurements to a “fused” estimate of PM_2.5_: the mean of satellite-based estimates and those from the TM5 chemical transport model, calculated for each 0.1°× 0.1° grid cell. This approach was recognised to represent a trade-off between accuracy and computational efficiency when utilising all the available data sources. In particular, the GBD 2013 exposure estimates were known to underestimate ground measurements in specific locations (see discussion in Brauer et al., 2015)^3^. This underestimation was largely due to the use of a single, global calibration function, whereas in reality, the relationship between ground measurements and other variables varies spatially.

In GBD 2015 and 2016, coefficients in the calibration model were estimated for each country through DIMAQ. Where data were insufficient within a country, information was “borrowed” from a region-level aggregation, and where information was still insufficient, from the super-region-level aggregation. Individual country-level estimates were therefore based on a combination of information from the country and its region and super-region. This was implemented within a Bayesian hierarchical modelling (BHM) framework. BHMs provide an extremely useful and flexible framework in which to model complex relationships and dependencies in data. Uncertainty can also be propagated through the model, allowing uncertainty arising from different components (both data sources and models) to be incorporated within estimates of uncertainty associated with the final estimates. The results of the modelling comprise a posterior distribution for each grid cell, rather than just a single point estimate, allowing a variety of summaries to be calculated. The primary outputs for this process are the median and 95% uncertainty intervals for each grid cell. Based on the availability of ground measurement data, modelling and evaluation were focused on the year 2016.

The model used from GBD 2017 onward (GBD 2017, 2019, and now 2021) also included within-country calibration variation^4^. This model, henceforth referred to as DIMAQ2, provides a number of substantial improvements over the initial formulation of DIMAQ. In DIMAQ, ground measurements from different years were all assumed to have been made in the primary year of interest and then regressed against values from other inputs (satellites, etc.) made in that year. In the presence of changes over time, therefore, and particularly in areas where no recent measurements were available, there was the possibility of mismatches between the ground measurements and other variables. In DIMAQ2, ground measurements are matched with other inputs (over time), and the (global-level) coefficients are allowed to vary over time, subject to smoothing that is induced by a first-order random walk process. In addition, the manner in which spatial variation can be incorporated within the model has developed: where there are sufficient data, the calibration equations can now vary (smoothly) both within and between countries, achieved by allowing the coefficients to follow (smooth) Gaussian processes. Where there are insufficient data within a country, to produce accurate equations, information is borrowed as before from lower down the hierarchy and is supplemented with information from the wider region.

DIMAQ2 as described above was used for all regions except for the North Africa/Middle East and sub-Saharan Africa super-regions, where there are insufficient data across years to allow the extra complexities of the new model to be implemented. In these super-regions, a simplified version of DIMAQ2 is used in which the temporal component is dropped.

*Flowchart*

**
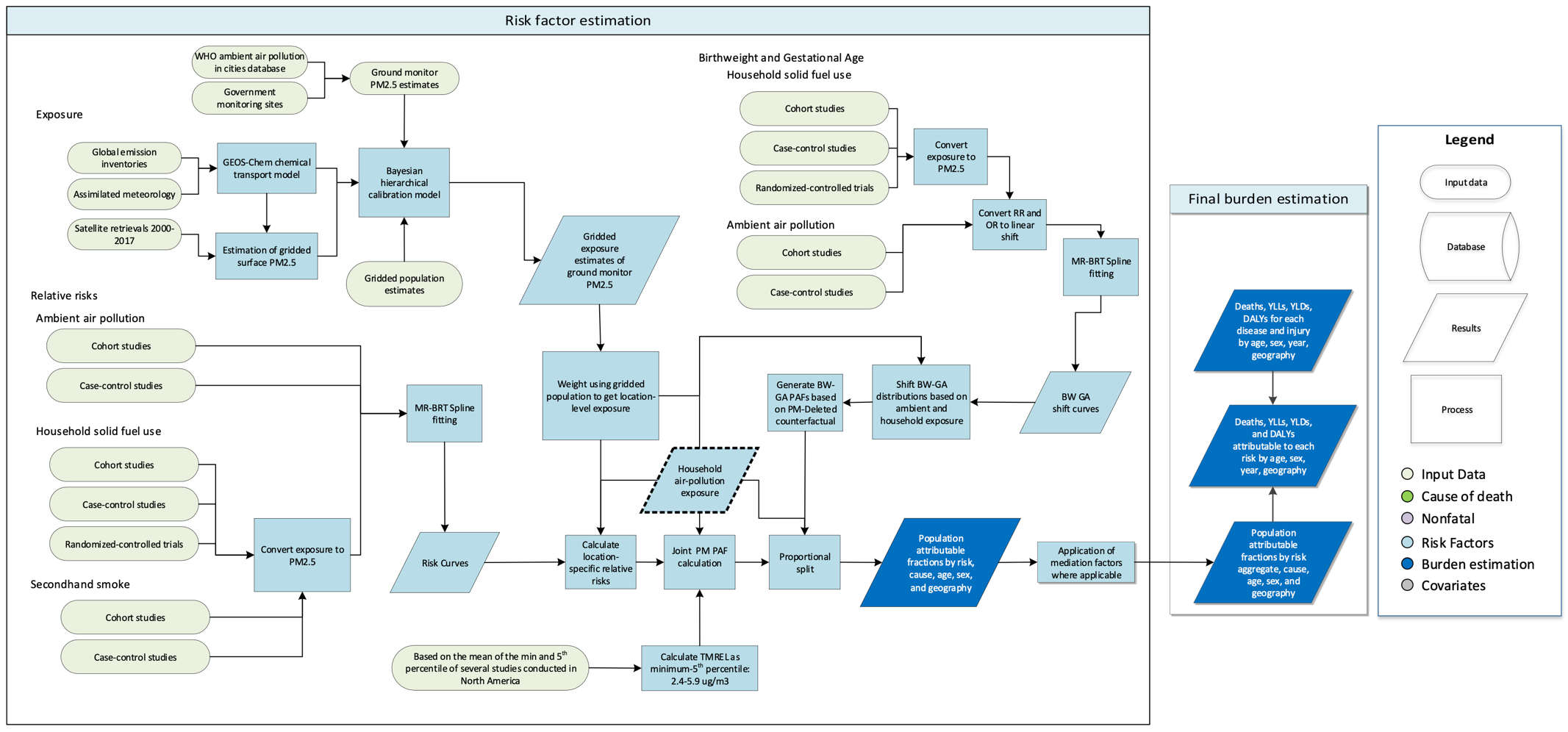
**

*References*

1. Shaddick G, Thomas ML, Green A, et al. Data Integration Model for Air Quality: A Hierarchical Approach to the Global Estimation of Exposures to Ambient Air Pollution. *Journal of the Royal Statistical Society Series C: Applied Statistics* 2018; **67**(1): 231-53.

2. Shaddick G, Thomas ML, Mudu P, Ruggeri G, Gumy S. Half the world’s population are exposed to increasing air pollution. *npj Climate and Atmospheric Science* 2020; **3**(1): 23.

3. Brauer M, Freedman G, Frostad J, et al. Ambient Air Pollution Exposure Estimation for the Global Burden of Disease 2013. *Environ Sci Technol* 2016; **50**(1): 79-88.

4. Shaddick G, Thomas ML, Amini H, et al. Data Integration for the Assessment of Population Exposure to Ambient Air Pollution for Global Burden of Disease Assessment. *Environ Sci Technol* 2018; **52**(16): 9069-78.

Appendix 3. GBD 2021 household particulate matter pollution-specific modelling descriptions

*Definition*

Exposure to household particulate matter pollution (HPMP) is estimated from both the proportion of individuals using solid cooking fuels and the level of exposure to particulate matter less than 2.5 micrometres in diameter (PM_2.5_) air pollution for these individuals. Solid fuels in our analysis include wood, coal/charcoal, dung, and agricultural residues.

*Input data*

We extracted information on the use of solid fuels for cooking from standard multi-country survey series, including the Demographic and Health Surveys (DHS), Living Standards Measurement Surveys (LSMS), Multiple Indicator Cluster Surveys (MICS), and World Health Surveys (WHS). We also used data from censuses and country-specific survey series, such as the Kenya Welfare Monitoring Survey and South Africa General Household Survey. To fill remaining gaps in survey and census data, we downloaded the WHO Household Energy Database and updated estimates using extracted information from literature through a systematic review (https://ghdx.healthdata.org/record/who-household-energy-database-1960-2017). From this combined body of input data, each nationally or subnationally representative datapoint provided an estimate of the percentage of households or individuals using solid cooking fuels. We used studies from 1980 to 2020 to inform our time series estimates.

We excluded sources that did not distinguish specific primary fuel types, estimated fuel used for purposes other than cooking (eg, lighting or heating), failed to report standard error or sample size, reported over 15% missingness for households surveyed, reported fuel use in physical units, or were secondary sources referencing primary analyses.

*Modelling strategy*

As in the Global Burden of Disease (GBD) Study 2019, household air pollution was modelled at the individual level using a three-step modelling strategy implementing linear regression, spatiotemporal regression, and Gaussian process regression (GPR). The full ST-GPR process is specified elsewhere in this appendix.

For GBD 2021, we updated the HPMP proportion model to disaggregate estimates of solid fuel use to estimate the proportion of individuals using each of the following component fuel type categories: 1) coal or charcoal, 2) crop residue, 3) dung, and 4) wood. With this strategy, we can more finely characterise individual exposure to PM_2.5_ due to solid fuel use by applying fuel-specific mapping values to fuel-specific proportion estimates. This change addresses an important limitation in our model, in that it previously assumed equal PM_2.5_ exposure for all solid fuel categories.

Fuel type-specific estimates were generated by first using ST-GPR to generate location- and year-specific estimates for coal, crop, dung, and wood. ST-GPR was also used to create estimates for the parent solid fuel category, as in GBD 2019. individuals using solid cooking fuels. For each of the linear models, maternal education and the proportion of population.The first step of the ST-GPR modelling process is a mixed-effect linear regression of logit-transformed proportion of individuals using solid cooking fuels. For each of the linear models, maternal education and the proportion of population living in urban areas were used as covariates. These models also included nested random effects by GBD region and GBD super-region.

*Flowchart*

**
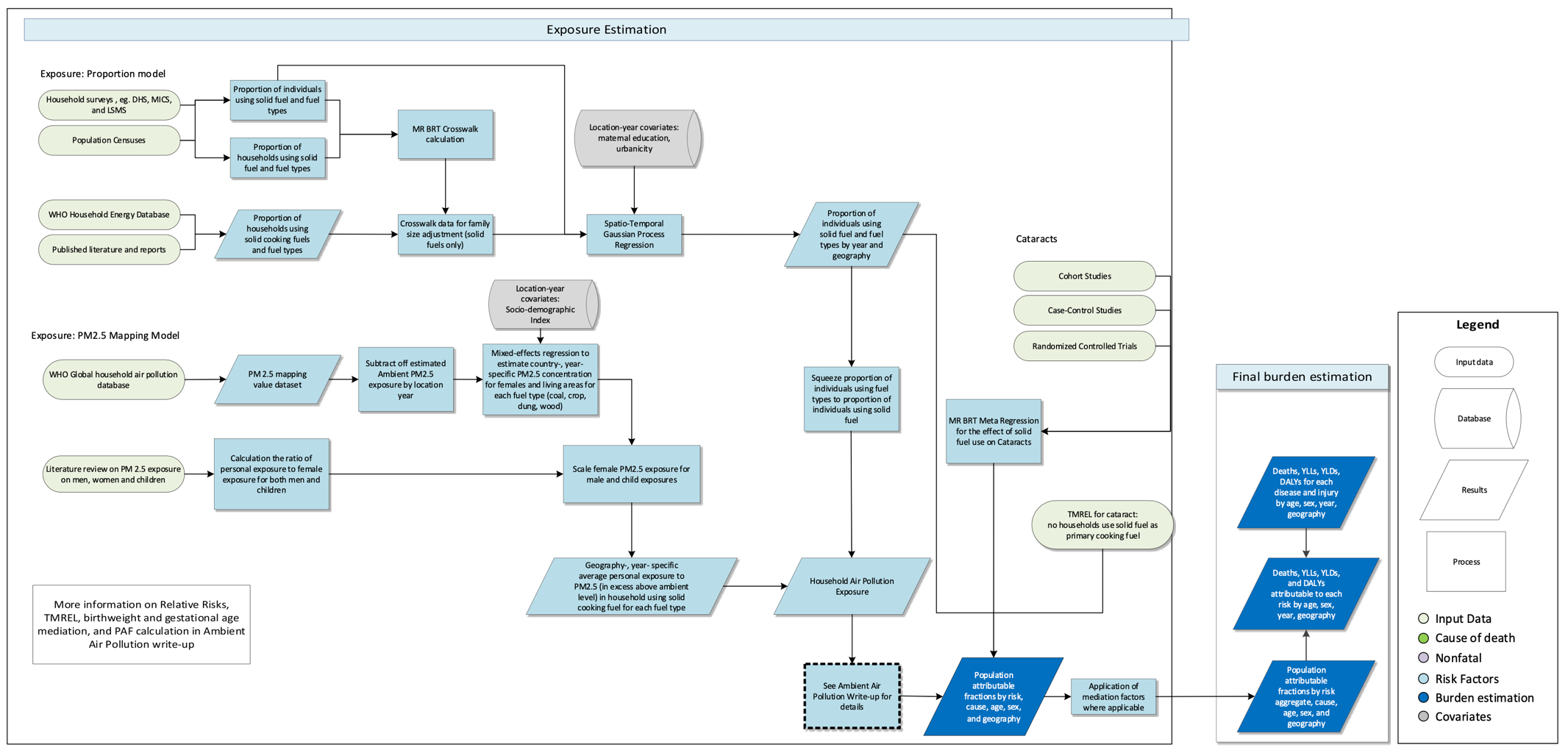
**

Table S1. Global deaths attributable to particulate matter pollution in 1990 and 2021, and estimated annual percentage changes from 1990 to 2021.

| **Cause of deaths** | **1990** |  |  | **2021** |  |  | **1990–2021** |
| --- | --- | --- | --- | --- | --- | --- | --- |
|  | **Number of cases** | **Age-standardised rates per 100 000 people** | **Age-standardised PAF (%)** | **Number of cases** | **Age-standardised rates per 100 000 people** | **Age-standardised PAF (%)** | **EAPC in age-standardised rates (%)** |
| **All causes** | 7250210.25 (6032279.35 to 8453897.77) | 182.73 (153.27 to 211.42) | 16.50 (13.88 to 18.93) | 7833220.92 (6479473.76 to 9263395.35) | 96.69 (79.91 to 114.40) | 11.57 (9.73 to 13.53) | -2.18 (-2.31 to -2.04) |
| Female | 3382534.78 (2769987.51 to 3976064.41) | 155.45 (128.50 to 181.36) | 16.32 (13.60 to 18.98) | 3478614.32 (2842903.78 to 4127413.84) | 78.35 (64.30 to 92.70) | 11.51 (9.52 to 13.43) | -2.35 (-2.47 to -2.23) |
| Male | 3867675.47 (3238507.14 to 4480010.50) | 218.25 (183.44 to 252.10) | 16.83 (14.24 to 19.15) | 4354606.60 (3629597.41 to 5195553.86) | 119.23 (99.30 to 142.09) | 11.70 (9.83 to 13.72) | -2.06 (-2.21 to -1.90) |
| **Cardiovascular diseases** | 3325404.39 (2815934.49 to 3860662.77) | 93.93 (79.12 to 109.67) | 26.23 (22.03 to 30.48) | 4482496.23 (3567564.40 to 5392617.34) | 53.62 (42.70 to 64.57) | 22.80 (18.84 to 26.90) | -1.92 (-2.06 to -1.78) |
| Ischemic heart disease | 1570387.14 (1199838.22 to 1966478.22) | 45.07 (33.95 to 56.58) | 28.37 (21.71 to 35.41) | 2492809.91 (1866677.65 to 3103230.08) | 29.88 (22.33 to 37.22) | 27.48 (21.21 to 33.85) | -1.37 (-1.47 to -1.28) |
| Stroke | 1755017.25 (1434138.60 to 2094574.28) | 48.86 (39.69 to 58.76) | 33.86 (27.93 to 40.55) | 1989686.32 (1530479.07 to 2493237.87) | 23.74 (18.26 to 29.80) | 27.15 (21.64 to 33.64) | -2.51 (-2.72 to -2.30) |
| Intracerebral hemorrhage | 932433.78 (759243.63 to 1121403.81) | 24.55 (19.97 to 29.59) | 39.83 (33.22 to 46.90) | 995650.44 (763171.63 to 1249510.29) | 11.69 (8.94 to 14.69) | 29.91 (23.60 to 36.66) | -2.54 (-2.82 to -2.26) |
| Ischemic stroke | 681182.99 (537100.48 to 836697.10) | 20.65 (16.22 to 25.54) | 28.23 (22.79 to 34.72) | 905602.35 (694785.38 to 1144793.07) | 11.01 (8.44 to 13.96) | 24.93 (19.93 to 31.10) | -2.19 (-2.37 to -2.02) |
| Subarachnoid hemorrhage | 141400.48 (90259.94 to 191691.06) | 3.65 (2.29 to 4.97) | 38.15 (31.20 to 44.85) | 88433.52 (65255.07 to 116468.07) | 1.04 (0.76 to 1.36) | 24.73 (19.35 to 30.95) | -4.64 (-4.92 to -4.35) |
| **Maternal and neonatal disorders** | 796665.63 (729077.95 to 862650.30) | 12.45 (11.39 to 13.48) | 23.84 (22.20 to 25.33) | 496966.05 (419486.39 to 580880.21) | 8.03 (6.78 to 9.39) | 25.13 (23.34 to 27.04) | -1.53 (-1.63 to -1.43) |
| Neonatal disorders | 796665.63 (729077.95 to 862650.30) | 12.45 (11.39 to 13.48) | 27.03 (25.24 to 28.68) | 496966.05 (419486.39 to 580880.21) | 8.03 (6.78 to 9.39) | 27.18 (25.28 to 29.26) | -1.53 (-1.63 to -1.43) |
| Neonatal preterm birth | 399135.87 (360229.86 to 444496.55) | 6.24 (5.63 to 6.94) | 30.77 (28.76 to 32.74) | 227615.56 (189160.11 to 271466.91) | 3.68 (3.06 to 4.39) | 30.84 (28.69 to 33.10) | -1.73 (-1.80 to -1.66) |
| Neonatal encephalopathy due to birth asphyxia and trauma | 218251.53 (190582.27 to 252965.02) | 3.41 (2.98 to 3.95) | 24.68 (22.69 to 26.56) | 155361.31 (129104.49 to 187255.72) | 2.51 (2.09 to 3.03) | 25.77 (23.38 to 28.09) | -1.15 (-1.32 to -0.98) |
| Neonatal sepsis and other neonatal infections | 70606.64 (61145.76 to 81143.52) | 1.10 (0.96 to 1.27) | 23.18 (21.44 to 24.93) | 54025.69 (45371.42 to 64084.44) | 0.87 (0.73 to 1.04) | 23.24 (21.62 to 24.81) | -0.96 (-1.09 to -0.84) |
| Other neonatal disorders | 85619.55 (60140.57 to 106991.40) | 1.34 (0.94 to 1.67) | 23.84 (21.45 to 26.02) | 52435.68 (36764.50 to 65587.59) | 0.85 (0.59 to 1.06) | 23.65 (21.45 to 25.59) | -1.77 (-1.94 to -1.61) |
| Hemolytic disease and other neonatal jaundice | 23052.04 (19371.92 to 29079.47) | 0.36 (0.30 to 0.45) | 22.32 (20.59 to 23.96) | 7527.82 (5818.67 to 9920.66) | 0.12 (0.09 to 0.16) | 22.40 (20.29 to 24.79) | -3.52 (-3.69 to -3.35) |
| **Respiratory infections and tuberculosis** | 1211283.75 (270846.59 to 1918619.59) | 23.36 (4.96 to 37.23) | 22.72 (4.96 to 35.24) | 651356.39 (121714.90 to 1076711.75) | 8.68 (1.71 to 14.39) | 6.34 (1.26 to 10.44) | -3.02 (-3.19 to -2.86) |
| Lower respiratory infections | 1211023.55 (270570.48 to 1918459.48) | 23.35 (4.95 to 37.23) | 37.73 (8.25 to 58.14) | 651238.01 (121605.08 to 1076503.21) | 8.68 (1.71 to 14.39) | 30.21 (6.15 to 48.36) | -3.02 (-3.19 to -2.86) |
| Upper respiratory infections | 247.04 (56.55 to 515.44) | 0.00 (0.00 to 0.01) | 0.45 (0.28 to 0.70) | 116.58 (18.26 to 299.46) | 0.00 (0.00 to 0.00) | 0.59 (0.28 to 0.85) | -2.44 (-2.52 to -2.37) |
| Otitis media | 13.16 (4.07 to 30.64) | 0.00 (0.00 to 0.00) | 0.65 (0.36 to 1.24) | 1.80 (0.55 to 4.62) | 0.00 (0.00 to 0.00) | 0.41 (0.24 to 0.74) | -6.28 (-6.51 to -6.05) |
| **Chronic respiratory diseases** | 1490215.77 (1238034.05 to 1698893.63) | 42.53 (35.33 to 48.43) | 50.29 (43.52 to 55.69) | 1535298.01 (1214704.19 to 1918255.85) | 18.51 (14.64 to 23.13) | 34.57 (28.32 to 42.55) | -2.96 (-3.13 to -2.79) |
| Chronic obstructive pulmonary disease | 1490215.77 (1238034.05 to 1698893.63) | 42.53 (35.33 to 48.43) | 59.12 (51.90 to 65.24) | 1535298.01 (1214704.19 to 1918255.85) | 18.51 (14.64 to 23.13) | 40.95 (33.37 to 50.00) | -2.96 (-3.13 to -2.79) |
| **Diabetes and kidney diseases** | 117055.20 (69992.05 to 165962.17) | 3.19 (1.91 to 4.55) | 9.59 (5.74 to 13.54) | 281908.84 (165678.17 to 395527.82) | 3.32 (1.95 to 4.66) | 8.69 (5.35 to 12.41) | 0.03 (-0.05 to 0.10) |
| Diabetes mellitus | 117055.20 (69992.05 to 165962.17) | 3.19 (1.91 to 4.55) | 17.58 (10.58 to 24.89) | 281908.84 (165678.17 to 395527.82) | 3.32 (1.95 to 4.66) | 16.94 (10.38 to 24.09) | 0.03 (-0.05 to 0.10) |
| Diabetes mellitus type 2 | 117055.20 (69992.05 to 165962.17) | 3.19 (1.91 to 4.55) | 18.43 (11.15 to 26.11) | 281908.84 (165678.17 to 395527.82) | 3.32 (1.95 to 4.66) | 17.47 (10.69 to 24.84) | 0.03 (-0.05 to 0.10) |
| **Neoplasms** | 253290.63 (163148.68 to 345122.52) | 6.39 (4.12 to 8.71) | 4.31 (2.79 to 5.86) | 374212.72 (236358.33 to 520255.39) | 4.34 (2.74 to 6.04) | 3.73 (2.42 to 5.17) | -1.32 (-1.49 to -1.16) |
| Tracheal, bronchus, and lung cancer | 253290.63 (163148.68 to 345122.52) | 6.39 (4.12 to 8.71) | 23.19 (15.15 to 31.65) | 374212.72 (236358.33 to 520255.39) | 4.34 (2.74 to 6.04) | 18.47 (11.87 to 25.52) | -1.32 (-1.49 to -1.16) |
| **Sense organ diseases** | NA | NA | NA | NA | NA | NA | NA |
| Blindness and vision loss | NA | NA | NA | NA | NA | NA | NA |
| Cataract | NA | NA | NA | NA | NA | NA | NA |
| **Enteric infections** | 45850.59 (32228.19 to 56815.13) | 0.72 (0.50 to 0.89) | 1.11 (0.83 to 1.42) | 6558.80 (4931.11 to 9244.79) | 0.11 (0.08 to 0.15) | 0.60 (0.41 to 0.85) | -6.20 (-6.49 to -5.92) |
| Diarrheal diseases | 45850.59 (32228.19 to 56815.13) | 0.72 (0.50 to 0.89) | 1.20 (0.88 to 1.55) | 6558.80 (4931.11 to 9244.79) | 0.11 (0.08 to 0.15) | 0.70 (0.46 to 1.02) | -6.20 (-6.49 to -5.92) |
| **Other infectious diseases** | 8405.65 (7061.81 to 10543.20) | 0.13 (0.11 to 0.17) | 0.41 (0.29 to 0.55) | 3715.75 (2867.22 to 4849.19) | 0.06 (0.05 to 0.08) | 0.74 (0.59 to 0.97) | -2.65 (-3.03 to -2.26) |
| Meningitis | 7954.38 (6668.58 to 10128.07) | 0.12 (0.10 to 0.16) | 1.68 (1.45 to 2.06) | 3434.16 (2619.15 to 4550.79) | 0.06 (0.04 to 0.07) | 1.89 (1.59 to 2.38) | -2.70 (-3.11 to -2.29) |
| Encephalitis | 451.27 (332.01 to 523.18) | 0.01 (0.01 to 0.01) | 0.44 (0.37 to 0.51) | 281.58 (211.51 to 363.29) | 0.00 (0.00 to 0.01) | 0.38 (0.31 to 0.46) | -1.85 (-2.03 to -1.68) |
| **Other non-communicable diseases** | 2038.64 (887.07 to 3503.68) | 0.03 (0.01 to 0.06) | 0.14 (0.06 to 0.24) | 708.14 (371.35 to 1049.21) | 0.01 (0.01 to 0.02) | 0.07 (0.04 to 0.10) | -3.69 (-3.96 to -3.42) |
| Sudden infant death syndrome | 2038.64 (887.07 to 3503.68) | 0.03 (0.01 to 0.06) | 2.62 (1.75 to 3.40) | 708.14 (371.35 to 1049.21) | 0.01 (0.01 to 0.02) | 2.32 (1.84 to 2.83) | -3.69 (-3.96 to -3.42) |

Table S2. Global deaths attributable to ambient particulate matter pollution in 1990 and 2021, and estimated annual percentage changes from 1990 to 2021.

| **Cause of deaths** | **1990** |  |  | **2021** |  |  | **1990–2021** |
| --- | --- | --- | --- | --- | --- | --- | --- |
|  | **Number of cases** | **Age-standardised rates per 100 000 people** | **Age-standardised PAF (%)** | **Number of cases** | **Age-standardised rates per 100 000 people** | **Age-standardised PAF (%)** | **EAPC in age-standardised rates (%)** |
| **All causes** | 2433557.86 (1750350.02 to 3155644.05) | 66.95 (48.23 to 86.33) | 6.05 (4.42 to 7.76) | 4718812.24 (3480471.83 to 5795946.44) | 57.62 (42.32 to 70.99) | 6.90 (5.13 to 8.41) | -0.34 (-0.48 to -0.20) |
| Female | 1089153.87 (785191.01 to 1410162.67) | 53.86 (38.48 to 69.66) | 5.65 (4.05 to 7.32) | 1993962.52 (1407526.55 to 2522560.50) | 43.72 (30.76 to 55.38) | 6.42 (4.59 to 7.96) | -0.56 (-0.70 to -0.42) |
| Male | 1344403.98 (964231.51 to 1746985.26) | 83.52 (60.15 to 107.21) | 6.44 (4.70 to 8.22) | 2724849.72 (2060428.49 to 3333084.38) | 75.02 (56.85 to 91.52) | 7.36 (5.59 to 8.86) | -0.17 (-0.32 to -0.02) |
| **Cardiovascular diseases** | 1444385.97 (1016790.55 to 1861497.27) | 42.98 (30.21 to 55.62) | 12.00 (8.51 to 15.44) | 2960398.58 (2111795.16 to 3701437.50) | 35.64 (25.39 to 44.52) | 15.15 (10.82 to 18.75) | -0.48 (-0.61 to -0.34) |
| Ischemic heart disease | 849815.79 (578301.40 to 1131092.04) | 25.55 (17.27 to 34.24) | 16.08 (10.90 to 21.59) | 1729546.17 (1212500.98 to 2286981.38) | 20.85 (14.63 to 27.57) | 19.18 (13.53 to 24.96) | -0.55 (-0.73 to -0.37) |
| Stroke | 594570.18 (410655.54 to 816223.87) | 17.42 (12.14 to 23.95) | 12.07 (8.63 to 16.34) | 1230852.41 (834766.63 to 1575049.10) | 14.78 (10.05 to 18.92) | 16.90 (11.81 to 21.22) | -0.37 (-0.51 to -0.23) |
| Intracerebral hemorrhage | 243475.20 (157445.83 to 352272.68) | 6.50 (4.19 to 9.42) | 10.54 (7.02 to 15.09) | 574180.15 (377388.13 to 744661.19) | 6.79 (4.47 to 8.81) | 17.36 (11.68 to 21.94) | 0.40 (0.16 to 0.63) |
| Ischemic stroke | 312965.14 (220993.96 to 425504.35) | 9.94 (7.02 to 13.40) | 13.58 (9.91 to 18.17) | 603110.61 (418419.59 to 758667.60) | 7.37 (5.12 to 9.26) | 16.67 (12.04 to 20.85) | -0.85 (-0.98 to -0.72) |
| Subarachnoid hemorrhage | 38129.84 (23179.25 to 57695.62) | 0.99 (0.60 to 1.50) | 10.39 (6.81 to 15.40) | 53561.65 (36516.80 to 69717.63) | 0.63 (0.43 to 0.82) | 15.09 (10.35 to 19.09) | -1.73 (-1.99 to -1.47) |
| **Chronic respiratory diseases** | 363675.77 (240011.63 to 531999.39) | 10.53 (6.98 to 15.32) | 12.46 (8.50 to 17.94) | 841465.56 (610674.96 to 1036867.11) | 10.23 (7.44 to 12.62) | 19.09 (13.98 to 22.91) | -0.07 (-0.26 to 0.12) |
| Chronic obstructive pulmonary disease | 363675.77 (240011.63 to 531999.39) | 10.53 (6.98 to 15.32) | 14.64 (9.87 to 20.81) | 841465.56 (610674.96 to 1036867.11) | 10.23 (7.44 to 12.62) | 22.61 (16.49 to 27.43) | -0.07 (-0.26 to 0.12) |
| **Maternal and neonatal disorders** | 152759.00 (101572.74 to 211126.90) | 2.39 (1.59 to 3.30) | 4.57 (3.02 to 6.24) | 140758.31 (83378.21 to 213926.49) | 2.27 (1.35 to 3.46) | 7.12 (4.33 to 10.27) | 0.19 (-0.03 to 0.41) |
| Neonatal disorders | 152759.00 (101572.74 to 211126.90) | 2.39 (1.59 to 3.30) | 5.18 (3.44 to 7.05) | 140758.31 (83378.21 to 213926.49) | 2.27 (1.35 to 3.46) | 7.70 (4.69 to 11.09) | 0.19 (-0.03 to 0.41) |
| Neonatal preterm birth | 87020.24 (57701.71 to 116942.15) | 1.36 (0.90 to 1.83) | 6.71 (4.48 to 8.89) | 72729.87 (44270.94 to 109919.10) | 1.18 (0.72 to 1.78) | 9.85 (6.07 to 13.86) | -0.04 (-0.28 to 0.19) |
| Neonatal encephalopathy due to birth asphyxia and trauma | 36326.92 (23171.11 to 51222.21) | 0.57 (0.36 to 0.80) | 4.11 (2.63 to 5.78) | 39153.30 (22021.01 to 60103.89) | 0.63 (0.36 to 0.97) | 6.49 (3.76 to 9.82) | 0.68 (0.44 to 0.91) |
| Other neonatal disorders | 14026.50 (8859.04 to 20526.86) | 0.22 (0.14 to 0.32) | 3.92 (2.61 to 5.61) | 13513.36 (7775.38 to 20413.07) | 0.22 (0.13 to 0.33) | 6.11 (3.75 to 9.02) | 0.09 (-0.12 to 0.29) |
| Neonatal sepsis and other neonatal infections | 11569.70 (7498.35 to 16496.11) | 0.18 (0.12 to 0.26) | 3.80 (2.57 to 5.32) | 13079.66 (7634.92 to 20047.64) | 0.21 (0.12 to 0.32) | 5.63 (3.35 to 8.16) | 0.69 (0.47 to 0.91) |
| Hemolytic disease and other neonatal jaundice | 3815.63 (2400.47 to 5751.26) | 0.06 (0.04 to 0.09) | 3.70 (2.42 to 5.39) | 2282.11 (1322.50 to 3474.55) | 0.04 (0.02 to 0.06) | 6.82 (3.97 to 10.06) | -1.21 (-1.51 to -0.91) |
| **Respiratory infections and tuberculosis** | 279021.14 (54132.85 to 471110.34) | 6.04 (1.06 to 10.35) | 5.88 (1.06 to 10.11) | 290581.22 (44192.28 to 512773.66) | 3.79 (0.60 to 6.69) | 2.76 (0.45 to 4.84) | -1.07 (-1.30 to -0.85) |
| Lower respiratory infections | 278969.47 (54085.11 to 471066.82) | 6.04 (1.06 to 10.35) | 9.76 (1.74 to 16.89) | 290558.44 (44165.10 to 512744.41) | 3.79 (0.60 to 6.69) | 13.18 (2.16 to 22.86) | -1.07 (-1.30 to -0.85) |
| Upper respiratory infections | 49.46 (20.85 to 100.92) | 0.00 (0.00 to 0.00) | 0.10 (0.06 to 0.17) | 22.58 (6.81 to 57.68) | 0.00 (0.00 to 0.00) | 0.14 (0.08 to 0.22) | -1.98 (-2.30 to -1.65) |
| Otitis media | 2.21 (0.82 to 4.83) | 0.00 (0.00 to 0.00) | 0.12 (0.06 to 0.21) | 0.20 (0.08 to 0.45) | 0.00 (0.00 to 0.00) | 0.05 (0.03 to 0.08) | -6.81 (-7.80 to -5.81) |
| **Diabetes and kidney diseases** | 57093.66 (32912.65 to 82748.59) | 1.62 (0.92 to 2.34) | 4.85 (2.82 to 7.08) | 185418.62 (107502.21 to 271870.55) | 2.19 (1.27 to 3.21) | 5.74 (3.32 to 8.43) | 1.00 (0.90 to 1.10) |
| Diabetes mellitus | 57093.66 (32912.65 to 82748.59) | 1.62 (0.92 to 2.34) | 8.89 (5.16 to 12.96) | 185418.62 (107502.21 to 271870.55) | 2.19 (1.27 to 3.21) | 11.18 (6.46 to 16.42) | 1.00 (0.90 to 1.10) |
| Diabetes mellitus type 2 | 57093.66 (32912.65 to 82748.59) | 1.62 (0.92 to 2.34) | 9.32 (5.42 to 13.58) | 185418.62 (107502.21 to 271870.55) | 2.19 (1.27 to 3.21) | 11.53 (6.67 to 16.97) | 1.00 (0.90 to 1.10) |
| **Neoplasms** | 127406.80 (74893.48 to 187301.08) | 3.25 (1.91 to 4.80) | 2.19 (1.28 to 3.24) | 297597.93 (183710.52 to 414740.30) | 3.46 (2.14 to 4.82) | 2.97 (1.88 to 4.22) | 0.45 (0.32 to 0.59) |
| Tracheal, bronchus, and lung cancer | 127406.80 (74893.48 to 187301.08) | 3.25 (1.91 to 4.80) | 11.80 (6.94 to 17.38) | 297597.93 (183710.52 to 414740.30) | 3.46 (2.14 to 4.82) | 14.71 (9.24 to 20.73) | 0.45 (0.32 to 0.59) |
| **Enteric infections** | 7460.79 (4550.67 to 11353.95) | 0.12 (0.07 to 0.18) | 0.18 (0.11 to 0.28) | 1557.50 (897.27 to 2513.99) | 0.03 (0.01 to 0.04) | 0.14 (0.08 to 0.23) | -4.56 (-4.78 to -4.34) |
| Diarrheal diseases | 7460.79 (4550.67 to 11353.95) | 0.12 (0.07 to 0.18) | 0.19 (0.12 to 0.30) | 1557.50 (897.27 to 2513.99) | 0.03 (0.01 to 0.04) | 0.17 (0.09 to 0.28) | -4.56 (-4.78 to -4.34) |
| **Other infectious diseases** | 1368.33 (893.66 to 1965.67) | 0.02 (0.01 to 0.03) | 0.07 (0.04 to 0.10) | 819.79 (482.83 to 1329.34) | 0.01 (0.01 to 0.02) | 0.16 (0.09 to 0.26) | -1.05 (-1.36 to -0.73) |
| Meningitis | 1283.29 (836.50 to 1860.52) | 0.02 (0.01 to 0.03) | 0.27 (0.18 to 0.39) | 738.01 (428.53 to 1213.64) | 0.01 (0.01 to 0.02) | 0.41 (0.23 to 0.65) | -1.12 (-1.46 to -0.79) |
| Encephalitis | 85.04 (54.54 to 118.56) | 0.00 (0.00 to 0.00) | 0.08 (0.05 to 0.12) | 81.78 (49.79 to 120.86) | 0.00 (0.00 to 0.00) | 0.11 (0.07 to 0.16) | -0.13 (-0.34 to 0.08) |
| **Other non-communicable diseases** | 386.39 (196.41 to 663.66) | 0.01 (0.00 to 0.01) | 0.03 (0.01 to 0.04) | 214.73 (108.12 to 356.98) | 0.00 (0.00 to 0.01) | 0.02 (0.01 to 0.03) | -1.67 (-1.84 to -1.51) |
| Sudden infant death syndrome | 386.39 (196.41 to 663.66) | 0.01 (0.00 to 0.01) | 0.50 (0.34 to 0.71) | 214.73 (108.12 to 356.98) | 0.00 (0.00 to 0.01) | 0.71 (0.46 to 1.00) | -1.67 (-1.84 to -1.51) |

Table S3. Global deaths attributable to household particulate matter pollution in 1990 and 2021, and estimated annual percentage changes from 1990 to 2021.

| **Cause of deaths** | **1990** |  |  | **2021** |  |  | **1990–2021** |
| --- | --- | --- | --- | --- | --- | --- | --- |
|  | **Number of cases** | **Age-standardised rates per 100 000 people** | **Age-standardised PAF (%)** | **Number of cases** | **Age-standardised rates per 100 000 people** | **Age-standardised PAF (%)** | **EAPC in age-standardised rates (%)** |
| **All causes** | 4815870.19 (3773986.88 to 5859598.13) | 115.77 (90.88 to 140.88) | 10.46 (8.26 to 12.62) | 3112926.41 (1895168.20 to 5188696.63) | 39.05 (23.97 to 64.45) | 4.67 (2.86 to 7.63) | -3.81 (-4.13 to -3.49) |
| Female | 2293002.94 (1774558.48 to 2802608.64) | 101.57 (80.46 to 124.23) | 10.66 (8.48 to 13.01) | 1484003.79 (897203.11 to 2473697.25) | 34.62 (21.13 to 56.77) | 5.08 (3.16 to 8.20) | -3.75 (-4.04 to -3.46) |
| Male | 2522867.25 (1959507.33 to 3106686.57) | 134.71 (105.92 to 165.36) | 10.39 (8.12 to 12.56) | 1628922.62 (992877.48 to 2812294.63) | 44.19 (27.01 to 76.62) | 4.34 (2.61 to 7.30) | -3.92 (-4.28 to -3.56) |
| **Maternal and neonatal disorders** | 643799.44 (569756.78 to 716523.36) | 10.06 (8.90 to 11.19) | 19.27 (17.15 to 21.17) | 356105.30 (281809.12 to 441046.61) | 5.76 (4.55 to 7.13) | 18.01 (14.87 to 21.23) | -2.07 (-2.24 to -1.89) |
| Neonatal disorders | 643799.44 (569756.78 to 716523.36) | 10.06 (8.90 to 11.19) | 21.84 (19.46 to 23.98) | 356105.30 (281809.12 to 441046.61) | 5.76 (4.55 to 7.13) | 19.48 (16.11 to 22.96) | -2.07 (-2.24 to -1.89) |
| Neonatal preterm birth | 312053.79 (271256.90 to 357723.63) | 4.88 (4.24 to 5.59) | 24.06 (21.33 to 26.62) | 154834.32 (118669.94 to 196763.28) | 2.50 (1.92 to 3.18) | 20.98 (16.91 to 24.86) | -2.36 (-2.49 to -2.22) |
| Neonatal encephalopathy due to birth asphyxia and trauma | 181899.46 (155672.88 to 213149.44) | 2.84 (2.43 to 3.33) | 20.56 (18.29 to 22.78) | 116175.84 (90402.84 to 145651.59) | 1.88 (1.46 to 2.35) | 19.27 (15.72 to 22.74) | -1.63 (-1.87 to -1.38) |
| Neonatal sepsis and other neonatal infections | 59029.19 (50636.79 to 68921.59) | 0.92 (0.79 to 1.08) | 19.38 (17.28 to 21.38) | 40936.59 (32376.82 to 51168.44) | 0.66 (0.52 to 0.83) | 17.61 (15.00 to 20.28) | -1.39 (-1.57 to -1.20) |
| Other neonatal disorders | 71583.05 (48796.39 to 91502.04) | 1.12 (0.76 to 1.43) | 19.92 (17.31 to 22.34) | 38914.50 (26005.88 to 49960.03) | 0.63 (0.42 to 0.81) | 17.53 (14.42 to 20.53) | -2.26 (-2.49 to -2.03) |
| Hemolytic disease and other neonatal jaundice | 19233.96 (15699.04 to 24622.78) | 0.30 (0.25 to 0.39) | 18.62 (16.26 to 20.58) | 5244.05 (3724.24 to 7550.46) | 0.08 (0.06 to 0.12) | 15.58 (12.31 to 18.88) | -4.20 (-4.45 to -3.95) |
| **Cardiovascular diseases** | 1880657.88 (1472837.26 to 2357817.40) | 50.94 (39.86 to 64.22) | 14.23 (11.15 to 18.02) | 1521318.10 (850724.66 to 2685934.99) | 17.97 (10.01 to 31.95) | 7.64 (4.24 to 13.69) | -3.69 (-4.10 to -3.28) |
| Ischemic heart disease | 720347.31 (526576.88 to 963466.42) | 19.51 (14.16 to 26.53) | 12.28 (9.05 to 16.60) | 762836.06 (412878.66 to 1370924.53) | 9.02 (4.85 to 16.30) | 8.30 (4.55 to 15.19) | -2.71 (-3.10 to -2.32) |
| Stroke | 1160310.57 (903175.22 to 1454288.17) | 31.43 (24.47 to 39.50) | 21.78 (16.94 to 27.16) | 758482.04 (432749.89 to 1311607.95) | 8.95 (5.08 to 15.57) | 10.24 (5.72 to 18.39) | -4.46 (-4.90 to -4.02) |
| Intracerebral hemorrhage | 688906.17 (538085.23 to 842487.03) | 18.05 (14.14 to 22.09) | 29.29 (23.19 to 35.32) | 421296.25 (237601.82 to 718017.12) | 4.90 (2.75 to 8.38) | 12.54 (7.00 to 21.85) | -4.57 (-5.05 to -4.10) |
| Ischemic stroke | 368144.16 (273803.90 to 486640.08) | 10.71 (7.96 to 14.26) | 14.65 (10.95 to 19.48) | 302332.18 (163067.76 to 560673.51) | 3.64 (1.96 to 6.78) | 8.25 (4.53 to 15.73) | -3.91 (-4.37 to -3.44) |
| Subarachnoid hemorrhage | 103260.24 (61483.19 to 142558.85) | 2.66 (1.57 to 3.68) | 27.76 (21.18 to 34.35) | 34853.60 (16853.45 to 61351.19) | 0.41 (0.20 to 0.72) | 9.63 (4.91 to 17.16) | -6.83 (-7.12 to -6.55) |
| **Respiratory infections and tuberculosis** | 932088.34 (213780.77 to 1486053.43) | 17.31 (3.80 to 27.44) | 16.84 (3.93 to 26.37) | 360603.05 (81420.54 to 648315.65) | 4.89 (1.16 to 8.79) | 3.57 (0.84 to 6.45) | -4.02 (-4.26 to -3.78) |
| Lower respiratory infections | 931879.84 (213533.91 to 1485795.86) | 17.31 (3.79 to 27.43) | 27.96 (6.53 to 43.49) | 360507.47 (81329.14 to 648205.82) | 4.89 (1.16 to 8.79) | 17.02 (4.11 to 29.78) | -4.02 (-4.26 to -3.78) |
| Upper respiratory infections | 197.55 (29.80 to 427.45) | 0.00 (0.00 to 0.01) | 0.35 (0.18 to 0.57) | 93.98 (9.91 to 245.34) | 0.00 (0.00 to 0.00) | 0.45 (0.16 to 0.72) | -2.56 (-2.65 to -2.46) |
| Otitis media | 10.95 (3.01 to 27.25) | 0.00 (0.00 to 0.00) | 0.53 (0.26 to 1.08) | 1.60 (0.47 to 4.18) | 0.00 (0.00 to 0.00) | 0.36 (0.21 to 0.66) | -6.19 (-6.35 to -6.04) |
| **Chronic respiratory diseases** | 1126466.64 (893942.43 to 1352064.45) | 32.00 (25.34 to 38.36) | 37.83 (31.13 to 44.26) | 693603.75 (412268.39 to 1180015.39) | 8.29 (4.90 to 14.16) | 15.48 (9.22 to 26.82) | -4.77 (-5.08 to -4.47) |
| Chronic obstructive pulmonary disease | 1126466.64 (893942.43 to 1352064.45) | 32.00 (25.34 to 38.36) | 44.47 (36.97 to 51.72) | 693603.75 (412268.39 to 1180015.39) | 8.29 (4.90 to 14.16) | 18.33 (10.93 to 31.53) | -4.77 (-5.08 to -4.47) |
| **Diabetes and kidney diseases** | 59941.29 (34969.66 to 88590.09) | 1.58 (0.92 to 2.34) | 4.74 (2.78 to 7.04) | 96425.20 (46870.53 to 169273.57) | 1.13 (0.55 to 1.99) | 2.95 (1.46 to 5.11) | -1.30 (-1.54 to -1.06) |
| Diabetes mellitus | 59941.29 (34969.66 to 88590.09) | 1.58 (0.92 to 2.34) | 8.69 (5.03 to 12.85) | 96425.20 (46870.53 to 169273.57) | 1.13 (0.55 to 1.99) | 5.76 (2.81 to 9.89) | -1.30 (-1.54 to -1.06) |
| Diabetes mellitus type 2 | 59941.29 (34969.66 to 88590.09) | 1.58 (0.92 to 2.34) | 9.11 (5.27 to 13.46) | 96425.20 (46870.53 to 169273.57) | 1.13 (0.55 to 1.99) | 5.94 (2.90 to 10.19) | -1.30 (-1.54 to -1.06) |
| **Sense organ diseases** | NA | NA | NA | NA | NA | NA | NA |
| Blindness and vision loss | NA | NA | NA | NA | NA | NA | NA |
| Cataract | NA | NA | NA | NA | NA | NA | NA |
| **Neoplasms** | 125843.80 (77842.62 to 177113.86) | 3.14 (1.94 to 4.43) | 2.12 (1.34 to 2.97) | 76482.48 (28603.77 to 187340.16) | 0.88 (0.33 to 2.16) | 0.76 (0.27 to 1.88) | -4.60 (-5.18 to -4.01) |
| Tracheal, bronchus, and lung cancer | 125843.80 (77842.62 to 177113.86) | 3.14 (1.94 to 4.43) | 11.38 (7.23 to 16.16) | 76482.48 (28603.77 to 187340.16) | 0.88 (0.33 to 2.16) | 3.75 (1.36 to 8.98) | -4.60 (-5.18 to -4.01) |
| **Enteric infections** | 38384.53 (26045.91 to 49245.26) | 0.60 (0.41 to 0.77) | 0.93 (0.69 to 1.21) | 5000.03 (3557.16 to 7137.28) | 0.08 (0.06 to 0.12) | 0.46 (0.31 to 0.68) | -6.62 (-6.96 to -6.27) |
| Diarrheal diseases | 38384.53 (26045.91 to 49245.26) | 0.60 (0.41 to 0.77) | 1.00 (0.73 to 1.31) | 5000.03 (3557.16 to 7137.28) | 0.08 (0.06 to 0.12) | 0.53 (0.35 to 0.81) | -6.62 (-6.96 to -6.27) |
| **Other infectious diseases** | 7036.35 (5744.56 to 8966.81) | 0.11 (0.09 to 0.14) | 0.34 (0.24 to 0.47) | 2895.27 (2211.46 to 3956.40) | 0.05 (0.04 to 0.06) | 0.58 (0.45 to 0.76) | -3.03 (-3.47 to -2.58) |
| Meningitis | 6670.19 (5463.83 to 8607.16) | 0.10 (0.09 to 0.13) | 1.41 (1.20 to 1.75) | 2695.51 (2047.01 to 3739.81) | 0.04 (0.03 to 0.06) | 1.48 (1.20 to 1.89) | -3.07 (-3.53 to -2.60) |
| Encephalitis | 366.16 (267.71 to 432.91) | 0.01 (0.00 to 0.01) | 0.35 (0.29 to 0.42) | 199.75 (143.22 to 270.30) | 0.00 (0.00 to 0.00) | 0.27 (0.20 to 0.34) | -2.40 (-2.62 to -2.18) |
| **Other non-communicable diseases** | 1651.93 (661.14 to 2914.89) | 0.03 (0.01 to 0.05) | 0.11 (0.04 to 0.20) | 493.24 (244.84 to 753.32) | 0.01 (0.00 to 0.01) | 0.05 (0.02 to 0.07) | -4.33 (-4.70 to -3.96) |
| Sudden infant death syndrome | 1651.93 (661.14 to 2914.89) | 0.03 (0.01 to 0.05) | 2.12 (1.31 to 2.84) | 493.24 (244.84 to 753.32) | 0.01 (0.00 to 0.01) | 1.62 (1.19 to 2.11) | -4.33 (-4.70 to -3.96) |

Table S4. Global DALYs attributable to particulate matter pollution in 1990 and 2021, and estimated annual percentage changes from 1990 to 2021.

| **Cause of DALYs** | **1990** |  |  | **2021** |  |  | **1990–2021** |
| --- | --- | --- | --- | --- | --- | --- | --- |
|  | **Number of cases** | **Age-standardised rates per 100 000 people** | **Age-standardised PAF (%)** | **Number of cases** | **Age-standardised rates per 100 000 people** | **Age-standardised PAF (%)** | **EAPC in age-standardised rates (%)** |
| **All causes** | 289354268.77 (222295153.05 to 350589635.43) | 5865.15 (4690.12 to 6998.30) | 11.56 (9.14 to 13.63) | 231511232.94 (194538892.20 to 270855451.37) | 2984.47 (2489.63 to 3487.35) | 8.25 (6.85 to 9.69) | -2.27 (-2.39 to -2.15) |
| Female | 130510933.65 (96977292.61 to 160665647.67) | 5130.84 (3925.85 to 6220.82) | 10.99 (8.31 to 13.30) | 99047039.32 (82679826.81 to 117756358.69) | 2480.23 (2059.40 to 2951.19) | 7.60 (6.20 to 9.00) | -2.43 (-2.54 to -2.31) |
| Male | 158843335.12 (124606241.94 to 190518631.45) | 6706.07 (5501.04 to 7859.56) | 12.15 (9.89 to 14.06) | 132464193.62 (110995515.82 to 154576904.15) | 3535.80 (2953.30 to 4130.72) | 8.83 (7.39 to 10.23) | -2.16 (-2.28 to -2.03) |
| **Cardiovascular diseases** | 78872929.19 (67260860.28 to 90923665.04) | 2009.92 (1706.72 to 2320.09) | 26.62 (22.64 to 30.67) | 99637837.09 (80843498.77 to 118292884.66) | 1161.77 (939.61 to 1380.37) | 22.98 (19.09 to 26.85) | -1.90 (-2.03 to -1.76) |
| Ischemic heart disease | 36568811.69 (28680229.70 to 45510296.29) | 936.39 (729.98 to 1165.82) | 30.13 (23.40 to 37.21) | 54675670.12 (41652489.01 to 67418885.61) | 638.48 (486.47 to 787.82) | 28.86 (22.37 to 35.37) | -1.31 (-1.41 to -1.22) |
| Stroke | 42304117.50 (34553909.64 to 49981909.54) | 1073.52 (877.41 to 1276.32) | 34.87 (28.82 to 41.31) | 44962166.97 (35020338.76 to 55467023.53) | 523.30 (407.96 to 645.58) | 27.75 (22.32 to 34.22) | -2.49 (-2.68 to -2.30) |
| Intracerebral hemorrhage | 24071140.41 (19643860.70 to 28848702.08) | 590.43 (481.56 to 709.05) | 38.92 (32.43 to 45.72) | 24015341.78 (18414608.05 to 29838879.10) | 276.93 (212.21 to 344.36) | 29.98 (23.83 to 36.70) | -2.58 (-2.83 to -2.33) |
| Ischemic stroke | 14141351.82 (11284670.57 to 17056670.42) | 385.24 (306.72 to 467.72) | 29.94 (24.21 to 36.43) | 18295352.08 (14324970.64 to 22541397.17) | 215.64 (168.84 to 266.03) | 25.76 (20.60 to 32.03) | -2.04 (-2.20 to -1.87) |
| Subarachnoid hemorrhage | 4091625.27 (2770549.98 to 5366896.54) | 97.85 (65.80 to 128.72) | 35.36 (28.90 to 41.67) | 2651473.11 (1994097.80 to 3505548.54) | 30.73 (23.13 to 40.64) | 24.50 (19.35 to 30.48) | -4.20 (-4.43 to -3.97) |
| **Maternal and neonatal disorders** | 71710489.07 (65623349.01 to 77654692.10) | 1120.38 (1025.34 to 1213.25) | 23.69 (22.08 to 25.18) | 44737310.78 (37766689.50 to 52293054.19) | 723.06 (610.39 to 845.18) | 23.35 (21.54 to 25.21) | -1.53 (-1.63 to -1.43) |
| Neonatal disorders | 71710489.07 (65623349.01 to 77654692.10) | 1120.38 (1025.34 to 1213.25) | 25.80 (24.05 to 27.45) | 44737310.78 (37766689.50 to 52293054.19) | 723.06 (610.39 to 845.18) | 24.58 (22.65 to 26.55) | -1.53 (-1.63 to -1.43) |
| Neonatal preterm birth | 35933559.30 (32432942.71 to 40014518.67) | 561.40 (506.76 to 625.10) | 28.62 (26.66 to 30.65) | 20494977.58 (17038342.06 to 24435506.76) | 331.25 (275.38 to 394.95) | 26.41 (24.31 to 28.80) | -1.73 (-1.80 to -1.66) |
| Neonatal encephalopathy due to birth asphyxia and trauma | 19639042.51 (17149515.35 to 22762941.10) | 306.68 (267.81 to 355.51) | 24.13 (22.04 to 25.94) | 13980138.89 (11617949.83 to 16850172.87) | 225.97 (187.79 to 272.36) | 24.24 (21.91 to 26.44) | -1.15 (-1.32 to -0.98) |
| Neonatal sepsis and other neonatal infections | 6354500.40 (5502797.14 to 7302612.37) | 99.40 (86.07 to 114.19) | 22.10 (20.31 to 23.90) | 4861948.25 (4083310.36 to 5767175.69) | 78.57 (65.98 to 93.19) | 21.20 (19.59 to 22.79) | -0.96 (-1.09 to -0.84) |
| Other neonatal disorders | 7706367.40 (5413793.11 to 9629689.26) | 120.41 (84.61 to 150.43) | 23.66 (21.29 to 25.83) | 4720040.50 (3309887.35 to 5902580.05) | 76.29 (53.49 to 95.40) | 23.34 (21.15 to 25.30) | -1.77 (-1.93 to -1.61) |
| Hemolytic disease and other neonatal jaundice | 2077019.45 (1745464.57 to 2618974.35) | 32.49 (27.30 to 40.96) | 21.30 (19.61 to 22.81) | 680205.56 (526741.16 to 895348.64) | 10.99 (8.51 to 14.47) | 18.82 (16.78 to 20.90) | -3.51 (-3.68 to -3.34) |
| **Respiratory infections and tuberculosis** | 87340785.25 (20807396.36 to 139706071.98) | 1462.60 (341.01 to 2338.69) | 27.76 (6.79 to 42.36) | 29110366.69 (7007734.71 to 48141777.96) | 420.28 (106.66 to 693.37) | 9.63 (2.44 to 15.57) | -3.76 (-3.96 to -3.55) |
| Lower respiratory infections | 87315640.36 (20780746.26 to 139682971.21) | 1462.20 (340.59 to 2338.31) | 42.07 (10.39 to 64.24) | 29098330.84 (6988264.72 to 48127683.27) | 420.09 (106.35 to 693.12) | 35.84 (9.29 to 56.41) | -3.76 (-3.96 to -3.55) |
| Upper respiratory infections | 23781.75 (6477.74 to 48147.78) | 0.37 (0.10 to 0.75) | 0.36 (0.14 to 0.64) | 11708.46 (2800.50 to 28081.79) | 0.19 (0.05 to 0.45) | 0.23 (0.07 to 0.49) | -2.32 (-2.39 to -2.25) |
| Otitis media | 1363.14 (512.18 to 2946.84) | 0.02 (0.01 to 0.05) | 0.06 (0.02 to 0.14) | 327.40 (156.27 to 626.36) | 0.01 (0.00 to 0.01) | 0.02 (0.01 to 0.03) | -4.54 (-4.80 to -4.28) |
| **Chronic respiratory diseases** | 33509790.14 (28117101.83 to 38092060.04) | 877.48 (736.13 to 995.54) | 42.29 (36.62 to 46.91) | 33238712.44 (26680066.05 to 41336741.04) | 389.50 (312.59 to 484.62) | 30.10 (24.54 to 36.88) | -2.87 (-3.01 to -2.74) |
| Chronic obstructive pulmonary disease | 33509790.14 (28117101.83 to 38092060.04) | 877.48 (736.13 to 995.54) | 58.77 (51.63 to 64.81) | 33238712.44 (26680066.05 to 41336741.04) | 389.50 (312.59 to 484.62) | 41.41 (34.01 to 50.17) | -2.87 (-3.01 to -2.74) |
| **Diabetes and kidney diseases** | 4568390.09 (2676365.07 to 6604453.55) | 112.67 (66.24 to 162.08) | 9.75 (5.85 to 13.65) | 12904493.55 (7501414.23 to 19485253.91) | 148.92 (86.50 to 224.91) | 10.25 (6.18 to 14.71) | 0.77 (0.71 to 0.83) |
| Diabetes mellitus | 4568390.09 (2676365.07 to 6604453.55) | 112.67 (66.24 to 162.08) | 16.98 (10.18 to 24.04) | 12904493.55 (7501414.23 to 19485253.91) | 148.92 (86.50 to 224.91) | 16.24 (9.94 to 23.12) | 0.77 (0.71 to 0.83) |
| Diabetes mellitus type 2 | 4568390.09 (2676365.07 to 6604453.55) | 112.67 (66.24 to 162.08) | 18.39 (11.08 to 26.08) | 12904493.55 (7501414.23 to 19485253.91) | 148.92 (86.50 to 224.91) | 17.07 (10.48 to 24.30) | 0.77 (0.71 to 0.83) |
| **Neoplasms** | 6863711.43 (4419306.57 to 9265547.03) | 165.53 (106.63 to 223.80) | 4.17 (2.71 to 5.61) | 8934120.30 (5681090.30 to 12409780.48) | 102.08 (64.89 to 141.62) | 3.45 (2.24 to 4.78) | -1.64 (-1.80 to -1.48) |
| Tracheal, bronchus, and lung cancer | 6863711.43 (4419306.57 to 9265547.03) | 165.53 (106.63 to 223.80) | 23.96 (15.73 to 32.55) | 8934120.30 (5681090.30 to 12409780.48) | 102.08 (64.89 to 141.62) | 19.15 (12.35 to 26.33) | -1.64 (-1.80 to -1.48) |
| **Sense organ diseases** | 1414029.27 (-564248.53 to 2610528.48) | 37.09 (-14.86 to 68.78) | 4.26 (-1.31 to 7.74) | 1956323.97 (-612092.84 to 4033705.23) | 22.84 (-7.15 to 47.05) | 2.54 (-0.60 to 5.17) | -1.51 (-1.72 to -1.29) |
| Blindness and vision loss | 1414029.27 (-564248.53 to 2610528.48) | 37.09 (-14.86 to 68.78) | 11.03 (-3.55 to 20.34) | 1956323.97 (-612092.84 to 4033705.23) | 22.84 (-7.15 to 47.05) | 6.83 (-1.66 to 14.11) | -1.51 (-1.72 to -1.29) |
| Cataract | 1414029.27 (-564248.53 to 2610528.48) | 37.09 (-14.86 to 68.78) | 40.97 (-14.44 to 71.07) | 1956323.97 (-612092.84 to 4033705.23) | 22.84 (-7.15 to 47.05) | 29.81 (-7.97 to 57.77) | -1.51 (-1.72 to -1.29) |
| **Enteric infections** | 4134309.48 (2908927.28 to 5122752.78) | 64.76 (45.57 to 80.25) | 1.75 (1.37 to 2.14) | 593959.88 (446774.05 to 835347.37) | 9.60 (7.22 to 13.50) | 0.94 (0.73 to 1.26) | -6.19 (-6.47 to -5.91) |
| Diarrheal diseases | 4134309.48 (2908927.28 to 5122752.78) | 64.76 (45.57 to 80.25) | 1.94 (1.55 to 2.35) | 593959.88 (446774.05 to 835347.37) | 9.60 (7.22 to 13.50) | 1.15 (0.88 to 1.49) | -6.19 (-6.47 to -5.91) |
| **Other infectious diseases** | 756411.52 (635478.71 to 948753.57) | 11.85 (9.95 to 14.86) | 0.47 (0.33 to 0.65) | 334394.98 (258055.71 to 436365.86) | 5.40 (4.17 to 7.05) | 0.91 (0.73 to 1.21) | -2.65 (-3.03 to -2.26) |
| Meningitis | 715795.51 (600123.43 to 911397.38) | 11.21 (9.40 to 14.27) | 2.04 (1.76 to 2.49) | 309052.23 (235708.82 to 409534.00) | 4.99 (3.81 to 6.62) | 2.40 (2.01 to 3.03) | -2.70 (-3.11 to -2.29) |
| Encephalitis | 40616.01 (29885.89 to 47086.45) | 0.64 (0.47 to 0.74) | 0.60 (0.50 to 0.72) | 25342.75 (19039.85 to 32694.56) | 0.41 (0.31 to 0.53) | 0.61 (0.51 to 0.72) | -1.85 (-2.03 to -1.68) |
| **Other non-communicable diseases** | 183423.34 (79812.57 to 315237.43) | 2.88 (1.25 to 4.95) | 0.11 (0.05 to 0.19) | 63713.25 (33411.31 to 94401.21) | 1.03 (0.54 to 1.52) | 0.05 (0.03 to 0.08) | -3.69 (-3.96 to -3.42) |
| Sudden infant death syndrome | 183423.34 (79812.57 to 315237.43) | 2.88 (1.25 to 4.95) | 2.63 (1.76 to 3.41) | 63713.25 (33411.31 to 94401.21) | 1.03 (0.54 to 1.52) | 2.33 (1.84 to 2.83) | -3.69 (-3.96 to -3.42) |

Table S5. Global DALYs attributable to ambient particulate matter pollution in 1990 and 2021, and estimated annual percentage changes from 1990 to 2021.

| **Cause of DALYs** | **1990** |  |  | **2021** |  |  | **1990–2021** |
| --- | --- | --- | --- | --- | --- | --- | --- |
|  | **Number of cases** | **Age-standardised rates per 100 000 people** | **Age-standardised PAF (%)** | **Number of cases** | **Age-standardised rates per 100 000 people** | **Age-standardised PAF (%)** | **EAPC in age-standardised rates (%)** |
| **All causes** | 77459727.96 (55118401.96 to 102684107.20) | 1716.81 (1240.07 to 2235.75) | 3.38 (2.41 to 4.45) | 120004672.01 (86560331.22 to 149810185.34) | 1483.61 (1069.48 to 1869.55) | 4.10 (2.98 to 5.13) | -0.28 (-0.43 to -0.12) |
| Female | 32027922.87 (22757013.61 to 42891498.26) | 1349.72 (957.36 to 1775.82) | 2.89 (2.03 to 3.87) | 48213500.68 (33756851.68 to 61650462.89) | 1138.28 (790.32 to 1475.56) | 3.49 (2.43 to 4.53) | -0.35 (-0.52 to -0.18) |
| Male | 45431805.09 (32419305.04 to 60456725.82) | 2130.95 (1529.03 to 2803.16) | 3.86 (2.75 to 5.09) | 71791171.33 (53219588.89 to 88960503.19) | 1871.56 (1386.55 to 2332.98) | 4.68 (3.49 to 5.74) | -0.22 (-0.37 to -0.07) |
| **Cardiovascular diseases** | 31409331.37 (22014519.12 to 40460986.80) | 829.57 (583.32 to 1070.32) | 10.99 (7.75 to 14.21) | 63295219.67 (45183884.54 to 78749625.44) | 740.66 (528.90 to 920.32) | 14.65 (10.44 to 18.14) | -0.26 (-0.39 to -0.12) |
| Ischemic heart disease | 18071404.49 (12480714.62 to 23961379.64) | 479.87 (328.35 to 640.66) | 15.44 (10.46 to 20.63) | 36515788.94 (25539478.57 to 48130575.91) | 427.81 (299.61 to 564.17) | 19.34 (13.57 to 25.17) | -0.30 (-0.46 to -0.13) |
| Stroke | 13337926.87 (9219590.30 to 18469720.18) | 349.71 (241.10 to 483.16) | 11.36 (7.99 to 15.55) | 26779430.73 (18076187.58 to 34223093.49) | 312.85 (211.45 to 399.79) | 16.59 (11.51 to 20.89) | -0.20 (-0.34 to -0.06) |
| Intracerebral hemorrhage | 6205569.48 (4050193.22 to 8958618.38) | 153.12 (99.69 to 221.12) | 10.09 (6.65 to 14.42) | 13249505.78 (8726215.06 to 17131011.76) | 153.27 (101.04 to 198.05) | 16.59 (11.13 to 20.99) | 0.24 (0.03 to 0.45) |
| Ischemic stroke | 5995852.81 (4230527.00 to 8130862.41) | 169.47 (119.47 to 229.77) | 13.17 (9.47 to 17.68) | 11998572.07 (8338820.48 to 15136954.73) | 141.82 (98.69 to 178.97) | 16.93 (12.15 to 21.15) | -0.45 (-0.58 to -0.32) |
| Subarachnoid hemorrhage | 1136504.59 (725374.86 to 1657604.70) | 27.12 (17.20 to 39.78) | 9.84 (6.63 to 14.37) | 1531352.88 (1031890.49 to 1965982.25) | 17.77 (11.98 to 22.81) | 14.20 (9.82 to 17.97) | -1.57 (-1.80 to -1.34) |
| **Chronic respiratory diseases** | 8182681.77 (5467615.88 to 11813479.44) | 216.26 (145.04 to 312.40) | 10.43 (7.08 to 14.84) | 17683775.82 (12693342.40 to 21774923.11) | 208.27 (149.56 to 256.57) | 16.09 (11.51 to 19.59) | -0.08 (-0.25 to 0.09) |
| Chronic obstructive pulmonary disease | 8182681.77 (5467615.88 to 11813479.44) | 216.26 (145.04 to 312.40) | 14.49 (9.85 to 20.40) | 17683775.82 (12693342.40 to 21774923.11) | 208.27 (149.56 to 256.57) | 22.14 (15.99 to 26.91) | -0.08 (-0.25 to 0.09) |
| **Maternal and neonatal disorders** | 13750594.24 (9142778.55 to 19004071.45) | 214.83 (142.84 to 296.90) | 4.54 (3.01 to 6.17) | 12672378.33 (7505545.97 to 19257737.91) | 204.81 (121.31 to 311.25) | 6.61 (3.96 to 9.59) | 0.19 (-0.03 to 0.42) |
| Neonatal disorders | 13750594.24 (9142778.55 to 19004071.45) | 214.83 (142.84 to 296.90) | 4.95 (3.27 to 6.70) | 12672378.33 (7505545.97 to 19257737.91) | 204.81 (121.31 to 311.25) | 6.96 (4.18 to 10.16) | 0.19 (-0.03 to 0.42) |
| Neonatal preterm birth | 7833737.87 (5194412.32 to 10526408.29) | 122.38 (81.15 to 164.44) | 6.24 (4.17 to 8.23) | 6549048.88 (3986905.37 to 9898491.63) | 105.85 (64.44 to 159.98) | 8.44 (5.25 to 11.82) | -0.04 (-0.28 to 0.19) |
| Neonatal encephalopathy due to birth asphyxia and trauma | 3268927.41 (2085193.70 to 4609276.04) | 51.05 (32.56 to 71.98) | 4.02 (2.58 to 5.67) | 3523320.14 (1981624.45 to 5408506.23) | 56.95 (32.03 to 87.42) | 6.11 (3.51 to 9.15) | 0.68 (0.44 to 0.91) |
| Other neonatal disorders | 1262568.94 (797420.33 to 1847604.51) | 19.73 (12.46 to 28.87) | 3.89 (2.59 to 5.57) | 1216566.54 (700119.62 to 1837529.31) | 19.66 (11.32 to 29.70) | 6.04 (3.71 to 8.92) | 0.09 (-0.12 to 0.29) |
| Neonatal sepsis and other neonatal infections | 1041344.81 (674951.12 to 1484892.60) | 16.29 (10.56 to 23.23) | 3.62 (2.42 to 5.02) | 1177161.59 (687184.57 to 1804263.69) | 19.02 (11.10 to 29.16) | 5.13 (3.07 to 7.52) | 0.69 (0.47 to 0.91) |
| Hemolytic disease and other neonatal jaundice | 344015.21 (216477.32 to 518490.02) | 5.38 (3.39 to 8.11) | 3.53 (2.32 to 5.15) | 206281.18 (119624.69 to 313931.32) | 3.33 (1.93 to 5.07) | 5.72 (3.40 to 8.37) | -1.20 (-1.50 to -0.90) |
| **Respiratory infections and tuberculosis** | 17786043.54 (3971807.70 to 30303497.28) | 307.51 (65.30 to 522.11) | 5.84 (1.28 to 10.07) | 10227580.03 (2076636.29 to 18221838.08) | 143.40 (31.62 to 254.88) | 3.29 (0.70 to 5.74) | -1.81 (-2.09 to -1.53) |
| Lower respiratory infections | 17780940.30 (3965508.09 to 30297308.21) | 307.43 (65.22 to 522.02) | 8.85 (1.92 to 15.32) | 10224991.17 (2073436.76 to 18218626.81) | 143.36 (31.57 to 254.84) | 12.25 (2.70 to 21.34) | -1.81 (-2.09 to -1.53) |
| Upper respiratory infections | 4864.38 (2198.29 to 9624.25) | 0.08 (0.03 to 0.15) | 0.08 (0.04 to 0.13) | 2511.13 (982.72 to 5804.58) | 0.04 (0.02 to 0.09) | 0.05 (0.03 to 0.10) | -1.63 (-1.94 to -1.33) |
| Otitis media | 238.87 (105.30 to 484.51) | 0.00 (0.00 to 0.01) | 0.01 (0.00 to 0.02) | 77.73 (36.35 to 143.79) | 0.00 (0.00 to 0.00) | 0.00 (0.00 to 0.01) | -2.74 (-3.59 to -1.89) |
| **Diabetes and kidney diseases** | 2155320.74 (1207386.90 to 3247825.78) | 54.24 (30.49 to 81.95) | 4.69 (2.67 to 6.86) | 8926684.25 (4945568.75 to 13796527.48) | 103.05 (57.06 to 159.22) | 7.09 (4.13 to 10.50) | 2.12 (2.04 to 2.20) |
| Diabetes mellitus | 2155320.74 (1207386.90 to 3247825.78) | 54.24 (30.49 to 81.95) | 8.17 (4.71 to 11.94) | 8926684.25 (4945568.75 to 13796527.48) | 103.05 (57.06 to 159.22) | 11.23 (6.41 to 16.46) | 2.12 (2.04 to 2.20) |
| Diabetes mellitus type 2 | 2155320.74 (1207386.90 to 3247825.78) | 54.24 (30.49 to 81.95) | 8.85 (5.11 to 12.98) | 8926684.25 (4945568.75 to 13796527.48) | 103.05 (57.06 to 159.22) | 11.81 (6.76 to 17.32) | 2.12 (2.04 to 2.20) |
| **Neoplasms** | 3344737.85 (1965943.04 to 4895086.50) | 81.37 (47.85 to 119.20) | 2.05 (1.20 to 3.02) | 6964530.94 (4284313.50 to 9719776.98) | 79.63 (48.96 to 111.17) | 2.69 (1.68 to 3.84) | 0.19 (0.05 to 0.33) |
| Tracheal, bronchus, and lung cancer | 3344737.85 (1965943.04 to 4895086.50) | 81.37 (47.85 to 119.20) | 11.78 (6.99 to 17.53) | 6964530.94 (4284313.50 to 9719776.98) | 79.63 (48.96 to 111.17) | 14.94 (9.34 to 21.01) | 0.19 (0.05 to 0.33) |
| **Enteric infections** | 673119.68 (411147.42 to 1023611.88) | 10.54 (6.44 to 16.04) | 0.29 (0.18 to 0.43) | 141405.64 (81663.07 to 227931.88) | 2.28 (1.32 to 3.68) | 0.22 (0.13 to 0.35) | -4.54 (-4.76 to -4.32) |
| Diarrheal diseases | 673119.68 (411147.42 to 1023611.88) | 10.54 (6.44 to 16.04) | 0.32 (0.20 to 0.47) | 141405.64 (81663.07 to 227931.88) | 2.28 (1.32 to 3.68) | 0.27 (0.16 to 0.42) | -4.54 (-4.76 to -4.32) |
| **Other infectious diseases** | 123134.34 (80419.09 to 176887.03) | 1.93 (1.26 to 2.77) | 0.08 (0.04 to 0.12) | 73777.47 (43455.00 to 119629.60) | 1.19 (0.70 to 1.93) | 0.20 (0.11 to 0.33) | -1.05 (-1.36 to -0.73) |
| Meningitis | 115480.56 (75272.76 to 167425.85) | 1.81 (1.18 to 2.62) | 0.33 (0.22 to 0.48) | 66416.36 (38566.62 to 109218.11) | 1.07 (0.62 to 1.76) | 0.52 (0.30 to 0.84) | -1.12 (-1.46 to -0.79) |
| Encephalitis | 7653.77 (4908.54 to 10669.72) | 0.12 (0.08 to 0.17) | 0.11 (0.07 to 0.16) | 7361.11 (4481.45 to 10877.36) | 0.12 (0.07 to 0.18) | 0.18 (0.11 to 0.25) | -0.13 (-0.34 to 0.08) |
| **Other non-communicable diseases** | 34764.44 (17671.42 to 59711.48) | 0.55 (0.28 to 0.94) | 0.02 (0.01 to 0.03) | 19319.87 (9727.68 to 32118.40) | 0.31 (0.16 to 0.52) | 0.02 (0.01 to 0.03) | -1.67 (-1.84 to -1.51) |
| Sudden infant death syndrome | 34764.44 (17671.42 to 59711.48) | 0.55 (0.28 to 0.94) | 0.51 (0.34 to 0.71) | 19319.87 (9727.68 to 32118.40) | 0.31 (0.16 to 0.52) | 0.71 (0.46 to 1.00) | -1.67 (-1.84 to -1.51) |

Table S6. Global DALYs attributable to household particulate matter pollution in 1990 and 2021, and estimated annual percentage changes from 1990 to 2021.

| **Cause of DALYs** | **1990** |  |  | **2021** |  |  | **1990–2021** |
| --- | --- | --- | --- | --- | --- | --- | --- |
|  | **Number of cases** | **Age-standardised rates per 100 000 people** | **Age-standardised PAF (%)** | **Number of cases** | **Age-standardised rates per 100 000 people** | **Age-standardised PAF (%)** | **EAPC in age-standardised rates (%)** |
| **All causes** | 211860686.87 (154596435.41 to 265090267.09) | 4147.68 (3101.41 to 5104.55) | 8.18 (5.99 to 10.02) | 111462958.29 (75085852.45 to 163710710.53) | 1500.29 (1028.38 to 2195.56) | 4.15 (2.79 to 5.97) | -3.52 (-3.77 to -3.27) |
| Female | 98467661.17 (69539490.64 to 124514327.01) | 3780.53 (2792.17 to 4718.77) | 8.10 (5.74 to 9.99) | 50815051.99 (33334557.20 to 75188150.33) | 1341.48 (900.54 to 1944.46) | 4.11 (2.73 to 5.88) | -3.54 (-3.77 to -3.30) |
| Male | 113393025.69 (84356036.01 to 141234869.55) | 4574.38 (3511.07 to 5619.10) | 8.29 (6.24 to 10.19) | 60647906.30 (41429685.65 to 90336007.43) | 1663.57 (1140.02 to 2428.02) | 4.15 (2.83 to 6.10) | -3.53 (-3.80 to -3.26) |
| **Maternal and neonatal disorders** | 57950246.89 (51283700.91 to 64491477.52) | 905.40 (801.24 to 1007.53) | 19.15 (17.07 to 21.06) | 32055710.45 (25371698.77 to 39702119.10) | 518.10 (410.06 to 641.68) | 16.73 (13.91 to 19.86) | -2.07 (-2.24 to -1.89) |
| Neonatal disorders | 57950246.89 (51283700.91 to 64491477.52) | 905.40 (801.24 to 1007.53) | 20.85 (18.57 to 22.97) | 32055710.45 (25371698.77 to 39702119.10) | 518.10 (410.06 to 641.68) | 17.61 (14.63 to 20.86) | -2.07 (-2.24 to -1.89) |
| Neonatal preterm birth | 28094255.28 (24429124.72 to 32201737.84) | 438.93 (381.72 to 503.08) | 22.37 (19.85 to 24.94) | 13941304.53 (10685511.42 to 17714113.66) | 225.33 (172.70 to 286.31) | 17.97 (14.63 to 21.57) | -2.36 (-2.49 to -2.22) |
| Neonatal encephalopathy due to birth asphyxia and trauma | 16367852.41 (14007640.43 to 19179521.19) | 255.60 (218.73 to 299.48) | 20.11 (17.93 to 22.31) | 10453924.92 (8134819.89 to 13105981.31) | 168.97 (131.49 to 211.84) | 18.13 (14.99 to 21.27) | -1.63 (-1.87 to -1.38) |
| Neonatal sepsis and other neonatal infections | 5312457.96 (4557441.60 to 6202776.96) | 83.10 (71.28 to 97.02) | 18.48 (16.42 to 20.52) | 3683937.55 (2913626.17 to 4604471.93) | 59.53 (47.08 to 74.41) | 16.06 (13.63 to 18.67) | -1.39 (-1.57 to -1.20) |
| Other neonatal disorders | 6442898.47 (4392711.07 to 8235189.37) | 100.67 (68.64 to 128.66) | 19.77 (17.18 to 22.21) | 3502769.71 (2341383.41 to 4496747.27) | 56.61 (37.84 to 72.68) | 17.30 (14.21 to 20.30) | -2.26 (-2.49 to -2.03) |
| Hemolytic disease and other neonatal jaundice | 1732782.76 (1414396.09 to 2217544.40) | 27.11 (22.13 to 34.68) | 17.77 (15.49 to 19.68) | 473773.75 (337192.18 to 682468.43) | 7.66 (5.45 to 11.03) | 13.09 (10.15 to 16.09) | -4.19 (-4.45 to -3.94) |
| **Cardiovascular diseases** | 47455419.97 (37623080.46 to 58625279.36) | 1180.13 (930.98 to 1464.78) | 15.63 (12.42 to 19.42) | 36325996.23 (20978352.52 to 61347661.86) | 420.92 (242.75 to 713.21) | 8.33 (4.79 to 14.33) | -3.65 (-4.01 to -3.29) |
| Ischemic heart disease | 18492313.47 (13766442.72 to 24264678.32) | 456.40 (337.88 to 603.97) | 14.69 (11.01 to 19.24) | 18150787.17 (10274368.81 to 31394757.25) | 210.56 (118.90 to 365.27) | 9.52 (5.43 to 16.49) | -2.74 (-3.08 to -2.39) |
| Stroke | 28963106.50 (22528486.69 to 36117913.11) | 723.74 (562.98 to 904.58) | 23.51 (18.37 to 28.94) | 18175209.06 (10671998.53 to 30875847.53) | 210.35 (123.22 to 358.01) | 11.16 (6.40 to 19.31) | -4.38 (-4.77 to -3.98) |
| Intracerebral hemorrhage | 17864178.69 (13880659.54 to 21806845.90) | 437.28 (340.54 to 533.74) | 28.83 (22.84 to 34.75) | 10761863.82 (6305255.34 to 17884173.26) | 123.61 (72.34 to 205.70) | 13.38 (7.76 to 22.35) | -4.42 (-4.84 to -4.00) |
| Ischemic stroke | 8144118.86 (6138196.60 to 10702875.33) | 215.73 (162.27 to 282.62) | 16.77 (12.71 to 21.92) | 6293712.32 (3469052.03 to 11655620.60) | 73.78 (40.65 to 136.68) | 8.82 (4.90 to 16.50) | -3.88 (-4.31 to -3.45) |
| Subarachnoid hemorrhage | 2954808.95 (1918539.15 to 4030113.20) | 70.73 (45.12 to 96.73) | 25.52 (19.34 to 31.54) | 1119632.92 (575794.55 to 1959215.25) | 12.96 (6.67 to 22.68) | 10.29 (5.50 to 17.36) | -6.12 (-6.34 to -5.89) |
| **Respiratory infections and tuberculosis** | 69542855.18 (16874582.06 to 113052650.85) | 1154.88 (274.68 to 1869.55) | 21.91 (5.60 to 33.95) | 18876097.62 (5148727.42 to 33540360.21) | 276.78 (78.39 to 489.70) | 6.34 (1.75 to 11.08) | -4.48 (-4.74 to -4.22) |
| Lower respiratory infections | 69522816.15 (16850710.37 to 113037433.13) | 1154.57 (274.31 to 1869.31) | 33.21 (8.59 to 51.08) | 18866652.62 (5139689.45 to 33524607.68) | 276.63 (78.26 to 489.44) | 23.58 (6.77 to 39.84) | -4.48 (-4.74 to -4.22) |
| Upper respiratory infections | 18914.97 (3861.43 to 39761.06) | 0.30 (0.06 to 0.62) | 0.28 (0.08 to 0.53) | 9195.39 (1616.43 to 22783.04) | 0.15 (0.03 to 0.37) | 0.18 (0.04 to 0.40) | -2.50 (-2.60 to -2.41) |
| Otitis media | 1124.07 (381.90 to 2569.97) | 0.02 (0.01 to 0.04) | 0.05 (0.02 to 0.12) | 249.61 (110.04 to 520.97) | 0.00 (0.00 to 0.01) | 0.01 (0.01 to 0.03) | -4.94 (-5.11 to -4.77) |
| **Chronic respiratory diseases** | 25325454.14 (20283268.55 to 30207187.80) | 661.17 (528.97 to 790.08) | 31.86 (26.04 to 37.74) | 15550114.50 (9559902.65 to 25344665.29) | 181.17 (111.06 to 296.85) | 14.01 (8.62 to 23.59) | -4.57 (-4.85 to -4.29) |
| Chronic obstructive pulmonary disease | 25325454.14 (20283268.55 to 30207187.80) | 661.17 (528.97 to 790.08) | 44.27 (36.90 to 51.46) | 15550114.50 (9559902.65 to 25344665.29) | 181.17 (111.06 to 296.85) | 19.27 (11.80 to 32.03) | -4.57 (-4.85 to -4.29) |
| **Diabetes and kidney diseases** | 2412269.39 (1400628.62 to 3583798.97) | 58.41 (33.91 to 86.44) | 5.06 (2.92 to 7.40) | 3974916.65 (1861591.30 to 7234172.48) | 45.84 (21.48 to 83.46) | 3.16 (1.55 to 5.69) | -1.12 (-1.36 to -0.88) |
| Diabetes mellitus | 2412269.39 (1400628.62 to 3583798.97) | 58.41 (33.91 to 86.44) | 8.81 (5.10 to 13.03) | 3974916.65 (1861591.30 to 7234172.48) | 45.84 (21.48 to 83.46) | 5.01 (2.43 to 8.82) | -1.12 (-1.36 to -0.88) |
| Diabetes mellitus type 2 | 2412269.39 (1400628.62 to 3583798.97) | 58.41 (33.91 to 86.44) | 9.53 (5.51 to 14.09) | 3974916.65 (1861591.30 to 7234172.48) | 45.84 (21.48 to 83.46) | 5.26 (2.56 to 9.26) | -1.12 (-1.36 to -0.88) |
| **Sense organ diseases** | 1414029.27 (-564248.53 to 2610528.48) | 37.09 (-14.86 to 68.78) | 4.26 (-1.31 to 7.74) | 1956323.97 (-612092.84 to 4033705.23) | 22.84 (-7.15 to 47.05) | 2.54 (-0.60 to 5.17) | -1.51 (-1.72 to -1.29) |
| Blindness and vision loss | 1414029.27 (-564248.53 to 2610528.48) | 37.09 (-14.86 to 68.78) | 11.03 (-3.55 to 20.34) | 1956323.97 (-612092.84 to 4033705.23) | 22.84 (-7.15 to 47.05) | 6.83 (-1.66 to 14.11) | -1.51 (-1.72 to -1.29) |
| Cataract | 1414029.27 (-564248.53 to 2610528.48) | 37.09 (-14.86 to 68.78) | 40.97 (-14.44 to 71.07) | 1956323.97 (-612092.84 to 4033705.23) | 22.84 (-7.15 to 47.05) | 29.81 (-7.97 to 57.77) | -1.51 (-1.72 to -1.29) |
| **Neoplasms** | 3517878.29 (2180370.13 to 4954257.07) | 84.14 (52.12 to 118.39) | 2.12 (1.35 to 2.97) | 1966425.73 (758404.89 to 4632387.15) | 22.41 (8.64 to 52.88) | 0.76 (0.29 to 1.81) | -4.77 (-5.32 to -4.21) |
| Tracheal, bronchus, and lung cancer | 3517878.29 (2180370.13 to 4954257.07) | 84.14 (52.12 to 118.39) | 12.17 (7.73 to 17.17) | 1966425.73 (758404.89 to 4632387.15) | 22.41 (8.64 to 52.88) | 4.21 (1.60 to 9.70) | -4.77 (-5.32 to -4.21) |
| **Enteric infections** | 3460714.36 (2349318.57 to 4437822.36) | 54.21 (36.80 to 69.51) | 1.46 (1.13 to 1.80) | 452439.57 (322003.73 to 644502.81) | 7.31 (5.20 to 10.41) | 0.72 (0.54 to 0.99) | -6.60 (-6.95 to -6.26) |
| Diarrheal diseases | 3460714.36 (2349318.57 to 4437822.36) | 54.21 (36.80 to 69.51) | 1.62 (1.28 to 1.98) | 452439.57 (322003.73 to 644502.81) | 7.31 (5.20 to 10.41) | 0.88 (0.67 to 1.18) | -6.60 (-6.95 to -6.26) |
| **Other infectious diseases** | 633189.63 (516970.10 to 806881.85) | 9.92 (8.10 to 12.63) | 0.39 (0.27 to 0.54) | 260555.26 (199030.30 to 356031.19) | 4.21 (3.22 to 5.75) | 0.71 (0.55 to 0.94) | -3.03 (-3.47 to -2.58) |
| Meningitis | 600233.77 (491690.95 to 774511.57) | 9.40 (7.70 to 12.13) | 1.71 (1.46 to 2.15) | 242577.74 (184232.87 to 336532.33) | 3.92 (2.98 to 5.44) | 1.88 (1.52 to 2.42) | -3.07 (-3.53 to -2.60) |
| Encephalitis | 32955.86 (24101.26 to 38962.39) | 0.52 (0.38 to 0.61) | 0.49 (0.40 to 0.59) | 17977.52 (12891.48 to 24325.78) | 0.29 (0.21 to 0.39) | 0.43 (0.33 to 0.54) | -2.40 (-2.62 to -2.18) |
| **Other non-communicable diseases** | 148629.74 (59484.99 to 262262.04) | 2.33 (0.93 to 4.12) | 0.09 (0.04 to 0.16) | 44378.31 (22028.67 to 67779.01) | 0.72 (0.36 to 1.09) | 0.04 (0.02 to 0.06) | -4.33 (-4.70 to -3.96) |
| Sudden infant death syndrome | 148629.74 (59484.99 to 262262.04) | 2.33 (0.93 to 4.12) | 2.12 (1.31 to 2.85) | 44378.31 (22028.67 to 67779.01) | 0.72 (0.36 to 1.09) | 1.62 (1.20 to 2.11) | -4.33 (-4.70 to -3.96) |

Table S7. Number and age-standardised rates of deaths attributable to ambient particulate matter pollution in 1990 and 2021, and estimated annual percentage changes from 1990 to 2021, by region.

| **Cause of deaths** | **1990** |  |  | **2021** |  |  | **1990–2021** |
| --- | --- | --- | --- | --- | --- | --- | --- |
|  | **Number of cases** | **Age-standardised rates per 100 000 people** | **Age-standardised PAF (%)** | **Number of cases** | **Age-standardised rates per 100 000 people** | **Age-standardised PAF (%)** | **EAPC in age-standardised rates (%)** |
| **Global** | 2433557.86 (1750350.02 to 3155644.05) | 66.95 (48.23 to 86.33) | 6.05 (4.42 to 7.76) | 4718812.24 (3480471.83 to 5795946.44) | 57.62 (42.32 to 70.99) | 6.90 (5.13 to 8.41) | -0.34 (-0.48 to -0.20) |
| High SDI | 575501.92 (378505.58 to 799345.49) | 52.60 (34.73 to 73.01) | 7.59 (5.00 to 10.51) | 381316.34 (280395.75 to 493910.70) | 16.58 (12.52 to 21.24) | 3.46 (2.61 to 4.42) | -3.91 (-4.04 to -3.79) |
| High-middle SDI | 801073.30 (564288.39 to 1068526.50) | 94.99 (66.72 to 125.85) | 9.76 (6.90 to 13.01) | 1232808.48 (966175.94 to 1499447.50) | 64.36 (50.50 to 78.17) | 9.32 (7.42 to 11.05) | -1.24 (-1.46 to -1.03) |
| Middle SDI | 590560.65 (387915.11 to 829680.19) | 65.17 (43.42 to 92.67) | 5.89 (3.90 to 8.33) | 1959596.74 (1348370.38 to 2379509.29) | 83.81 (57.81 to 101.88) | 10.24 (7.14 to 12.32) | 1.14 (0.95 to 1.32) |
| Low-middle SDI | 328612.50 (204935.77 to 471927.42) | 48.33 (32.08 to 68.78) | 3.51 (2.31 to 4.95) | 924841.16 (581045.84 to 1293372.65) | 71.21 (44.82 to 99.49) | 6.19 (3.86 to 8.51) | 1.66 (1.36 to 1.96) |
| Low SDI | 134249.04 (81423.83 to 197304.98) | 41.69 (26.77 to 59.93) | 2.29 (1.47 to 3.25) | 216865.84 (140237.56 to 322116.26) | 41.23 (27.24 to 58.86) | 2.79 (1.84 to 3.94) | 0.42 (0.04 to 0.80) |
| Andean Latin America | 18774.80 (8442.99 to 31180.48) | 78.18 (37.42 to 128.72) | 8.64 (4.04 to 14.13) | 21844.98 (13160.77 to 32593.86) | 38.43 (23.15 to 57.45) | 3.95 (2.39 to 5.59) | -2.65 (-2.95 to -2.36) |
| Australasia | 3640.55 (171.82 to 9808.43) | 16.14 (0.91 to 43.21) | 2.48 (0.14 to 6.60) | 4859.69 (2915.27 to 7147.92) | 8.08 (4.91 to 11.83) | 2.19 (1.33 to 3.20) | -2.80 (-3.34 to -2.26) |
| Caribbean | 12103.78 (4886.70 to 22578.44) | 48.89 (19.63 to 91.65) | 5.06 (2.02 to 9.54) | 20880.04 (11124.85 to 32755.10) | 38.83 (20.85 to 60.94) | 4.14 (2.19 to 6.44) | -0.61 (-0.80 to -0.42) |
| Central Asia | 40799.24 (16134.59 to 73712.61) | 87.18 (34.63 to 158.67) | 8.59 (3.42 to 15.71) | 65383.60 (45936.53 to 83789.63) | 94.94 (66.91 to 121.64) | 9.69 (6.86 to 12.39) | 0.76 (0.32 to 1.21) |
| Central Europe | 158042.85 (82028.29 to 239740.43) | 118.25 (60.87 to 179.02) | 11.80 (6.08 to 17.87) | 109551.31 (86364.71 to 134954.86) | 46.94 (36.89 to 57.80) | 5.94 (4.71 to 7.33) | -2.93 (-3.16 to -2.69) |
| Central Latin America | 57875.43 (31740.28 to 85270.33) | 67.43 (36.91 to 99.83) | 7.84 (4.28 to 11.56) | 75926.84 (53427.47 to 100314.19) | 31.99 (22.47 to 42.20) | 3.75 (2.67 to 4.88) | -2.55 (-2.74 to -2.36) |
| Central Sub-Saharan Africa | 9459.97 (5018.47 to 16114.47) | 33.35 (17.85 to 54.15) | 1.75 (0.98 to 2.84) | 18228.43 (10927.34 to 27526.83) | 34.55 (20.99 to 52.42) | 2.04 (1.26 to 3.05) | 0.50 (0.22 to 0.78) |
| East Asia | 463585.96 (223158.93 to 815441.34) | 73.87 (35.09 to 129.34) | 6.24 (3.00 to 10.82) | 1876621.31 (1316734.03 to 2314102.49) | 99.21 (69.82 to 122.37) | 15.46 (10.88 to 18.26) | 1.48 (1.06 to 1.89) |
| Eastern Europe | 338332.87 (176807.69 to 504191.22) | 137.94 (72.57 to 205.56) | 13.58 (7.14 to 20.25) | 164771.28 (105176.05 to 239281.85) | 46.50 (29.74 to 67.38) | 4.54 (2.90 to 6.62) | -4.17 (-4.58 to -3.75) |
| Eastern Sub-Saharan Africa | 22500.68 (13685.27 to 33634.52) | 19.41 (12.87 to 28.71) | 0.97 (0.65 to 1.41) | 31226.29 (19448.25 to 47585.91) | 17.23 (10.68 to 26.00) | 1.07 (0.68 to 1.58) | 0.03 (-0.12 to 0.19) |
| High-income Asia Pacific | 57689.25 (17160.32 to 111790.36) | 32.34 (9.65 to 62.94) | 5.46 (1.63 to 10.61) | 76371.10 (44575.85 to 110347.97) | 12.93 (7.71 to 18.19) | 4.03 (2.40 to 5.66) | -3.07 (-3.36 to -2.78) |
| High-income North America | 144468.64 (61191.92 to 244155.64) | 40.19 (17.21 to 67.67) | 6.02 (2.57 to 10.14) | 53331.60 (25904.67 to 84386.80) | 7.75 (3.84 to 12.15) | 1.32 (0.66 to 2.08) | -5.77 (-6.22 to -5.32) |
| North Africa and Middle East | 203052.10 (148290.79 to 252503.96) | 119.23 (87.82 to 149.15) | 10.20 (7.52 to 12.62) | 394470.44 (322582.52 to 464021.19) | 102.51 (83.74 to 120.94) | 10.34 (8.59 to 12.05) | -0.32 (-0.44 to -0.20) |
| Oceania | 863.06 (249.97 to 2167.55) | 30.55 (8.79 to 73.64) | 2.18 (0.69 to 5.10) | 2388.69 (839.18 to 5203.50) | 35.90 (12.66 to 77.24) | 2.59 (0.91 to 5.41) | 0.43 (0.25 to 0.60) |
| South Asia | 294654.92 (159412.99 to 476520.35) | 46.04 (24.89 to 72.92) | 3.32 (1.81 to 5.25) | 1105541.56 (709645.97 to 1495436.31) | 85.06 (54.60 to 114.91) | 7.79 (4.98 to 10.35) | 2.39 (1.96 to 2.82) |
| Southeast Asia | 107684.97 (48368.22 to 189316.80) | 43.56 (19.62 to 76.66) | 3.80 (1.75 to 6.75) | 332199.12 (210740.53 to 449192.65) | 58.94 (37.36 to 79.50) | 6.27 (3.80 to 8.41) | 0.75 (0.61 to 0.90) |
| Southern Latin America | 23956.74 (12150.77 to 39355.33) | 55.67 (28.31 to 91.70) | 6.81 (3.46 to 11.20) | 22971.00 (12721.69 to 34217.81) | 25.84 (14.55 to 38.47) | 4.08 (2.30 to 6.05) | -2.36 (-2.56 to -2.16) |
| Southern Sub-Saharan Africa | 16290.58 (9889.08 to 22824.29) | 52.72 (33.69 to 72.31) | 4.61 (2.93 to 6.33) | 31050.50 (21874.84 to 40518.08) | 60.32 (42.21 to 78.99) | 3.35 (2.34 to 4.30) | 0.86 (0.48 to 1.24) |
| Tropical Latin America | 39400.85 (14052.37 to 72050.09) | 45.84 (16.30 to 85.38) | 4.99 (1.78 to 9.32) | 54180.44 (30985.00 to 79095.82) | 21.99 (12.57 to 32.05) | 2.95 (1.70 to 4.29) | -2.36 (-2.54 to -2.17) |
| Western Europe | 338297.99 (175210.48 to 533610.34) | 57.42 (29.99 to 90.36) | 8.43 (4.40 to 13.25) | 128586.15 (90660.24 to 171069.59) | 11.47 (8.21 to 15.09) | 2.67 (1.91 to 3.51) | -5.30 (-5.51 to -5.09) |
| Western Sub-Saharan Africa | 82082.61 (44496.45 to 124440.03) | 58.54 (33.80 to 86.45) | 3.52 (2.04 to 5.24) | 128427.87 (71842.29 to 210404.64) | 53.31 (30.42 to 83.12) | 3.74 (2.19 to 5.76) | 0.47 (0.06 to 0.89) |

Table S8. Number and age-standardised rates of DALYs attributable to ambient particulate matter pollution in 1990 and 2021, and estimated annual percentage changes from 1990 to 2021, by region.

| **Cause of DALYs** | **1990** |  |  | **2021** |  |  | **1990–2021** |
| --- | --- | --- | --- | --- | --- | --- | --- |
|  | **Number of cases** | **Age-standardised rates per 100 000 people** | **Age-standardised PAF (%)** | **Number of cases** | **Age-standardised rates per 100 000 people** | **Age-standardised PAF (%)** | **EAPC in age-standardised rates (%)** |
| **Global** | 77459727.96 (55118401.96 to 102684107.20) | 1716.81 (1240.07 to 2235.75) | 3.38 (2.41 to 4.45) | 120004672.01 (86560331.22 to 149810185.34) | 1483.61 (1069.48 to 1869.55) | 4.10 (2.98 to 5.13) | -0.28 (-0.43 to -0.12) |
| High SDI | 12133360.31 (8398970.30 to 16479045.21) | 1157.40 (809.80 to 1557.67) | 4.15 (2.84 to 5.54) | 8190411.68 (6171836.31 to 10298943.46) | 429.14 (331.87 to 534.35) | 1.90 (1.47 to 2.42) | -3.35 (-3.44 to -3.26) |
| High-middle SDI | 19499442.98 (13855298.32 to 25710897.55) | 2087.25 (1479.51 to 2745.22) | 5.58 (3.90 to 7.46) | 25550504.46 (20281517.62 to 30717554.97) | 1372.34 (1094.84 to 1641.94) | 5.33 (4.28 to 6.34) | -1.38 (-1.59 to -1.17) |
| Middle SDI | 21703176.48 (13692864.24 to 31028287.90) | 1704.19 (1110.22 to 2377.98) | 3.73 (2.39 to 5.33) | 47638288.17 (32828319.75 to 57838741.67) | 1941.77 (1329.13 to 2353.31) | 6.12 (4.26 to 7.49) | 0.72 (0.55 to 0.89) |
| Low-middle SDI | 15990862.74 (9661878.79 to 23729962.68) | 1493.26 (927.66 to 2152.52) | 2.33 (1.47 to 3.37) | 28774252.51 (17494206.11 to 40814436.06) | 1887.34 (1155.59 to 2660.68) | 4.21 (2.64 to 5.91) | 1.16 (0.87 to 1.46) |
| Low SDI | 8044069.08 (4656296.56 to 12188498.11) | 1399.55 (871.53 to 2049.07) | 1.59 (1.00 to 2.35) | 9771098.45 (6154944.88 to 15195026.90) | 1137.42 (739.90 to 1690.95) | 1.97 (1.27 to 2.84) | -0.20 (-0.53 to 0.14) |
| Andean Latin America | 877928.29 (372442.05 to 1489230.15) | 2443.55 (1083.97 to 4056.01) | 5.50 (2.39 to 9.29) | 582616.33 (361645.32 to 841865.14) | 980.46 (608.57 to 1414.74) | 2.77 (1.73 to 3.89) | -3.21 (-3.48 to -2.94) |
| Australasia | 75686.01 (7323.94 to 196219.75) | 336.08 (37.12 to 852.63) | 1.24 (0.14 to 3.14) | 94481.84 (57262.64 to 135858.11) | 183.19 (112.47 to 259.73) | 0.94 (0.56 to 1.36) | -2.43 (-3.00 to -1.87) |
| Caribbean | 349259.21 (142458.45 to 646048.72) | 1198.12 (485.34 to 2229.86) | 2.65 (1.07 to 4.88) | 537061.56 (293506.84 to 812505.90) | 1051.89 (588.58 to 1572.55) | 2.57 (1.48 to 3.88) | -0.23 (-0.41 to -0.05) |
| Central Asia | 1446058.32 (580167.23 to 2673997.25) | 2388.81 (962.33 to 4333.63) | 5.48 (2.24 to 9.81) | 1785384.78 (1247429.01 to 2292821.13) | 2200.39 (1541.49 to 2812.33) | 6.16 (4.31 to 7.92) | 0.17 (-0.23 to 0.56) |
| Central Europe | 3577062.11 (1860235.09 to 5362026.76) | 2607.32 (1354.35 to 3862.29) | 7.14 (3.74 to 10.57) | 2142048.77 (1703642.58 to 2617366.02) | 1009.50 (799.36 to 1229.35) | 3.58 (2.79 to 4.46) | -2.99 (-3.21 to -2.77) |
| Central Latin America | 2352168.28 (1271270.03 to 3475921.17) | 1831.88 (1001.01 to 2707.58) | 4.68 (2.58 to 6.90) | 2024365.67 (1417298.26 to 2641894.22) | 834.34 (586.37 to 1089.87) | 2.41 (1.68 to 3.13) | -2.65 (-2.81 to -2.48) |
| Central Sub-Saharan Africa | 539736.40 (271812.72 to 935300.44) | 980.04 (529.04 to 1654.77) | 1.09 (0.59 to 1.80) | 803625.77 (466393.01 to 1242052.00) | 905.12 (547.99 to 1357.32) | 1.44 (0.89 to 2.16) | 0.19 (-0.06 to 0.44) |
| East Asia | 13115958.63 (6363263.58 to 23257894.34) | 1576.55 (761.29 to 2786.61) | 3.70 (1.77 to 6.48) | 38253731.46 (26648475.20 to 47048423.30) | 1919.39 (1341.28 to 2360.59) | 8.44 (5.98 to 10.20) | 1.13 (0.80 to 1.47) |
| Eastern Europe | 7394478.50 (3967696.18 to 10931748.72) | 2894.20 (1595.03 to 4261.03) | 7.44 (3.98 to 11.06) | 3282509.32 (2124591.17 to 4773479.66) | 974.14 (637.78 to 1407.02) | 2.65 (1.72 to 3.86) | -4.21 (-4.65 to -3.77) |
| Eastern Sub-Saharan Africa | 1378973.51 (782084.35 to 2114263.47) | 644.03 (405.54 to 945.34) | 0.68 (0.43 to 0.99) | 1444116.77 (902338.85 to 2224813.71) | 477.76 (304.33 to 723.15) | 0.79 (0.51 to 1.20) | -0.50 (-0.66 to -0.33) |
| High-income Asia Pacific | 1329599.41 (430921.88 to 2475940.81) | 713.63 (242.35 to 1318.03) | 2.99 (1.00 to 5.71) | 1573929.43 (990270.71 to 2192201.27) | 362.67 (239.27 to 491.68) | 2.21 (1.42 to 3.08) | -2.34 (-2.62 to -2.05) |
| High-income North America | 3071980.13 (1345420.66 to 5075572.94) | 908.95 (409.41 to 1482.29) | 3.17 (1.39 to 5.25) | 1272912.15 (638963.42 to 2031469.05) | 211.90 (111.01 to 328.72) | 0.76 (0.41 to 1.18) | -5.10 (-5.48 to -4.71) |
| North Africa and Middle East | 8597395.99 (6057909.81 to 11218610.33) | 3218.20 (2346.88 to 4007.37) | 6.32 (4.72 to 7.94) | 11150525.90 (9093690.53 to 13160922.86) | 2399.07 (1971.08 to 2820.32) | 6.55 (5.46 to 7.67) | -0.76 (-0.89 to -0.62) |
| Oceania | 37433.65 (10836.86 to 96634.41) | 814.56 (235.73 to 2026.86) | 1.44 (0.45 to 3.56) | 88889.30 (30837.76 to 197691.42) | 924.95 (326.70 to 2013.49) | 1.79 (0.65 to 3.75) | 0.37 (0.22 to 0.54) |
| South Asia | 14665716.55 (7455963.93 to 24458052.79) | 1482.38 (797.84 to 2406.60) | 2.30 (1.26 to 3.73) | 33371055.11 (21122802.27 to 45825015.96) | 2266.08 (1436.63 to 3105.10) | 5.28 (3.36 to 7.10) | 1.74 (1.34 to 2.15) |
| Southeast Asia | 4239309.48 (1902120.06 to 7520997.08) | 1227.02 (549.62 to 2149.50) | 2.47 (1.11 to 4.37) | 9242662.70 (5835475.96 to 12510274.81) | 1458.03 (919.19 to 1973.88) | 4.15 (2.51 to 5.60) | 0.33 (0.19 to 0.47) |
| Southern Latin America | 617131.83 (327537.44 to 994282.18) | 1334.91 (706.79 to 2150.41) | 4.02 (2.09 to 6.55) | 516030.04 (307838.16 to 741428.23) | 632.91 (388.65 to 892.56) | 2.40 (1.45 to 3.43) | -2.43 (-2.63 to -2.24) |
| Southern Sub-Saharan Africa | 771327.43 (427409.98 to 1156610.61) | 1695.32 (1016.78 to 2397.69) | 3.04 (1.84 to 4.32) | 1063840.76 (724225.51 to 1424493.85) | 1686.17 (1163.61 to 2217.86) | 2.49 (1.75 to 3.26) | 0.41 (0.08 to 0.75) |
| Tropical Latin America | 1392600.07 (547067.34 to 2397804.24) | 1262.02 (462.58 to 2228.05) | 2.96 (1.09 to 5.35) | 1429168.16 (859585.12 to 2058412.52) | 580.68 (357.80 to 831.22) | 1.76 (1.03 to 2.49) | -2.56 (-2.76 to -2.37) |
| Western Europe | 6456301.90 (3393694.27 to 9919490.91) | 1168.43 (632.02 to 1773.75) | 4.37 (2.31 to 6.61) | 2378504.32 (1696295.69 to 3135763.04) | 265.63 (192.90 to 348.65) | 1.32 (0.96 to 1.76) | -4.90 (-5.07 to -4.73) |
| Western Sub-Saharan Africa | 5173622.26 (2677641.08 to 8029348.85) | 2086.67 (1120.79 to 3154.80) | 2.48 (1.37 to 3.72) | 6967211.87 (3773030.06 to 11932167.19) | 1580.81 (892.93 to 2555.14) | 2.68 (1.51 to 4.26) | -0.07 (-0.47 to 0.33) |

Table S9. Number and age-standardised rates of deaths attributable to household particulate matter pollution in 1990 and 2021, and estimated annual percentage changes from 1990 to 2021, by region.

| **Cause of deaths** | **1990** |  |  | **2021** |  |  | **1990–2021** |
| --- | --- | --- | --- | --- | --- | --- | --- |
|  | **Number of cases** | **Age-standardised rates per 100 000 people** | **Age-standardised PAF (%)** | **Number of cases** | **Age-standardised rates per 100 000 people** | **Age-standardised PAF (%)** | **EAPC in age-standardised rates (%)** |
| **Global** | 4815870.19 (3773986.88 to 5859598.13) | 115.77 (90.88 to 140.88) | 10.46 (8.26 to 12.62) | 3112926.41 (1895168.20 to 5188696.63) | 39.05 (23.97 to 64.45) | 4.67 (2.86 to 7.63) | -3.81 (-4.13 to -3.49) |
| High SDI | 52480.75 (17098.11 to 125992.52) | 4.75 (1.55 to 11.42) | 0.69 (0.22 to 1.65) | 1783.88 (2.67 to 16949.80) | 0.08 (0.00 to 0.72) | 0.02 (0.00 to 0.15) | -14.16 (-14.70 to -13.61) |
| High-middle SDI | 744120.15 (522797.30 to 1036158.20) | 84.49 (59.28 to 118.04) | 8.68 (6.17 to 12.05) | 94324.94 (5875.00 to 508823.45) | 4.87 (0.30 to 26.34) | 0.70 (0.04 to 3.78) | -10.13 (-11.25 to -8.99) |
| Middle SDI | 1687923.70 (1339104.64 to 2025035.89) | 186.42 (151.88 to 223.38) | 16.85 (13.80 to 19.80) | 630981.79 (154774.97 to 1612423.93) | 27.23 (6.69 to 69.37) | 3.32 (0.81 to 8.48) | -6.45 (-7.13 to -5.76) |
| Low-middle SDI | 1502149.44 (1177046.92 to 1771344.96) | 206.01 (167.90 to 242.90) | 14.95 (12.21 to 17.58) | 1449665.84 (949897.93 to 2012589.04) | 111.40 (72.36 to 155.82) | 9.68 (6.32 to 13.23) | -1.98 (-2.19 to -1.78) |
| Low SDI | 826044.76 (582734.54 to 1039890.06) | 243.32 (190.77 to 288.28) | 13.37 (10.48 to 15.75) | 934006.84 (733838.26 to 1159525.98) | 170.15 (136.54 to 204.45) | 11.51 (9.15 to 13.66) | -1.16 (-1.23 to -1.09) |
| Andean Latin America | 16028.87 (7306.53 to 26756.58) | 63.95 (29.92 to 105.01) | 7.07 (3.28 to 11.62) | 4543.70 (815.87 to 14386.26) | 7.97 (1.42 to 25.28) | 0.82 (0.15 to 2.57) | -6.54 (-6.98 to -6.11) |
| Australasia | 61.54 (0.03 to 664.00) | 0.27 (0.00 to 2.98) | 0.04 (0.00 to 0.46) | 3.93 (0.00 to 25.38) | 0.01 (0.00 to 0.04) | 0.00 (0.00 to 0.01) | -12.29 (-13.01 to -11.57) |
| Caribbean | 19464.56 (13734.60 to 25393.31) | 68.47 (47.65 to 89.97) | 7.08 (5.00 to 9.24) | 17921.07 (12730.22 to 23702.55) | 35.83 (25.72 to 47.40) | 3.81 (2.93 to 4.92) | -1.92 (-2.08 to -1.76) |
| Central Asia | 41807.96 (19797.82 to 72786.92) | 86.90 (41.07 to 155.72) | 8.56 (4.03 to 15.21) | 16245.34 (6889.52 to 34850.14) | 23.19 (9.74 to 50.60) | 2.36 (0.99 to 5.12) | -5.55 (-6.60 to -4.49) |
| Central Europe | 60543.17 (15151.13 to 159408.50) | 46.14 (11.67 to 121.00) | 4.60 (1.16 to 12.06) | 8301.33 (287.25 to 53779.15) | 3.54 (0.12 to 22.90) | 0.45 (0.02 to 2.91) | -9.58 (-10.30 to -8.87) |
| Central Latin America | 32628.28 (14817.00 to 58700.86) | 34.97 (14.81 to 65.73) | 4.07 (1.72 to 7.61) | 22207.98 (7635.79 to 55032.68) | 9.46 (3.27 to 23.40) | 1.11 (0.39 to 2.71) | -4.30 (-4.40 to -4.20) |
| Central Sub-Saharan Africa | 78066.07 (53248.52 to 101972.43) | 247.47 (181.24 to 311.65) | 13.01 (9.54 to 15.89) | 90188.24 (65800.57 to 117168.92) | 171.51 (124.85 to 223.31) | 10.15 (7.74 to 12.59) | -1.35 (-1.46 to -1.24) |
| East Asia | 1821703.80 (1426075.51 to 2181059.59) | 285.67 (225.78 to 342.96) | 24.13 (19.58 to 28.16) | 478939.04 (123132.31 to 1415105.01) | 25.06 (6.38 to 74.14) | 3.89 (0.96 to 11.49) | -8.32 (-9.12 to -7.51) |
| Eastern Europe | 27264.15 (6172.48 to 105359.21) | 11.29 (2.58 to 43.33) | 1.11 (0.25 to 4.26) | 8129.69 (1189.43 to 33473.72) | 2.30 (0.34 to 9.44) | 0.22 (0.03 to 0.91) | -7.47 (-9.11 to -5.81) |
| Eastern Sub-Saharan Africa | 301142.19 (196123.22 to 391710.52) | 240.40 (173.90 to 293.43) | 11.97 (8.69 to 14.52) | 298305.58 (232086.92 to 365828.26) | 154.58 (122.17 to 187.61) | 9.60 (7.67 to 11.42) | -1.62 (-1.69 to -1.54) |
| High-income Asia Pacific | 979.29 (70.12 to 5682.88) | 0.54 (0.04 to 3.15) | 0.09 (0.01 to 0.53) | 35.16 (0.00 to 264.12) | 0.01 (0.00 to 0.04) | 0.00 (0.00 to 0.01) | -13.35 (-14.46 to -12.23) |
| High-income North America | 105.08 (0.01 to 849.44) | 0.03 (0.00 to 0.24) | 0.00 (0.00 to 0.04) | 17.57 (0.00 to 104.62) | 0.00 (0.00 to 0.02) | 0.00 (0.00 to 0.00) | -8.46 (-8.81 to -8.11) |
| North Africa and Middle East | 130615.03 (83639.03 to 199545.30) | 68.12 (43.42 to 108.29) | 5.83 (3.75 to 9.27) | 59463.03 (41413.73 to 82839.82) | 13.73 (9.53 to 19.48) | 1.38 (0.98 to 1.91) | -5.55 (-5.73 to -5.37) |
| Oceania | 8813.68 (6387.72 to 11319.02) | 297.16 (226.72 to 371.19) | 21.21 (17.04 to 24.81) | 15273.42 (11419.81 to 19202.01) | 219.79 (163.96 to 273.33) | 15.84 (11.82 to 20.22) | -0.91 (-0.97 to -0.85) |
| South Asia | 1453365.96 (1143384.25 to 1716293.41) | 216.12 (175.14 to 252.39) | 15.59 (12.47 to 18.40) | 1419263.83 (931276.99 to 2022026.07) | 108.87 (71.25 to 155.82) | 9.97 (6.59 to 14.03) | -2.16 (-2.38 to -1.94) |
| Southeast Asia | 459014.94 (356797.59 to 556884.40) | 180.70 (141.74 to 217.32) | 15.76 (12.52 to 18.76) | 308987.94 (143963.61 to 543732.10) | 55.21 (25.53 to 97.11) | 5.87 (2.61 to 10.31) | -3.72 (-4.17 to -3.28) |
| Southern Latin America | 8693.22 (2278.82 to 21918.08) | 20.34 (5.33 to 51.31) | 2.49 (0.65 to 6.27) | 553.76 (2.31 to 5219.75) | 0.62 (0.00 to 5.85) | 0.10 (0.00 to 0.92) | -11.04 (-11.29 to -10.79) |
| Southern Sub-Saharan Africa | 24196.35 (14381.29 to 36278.52) | 72.03 (41.79 to 108.45) | 6.29 (3.69 to 9.41) | 23230.25 (13541.80 to 38449.25) | 41.50 (23.49 to 70.63) | 2.30 (1.33 to 3.80) | -1.79 (-2.41 to -1.16) |
| Tropical Latin America | 48372.90 (26803.34 to 79650.83) | 55.83 (30.74 to 93.51) | 6.08 (3.34 to 10.16) | 11693.46 (2772.10 to 32836.72) | 4.79 (1.15 to 13.39) | 0.64 (0.15 to 1.80) | -7.86 (-8.13 to -7.59) |
| Western Europe | 1767.67 (18.30 to 14227.68) | 0.30 (0.00 to 2.42) | 0.04 (0.00 to 0.35) | 84.88 (0.03 to 763.27) | 0.01 (0.00 to 0.07) | 0.00 (0.00 to 0.02) | -11.66 (-12.28 to -11.03) |
| Western Sub-Saharan Africa | 281235.50 (189139.19 to 375090.57) | 191.21 (136.50 to 239.59) | 11.51 (8.33 to 14.33) | 329537.20 (227817.17 to 450701.16) | 126.09 (87.72 to 169.29) | 8.85 (6.21 to 11.68) | -1.56 (-1.75 to -1.36) |

Table S10. Number and age-standardised rates of DALYs attributable to household particulate matter pollution in 1990 and 2021, and estimated annual percentage changes from 1990 to 2021, by region.

| **Cause of DALYs** | **1990** |  |  | **2021** |  |  | **1990–2021** |
| --- | --- | --- | --- | --- | --- | --- | --- |
|  | **Number of cases** | **Age-standardised rates per 100 000 people** | **Age-standardised PAF (%)** | **Number of cases** | **Age-standardised rates per 100 000 people** | **Age-standardised PAF (%)** | **EAPC in age-standardised rates (%)** |
| **Global** | 211860686.87 (154596435.41 to 265090267.09) | 4147.68 (3101.41 to 5104.55) | 8.18 (5.99 to 10.02) | 111462958.29 (75085852.45 to 163710710.53) | 1500.29 (1028.38 to 2195.56) | 4.15 (2.79 to 5.97) | -3.52 (-3.77 to -3.27) |
| High SDI | 1217186.41 (411146.42 to 2909719.50) | 117.45 (39.84 to 280.27) | 0.42 (0.14 to 1.01) | 45052.62 (696.85 to 330784.32) | 2.19 (0.03 to 16.26) | 0.01 (0.00 to 0.08) | -13.65 (-14.11 to -13.19) |
| High-middle SDI | 20056837.00 (14695860.76 to 27327756.71) | 2116.40 (1544.43 to 2892.24) | 5.66 (4.11 to 7.74) | 2085967.71 (199697.13 to 10426833.46) | 109.16 (10.32 to 548.80) | 0.42 (0.04 to 2.19) | -10.39 (-11.41 to -9.36) |
| Middle SDI | 59770450.95 (45401442.94 to 74365388.77) | 4793.87 (3753.62 to 5828.73) | 10.49 (8.20 to 12.67) | 15919175.61 (4633862.35 to 39053893.99) | 662.95 (200.96 to 1599.88) | 2.09 (0.61 to 5.05) | -6.59 (-7.18 to -5.99) |
| Low-middle SDI | 79426564.30 (57402434.91 to 98902969.91) | 7016.87 (5452.55 to 8318.77) | 10.97 (8.36 to 13.09) | 48646709.90 (32619844.84 to 66364965.19) | 3158.28 (2086.10 to 4312.30) | 7.05 (4.76 to 9.54) | -2.58 (-2.79 to -2.38) |
| Low SDI | 51264375.47 (32828205.19 to 68080092.18) | 8602.80 (6224.26 to 10680.09) | 9.80 (6.97 to 12.00) | 44684293.10 (34615726.52 to 56190685.07) | 4976.28 (3967.68 to 6147.74) | 8.60 (6.75 to 10.25) | -1.82 (-1.89 to -1.74) |
| Andean Latin America | 810003.74 (371557.49 to 1361581.06) | 2181.61 (1020.65 to 3590.15) | 4.90 (2.31 to 8.13) | 136700.60 (30141.91 to 386705.47) | 230.73 (50.96 to 651.24) | 0.65 (0.15 to 1.90) | -6.97 (-7.38 to -6.56) |
| Australasia | 1514.36 (14.01 to 13925.07) | 6.75 (0.06 to 62.43) | 0.02 (0.00 to 0.23) | 165.98 (-5.95 to 600.12) | 0.31 (-0.01 to 1.12) | 0.00 (-0.00 to 0.01) | -10.08 (-10.73 to -9.42) |
| Caribbean | 889726.29 (611779.28 to 1174153.56) | 2579.99 (1811.62 to 3386.64) | 5.70 (3.95 to 7.30) | 685490.93 (493613.08 to 904811.77) | 1501.00 (1072.26 to 1977.44) | 3.66 (2.76 to 4.68) | -1.50 (-1.70 to -1.31) |
| Central Asia | 1626693.12 (743686.98 to 2919380.79) | 2550.33 (1225.54 to 4524.72) | 5.85 (2.86 to 10.25) | 508717.41 (240303.99 to 1047363.49) | 608.79 (281.72 to 1261.43) | 1.70 (0.78 to 3.49) | -5.84 (-6.82 to -4.85) |
| Central Europe | 1420963.66 (383476.19 to 3654512.58) | 1073.89 (300.85 to 2732.83) | 2.94 (0.86 to 7.32) | 165322.74 (9328.31 to 1025511.09) | 77.68 (4.32 to 479.29) | 0.28 (0.02 to 1.75) | -9.77 (-10.48 to -9.06) |
| Central Latin America | 1542920.58 (725271.74 to 2719794.22) | 1077.97 (499.78 to 1901.57) | 2.75 (1.27 to 5.03) | 643604.81 (249052.67 to 1447672.13) | 273.49 (107.96 to 604.20) | 0.79 (0.32 to 1.77) | -4.50 (-4.59 to -4.41) |
| Central Sub-Saharan Africa | 4742971.67 (2939405.11 to 6529400.50) | 7837.36 (5515.39 to 10058.44) | 8.69 (6.00 to 11.02) | 4019095.98 (2949822.65 to 5312966.71) | 4457.75 (3329.43 to 5705.83) | 7.10 (5.52 to 8.84) | -1.96 (-2.10 to -1.82) |
| East Asia | 54588804.90 (41394134.35 to 66241873.62) | 6416.24 (4966.04 to 7747.67) | 15.04 (11.77 to 17.85) | 10329325.67 (2969091.56 to 29403511.02) | 523.32 (152.16 to 1482.68) | 2.30 (0.65 to 6.58) | -8.57 (-9.30 to -7.84) |
| Eastern Europe | 607788.10 (152299.00 to 2259632.85) | 245.51 (64.27 to 893.83) | 0.63 (0.16 to 2.34) | 160552.56 (25606.05 to 652108.69) | 47.79 (7.74 to 192.74) | 0.13 (0.02 to 0.53) | -7.59 (-9.19 to -5.97) |
| Eastern Sub-Saharan Africa | 19466220.22 (12036911.24 to 26152197.90) | 8475.13 (5699.98 to 10809.00) | 8.96 (6.05 to 11.30) | 15098087.96 (11571616.47 to 19040192.16) | 4582.29 (3649.78 to 5550.72) | 7.62 (6.09 to 8.96) | -2.13 (-2.20 to -2.07) |
| High-income Asia Pacific | 25924.69 (2201.77 to 141776.86) | 14.26 (1.16 to 76.74) | 0.06 (0.01 to 0.32) | 1135.16 (-11.31 to 5476.43) | 0.24 (-0.00 to 1.15) | 0.00 (-0.00 to 0.01) | -11.99 (-13.18 to -10.79) |
| High-income North America | 2625.38 (7.45 to 18141.68) | 0.77 (0.00 to 5.44) | 0.00 (0.00 to 0.02) | 830.35 (-33.21 to 3195.12) | 0.13 (-0.00 to 0.51) | 0.00 (-0.00 to 0.00) | -6.26 (-6.54 to -5.99) |
| North Africa and Middle East | 6393547.07 (3889565.99 to 9868167.96) | 2114.03 (1361.86 to 3191.19) | 4.15 (2.66 to 6.25) | 2455869.15 (1756122.25 to 3311268.08) | 471.88 (337.70 to 637.74) | 1.29 (0.94 to 1.75) | -5.23 (-5.44 to -5.02) |
| Oceania | 407985.63 (274854.62 to 554284.42) | 8327.83 (6168.92 to 10585.00) | 14.80 (11.25 to 17.87) | 620556.68 (454451.20 to 813617.35) | 5997.06 (4497.86 to 7526.17) | 11.59 (8.76 to 14.45) | -0.93 (-1.00 to -0.86) |
| South Asia | 77877708.05 (58288914.31 to 95423272.27) | 7501.40 (5922.40 to 8825.88) | 11.67 (9.03 to 13.77) | 47176947.32 (31869885.88 to 64917492.78) | 3213.46 (2165.35 to 4409.39) | 7.49 (5.00 to 10.27) | -2.70 (-2.91 to -2.49) |
| Southeast Asia | 19588863.13 (14206943.18 to 24861101.09) | 5382.37 (4202.90 to 6539.55) | 10.84 (8.27 to 13.15) | 9057136.67 (4433437.94 to 15355762.42) | 1457.66 (722.96 to 2455.65) | 4.14 (2.01 to 6.98) | -4.12 (-4.50 to -3.74) |
| Southern Latin America | 225498.98 (61145.21 to 550068.75) | 488.68 (132.30 to 1196.62) | 1.47 (0.41 to 3.60) | 14067.10 (370.76 to 115929.55) | 17.17 (0.44 to 141.77) | 0.07 (0.00 to 0.55) | -10.71 (-10.96 to -10.45) |
| Southern Sub-Saharan Africa | 1298667.39 (764909.64 to 1958391.01) | 2590.95 (1538.95 to 3861.04) | 4.64 (2.80 to 6.96) | 968757.81 (601345.18 to 1527694.30) | 1437.51 (896.96 to 2304.28) | 2.12 (1.29 to 3.33) | -1.83 (-2.27 to -1.39) |
| Tropical Latin America | 1977437.28 (1195855.06 to 3053208.02) | 1685.54 (981.94 to 2655.85) | 3.95 (2.32 to 6.22) | 326724.83 (89550.55 to 880411.21) | 135.76 (38.62 to 364.37) | 0.41 (0.12 to 1.05) | -8.10 (-8.39 to -7.80) |
| Western Europe | 39152.62 (1549.36 to 274066.59) | 7.20 (0.28 to 49.56) | 0.03 (0.00 to 0.19) | 3212.28 (-33.93 to 14935.03) | 0.34 (-0.00 to 1.59) | 0.00 (-0.00 to 0.01) | -9.64 (-10.27 to -9.01) |
| Western Sub-Saharan Africa | 18325670.01 (11505700.61 to 25739039.34) | 7133.87 (4801.22 to 9394.51) | 8.47 (5.72 to 11.23) | 19090656.28 (13233825.72 to 26297477.96) | 4075.18 (2891.75 to 5520.98) | 6.92 (4.92 to 9.12) | -1.98 (-2.17 to -1.80) |

Table S11. Number and age-standardised rates of deaths attributable to particulate matter pollution in 1990 and 2021, and estimated annual percentage changes from 1990 to 2021, by region.

| **Cause of deaths** | **1990** |  |  | **2021** |  |  | **1990–2021** |
| --- | --- | --- | --- | --- | --- | --- | --- |
|  | **Number of cases** | **Age-standardised rates per 100 000 people** | **Age-standardised PAF (%)** | **Number of cases** | **Age-standardised rates per 100 000 people** | **Age-standardised PAF (%)** | **EAPC in age-standardised rates (%)** |
| **Global** | 7250210.25 (6032279.35 to 8453897.77) | 182.73 (153.27 to 211.42) | 16.50 (13.88 to 18.93) | 7833220.92 (6479473.76 to 9263395.35) | 96.69 (79.91 to 114.40) | 11.57 (9.73 to 13.53) | -2.18 (-2.31 to -2.04) |
| High SDI | 628074.34 (431267.40 to 853165.61) | 57.36 (39.46 to 77.68) | 8.27 (5.70 to 11.19) | 383144.70 (282205.87 to 494136.19) | 16.66 (12.57 to 21.28) | 3.48 (2.62 to 4.42) | -4.19 (-4.30 to -4.08) |
| High-middle SDI | 1545409.01 (1270967.81 to 1828147.49) | 179.51 (146.41 to 212.58) | 18.45 (15.19 to 21.60) | 1327461.04 (1053671.97 to 1646768.22) | 69.25 (55.06 to 85.76) | 10.03 (8.13 to 12.24) | -3.44 (-3.73 to -3.15) |
| Middle SDI | 2278719.52 (1955651.99 to 2595307.11) | 251.62 (217.33 to 285.05) | 22.74 (19.88 to 25.53) | 2591253.18 (2065517.38 to 3185581.19) | 111.07 (88.84 to 136.20) | 13.56 (11.00 to 16.35) | -2.75 (-2.94 to -2.56) |
| Low-middle SDI | 1830923.63 (1483692.45 to 2119912.90) | 254.35 (215.24 to 291.93) | 18.45 (15.64 to 20.98) | 2374835.95 (2010868.53 to 2697814.85) | 182.63 (153.24 to 207.76) | 15.87 (13.46 to 17.97) | -0.98 (-1.08 to -0.88) |
| Low SDI | 960370.32 (665637.88 to 1201443.13) | 285.03 (222.46 to 333.36) | 15.66 (12.41 to 18.13) | 1150978.19 (925885.00 to 1364944.83) | 211.39 (174.87 to 245.86) | 14.30 (12.08 to 16.25) | -0.90 (-1.00 to -0.79) |
| Andean Latin America | 34813.64 (23237.77 to 44318.09) | 142.17 (100.59 to 176.39) | 15.72 (11.11 to 19.52) | 26399.52 (17789.21 to 36776.35) | 46.43 (31.23 to 64.63) | 4.77 (3.39 to 6.32) | -3.83 (-4.03 to -3.64) |
| Australasia | 3703.56 (172.32 to 9816.27) | 16.42 (0.91 to 43.43) | 2.52 (0.14 to 6.66) | 4864.07 (2915.19 to 7163.12) | 8.09 (4.90 to 11.83) | 2.19 (1.33 to 3.20) | -2.84 (-3.39 to -2.29) |
| Caribbean | 31575.40 (23144.56 to 41842.42) | 117.39 (85.01 to 158.59) | 12.13 (8.78 to 16.25) | 38805.75 (26856.86 to 51629.70) | 74.67 (52.85 to 98.56) | 7.95 (5.79 to 10.39) | -1.30 (-1.46 to -1.13) |
| Central Asia | 82636.18 (52952.85 to 115114.52) | 174.14 (114.44 to 240.33) | 17.15 (11.17 to 23.51) | 81648.68 (63892.06 to 101951.26) | 118.16 (91.88 to 147.44) | 12.05 (9.41 to 14.93) | -1.71 (-2.05 to -1.37) |
| Central Europe | 218654.89 (143960.69 to 295309.90) | 164.45 (108.31 to 222.14) | 16.41 (10.79 to 22.12) | 117889.50 (90302.71 to 155957.85) | 50.50 (38.71 to 66.60) | 6.39 (4.92 to 8.44) | -4.08 (-4.30 to -3.85) |
| Central Latin America | 90535.42 (66439.86 to 113930.93) | 102.43 (74.51 to 129.69) | 11.91 (8.67 to 15.03) | 98163.42 (71435.74 to 131147.30) | 41.47 (30.24 to 55.32) | 4.86 (3.55 to 6.42) | -3.04 (-3.18 to -2.91) |
| Central Sub-Saharan Africa | 87528.76 (60941.14 to 112049.22) | 280.83 (213.70 to 348.38) | 14.77 (11.47 to 17.65) | 108424.06 (80375.67 to 138326.16) | 206.07 (154.33 to 264.54) | 12.19 (9.63 to 14.69) | -1.10 (-1.17 to -1.04) |
| East Asia | 2285384.49 (1962765.07 to 2623330.34) | 359.56 (310.17 to 411.67) | 30.37 (26.66 to 33.68) | 2356176.77 (1849554.88 to 2981328.63) | 124.30 (97.54 to 156.01) | 19.35 (15.90 to 23.41) | -3.57 (-3.86 to -3.27) |
| Eastern Europe | 365690.84 (194915.80 to 528228.09) | 149.27 (80.08 to 215.27) | 14.70 (7.88 to 21.17) | 172944.91 (111030.11 to 252061.23) | 48.81 (31.49 to 71.01) | 4.77 (3.06 to 7.01) | -4.43 (-4.95 to -3.91) |
| Eastern Sub-Saharan Africa | 323652.12 (212928.75 to 414975.15) | 259.82 (189.52 to 317.23) | 12.94 (9.49 to 15.62) | 329544.59 (258402.86 to 400431.54) | 171.82 (136.68 to 205.19) | 10.67 (8.53 to 12.47) | -1.47 (-1.54 to -1.41) |
| High-income Asia Pacific | 58681.32 (17654.74 to 112925.76) | 32.88 (9.92 to 63.66) | 5.55 (1.68 to 10.73) | 76410.49 (44566.92 to 110398.29) | 12.94 (7.71 to 18.28) | 4.03 (2.40 to 5.69) | -3.10 (-3.39 to -2.81) |
| High-income North America | 144582.42 (61240.66 to 244160.52) | 40.22 (17.22 to 67.68) | 6.02 (2.58 to 10.14) | 53353.50 (25905.12 to 84460.77) | 7.75 (3.84 to 12.17) | 1.32 (0.66 to 2.08) | -5.77 (-6.23 to -5.32) |
| North Africa and Middle East | 333734.59 (271290.96 to 398620.30) | 187.38 (154.50 to 220.36) | 16.03 (13.41 to 18.66) | 453957.25 (370871.84 to 538107.84) | 116.25 (95.01 to 136.53) | 11.73 (9.85 to 13.55) | -1.49 (-1.55 to -1.43) |
| Oceania | 9678.37 (7510.11 to 12197.32) | 327.77 (261.38 to 403.92) | 23.39 (19.78 to 26.90) | 17664.43 (13519.93 to 21816.89) | 255.72 (197.08 to 314.71) | 18.43 (14.05 to 22.63) | -0.76 (-0.80 to -0.72) |
| South Asia | 1748163.56 (1429077.74 to 2010951.69) | 262.18 (220.22 to 300.55) | 18.91 (16.04 to 21.46) | 2525171.15 (2136169.76 to 2868314.64) | 193.96 (163.84 to 221.42) | 17.76 (15.19 to 19.99) | -0.86 (-0.99 to -0.72) |
| Southeast Asia | 566753.97 (458378.43 to 664456.52) | 224.28 (185.24 to 258.69) | 19.56 (16.12 to 22.52) | 641348.99 (488922.16 to 806636.23) | 114.17 (87.09 to 143.34) | 12.14 (9.26 to 15.11) | -2.24 (-2.48 to -2.00) |
| Southern Latin America | 32663.38 (19060.92 to 48070.92) | 76.05 (44.22 to 112.26) | 9.30 (5.41 to 13.75) | 23533.73 (13000.46 to 34909.24) | 26.47 (14.88 to 38.92) | 4.17 (2.36 to 6.15) | -3.17 (-3.40 to -2.95) |
| Southern Sub-Saharan Africa | 40496.49 (29586.97 to 51777.97) | 124.77 (92.70 to 157.35) | 10.91 (8.27 to 13.73) | 54295.11 (40445.33 to 70357.05) | 101.85 (76.09 to 130.25) | 5.66 (4.26 to 7.14) | -0.46 (-0.93 to 0.01) |
| Tropical Latin America | 87804.20 (55664.85 to 122187.38) | 101.71 (63.36 to 143.71) | 11.07 (6.87 to 15.60) | 65894.44 (38440.55 to 93250.84) | 26.78 (15.70 to 37.87) | 3.59 (2.11 to 5.07) | -4.26 (-4.38 to -4.13) |
| Western Europe | 340096.11 (175253.74 to 536657.21) | 57.72 (30.00 to 90.78) | 8.48 (4.40 to 13.33) | 128675.35 (90699.30 to 171347.71) | 11.48 (8.21 to 15.14) | 2.67 (1.91 to 3.53) | -5.31 (-5.52 to -5.11) |
| Western Sub-Saharan Africa | 363380.54 (249741.07 to 464513.65) | 249.78 (191.81 to 299.71) | 15.03 (11.58 to 17.79) | 458055.19 (354251.94 to 566006.02) | 179.43 (143.01 to 214.86) | 12.60 (10.10 to 14.86) | -1.03 (-1.13 to -0.93) |

Table S12. Number and age-standardised rates of DALYs attributable to particulate matter pollution in 1990 and 2021, and estimated annual percentage changes from 1990 to 2021, by region.

| **Cause of DALYs** | **1990** |  |  | **2021** |  |  | **1990–2021** |
| --- | --- | --- | --- | --- | --- | --- | --- |
|  | **Number of cases** | **Age-standardised rates per 100 000 people** | **Age-standardised PAF (%)** | **Number of cases** | **Age-standardised rates per 100 000 people** | **Age-standardised PAF (%)** | **EAPC in age-standardised rates (%)** |
| **Global** | 289354268.77 (222295153.05 to 350589635.43) | 5865.15 (4690.12 to 6998.30) | 11.56 (9.14 to 13.63) | 231511232.94 (194538892.20 to 270855451.37) | 2984.47 (2489.63 to 3487.35) | 8.25 (6.85 to 9.69) | -2.27 (-2.39 to -2.15) |
| High SDI | 13352746.38 (9438088.29 to 17610834.56) | 1275.07 (910.16 to 1668.60) | 4.57 (3.20 to 6.02) | 8236387.24 (6239929.47 to 10398102.85) | 431.38 (334.22 to 537.24) | 1.91 (1.47 to 2.43) | -3.66 (-3.73 to -3.58) |
| High-middle SDI | 39562456.38 (32894449.17 to 46737495.99) | 4204.32 (3481.76 to 4975.45) | 11.24 (9.23 to 13.42) | 27643307.37 (22477546.99 to 34211203.46) | 1481.88 (1216.13 to 1815.40) | 5.76 (4.61 to 7.08) | -3.73 (-3.98 to -3.48) |
| Middle SDI | 81484628.17 (64637634.23 to 97016242.35) | 6498.81 (5455.31 to 7466.23) | 14.22 (11.77 to 16.35) | 63574457.14 (52026702.43 to 76406830.82) | 2605.44 (2142.77 to 3121.59) | 8.20 (6.66 to 9.89) | -3.03 (-3.16 to -2.89) |
| Low-middle SDI | 95426824.22 (70303340.48 to 115552090.34) | 8510.87 (6868.02 to 9918.36) | 13.30 (10.63 to 15.38) | 77433511.60 (64788177.89 to 90113244.67) | 5046.41 (4254.45 to 5819.22) | 11.27 (9.56 to 12.85) | -1.61 (-1.70 to -1.53) |
| Low SDI | 59313476.34 (38470947.32 to 78007375.56) | 10003.12 (7172.85 to 12394.84) | 11.39 (8.15 to 13.85) | 54461665.61 (42615604.99 to 66989287.19) | 6114.26 (5006.33 to 7220.39) | 10.56 (8.69 to 12.03) | -1.56 (-1.63 to -1.48) |
| Andean Latin America | 1688478.37 (1023509.00 to 2231530.31) | 4626.50 (3122.62 to 5917.55) | 10.40 (6.88 to 13.24) | 719629.46 (510431.95 to 984296.83) | 1211.71 (860.75 to 1656.88) | 3.42 (2.47 to 4.50) | -4.43 (-4.60 to -4.26) |
| Australasia | 77233.13 (7640.19 to 199095.60) | 342.98 (37.92 to 864.90) | 1.26 (0.14 to 3.21) | 94657.64 (57315.07 to 136290.19) | 183.52 (112.59 to 260.79) | 0.94 (0.56 to 1.36) | -2.49 (-3.05 to -1.91) |
| Caribbean | 1239259.08 (905973.50 to 1588008.03) | 3778.94 (2799.27 to 4913.68) | 8.34 (6.17 to 10.78) | 1222689.59 (907210.44 to 1561367.64) | 2553.18 (1880.54 to 3267.52) | 6.23 (4.78 to 7.80) | -1.03 (-1.21 to -0.86) |
| Central Asia | 3073798.91 (1918945.98 to 4452405.12) | 4940.86 (3170.90 to 7055.75) | 11.33 (7.19 to 15.95) | 2294667.91 (1811714.28 to 2839442.67) | 2809.87 (2236.53 to 3473.88) | 7.86 (6.27 to 9.77) | -2.28 (-2.62 to -1.94) |
| Central Europe | 4999733.88 (3371052.15 to 6663008.03) | 3682.52 (2523.41 to 4896.16) | 10.09 (6.84 to 13.50) | 2308103.56 (1821968.26 to 2981049.46) | 1087.55 (859.68 to 1391.42) | 3.86 (3.05 to 4.99) | -4.20 (-4.41 to -4.00) |
| Central Latin America | 3896556.39 (2838224.43 to 4964443.60) | 2910.86 (2181.98 to 3666.57) | 7.43 (5.45 to 9.35) | 2668776.13 (2000673.51 to 3493410.47) | 1108.17 (835.61 to 1445.30) | 3.20 (2.42 to 4.09) | -3.21 (-3.33 to -3.10) |
| Central Sub-Saharan Africa | 5282837.78 (3349086.78 to 7147050.96) | 8817.68 (6304.42 to 11118.22) | 9.78 (6.98 to 12.24) | 4823124.85 (3607614.71 to 6286531.12) | 5363.23 (4051.65 to 6767.83) | 8.54 (6.78 to 10.38) | -1.68 (-1.77 to -1.59) |
| East Asia | 67708666.56 (54601402.09 to 79052662.71) | 7993.20 (6673.06 to 9261.57) | 18.74 (15.61 to 21.37) | 48595899.06 (38257555.42 to 61689086.45) | 2443.38 (1936.26 to 3055.29) | 10.74 (8.77 to 13.04) | -4.00 (-4.23 to -3.78) |
| Eastern Europe | 8004362.28 (4402863.13 to 11534228.10) | 3140.55 (1772.34 to 4451.34) | 8.08 (4.56 to 11.67) | 3443922.54 (2252038.41 to 4922809.80) | 1022.19 (678.31 to 1445.77) | 2.78 (1.81 to 3.98) | -4.48 (-5.02 to -3.94) |
| Eastern Sub-Saharan Africa | 20845677.47 (13081104.60 to 27884952.61) | 9119.45 (6197.63 to 11483.83) | 9.64 (6.50 to 12.10) | 16542897.87 (12848721.97 to 20551011.92) | 5060.24 (4014.19 to 6051.27) | 8.41 (6.79 to 9.77) | -2.00 (-2.06 to -1.94) |
| High-income Asia Pacific | 1355826.07 (449709.93 to 2505407.85) | 728.05 (250.24 to 1334.86) | 3.05 (1.06 to 5.74) | 1575140.07 (990802.85 to 2195045.03) | 362.93 (239.33 to 492.07) | 2.21 (1.43 to 3.09) | -2.37 (-2.67 to -2.08) |
| High-income North America | 3074792.49 (1346333.35 to 5077147.94) | 909.77 (409.68 to 1482.70) | 3.18 (1.39 to 5.25) | 1273854.77 (639499.08 to 2032258.90) | 212.05 (111.00 to 329.63) | 0.76 (0.41 to 1.18) | -5.10 (-5.48 to -4.71) |
| North Africa and Middle East | 14994360.35 (11287109.84 to 19027794.62) | 5333.31 (4339.59 to 6381.14) | 10.47 (8.59 to 12.37) | 13607276.20 (11124473.06 to 15977676.37) | 2871.13 (2365.27 to 3385.30) | 7.83 (6.49 to 9.11) | -1.96 (-2.03 to -1.90) |
| Oceania | 445496.07 (299429.43 to 584768.15) | 9143.94 (7219.39 to 11459.07) | 16.25 (12.93 to 19.30) | 709540.19 (552344.81 to 894491.83) | 6922.92 (5336.57 to 8527.45) | 13.38 (10.61 to 16.12) | -0.79 (-0.84 to -0.74) |
| South Asia | 92551626.97 (69742244.27 to 110668771.62) | 8984.51 (7294.60 to 10413.94) | 13.97 (11.20 to 15.94) | 80560949.39 (68745929.85 to 92313537.07) | 5480.42 (4694.75 to 6270.19) | 12.77 (10.91 to 14.41) | -1.50 (-1.60 to -1.41) |
| Southeast Asia | 23830819.56 (18122401.43 to 29258275.43) | 6610.05 (5336.42 to 7768.10) | 13.31 (10.60 to 15.47) | 18304669.10 (14425216.99 to 22601241.48) | 2916.48 (2305.56 to 3588.58) | 8.29 (6.49 to 10.13) | -2.70 (-2.89 to -2.51) |
| Southern Latin America | 843027.27 (523518.57 to 1204220.11) | 1824.43 (1126.90 to 2618.41) | 5.49 (3.30 to 7.77) | 530293.78 (325881.54 to 754529.69) | 650.33 (408.66 to 910.17) | 2.47 (1.55 to 3.48) | -3.23 (-3.45 to -3.01) |
| Southern Sub-Saharan Africa | 2070545.69 (1393212.95 to 2701828.93) | 4287.29 (3102.77 to 5444.93) | 7.69 (5.53 to 9.78) | 2033153.21 (1520703.05 to 2653978.00) | 3124.52 (2372.35 to 4045.47) | 4.61 (3.53 to 5.80) | -0.78 (-1.14 to -0.41) |
| Tropical Latin America | 3371278.33 (2288627.47 to 4568220.20) | 2948.61 (1956.88 to 4014.72) | 6.92 (4.55 to 9.43) | 1756467.51 (1070407.03 to 2434815.35) | 716.68 (444.88 to 981.23) | 2.17 (1.35 to 2.99) | -4.58 (-4.71 to -4.44) |
| Western Europe | 6496052.71 (3404530.21 to 9980466.19) | 1175.74 (633.44 to 1784.53) | 4.40 (2.31 to 6.63) | 2381794.96 (1701432.01 to 3136163.68) | 265.98 (193.43 to 348.72) | 1.33 (0.97 to 1.76) | -4.91 (-5.08 to -4.75) |
| Western Sub-Saharan Africa | 23503839.40 (14922778.65 to 31485893.60) | 9222.06 (6425.41 to 11726.77) | 10.96 (7.48 to 13.75) | 26063725.14 (19465252.67 to 33205370.12) | 5657.04 (4437.86 to 6939.28) | 9.60 (7.52 to 11.47) | -1.50 (-1.62 to -1.39) |

Table S13. Number and age-standardised rates of DALYs attributable to ambient particulate matter pollution in 1990 and 2021, and estimated annual percentage changes from 1990 to 2021, by country.

| **Cause of DALYs** | **1990** |  |  | **2021** |  |  | **1990–2021** |
| --- | --- | --- | --- | --- | --- | --- | --- |
|  | **Number of cases** | **Age-standardised rates per 100 000 people** | **Age-standardised PAF (%)** | **Number of cases** | **Age-standardised rates per 100 000 people** | **Age-standardised PAF (%)** | **EAPC in age-standardised rates (%)** |
| Afghanistan | 225544.90 (94767.13 to 406875.67) | 2026.71 (913.24 to 3754.86) | 2.22 (1.00 to 4.11) | 197477.25 (123733.30 to 305788.99) | 1115.26 (695.69 to 1705.94) | 1.51 (0.96 to 2.22) | -1.57 (-2.03 to -1.09) |
| Albania | 25303.90 (10609.47 to 49283.37) | 1046.28 (446.58 to 1989.60) | 3.09 (1.32 to 6.05) | 35799.27 (20730.78 to 47866.52) | 931.16 (511.30 to 1254.00) | 3.50 (1.98 to 4.80) | 0.87 (0.47 to 1.28) |
| Algeria | 421781.09 (282107.15 to 566449.14) | 2496.88 (1769.94 to 3218.31) | 6.02 (4.30 to 7.88) | 584254.44 (425139.61 to 754452.38) | 1737.07 (1260.41 to 2261.11) | 5.39 (3.91 to 6.87) | -1.33 (-1.54 to -1.12) |
| American Samoa | 174.68 (15.76 to 494.32) | 567.86 (20.92 to 1747.24) | 1.56 (0.06 to 4.96) | 290.47 (44.03 to 603.98) | 612.18 (99.52 to 1260.06) | 1.71 (0.28 to 3.44) | -0.05 (-0.31 to 0.21) |
| Andorra | 373.99 (163.22 to 633.65) | 698.22 (317.91 to 1163.81) | 2.99 (1.33 to 4.90) | 264.72 (144.76 to 410.28) | 171.51 (93.31 to 265.64) | 0.91 (0.52 to 1.37) | -4.27 (-4.59 to -3.95) |
| Angola | 157428.16 (70562.13 to 280740.31) | 1351.60 (658.36 to 2357.03) | 1.32 (0.65 to 2.26) | 398844.75 (170497.66 to 685276.12) | 1804.92 (817.81 to 2936.39) | 2.89 (1.30 to 4.59) | 1.75 (1.40 to 2.11) |
| Antigua and Barbuda | 752.92 (220.55 to 1438.91) | 1384.97 (401.98 to 2647.16) | 4.58 (1.32 to 8.63) | 1010.16 (467.72 to 1599.81) | 1022.80 (488.66 to 1605.71) | 3.60 (1.77 to 5.69) | -1.13 (-1.32 to -0.93) |
| Argentina | 478183.99 (252314.45 to 769199.97) | 1499.22 (780.07 to 2412.92) | 4.43 (2.32 to 7.06) | 339069.29 (187834.76 to 511400.57) | 646.63 (365.81 to 956.09) | 2.37 (1.34 to 3.56) | -2.71 (-2.94 to -2.48) |
| Armenia | 71443.12 (26731.99 to 126595.31) | 2479.88 (910.30 to 4396.29) | 6.65 (2.57 to 11.70) | 81732.20 (57645.57 to 106881.10) | 2045.57 (1464.13 to 2658.48) | 7.35 (5.39 to 9.49) | 0.25 (-0.28 to 0.79) |
| Australia | 63044.92 (5986.74 to 162912.42) | 336.55 (37.32 to 847.40) | 1.26 (0.14 to 3.16) | 82134.70 (51497.62 to 117798.55) | 188.11 (117.30 to 264.72) | 0.97 (0.60 to 1.40) | -2.36 (-2.95 to -1.76) |
| Austria | 148699.39 (81921.68 to 222042.93) | 1305.19 (727.19 to 1913.16) | 4.79 (2.73 to 7.04) | 52794.76 (38330.12 to 68218.22) | 292.75 (215.15 to 376.57) | 1.48 (1.09 to 1.95) | -5.01 (-5.18 to -4.84) |
| Azerbaijan | 186555.49 (56791.19 to 380804.64) | 2844.46 (876.99 to 5669.50) | 6.10 (1.94 to 12.02) | 203459.66 (121462.25 to 297408.25) | 2314.55 (1394.53 to 3331.36) | 6.23 (3.79 to 8.94) | 0.67 (-0.13 to 1.48) |
| Bahamas | 2718.23 (823.20 to 4704.13) | 1511.83 (403.94 to 2693.54) | 4.18 (1.11 to 7.52) | 3816.77 (1462.56 to 6581.99) | 981.18 (391.80 to 1682.98) | 2.56 (1.06 to 4.25) | -1.33 (-1.48 to -1.18) |
| Bahrain | 13146.66 (10821.71 to 15275.16) | 6452.66 (5272.55 to 7459.05) | 15.63 (12.89 to 17.97) | 23804.58 (19075.65 to 28762.15) | 2936.52 (2397.90 to 3489.90) | 9.27 (7.63 to 10.87) | -2.96 (-3.26 to -2.66) |
| Bangladesh | 1252563.32 (571344.21 to 2284450.54) | 1133.93 (562.43 to 2028.49) | 1.64 (0.81 to 2.94) | 1188379.23 (635858.33 to 2029269.21) | 879.49 (469.26 to 1492.67) | 2.52 (1.37 to 4.15) | -0.77 (-1.17 to -0.37) |
| Barbados | 4484.72 (1705.98 to 7654.70) | 1646.34 (702.39 to 2775.81) | 5.35 (2.20 to 8.81) | 5278.04 (2703.13 to 8024.66) | 1180.96 (653.65 to 1777.43) | 4.31 (2.40 to 6.22) | -1.05 (-1.22 to -0.87) |
| Belarus | 386280.43 (215399.12 to 556704.47) | 3179.48 (1791.71 to 4548.87) | 9.00 (5.17 to 13.03) | 216454.90 (149943.11 to 293677.65) | 1381.27 (961.10 to 1867.11) | 3.93 (2.86 to 5.29) | -3.41 (-3.86 to -2.96) |
| Belgium | 196677.71 (106272.99 to 297639.64) | 1340.87 (731.87 to 2011.48) | 4.99 (2.67 to 7.50) | 64553.44 (46673.25 to 84637.94) | 290.77 (207.89 to 376.71) | 1.43 (1.05 to 1.90) | -4.92 (-5.18 to -4.65) |
| Belize | 2055.37 (669.63 to 4013.62) | 1310.82 (396.84 to 2585.70) | 3.83 (1.18 to 7.62) | 3920.59 (1688.43 to 6282.00) | 1239.76 (526.24 to 1999.04) | 3.82 (1.66 to 6.11) | -0.53 (-0.94 to -0.11) |
| Benin | 96701.86 (44380.51 to 166455.42) | 1584.78 (815.34 to 2631.80) | 2.00 (1.04 to 3.31) | 114857.18 (60806.47 to 198001.61) | 972.57 (545.25 to 1674.39) | 1.72 (0.97 to 2.89) | -0.83 (-1.27 to -0.39) |
| Bermuda | 374.58 (12.24 to 947.28) | 627.69 (28.68 to 1570.34) | 2.07 (0.10 to 5.19) | 220.58 (46.11 to 414.22) | 173.38 (39.36 to 320.37) | 0.79 (0.17 to 1.42) | -4.71 (-5.10 to -4.33) |
| Bhutan | 6139.14 (2812.54 to 11403.25) | 1043.11 (509.49 to 1912.63) | 1.60 (0.78 to 2.86) | 11835.35 (7038.41 to 16103.97) | 1981.90 (1186.37 to 2674.09) | 6.16 (3.64 to 8.12) | 2.68 (2.32 to 3.04) |
| Bolivia (Plurinational State of) | 205601.46 (75600.12 to 365582.76) | 3213.02 (1267.38 to 5558.24) | 5.25 (2.16 to 9.16) | 113538.14 (57385.80 to 181570.40) | 1205.61 (600.10 to 1922.45) | 2.52 (1.32 to 3.94) | -3.53 (-3.81 to -3.26) |
| Bosnia and Herzegovina | 29696.53 (15587.25 to 51376.42) | 766.29 (404.48 to 1329.60) | 2.46 (1.32 to 4.31) | 85209.53 (43429.68 to 112086.79) | 1433.66 (722.20 to 1885.79) | 5.26 (2.73 to 6.77) | 2.69 (2.02 to 3.36) |
| Botswana | 15266.05 (5872.92 to 28075.00) | 1512.47 (598.86 to 2758.45) | 2.52 (1.03 to 4.46) | 31173.45 (15068.97 to 46626.47) | 1800.45 (910.42 to 2631.94) | 2.69 (1.34 to 3.88) | 0.70 (0.14 to 1.27) |
| Brazil | 1374152.23 (539167.46 to 2359836.12) | 1282.39 (472.38 to 2260.52) | 2.99 (1.11 to 5.38) | 1396905.41 (828751.95 to 2016702.29) | 581.53 (356.77 to 833.51) | 1.76 (1.03 to 2.49) | -2.61 (-2.81 to -2.42) |
| Brunei Darussalam | 805.56 (123.49 to 1842.78) | 675.45 (67.85 to 1609.24) | 1.98 (0.20 to 4.84) | 1431.09 (348.74 to 2669.94) | 407.91 (103.57 to 749.90) | 1.61 (0.42 to 3.06) | -1.27 (-1.99 to -0.55) |
| Bulgaria | 352552.18 (187532.00 to 532300.40) | 3417.96 (1783.13 to 5076.41) | 8.81 (4.45 to 13.18) | 197019.60 (151772.25 to 252040.70) | 1506.04 (1157.69 to 1929.79) | 3.98 (3.06 to 5.07) | -2.86 (-3.10 to -2.62) |
| Burkina Faso | 169878.51 (76088.60 to 288411.79) | 1314.70 (650.56 to 2161.25) | 1.37 (0.67 to 2.26) | 201262.94 (105145.84 to 329796.82) | 916.49 (498.04 to 1502.81) | 1.42 (0.79 to 2.29) | -0.26 (-0.58 to 0.07) |
| Burundi | 47860.23 (21358.36 to 89338.35) | 871.34 (424.66 to 1606.08) | 0.85 (0.42 to 1.55) | 35641.02 (18101.74 to 62483.24) | 394.20 (201.83 to 681.08) | 0.68 (0.36 to 1.15) | -2.84 (-3.04 to -2.64) |
| Cabo Verde | 2609.36 (1427.51 to 3916.56) | 783.30 (452.36 to 1170.74) | 1.89 (1.08 to 2.83) | 8768.17 (5405.35 to 12748.25) | 1994.60 (1227.39 to 2874.74) | 6.29 (4.02 to 8.76) | 3.20 (2.64 to 3.76) |
| Cambodia | 116179.14 (40575.58 to 244012.23) | 1027.41 (364.43 to 2096.29) | 1.42 (0.51 to 2.89) | 106000.23 (51651.67 to 201867.64) | 824.67 (406.81 to 1551.34) | 1.97 (1.00 to 3.53) | -0.79 (-1.07 to -0.51) |
| Cameroon | 177415.41 (79751.75 to 310548.03) | 1552.39 (736.88 to 2671.62) | 2.20 (1.05 to 3.77) | 271502.06 (137696.55 to 482168.79) | 1212.70 (635.40 to 2125.98) | 1.95 (1.01 to 3.47) | -0.07 (-0.35 to 0.22) |
| Canada | 170192.03 (52062.12 to 308905.38) | 542.24 (173.48 to 976.34) | 2.17 (0.70 to 4.01) | 71649.81 (28305.71 to 127699.05) | 109.47 (42.35 to 188.27) | 0.53 (0.20 to 0.91) | -5.42 (-5.78 to -5.05) |
| Central African Republic | 33968.07 (16403.11 to 59594.56) | 1164.68 (601.09 to 2001.98) | 1.04 (0.54 to 1.76) | 34677.68 (17730.31 to 55285.52) | 830.46 (450.82 to 1319.14) | 0.85 (0.47 to 1.32) | -0.89 (-1.04 to -0.73) |
| Chad | 112448.68 (53800.37 to 189807.91) | 1344.83 (685.22 to 2244.47) | 1.53 (0.80 to 2.56) | 242715.65 (113878.73 to 427290.03) | 1332.60 (700.35 to 2244.37) | 1.89 (1.03 to 3.05) | 0.56 (0.27 to 0.85) |
| Chile | 103077.57 (36038.63 to 192406.17) | 990.30 (346.85 to 1880.98) | 3.09 (1.08 to 5.79) | 155822.46 (105320.99 to 202629.68) | 649.56 (452.20 to 834.73) | 2.71 (1.86 to 3.49) | -1.45 (-1.70 to -1.20) |
| China | 12726189.72 (6156141.06 to 22700337.42) | 1590.95 (768.20 to 2818.49) | 3.70 (1.77 to 6.48) | 37805874.42 (26280461.19 to 46518700.41) | 1970.10 (1373.00 to 2423.21) | 8.69 (6.14 to 10.49) | 1.19 (0.85 to 1.54) |
| Colombia | 385125.77 (164121.22 to 630192.33) | 1576.81 (712.17 to 2544.86) | 4.24 (1.90 to 6.76) | 308205.60 (201048.95 to 419529.00) | 587.02 (384.56 to 791.01) | 2.11 (1.40 to 2.77) | -3.42 (-3.59 to -3.26) |
| Comoros | 2301.08 (1091.62 to 3970.05) | 446.10 (228.29 to 782.91) | 0.64 (0.31 to 1.10) | 1535.73 (775.48 to 2762.63) | 258.38 (133.95 to 463.06) | 0.55 (0.29 to 1.00) | -1.63 (-1.81 to -1.46) |
| Congo | 24835.98 (9804.41 to 52636.82) | 1409.53 (581.90 to 2799.78) | 1.70 (0.69 to 3.45) | 55139.66 (22986.93 to 94714.00) | 1706.42 (708.63 to 2932.08) | 2.84 (1.23 to 4.67) | 0.94 (0.69 to 1.20) |
| Cook Islands | 104.17 (18.08 to 248.44) | 744.08 (105.04 to 1826.70) | 1.86 (0.27 to 4.60) | 95.62 (0.90 to 217.38) | 395.89 (8.25 to 893.23) | 1.49 (0.03 to 3.40) | -2.47 (-2.88 to -2.07) |
| Costa Rica | 19838.96 (8007.28 to 33375.55) | 920.77 (385.53 to 1509.97) | 3.47 (1.49 to 5.82) | 25064.99 (16854.43 to 34035.12) | 484.24 (322.07 to 653.01) | 1.85 (1.29 to 2.54) | -2.18 (-2.47 to -1.89) |
| Côte d'Ivoire | 269103.28 (128820.10 to 443114.99) | 2059.71 (1015.78 to 3368.19) | 2.59 (1.27 to 4.25) | 340682.31 (160635.25 to 583561.50) | 1546.62 (750.29 to 2617.15) | 2.73 (1.33 to 4.53) | -0.28 (-0.69 to 0.14) |
| Croatia | 126755.55 (68618.79 to 189809.03) | 2308.11 (1244.34 to 3428.95) | 6.94 (3.83 to 10.33) | 67472.72 (52082.68 to 85347.05) | 773.64 (595.45 to 972.70) | 3.19 (2.44 to 4.08) | -3.42 (-3.68 to -3.17) |
| Cuba | 144074.21 (53788.25 to 269655.04) | 1450.58 (548.61 to 2702.48) | 4.99 (1.82 to 9.25) | 189566.99 (96961.41 to 299179.63) | 1001.60 (520.35 to 1573.88) | 3.42 (1.79 to 5.44) | -1.07 (-1.37 to -0.77) |
| Cyprus | 11641.11 (5873.31 to 17812.31) | 1811.54 (909.62 to 2795.59) | 5.67 (2.92 to 8.56) | 10048.76 (7456.59 to 12947.29) | 535.55 (403.54 to 687.87) | 2.70 (2.03 to 3.43) | -4.31 (-4.51 to -4.12) |
| Czechia | 358705.09 (199370.81 to 525804.31) | 2738.41 (1559.71 to 3969.22) | 8.01 (4.60 to 11.75) | 138305.51 (106190.97 to 175381.95) | 655.81 (504.78 to 833.45) | 2.73 (2.08 to 3.47) | -4.24 (-4.48 to -4.00) |
| Democratic People's Republic of Korea | 199283.42 (84020.74 to 383641.44) | 1217.19 (512.12 to 2291.01) | 3.27 (1.45 to 6.18) | 208594.87 (132703.95 to 310554.65) | 664.87 (422.52 to 984.66) | 2.25 (1.45 to 3.27) | -1.45 (-1.72 to -1.17) |
| Democratic Republic of the Congo | 289263.95 (136062.26 to 537888.00) | 748.49 (380.30 to 1320.27) | 0.87 (0.44 to 1.52) | 262019.05 (156118.58 to 399603.04) | 472.35 (275.86 to 735.22) | 0.77 (0.47 to 1.22) | -1.23 (-1.43 to -1.03) |
| Denmark | 103987.37 (51117.49 to 168319.82) | 1325.41 (665.83 to 2120.26) | 4.68 (2.28 to 7.24) | 27932.68 (17533.76 to 38735.40) | 245.06 (156.89 to 338.52) | 1.24 (0.79 to 1.77) | -5.71 (-5.93 to -5.48) |
| Djibouti | 6433.06 (2840.12 to 11197.63) | 1850.70 (869.19 to 3092.44) | 3.04 (1.42 to 5.05) | 16210.97 (8698.47 to 26823.51) | 1977.25 (1049.03 to 3231.23) | 3.82 (2.09 to 6.03) | 0.44 (0.11 to 0.77) |
| Dominica | 690.16 (182.86 to 1460.87) | 1094.04 (288.21 to 2312.87) | 3.27 (0.84 to 6.93) | 1058.63 (477.11 to 1695.83) | 1496.10 (694.37 to 2296.44) | 3.92 (1.87 to 6.03) | 1.02 (0.78 to 1.25) |
| Dominican Republic | 57285.73 (15101.03 to 130621.71) | 926.72 (249.60 to 2068.73) | 2.36 (0.65 to 5.20) | 153542.21 (73549.18 to 240236.69) | 1510.95 (721.38 to 2372.51) | 4.63 (2.34 to 7.17) | 2.09 (1.82 to 2.37) |
| Ecuador | 164257.81 (82234.56 to 257462.38) | 2017.76 (993.60 to 3091.82) | 5.54 (2.67 to 8.62) | 128769.99 (79844.29 to 185668.32) | 804.05 (498.63 to 1152.72) | 2.60 (1.66 to 3.66) | -3.32 (-3.84 to -2.80) |
| Egypt | 2524439.59 (1572060.89 to 3508582.71) | 5837.72 (3897.01 to 7764.85) | 10.07 (6.88 to 13.55) | 3308557.17 (2581750.34 to 4154824.44) | 5279.18 (4149.30 to 6591.94) | 12.39 (9.86 to 14.96) | -0.12 (-0.39 to 0.15) |
| El Salvador | 39429.29 (16433.99 to 76462.57) | 856.84 (352.28 to 1620.45) | 1.98 (0.82 to 3.74) | 50411.40 (25785.68 to 74956.45) | 809.31 (414.20 to 1202.97) | 2.41 (1.28 to 3.58) | -0.08 (-0.34 to 0.19) |
| Equatorial Guinea | 5679.56 (2424.15 to 10352.61) | 1281.56 (602.88 to 2239.93) | 1.34 (0.63 to 2.30) | 22207.87 (12237.44 to 34714.27) | 2802.59 (1638.78 to 4273.76) | 4.61 (2.80 to 6.55) | 3.40 (2.54 to 4.27) |
| Eritrea | 37836.23 (17035.51 to 67682.07) | 1237.51 (608.10 to 2144.17) | 1.09 (0.54 to 1.85) | 39370.22 (19296.27 to 70694.11) | 911.77 (451.65 to 1573.02) | 1.52 (0.79 to 2.57) | -1.05 (-1.37 to -0.74) |
| Estonia | 28217.27 (11683.74 to 49863.91) | 1462.66 (618.61 to 2538.23) | 3.85 (1.64 to 6.73) | 4032.79 (1312.26 to 7023.89) | 154.57 (52.05 to 267.16) | 0.61 (0.21 to 1.06) | -8.31 (-8.85 to -7.77) |
| Eswatini | 8761.44 (3732.77 to 16725.54) | 1426.67 (640.85 to 2551.02) | 2.32 (1.02 to 4.27) | 15251.49 (5446.19 to 27049.17) | 2064.70 (795.48 to 3567.71) | 2.08 (0.84 to 3.46) | 1.33 (0.81 to 1.85) |
| Ethiopia | 419834.69 (230542.80 to 643642.09) | 713.18 (428.67 to 1062.73) | 0.63 (0.38 to 0.96) | 349295.88 (219883.97 to 525780.27) | 432.64 (270.28 to 644.06) | 0.83 (0.53 to 1.24) | -1.25 (-1.42 to -1.07) |
| Fiji | 4202.19 (953.67 to 11601.05) | 962.13 (220.40 to 2663.54) | 2.10 (0.50 to 5.66) | 14063.14 (4311.10 to 28895.40) | 1788.19 (554.38 to 3632.32) | 3.78 (1.24 to 7.67) | 1.79 (1.39 to 2.19) |
| Finland | 37519.80 (6980.12 to 74732.81) | 545.41 (110.40 to 1063.50) | 1.95 (0.39 to 3.87) | 8196.55 (1177.05 to 17319.69) | 69.28 (12.75 to 141.09) | 0.35 (0.07 to 0.70) | -7.06 (-7.46 to -6.65) |
| France | 570019.46 (274735.40 to 913429.75) | 714.75 (349.97 to 1130.25) | 2.73 (1.37 to 4.35) | 245392.29 (168918.71 to 330565.70) | 186.90 (127.72 to 252.48) | 0.94 (0.63 to 1.31) | -4.32 (-4.59 to -4.04) |
| Gabon | 28560.68 (11432.60 to 42764.89) | 3206.99 (1448.62 to 4728.65) | 4.98 (2.21 to 7.17) | 30736.76 (19286.03 to 46127.37) | 2512.53 (1581.75 to 3689.80) | 4.61 (3.08 to 6.42) | -0.57 (-0.69 to -0.45) |
| Gambia | 17776.94 (8684.72 to 30532.50) | 1711.36 (884.49 to 2808.41) | 2.52 (1.28 to 4.14) | 18915.19 (10899.67 to 31340.92) | 1203.55 (687.33 to 1999.63) | 2.16 (1.30 to 3.57) | -1.02 (-1.23 to -0.82) |
| Georgia | 121129.59 (42602.27 to 223674.25) | 2167.11 (736.19 to 4034.11) | 5.57 (1.86 to 10.22) | 65494.17 (38075.60 to 94932.98) | 1169.76 (670.95 to 1698.11) | 3.45 (1.97 to 4.98) | -0.71 (-1.70 to 0.29) |
| Germany | 1757481.95 (946305.60 to 2675723.43) | 1432.87 (789.49 to 2157.99) | 5.17 (2.75 to 7.75) | 544474.88 (387883.78 to 719804.09) | 295.51 (209.99 to 391.47) | 1.41 (1.02 to 1.89) | -4.99 (-5.16 to -4.82) |
| Ghana | 233300.48 (125729.99 to 360876.27) | 1704.21 (973.72 to 2602.89) | 2.52 (1.45 to 3.82) | 432657.77 (210850.94 to 734383.84) | 1861.84 (929.71 to 3085.12) | 3.63 (1.81 to 5.77) | 1.15 (0.74 to 1.56) |
| Greece | 209146.29 (117600.54 to 317857.98) | 1483.20 (840.68 to 2216.31) | 5.83 (3.34 to 8.71) | 132245.29 (102977.78 to 164311.87) | 578.06 (455.23 to 706.25) | 2.63 (2.03 to 3.33) | -3.29 (-3.63 to -2.96) |
| Greenland | 265.50 (41.74 to 631.61) | 708.93 (90.88 to 1777.90) | 1.38 (0.17 to 3.43) | 145.28 (11.77 to 366.04) | 224.98 (25.80 to 548.91) | 0.70 (0.08 to 1.66) | -4.16 (-4.58 to -3.74) |
| Grenada | 1305.25 (408.59 to 2555.52) | 1674.74 (508.84 to 3262.86) | 4.42 (1.29 to 8.83) | 1785.13 (887.40 to 2739.11) | 1686.47 (848.97 to 2546.52) | 4.46 (2.31 to 6.91) | -0.11 (-0.27 to 0.05) |
| Guam | 588.47 (61.51 to 1452.69) | 684.43 (33.96 to 1763.69) | 2.32 (0.12 to 5.99) | 1238.72 (630.14 to 1847.94) | 632.29 (322.11 to 942.94) | 2.33 (1.25 to 3.44) | 0.40 (-0.29 to 1.10) |
| Guatemala | 163028.26 (65467.45 to 306171.74) | 1905.09 (790.27 to 3461.16) | 3.22 (1.34 to 5.89) | 109158.60 (40150.13 to 195818.87) | 919.52 (342.53 to 1629.21) | 2.25 (0.82 to 3.99) | -2.49 (-2.79 to -2.19) |
| Guinea | 154228.16 (72317.37 to 261422.89) | 1768.55 (892.90 to 2926.59) | 1.92 (0.94 to 3.17) | 118221.85 (61184.13 to 198674.43) | 1053.95 (576.18 to 1740.82) | 1.62 (0.92 to 2.60) | -1.05 (-1.30 to -0.80) |
| Guinea-Bissau | 22628.35 (11331.98 to 42390.52) | 2186.46 (1114.16 to 3727.08) | 2.12 (1.11 to 3.73) | 17110.02 (9428.58 to 28841.23) | 1300.77 (730.44 to 2129.83) | 1.81 (1.05 to 3.02) | -1.47 (-1.63 to -1.31) |
| Guyana | 11949.25 (3542.72 to 25754.35) | 2087.38 (567.27 to 4629.99) | 3.78 (1.02 to 8.59) | 15735.02 (7554.30 to 25082.87) | 2373.93 (1134.20 to 3795.13) | 4.55 (2.21 to 7.07) | 0.71 (0.52 to 0.90) |
| Haiti | 49451.74 (15342.17 to 104673.25) | 849.32 (273.22 to 1841.56) | 0.99 (0.32 to 2.12) | 56500.25 (23080.23 to 111340.88) | 610.16 (252.85 to 1168.21) | 0.92 (0.39 to 1.72) | -0.62 (-0.84 to -0.40) |
| Honduras | 32230.26 (14823.24 to 59096.64) | 820.68 (381.19 to 1454.99) | 1.88 (0.86 to 3.35) | 51014.34 (22498.08 to 88244.68) | 783.84 (344.93 to 1341.18) | 1.88 (0.84 to 3.15) | 0.23 (-0.05 to 0.51) |
| Hungary | 349029.56 (174035.68 to 523707.56) | 2626.19 (1316.30 to 3871.31) | 6.71 (3.39 to 9.85) | 163218.74 (123818.22 to 206978.99) | 868.11 (658.47 to 1101.74) | 3.06 (2.33 to 3.88) | -3.48 (-3.74 to -3.23) |
| Iceland | 905.33 (117.55 to 2030.57) | 318.00 (45.67 to 708.36) | 1.33 (0.19 to 2.99) | 369.08 (75.04 to 781.22) | 65.48 (15.19 to 135.71) | 0.36 (0.08 to 0.76) | -5.45 (-5.81 to -5.09) |
| India | 11073875.75 (5851741.82 to 18278435.73) | 1486.42 (819.57 to 2404.90) | 2.32 (1.28 to 3.79) | 27408370.34 (17694217.53 to 36250426.15) | 2382.38 (1528.11 to 3149.89) | 5.65 (3.67 to 7.45) | 1.96 (1.55 to 2.37) |
| Indonesia | 1666936.96 (711068.23 to 3089679.52) | 1203.60 (512.92 to 2251.44) | 2.28 (0.98 to 4.22) | 3826057.14 (2328962.03 to 5341691.89) | 1644.73 (1009.26 to 2279.86) | 4.31 (2.50 to 5.81) | 0.72 (0.56 to 0.87) |
| Iran (Islamic Republic of) | 1255256.42 (975420.61 to 1540490.69) | 3194.28 (2551.89 to 3802.12) | 6.78 (5.35 to 8.11) | 1198675.61 (992306.46 to 1389424.61) | 1609.40 (1334.64 to 1859.28) | 5.31 (4.36 to 6.30) | -2.03 (-2.20 to -1.86) |
| Iraq | 671202.52 (451016.05 to 900178.64) | 4622.03 (3128.12 to 5901.42) | 9.81 (6.59 to 12.49) | 875927.04 (661572.86 to 1126193.23) | 3489.71 (2629.47 to 4439.81) | 8.95 (7.04 to 10.92) | -0.60 (-0.78 to -0.41) |
| Ireland | 44634.34 (18385.68 to 76146.46) | 1125.97 (466.11 to 1911.57) | 3.95 (1.68 to 6.79) | 11680.49 (6248.89 to 17772.07) | 154.78 (83.40 to 235.05) | 0.80 (0.43 to 1.22) | -6.56 (-6.84 to -6.28) |
| Israel | 68742.94 (41847.72 to 96597.89) | 1445.34 (877.72 to 2036.33) | 5.82 (3.57 to 8.29) | 54304.32 (43814.87 to 66313.85) | 438.78 (351.32 to 538.05) | 2.44 (1.93 to 3.01) | -4.14 (-4.27 to -4.00) |
| Italy | 1083069.70 (657649.95 to 1530563.42) | 1322.78 (840.68 to 1839.71) | 5.10 (3.17 to 7.17) | 489929.60 (379002.83 to 611083.69) | 334.06 (262.70 to 414.18) | 1.77 (1.38 to 2.25) | -4.53 (-4.69 to -4.36) |
| Jamaica | 14405.18 (4487.16 to 30461.59) | 703.66 (218.85 to 1447.29) | 2.39 (0.71 to 5.00) | 26519.16 (14713.98 to 41289.23) | 934.88 (503.83 to 1445.17) | 3.08 (1.77 to 4.57) | 0.83 (0.57 to 1.10) |
| Japan | 804164.43 (177216.57 to 1679683.44) | 508.96 (120.98 to 1050.93) | 2.38 (0.57 to 4.91) | 930221.42 (527442.64 to 1368892.45) | 267.88 (155.02 to 387.65) | 1.66 (0.94 to 2.49) | -2.20 (-2.49 to -1.91) |
| Jordan | 59970.45 (42425.63 to 80057.80) | 2531.11 (1989.39 to 3091.76) | 7.19 (5.77 to 8.73) | 124022.50 (92188.72 to 157148.78) | 1585.35 (1193.21 to 1998.56) | 5.64 (4.44 to 6.83) | -1.85 (-2.13 to -1.56) |
| Kazakhstan | 324008.24 (129939.92 to 552165.44) | 2380.23 (955.59 to 4108.44) | 5.58 (2.28 to 9.70) | 305526.83 (218944.89 to 397284.11) | 1821.15 (1305.87 to 2376.29) | 4.75 (3.51 to 6.25) | -1.16 (-1.47 to -0.84) |
| Kenya | 89374.38 (46849.41 to 144840.12) | 369.30 (208.42 to 574.33) | 0.63 (0.35 to 0.99) | 139009.33 (74570.07 to 225577.65) | 449.83 (236.26 to 732.28) | 0.85 (0.45 to 1.39) | 1.49 (1.13 to 1.85) |
| Kiribati | 211.87 (59.74 to 540.44) | 384.40 (113.29 to 974.50) | 0.57 (0.17 to 1.47) | 394.24 (134.64 to 853.13) | 466.30 (158.53 to 1015.48) | 0.89 (0.32 to 2.00) | 0.41 (0.23 to 0.59) |
| Kuwait | 23685.75 (18632.87 to 28072.24) | 2900.47 (2370.92 to 3404.61) | 9.37 (7.64 to 11.18) | 61186.26 (46796.62 to 76210.97) | 1851.37 (1425.97 to 2301.23) | 8.48 (6.85 to 10.14) | -0.91 (-1.37 to -0.45) |
| Kyrgyzstan | 73225.36 (23505.31 to 156583.30) | 1892.31 (600.49 to 3900.17) | 4.10 (1.32 to 8.40) | 61444.21 (31546.26 to 95919.54) | 1242.71 (637.86 to 1933.90) | 3.78 (1.97 to 5.84) | -1.19 (-1.85 to -0.52) |
| Lao People's Democratic Republic | 59152.62 (19970.59 to 117106.69) | 1371.97 (522.25 to 2655.35) | 1.56 (0.60 to 3.04) | 79246.69 (31165.25 to 145367.99) | 1484.45 (600.58 to 2673.86) | 3.50 (1.40 to 5.98) | 0.22 (-0.11 to 0.55) |
| Latvia | 91409.19 (45630.66 to 140136.36) | 2678.95 (1328.29 to 4090.97) | 6.93 (3.48 to 10.80) | 27007.02 (18603.84 to 36290.49) | 697.69 (476.99 to 926.16) | 2.26 (1.53 to 2.99) | -4.81 (-5.18 to -4.43) |
| Lebanon | 45527.83 (29452.14 to 63653.09) | 1833.05 (1170.87 to 2571.81) | 4.43 (2.95 to 6.04) | 65517.41 (43587.79 to 89341.34) | 1097.29 (733.31 to 1483.74) | 3.70 (2.58 to 4.87) | -1.58 (-1.81 to -1.35) |
| Lesotho | 10379.15 (5944.15 to 16203.63) | 691.34 (405.61 to 1045.47) | 1.14 (0.67 to 1.74) | 14391.53 (7587.82 to 24178.48) | 1064.73 (559.39 to 1781.39) | 0.90 (0.49 to 1.50) | 2.26 (1.96 to 2.56) |
| Liberia | 43998.04 (22025.32 to 70460.32) | 1315.98 (712.16 to 2035.67) | 1.22 (0.68 to 1.88) | 24288.95 (14388.11 to 37418.15) | 664.41 (401.13 to 1014.46) | 1.13 (0.72 to 1.70) | -1.09 (-1.61 to -0.57) |
| Libya | 69562.45 (49922.82 to 91309.20) | 2363.77 (1769.59 to 3044.54) | 6.77 (5.17 to 8.53) | 131581.54 (97808.37 to 176585.38) | 2476.72 (1831.71 to 3300.33) | 6.61 (5.07 to 8.33) | 0.58 (0.28 to 0.87) |
| Lithuania | 93509.54 (44285.58 to 148853.50) | 2155.80 (1041.73 to 3410.88) | 6.08 (2.89 to 9.64) | 29703.98 (18572.27 to 42046.62) | 522.67 (328.34 to 732.25) | 1.75 (1.10 to 2.50) | -4.94 (-5.28 to -4.59) |
| Luxembourg | 5743.69 (2625.41 to 9225.91) | 1103.90 (514.63 to 1765.04) | 3.90 (1.79 to 6.25) | 1746.53 (1042.67 to 2500.34) | 168.86 (101.88 to 242.83) | 0.90 (0.53 to 1.30) | -5.87 (-6.15 to -5.59) |
| Madagascar | 36206.32 (18501.90 to 58048.96) | 315.05 (176.56 to 503.37) | 0.41 (0.23 to 0.65) | 55238.14 (32111.24 to 91217.18) | 305.46 (188.37 to 489.62) | 0.52 (0.33 to 0.83) | 0.91 (0.55 to 1.27) |
| Malawi | 70099.95 (34989.92 to 113322.38) | 551.37 (300.25 to 900.56) | 0.54 (0.30 to 0.88) | 47249.06 (27039.44 to 79497.26) | 378.10 (220.83 to 620.54) | 0.54 (0.33 to 0.87) | -0.91 (-1.16 to -0.66) |
| Malaysia | 295486.41 (150862.42 to 434674.88) | 2694.64 (1317.43 to 4057.02) | 8.07 (4.05 to 12.17) | 402717.72 (281195.04 to 530284.29) | 1443.07 (1008.95 to 1906.56) | 4.67 (3.19 to 6.26) | -2.12 (-2.33 to -1.92) |
| Maldives | 2508.41 (820.01 to 5129.77) | 1544.98 (500.92 to 3161.11) | 3.16 (1.03 to 6.39) | 2038.36 (1242.91 to 2916.31) | 600.22 (365.08 to 850.27) | 2.72 (1.66 to 3.89) | -3.94 (-4.27 to -3.62) |
| Mali | 183234.77 (97708.14 to 285605.16) | 1496.11 (823.61 to 2334.82) | 1.51 (0.82 to 2.37) | 295634.95 (161444.16 to 511528.70) | 1164.25 (659.25 to 1898.45) | 1.71 (0.99 to 2.75) | -0.12 (-0.39 to 0.14) |
| Malta | 5181.53 (2476.86 to 8024.64) | 1277.47 (629.91 to 1963.97) | 5.01 (2.45 to 7.70) | 3513.29 (2549.07 to 4627.30) | 391.41 (282.41 to 517.87) | 2.04 (1.47 to 2.68) | -3.95 (-4.22 to -3.68) |
| Marshall Islands | 200.29 (54.77 to 507.61) | 877.89 (241.41 to 2201.60) | 1.58 (0.44 to 3.87) | 385.95 (122.62 to 875.13) | 981.61 (320.45 to 2214.59) | 1.98 (0.66 to 4.36) | 0.07 (-0.20 to 0.34) |
| Mauritania | 49787.50 (26409.14 to 84283.11) | 2411.51 (1307.56 to 3963.23) | 3.79 (2.05 to 6.18) | 65558.27 (36761.31 to 101658.61) | 2049.34 (1163.29 to 3119.28) | 5.12 (2.92 to 7.53) | -0.55 (-0.72 to -0.37) |
| Mauritius | 7100.15 (3449.65 to 12164.24) | 908.32 (433.40 to 1561.80) | 2.43 (1.20 to 4.17) | 11524.42 (4159.36 to 20161.17) | 687.15 (275.90 to 1153.55) | 2.17 (0.84 to 3.63) | -1.14 (-1.54 to -0.73) |
| Mexico | 1411236.92 (800409.95 to 2049607.73) | 2153.90 (1230.34 to 3058.91) | 5.48 (3.15 to 7.85) | 1125565.29 (758253.22 to 1489390.27) | 918.36 (621.91 to 1208.19) | 2.52 (1.72 to 3.35) | -2.89 (-3.09 to -2.69) |
| Micronesia (Federated States of) | 503.21 (134.63 to 1360.26) | 787.78 (218.26 to 2147.14) | 1.43 (0.41 to 3.81) | 757.99 (244.41 to 1811.45) | 965.84 (311.31 to 2282.24) | 2.19 (0.70 to 4.85) | 0.67 (0.59 to 0.75) |
| Monaco | 310.36 (53.85 to 619.14) | 470.59 (90.25 to 916.04) | 1.96 (0.37 to 3.71) | 258.82 (134.87 to 396.77) | 283.52 (153.67 to 432.74) | 1.22 (0.67 to 1.86) | -1.42 (-2.37 to -0.46) |
| Mongolia | 24065.60 (7889.09 to 52569.19) | 1281.31 (457.99 to 2713.19) | 2.26 (0.82 to 4.79) | 51483.12 (24985.34 to 70611.63) | 2127.34 (1031.30 to 2911.89) | 5.68 (2.72 to 7.71) | 2.83 (2.25 to 3.40) |
| Montenegro | 12808.38 (5556.95 to 19647.03) | 2143.63 (920.12 to 3283.53) | 7.56 (3.24 to 11.47) | 13212.56 (8243.69 to 17132.75) | 1448.39 (895.22 to 1871.17) | 4.45 (2.84 to 5.74) | -0.81 (-0.97 to -0.65) |
| Morocco | 403029.57 (226347.21 to 631847.50) | 1896.42 (1127.03 to 2827.94) | 4.00 (2.35 to 6.01) | 690858.15 (474486.09 to 904286.68) | 2075.01 (1431.02 to 2692.73) | 6.06 (4.40 to 7.80) | 0.53 (0.16 to 0.91) |
| Mozambique | 72110.85 (41016.74 to 112253.16) | 442.43 (272.58 to 671.73) | 0.46 (0.28 to 0.70) | 87367.46 (51756.67 to 135680.77) | 410.63 (256.95 to 628.93) | 0.52 (0.32 to 0.79) | 0.58 (0.31 to 0.86) |
| Myanmar | 500545.59 (191451.25 to 926171.86) | 1517.65 (639.55 to 2812.40) | 1.91 (0.80 to 3.35) | 876114.98 (400002.45 to 1451350.53) | 1825.12 (838.84 to 3002.64) | 4.22 (1.92 to 6.88) | 0.61 (0.33 to 0.89) |
| Namibia | 15717.01 (5595.35 to 30233.67) | 1408.49 (515.80 to 2504.83) | 2.43 (0.92 to 4.44) | 29761.43 (10782.75 to 48726.98) | 1795.44 (696.71 to 2889.83) | 2.72 (1.09 to 4.20) | 1.47 (1.24 to 1.70) |
| Nauru | 60.77 (5.91 to 195.85) | 907.54 (43.48 to 3119.92) | 1.49 (0.07 to 5.08) | 66.97 (14.41 to 148.32) | 935.18 (177.79 to 2089.76) | 1.65 (0.34 to 3.66) | -0.98 (-1.50 to -0.46) |
| Nepal | 355787.20 (166310.44 to 662933.16) | 1689.48 (825.18 to 3050.37) | 2.33 (1.15 to 4.22) | 363339.45 (228926.42 to 545591.09) | 1518.83 (956.79 to 2284.83) | 3.53 (2.26 to 5.18) | -0.21 (-0.70 to 0.27) |
| Netherlands | 257540.46 (138868.33 to 387620.64) | 1336.39 (727.41 to 1990.45) | 5.39 (2.96 to 8.10) | 96551.71 (69775.15 to 125685.54) | 288.28 (209.82 to 373.62) | 1.46 (1.07 to 1.95) | -5.25 (-5.50 to -5.00) |
| New Zealand | 12641.09 (1102.38 to 35375.06) | 334.08 (34.23 to 925.64) | 1.14 (0.11 to 3.15) | 12347.15 (5349.03 to 20003.58) | 156.55 (71.73 to 250.02) | 0.77 (0.35 to 1.24) | -2.87 (-3.28 to -2.45) |
| Nicaragua | 21160.13 (8446.28 to 40494.72) | 565.12 (235.33 to 1064.90) | 1.53 (0.64 to 2.86) | 18642.14 (8258.38 to 34181.42) | 365.65 (162.88 to 666.88) | 1.14 (0.51 to 2.09) | -0.48 (-0.78 to -0.19) |
| Niger | 283385.51 (108182.18 to 513816.94) | 2272.33 (995.40 to 3889.32) | 2.09 (0.92 to 3.56) | 237526.53 (111397.51 to 423180.21) | 964.82 (510.92 to 1620.16) | 1.62 (0.86 to 2.73) | -2.36 (-2.65 to -2.06) |
| Nigeria | 3036242.55 (1395113.45 to 4945274.73) | 2523.79 (1238.04 to 3967.10) | 2.98 (1.48 to 4.73) | 4358410.94 (2079334.75 to 7728974.13) | 1943.68 (986.42 to 3259.99) | 3.37 (1.73 to 5.50) | 0.17 (-0.33 to 0.67) |
| Niue | 17.24 (5.80 to 35.69) | 778.17 (260.35 to 1606.76) | 1.96 (0.68 to 4.05) | 14.81 (2.88 to 30.89) | 785.79 (194.30 to 1607.00) | 1.74 (0.41 to 3.52) | -0.95 (-1.33 to -0.57) |
| North Macedonia | 59093.05 (25603.88 to 88159.28) | 3340.70 (1444.88 to 4977.34) | 8.95 (3.91 to 13.21) | 59622.06 (39377.35 to 75868.33) | 2088.73 (1361.87 to 2625.64) | 5.93 (3.98 to 7.18) | -1.26 (-1.60 to -0.92) |
| Northern Mariana Islands | 200.86 (21.98 to 523.53) | 856.56 (42.41 to 2281.08) | 2.56 (0.12 to 6.36) | 418.17 (209.81 to 619.25) | 814.67 (425.61 to 1190.17) | 2.50 (1.30 to 3.71) | -0.08 (-0.64 to 0.47) |
| Norway | 44666.26 (14562.67 to 79076.94) | 674.79 (229.52 to 1177.20) | 2.59 (0.91 to 4.56) | 8666.72 (3445.35 to 15023.56) | 88.96 (38.14 to 151.07) | 0.49 (0.21 to 0.84) | -6.95 (-7.21 to -6.69) |
| Oman | 50642.29 (35336.13 to 68484.34) | 4504.59 (3173.14 to 6005.40) | 11.07 (8.20 to 13.92) | 51389.89 (38939.07 to 65113.11) | 2381.13 (1787.77 to 3018.21) | 7.41 (5.79 to 8.96) | -1.54 (-1.72 to -1.37) |
| Pakistan | 1977351.14 (933842.53 to 3519496.33) | 1707.23 (840.51 to 2984.66) | 2.88 (1.41 to 5.03) | 4399130.74 (2290878.28 to 6814136.04) | 2501.60 (1296.20 to 3839.21) | 4.84 (2.55 to 7.39) | 1.39 (1.04 to 1.74) |
| Palau | 105.82 (8.26 to 254.77) | 969.22 (63.34 to 2346.33) | 2.08 (0.14 to 5.10) | 185.04 (22.25 to 370.61) | 878.32 (138.64 to 1762.28) | 2.16 (0.36 to 4.16) | 0.21 (-0.63 to 1.05) |
| Palestine | 30212.81 (19627.99 to 43533.77) | 2177.62 (1404.86 to 3079.09) | 5.28 (3.59 to 7.23) | 54331.72 (40826.92 to 69361.31) | 2045.97 (1497.79 to 2609.75) | 6.45 (4.75 to 8.19) | -0.45 (-0.84 to -0.06) |
| Panama | 15255.70 (5837.55 to 26497.19) | 848.16 (337.70 to 1476.97) | 2.94 (1.19 to 5.15) | 20785.87 (12092.79 to 31489.48) | 485.47 (281.60 to 731.21) | 1.88 (1.13 to 2.72) | -1.65 (-1.96 to -1.33) |
| Papua New Guinea | 26060.40 (6640.93 to 76089.89) | 844.90 (221.09 to 2228.45) | 1.40 (0.38 to 3.76) | 61241.05 (17790.22 to 144975.71) | 859.96 (253.11 to 2059.94) | 1.58 (0.51 to 3.61) | 0.01 (-0.18 to 0.20) |
| Paraguay | 18447.84 (4076.22 to 43861.72) | 564.72 (134.55 to 1372.52) | 1.83 (0.44 to 4.47) | 32262.75 (11782.56 to 61135.24) | 547.32 (201.03 to 1036.52) | 1.59 (0.59 to 3.01) | -0.01 (-0.15 to 0.12) |
| Peru | 508069.02 (220832.23 to 869980.27) | 2417.27 (1055.19 to 4083.78) | 5.60 (2.37 to 9.60) | 340308.19 (200855.52 to 502687.87) | 1006.73 (594.07 to 1486.01) | 2.96 (1.74 to 4.27) | -3.00 (-3.30 to -2.71) |
| Philippines | 688861.05 (302103.49 to 1190195.74) | 1496.32 (668.51 to 2532.10) | 3.44 (1.50 to 5.98) | 1329211.39 (753990.36 to 1867209.74) | 1519.80 (858.88 to 2136.25) | 3.70 (2.19 to 5.11) | -0.12 (-0.33 to 0.10) |
| Poland | 1087675.93 (549475.65 to 1619923.19) | 2633.00 (1330.04 to 3909.71) | 7.37 (3.60 to 10.95) | 645583.11 (518632.07 to 789773.25) | 931.87 (752.55 to 1137.72) | 3.48 (2.80 to 4.29) | -3.33 (-3.55 to -3.12) |
| Portugal | 120248.40 (49253.10 to 204585.62) | 958.31 (418.22 to 1583.78) | 3.07 (1.33 to 5.06) | 42747.55 (24018.10 to 64955.12) | 183.09 (103.79 to 277.26) | 0.89 (0.50 to 1.34) | -5.57 (-5.99 to -5.15) |
| Puerto Rico | 12876.43 (717.54 to 35954.21) | 372.42 (22.25 to 1026.43) | 1.20 (0.07 to 3.20) | 11695.93 (2977.31 to 21283.98) | 195.15 (58.97 to 347.50) | 0.84 (0.26 to 1.47) | -2.34 (-2.71 to -1.97) |
| Qatar | 8134.92 (6473.64 to 9875.07) | 5897.80 (4789.02 to 7042.39) | 15.45 (12.89 to 17.79) | 23965.60 (17863.96 to 30528.25) | 2272.68 (1714.65 to 2829.23) | 9.23 (7.55 to 10.89) | -3.42 (-4.00 to -2.83) |
| Republic of Korea | 472520.18 (217522.54 to 749694.94) | 1685.32 (770.48 to 2692.27) | 5.06 (2.33 to 8.23) | 603286.11 (439969.50 to 762710.98) | 681.47 (502.81 to 863.20) | 3.90 (2.83 to 5.06) | -3.20 (-3.45 to -2.96) |
| Republic of Moldova | 68672.06 (24500.29 to 128342.65) | 1687.05 (598.38 to 3170.09) | 4.01 (1.41 to 7.44) | 50842.46 (24323.39 to 82678.78) | 911.38 (430.49 to 1468.38) | 2.78 (1.37 to 4.53) | -1.21 (-1.82 to -0.60) |
| Romania | 595913.33 (258395.21 to 928018.83) | 2493.62 (1072.98 to 3905.05) | 6.35 (2.73 to 9.88) | 351255.31 (262110.14 to 440995.53) | 1013.20 (753.67 to 1277.60) | 3.27 (2.45 to 4.19) | -3.01 (-3.31 to -2.70) |
| Russian Federation | 4605556.39 (2313730.04 to 6924118.73) | 2806.64 (1459.83 to 4188.73) | 7.08 (3.65 to 10.71) | 1889916.42 (1146332.73 to 2862614.82) | 826.57 (507.47 to 1238.26) | 2.20 (1.33 to 3.31) | -4.69 (-5.21 to -4.17) |
| Rwanda | 74848.08 (32002.10 to 140020.68) | 1032.98 (500.41 to 1832.33) | 1.04 (0.52 to 1.85) | 30201.99 (13026.00 to 54603.77) | 341.24 (151.75 to 617.13) | 0.67 (0.31 to 1.27) | -4.16 (-4.44 to -3.87) |
| Saint Kitts and Nevis | 393.05 (122.58 to 763.91) | 1037.25 (311.21 to 2039.82) | 2.36 (0.72 to 4.63) | 274.38 (103.35 to 462.18) | 453.08 (180.95 to 749.26) | 1.32 (0.54 to 2.18) | -2.70 (-3.11 to -2.28) |
| Saint Lucia | 1679.97 (489.71 to 3272.98) | 1743.81 (528.22 to 3416.95) | 4.49 (1.30 to 8.64) | 2769.28 (1319.64 to 4231.52) | 1351.37 (704.84 to 2011.06) | 4.04 (2.20 to 6.02) | -1.13 (-1.34 to -0.92) |
| Saint Vincent and the Grenadines | 1222.81 (368.92 to 2419.82) | 1538.01 (453.71 to 3117.77) | 4.01 (1.17 to 7.87) | 1945.99 (893.62 to 2993.73) | 1517.24 (725.18 to 2308.42) | 4.43 (2.16 to 6.69) | -0.27 (-0.47 to -0.08) |
| Samoa | 710.66 (178.12 to 2078.09) | 670.62 (169.19 to 1985.78) | 1.62 (0.42 to 4.66) | 1438.74 (413.19 to 3209.38) | 929.69 (268.72 to 2090.62) | 2.59 (0.71 to 5.82) | 1.35 (1.23 to 1.46) |
| San Marino | 188.28 (76.13 to 318.50) | 569.84 (247.65 to 940.50) | 2.56 (1.16 to 4.36) | 114.85 (59.79 to 189.91) | 157.46 (83.37 to 260.19) | 0.92 (0.49 to 1.42) | -3.87 (-4.31 to -3.43) |
| Sao Tome and Principe | 800.24 (366.06 to 1345.01) | 681.63 (340.11 to 1117.76) | 1.21 (0.62 to 2.00) | 1109.36 (577.94 to 1834.76) | 886.47 (467.19 to 1467.13) | 2.42 (1.27 to 4.09) | 1.89 (1.53 to 2.24) |
| Saudi Arabia | 404424.67 (281864.74 to 536828.58) | 4211.61 (3105.95 to 5513.99) | 9.50 (7.58 to 11.68) | 763878.94 (563133.28 to 982268.13) | 3175.47 (2459.10 to 3957.59) | 9.95 (8.15 to 11.97) | -0.78 (-0.98 to -0.58) |
| Senegal | 177395.64 (94479.95 to 288160.65) | 2125.87 (1247.23 to 3365.35) | 3.02 (1.79 to 4.79) | 100973.03 (52413.47 to 183095.91) | 893.97 (447.80 to 1590.91) | 1.84 (0.96 to 3.13) | -3.18 (-3.89 to -2.48) |
| Serbia | 319085.61 (136803.24 to 476292.08) | 3413.82 (1427.42 to 5092.58) | 8.94 (3.62 to 13.45) | 254516.38 (172713.58 to 320591.30) | 1588.31 (1065.71 to 1989.70) | 5.54 (3.67 to 6.92) | -2.24 (-2.48 to -1.99) |
| Seychelles | 418.41 (168.26 to 723.34) | 693.74 (268.37 to 1214.92) | 1.95 (0.75 to 3.50) | 639.87 (255.85 to 1121.97) | 586.16 (244.76 to 1009.42) | 1.91 (0.81 to 3.17) | -1.00 (-1.29 to -0.70) |
| Sierra Leone | 85840.15 (39853.06 to 145022.38) | 1502.54 (726.20 to 2489.85) | 1.57 (0.77 to 2.54) | 57398.15 (31866.60 to 94779.13) | 820.13 (448.35 to 1307.75) | 1.30 (0.75 to 2.04) | -1.28 (-1.60 to -0.95) |
| Singapore | 52109.24 (22524.27 to 81086.37) | 2373.62 (1021.19 to 3711.33) | 9.15 (3.92 to 14.69) | 38990.82 (21642.09 to 56948.82) | 469.37 (259.42 to 687.27) | 3.15 (1.81 to 4.70) | -4.85 (-5.27 to -4.43) |
| Slovakia | 167150.25 (95507.45 to 241292.05) | 2938.64 (1713.18 to 4206.85) | 8.34 (4.68 to 12.09) | 83469.98 (63488.34 to 104569.43) | 921.42 (705.84 to 1150.03) | 3.29 (2.52 to 4.10) | -3.54 (-3.79 to -3.30) |
| Slovenia | 36088.79 (19607.51 to 52462.63) | 1546.24 (875.34 to 2220.06) | 5.19 (2.90 to 7.43) | 16185.73 (12051.68 to 20642.24) | 372.71 (278.56 to 476.58) | 1.84 (1.37 to 2.38) | -4.73 (-4.93 to -4.53) |
| Solomon Islands | 1101.35 (289.07 to 3017.98) | 533.10 (147.73 to 1450.03) | 0.90 (0.26 to 2.33) | 2298.69 (775.54 to 5262.88) | 558.49 (186.28 to 1255.06) | 1.19 (0.41 to 2.64) | 0.02 (-0.22 to 0.26) |
| Somalia | 51577.00 (27530.23 to 84286.66) | 611.53 (361.68 to 942.05) | 0.58 (0.36 to 0.90) | 79183.29 (45308.07 to 127765.88) | 433.05 (259.65 to 692.64) | 0.48 (0.29 to 0.74) | -0.19 (-0.66 to 0.29) |
| South Africa | 664733.64 (376050.27 to 994247.88) | 2030.11 (1216.54 to 2857.52) | 3.70 (2.22 to 5.26) | 906084.86 (635464.03 to 1180761.83) | 1922.47 (1351.54 to 2500.08) | 3.06 (2.18 to 4.00) | 0.23 (-0.10 to 0.56) |
| South Sudan | 140379.02 (53804.95 to 268251.01) | 2095.30 (869.14 to 3748.41) | 2.27 (0.95 to 4.04) | 85453.50 (43160.98 to 157639.86) | 909.29 (469.98 to 1625.62) | 1.10 (0.61 to 1.91) | -1.96 (-2.39 to -1.53) |
| Spain | 432319.96 (189590.06 to 699767.72) | 858.21 (390.15 to 1366.58) | 3.32 (1.57 to 5.23) | 199334.67 (130454.20 to 278304.31) | 212.02 (138.12 to 293.55) | 1.13 (0.75 to 1.57) | -4.49 (-4.81 to -4.17) |
| Sri Lanka | 82836.65 (30789.42 to 170149.61) | 746.44 (277.72 to 1531.30) | 1.91 (0.71 to 3.89) | 279269.58 (108133.46 to 435149.63) | 1102.17 (420.54 to 1710.67) | 4.21 (1.71 to 6.24) | 1.58 (1.27 to 1.89) |
| Sudan | 399921.74 (214674.10 to 671951.92) | 2032.06 (1181.83 to 3269.34) | 2.81 (1.63 to 4.58) | 605842.01 (337682.77 to 973059.09) | 2344.65 (1347.54 to 3690.75) | 5.35 (3.03 to 8.11) | 1.37 (1.10 to 1.64) |
| Suriname | 7698.29 (2507.37 to 13062.15) | 2483.39 (825.75 to 4315.43) | 5.80 (1.87 to 10.01) | 11972.82 (6618.83 to 17924.74) | 2020.01 (1162.12 to 2991.62) | 5.27 (2.99 to 7.61) | -0.67 (-0.93 to -0.41) |
| Sweden | 83090.69 (23235.83 to 154089.96) | 557.67 (171.11 to 1021.32) | 2.34 (0.74 to 4.30) | 16643.08 (5043.24 to 30748.60) | 78.34 (24.96 to 141.21) | 0.44 (0.14 to 0.78) | -6.60 (-6.95 to -6.25) |
| Switzerland | 99694.10 (50178.37 to 156168.44) | 980.48 (503.76 to 1513.29) | 3.85 (1.97 to 5.87) | 31725.91 (20697.52 to 44259.16) | 182.62 (119.07 to 257.02) | 1.02 (0.68 to 1.41) | -5.41 (-5.60 to -5.22) |
| Syrian Arab Republic | 259473.57 (175253.77 to 366704.89) | 3095.97 (2128.17 to 4276.23) | 7.45 (5.30 to 9.90) | 329675.66 (230862.28 to 452722.06) | 2677.35 (1897.17 to 3603.16) | 7.71 (5.87 to 10.20) | -0.50 (-0.75 to -0.26) |
| Taiwan (Province of China) | 190485.48 (85556.70 to 316939.40) | 1265.57 (565.81 to 2114.20) | 4.60 (2.01 to 7.84) | 239262.17 (186383.78 to 297203.58) | 601.90 (473.85 to 748.56) | 3.09 (2.42 to 3.92) | -2.27 (-2.59 to -1.95) |
| Tajikistan | 103583.93 (30589.91 to 218902.40) | 2003.07 (644.11 to 3991.88) | 4.18 (1.32 to 8.40) | 119398.56 (66783.12 to 186506.32) | 1633.90 (937.83 to 2512.07) | 4.18 (2.45 to 6.28) | 0.53 (-0.29 to 1.36) |
| Thailand | 604715.19 (249260.83 to 1048308.18) | 1587.12 (647.50 to 2772.73) | 4.31 (1.74 to 7.82) | 1286231.28 (917718.42 to 1724024.80) | 1298.47 (935.62 to 1733.29) | 4.75 (3.50 to 6.00) | -1.51 (-1.94 to -1.08) |
| Timor-Leste | 7248.05 (1893.33 to 16540.35) | 832.08 (255.97 to 1889.46) | 1.23 (0.39 to 2.79) | 11978.87 (4073.59 to 24196.10) | 1140.00 (392.39 to 2229.24) | 2.76 (0.90 to 5.50) | 1.80 (1.33 to 2.28) |
| Togo | 56676.09 (28202.04 to 95678.28) | 1510.82 (792.73 to 2484.85) | 2.09 (1.12 to 3.43) | 59537.26 (33116.38 to 98574.66) | 1032.06 (582.62 to 1706.37) | 1.85 (1.10 to 2.90) | -0.72 (-1.12 to -0.31) |
| Tokelau | 9.71 (0.75 to 30.79) | 698.90 (50.62 to 2226.29) | 1.66 (0.12 to 5.31) | 8.59 (0.80 to 20.21) | 667.83 (88.65 to 1493.68) | 1.47 (0.19 to 3.32) | -1.55 (-2.10 to -1.00) |
| Tonga | 327.96 (91.11 to 882.35) | 488.20 (133.37 to 1292.16) | 1.37 (0.39 to 3.63) | 669.26 (210.32 to 1471.74) | 786.32 (245.64 to 1733.58) | 2.51 (0.80 to 5.32) | 1.37 (1.12 to 1.62) |
| Trinidad and Tobago | 23520.83 (8392.59 to 41106.65) | 2709.75 (875.84 to 4774.64) | 6.83 (2.22 to 12.06) | 30770.47 (14225.76 to 49144.80) | 1719.18 (825.05 to 2736.61) | 4.69 (2.25 to 7.21) | -1.78 (-2.03 to -1.53) |
| Tunisia | 121611.65 (76205.15 to 174518.70) | 1892.15 (1190.70 to 2604.91) | 5.22 (3.36 to 7.06) | 190456.51 (131095.73 to 264475.28) | 1526.07 (1060.95 to 2104.12) | 4.81 (3.41 to 6.48) | -0.86 (-1.22 to -0.50) |
| Türkiye | 1270515.09 (696957.75 to 1761603.43) | 2771.02 (1578.17 to 3678.07) | 6.12 (3.53 to 8.10) | 1308100.30 (1018452.77 to 1628242.48) | 1485.85 (1151.99 to 1843.21) | 5.22 (4.18 to 6.34) | -1.92 (-2.21 to -1.63) |
| Turkmenistan | 109070.53 (38072.75 to 209776.15) | 3317.96 (1137.08 to 6098.61) | 6.93 (2.39 to 12.67) | 106076.66 (65863.44 to 156798.15) | 2548.14 (1584.29 to 3759.65) | 6.05 (3.82 to 8.86) | -1.14 (-1.43 to -0.84) |
| Tuvalu | 20.91 (6.56 to 51.69) | 233.85 (74.70 to 570.79) | 0.37 (0.12 to 0.90) | 33.03 (12.60 to 67.25) | 312.70 (119.26 to 635.81) | 0.76 (0.30 to 1.56) | 0.51 (0.19 to 0.83) |
| Uganda | 113649.88 (64165.53 to 181139.54) | 585.51 (359.18 to 882.89) | 0.56 (0.35 to 0.89) | 168651.24 (94878.50 to 271715.04) | 540.86 (305.18 to 850.41) | 0.88 (0.50 to 1.40) | -0.34 (-0.64 to -0.04) |
| Ukraine | 2120833.61 (1086178.61 to 3196692.66) | 3222.13 (1647.23 to 4832.51) | 8.62 (4.49 to 13.13) | 1064551.76 (589247.35 to 1647870.77) | 1428.85 (804.55 to 2198.83) | 3.95 (2.31 to 6.05) | -3.28 (-3.62 to -2.94) |
| United Arab Emirates | 29353.04 (22416.37 to 37935.32) | 4231.90 (3209.57 to 5435.04) | 11.24 (9.04 to 13.58) | 80006.22 (60568.05 to 99804.83) | 2255.61 (1706.84 to 2822.60) | 8.16 (6.54 to 10.14) | -0.67 (-1.19 to -0.15) |
| United Kingdom | 1169112.52 (572905.32 to 1849700.25) | 1348.67 (683.45 to 2115.04) | 4.92 (2.42 to 7.69) | 332920.15 (220722.48 to 451156.97) | 278.44 (189.98 to 373.48) | 1.26 (0.87 to 1.72) | -5.69 (-5.94 to -5.45) |
| United Republic of Tanzania | 143449.57 (72215.95 to 229793.39) | 456.76 (256.87 to 708.54) | 0.56 (0.32 to 0.87) | 209549.36 (109876.80 to 363582.48) | 475.38 (260.02 to 810.81) | 0.87 (0.47 to 1.45) | 0.95 (0.73 to 1.17) |
| United States of America | 2901452.15 (1292988.13 to 4773390.20) | 947.06 (433.81 to 1537.76) | 3.26 (1.45 to 5.36) | 1201097.10 (608366.37 to 1904323.05) | 224.24 (118.08 to 345.47) | 0.78 (0.42 to 1.22) | -5.06 (-5.45 to -4.66) |
| United States Virgin Islands | 494.14 (80.44 to 1178.57) | 568.89 (83.38 to 1396.98) | 1.69 (0.23 to 4.13) | 504.40 (235.93 to 803.92) | 318.85 (154.48 to 499.98) | 1.13 (0.57 to 1.78) | -1.79 (-2.13 to -1.44) |
| Uruguay | 35840.96 (15089.35 to 65316.16) | 982.76 (415.13 to 1758.51) | 3.06 (1.31 to 5.38) | 21109.81 (8497.96 to 36302.06) | 406.71 (176.96 to 691.19) | 1.46 (0.63 to 2.50) | -3.28 (-3.55 to -3.01) |
| Uzbekistan | 432976.47 (148513.82 to 850577.68) | 2368.80 (903.13 to 4377.45) | 5.85 (2.19 to 10.90) | 790769.38 (498803.60 to 1057423.84) | 2946.48 (1862.08 to 3952.10) | 8.97 (5.64 to 11.91) | 1.15 (0.76 to 1.54) |
| Vanuatu | 445.03 (111.75 to 1224.11) | 492.87 (121.35 to 1375.69) | 0.93 (0.24 to 2.57) | 1297.95 (435.03 to 3066.22) | 642.34 (216.97 to 1517.44) | 1.36 (0.46 to 2.84) | 1.57 (0.88 to 2.25) |
| Venezuela (Bolivarian Republic of) | 264862.99 (171505.05 to 377897.42) | 1930.07 (1156.20 to 2864.39) | 5.51 (3.35 to 8.19) | 315517.44 (195190.93 to 474616.44) | 1104.67 (689.19 to 1668.65) | 2.83 (1.86 to 4.03) | -1.82 (-1.97 to -1.67) |
| Viet Nam | 201188.31 (86528.49 to 381118.81) | 376.41 (163.32 to 732.68) | 0.95 (0.40 to 1.84) | 1018740.61 (511729.90 to 1543668.37) | 1085.83 (539.84 to 1657.45) | 3.81 (1.87 to 5.67) | 4.59 (4.21 to 4.96) |
| Yemen | 305255.82 (153524.84 to 492608.24) | 2300.03 (1173.44 to 3590.61) | 3.26 (1.69 to 5.08) | 470616.96 (266893.09 to 728734.40) | 2334.27 (1302.60 to 3571.38) | 4.53 (2.53 to 6.73) | 0.23 (0.04 to 0.42) |
| Zambia | 72027.65 (33272.83 to 121999.35) | 824.42 (439.04 to 1372.90) | 0.89 (0.46 to 1.47) | 98902.36 (46497.23 to 190246.16) | 834.48 (412.36 to 1527.73) | 1.14 (0.58 to 2.06) | -0.05 (-0.81 to 0.71) |
| Zimbabwe | 56470.13 (26104.08 to 101360.08) | 654.13 (320.58 to 1134.99) | 1.10 (0.54 to 1.91) | 67177.99 (33884.55 to 115294.57) | 612.12 (327.16 to 1020.74) | 0.71 (0.39 to 1.16) | 0.14 (-0.34 to 0.61) |

Table S14. Number and age-standardised rates of deaths attributable to ambient particulate matter pollution in 1990 and 2021, and estimated annual percentage changes from 1990 to 2021, by country.

| **Cause of deaths** | **1990** |  |  | **2021** |  |  | **1990–2021** |
| --- | --- | --- | --- | --- | --- | --- | --- |
|  | **Number of cases** | **Age-standardised rates per 100 000 people** | **Age-standardised PAF (%)** | **Number of cases** | **Age-standardised rates per 100 000 people** | **Age-standardised PAF (%)** | **EAPC in age-standardised rates (%)** |
| Afghanistan | 5039.79 (2269.23 to 9252.76) | 66.85 (30.37 to 121.57) | 3.34 (1.59 to 5.96) | 4502.00 (2804.47 to 6925.27) | 43.91 (27.32 to 66.37) | 2.16 (1.39 to 3.20) | -1.02 (-1.50 to -0.54) |
| Albania | 828.33 (358.99 to 1596.54) | 45.15 (19.53 to 87.25) | 5.30 (2.31 to 10.16) | 1926.59 (1148.35 to 2591.32) | 47.86 (28.04 to 64.28) | 6.36 (3.68 to 8.42) | 1.54 (1.10 to 1.98) |
| Algeria | 10562.24 (7415.54 to 13833.67) | 111.24 (77.72 to 148.20) | 10.63 (7.56 to 13.79) | 21558.50 (14929.80 to 28894.30) | 84.22 (58.42 to 112.51) | 8.80 (6.31 to 11.37) | -0.93 (-1.12 to -0.74) |
| American Samoa | 4.46 (0.19 to 13.68) | 21.79 (0.25 to 71.76) | 2.15 (0.02 to 7.05) | 9.25 (1.43 to 19.38) | 22.22 (3.49 to 46.61) | 2.30 (0.38 to 4.72) | -0.23 (-0.50 to 0.04) |
| Andorra | 16.21 (6.67 to 28.18) | 32.61 (13.48 to 56.51) | 5.90 (2.74 to 9.76) | 12.68 (6.93 to 19.92) | 7.42 (4.03 to 11.66) | 1.93 (1.10 to 2.91) | -4.44 (-4.81 to -4.07) |
| Angola | 2470.06 (1165.52 to 4337.61) | 40.85 (20.67 to 71.15) | 1.92 (0.98 to 3.18) | 8251.35 (3664.77 to 13738.87) | 68.21 (30.89 to 109.97) | 4.01 (1.84 to 6.35) | 2.49 (2.12 to 2.85) |
| Antigua and Barbuda | 33.18 (8.51 to 66.19) | 58.30 (14.91 to 116.30) | 7.52 (1.95 to 14.85) | 38.07 (15.82 to 63.21) | 41.07 (17.40 to 67.56) | 5.38 (2.29 to 8.81) | -1.26 (-1.49 to -1.03) |
| Argentina | 18287.29 (9240.97 to 30016.45) | 60.91 (30.73 to 100.46) | 7.33 (3.70 to 12.11) | 15268.28 (7687.79 to 23730.14) | 26.85 (13.76 to 41.28) | 3.97 (2.04 to 6.14) | -2.44 (-2.69 to -2.20) |
| Armenia | 2418.30 (874.43 to 4276.03) | 99.14 (35.63 to 177.17) | 10.94 (3.94 to 19.59) | 3983.52 (2730.26 to 5310.45) | 93.92 (64.79 to 124.83) | 12.52 (8.77 to 16.30) | 0.59 (0.07 to 1.12) |
| Australia | 3051.08 (149.35 to 8201.86) | 16.28 (0.95 to 43.51) | 2.54 (0.15 to 6.75) | 4246.84 (2581.37 to 6152.76) | 8.31 (5.11 to 11.95) | 2.29 (1.40 to 3.27) | -2.75 (-3.32 to -2.17) |
| Austria | 7963.30 (4358.38 to 12208.86) | 65.25 (35.85 to 99.58) | 9.18 (5.02 to 13.99) | 2921.56 (2098.85 to 3825.38) | 13.60 (9.79 to 17.75) | 3.12 (2.26 to 4.06) | -5.25 (-5.46 to -5.04) |
| Azerbaijan | 4545.95 (1519.97 to 8846.70) | 91.63 (32.00 to 179.75) | 8.71 (2.95 to 16.66) | 7598.12 (4126.49 to 11469.33) | 96.09 (51.88 to 144.01) | 9.18 (5.14 to 13.66) | 1.68 (0.78 to 2.60) |
| Bahamas | 82.02 (18.74 to 153.93) | 54.59 (11.36 to 104.26) | 6.47 (1.34 to 12.60) | 126.98 (47.07 to 225.95) | 35.26 (13.33 to 62.23) | 3.42 (1.32 to 5.93) | -1.48 (-1.67 to -1.29) |
| Bahrain | 385.83 (315.78 to 444.84) | 316.40 (255.45 to 367.85) | 23.41 (19.23 to 26.89) | 746.77 (597.36 to 906.28) | 150.62 (121.41 to 178.25) | 14.73 (12.28 to 17.07) | -2.77 (-3.19 to -2.35) |
| Bangladesh | 22673.35 (11043.37 to 40339.02) | 34.17 (16.95 to 60.30) | 2.37 (1.17 to 4.13) | 41852.53 (22482.72 to 70165.75) | 34.52 (18.51 to 57.59) | 3.93 (2.09 to 6.39) | -0.01 (-0.39 to 0.36) |
| Barbados | 206.45 (62.20 to 372.20) | 69.39 (21.73 to 124.10) | 8.86 (2.78 to 15.54) | 234.83 (109.15 to 364.24) | 46.81 (22.84 to 72.32) | 6.91 (3.40 to 10.63) | -1.25 (-1.44 to -1.07) |
| Belarus | 18168.26 (10009.58 to 26477.80) | 149.44 (82.46 to 217.93) | 16.38 (9.03 to 24.00) | 11135.03 (7754.92 to 15417.87) | 68.37 (47.60 to 94.58) | 6.63 (4.76 to 8.99) | -3.14 (-3.53 to -2.75) |
| Belgium | 10254.08 (5358.71 to 15732.16) | 65.92 (34.68 to 100.96) | 9.44 (4.95 to 14.47) | 3304.79 (2290.43 to 4395.93) | 12.04 (8.53 to 15.82) | 2.83 (2.01 to 3.71) | -5.37 (-5.69 to -5.05) |
| Belize | 51.56 (15.49 to 103.08) | 46.10 (13.02 to 94.01) | 6.61 (1.89 to 13.40) | 126.39 (53.75 to 212.36) | 46.15 (19.46 to 78.10) | 5.89 (2.39 to 9.67) | -0.45 (-0.96 to 0.07) |
| Benin | 1593.21 (806.70 to 2690.92) | 47.58 (25.86 to 76.51) | 2.96 (1.61 to 4.67) | 2179.17 (1218.21 to 3715.17) | 33.67 (19.03 to 58.28) | 2.41 (1.40 to 4.06) | -0.41 (-0.85 to 0.02) |
| Bermuda | 16.56 (0.14 to 43.64) | 29.12 (0.33 to 76.71) | 3.42 (0.04 to 8.88) | 10.21 (1.94 to 19.39) | 6.86 (1.33 to 12.90) | 1.31 (0.27 to 2.42) | -5.32 (-5.72 to -4.92) |
| Bhutan | 103.08 (49.68 to 191.34) | 31.44 (14.88 to 56.81) | 2.28 (1.12 to 4.02) | 429.20 (270.80 to 565.07) | 77.69 (49.27 to 101.68) | 10.11 (6.26 to 12.84) | 3.61 (3.24 to 3.99) |
| Bolivia (Plurinational State of) | 3991.10 (1576.75 to 6892.82) | 103.25 (44.00 to 175.08) | 7.68 (3.30 to 12.94) | 3875.09 (1910.73 to 6319.79) | 49.11 (24.02 to 80.03) | 3.39 (1.70 to 5.34) | -2.73 (-2.99 to -2.47) |
| Bosnia and Herzegovina | 1151.33 (611.16 to 2002.21) | 33.41 (17.70 to 58.02) | 4.20 (2.23 to 7.34) | 4240.46 (2171.55 to 5600.33) | 67.07 (34.23 to 88.68) | 8.86 (4.53 to 11.23) | 2.92 (2.26 to 3.57) |
| Botswana | 324.93 (129.29 to 594.49) | 54.62 (22.15 to 98.55) | 3.70 (1.54 to 6.34) | 799.08 (420.43 to 1153.78) | 61.72 (33.60 to 89.21) | 3.25 (1.73 to 4.57) | 0.59 (0.04 to 1.13) |
| Brazil | 38894.26 (13926.91 to 70978.80) | 46.56 (16.63 to 86.38) | 5.03 (1.80 to 9.34) | 52992.70 (30246.52 to 77620.81) | 22.00 (12.60 to 32.11) | 2.96 (1.70 to 4.34) | -2.41 (-2.60 to -2.22) |
| Brunei Darussalam | 26.75 (2.64 to 63.93) | 30.21 (2.28 to 74.57) | 3.01 (0.23 to 7.50) | 40.20 (8.75 to 75.41) | 15.69 (3.44 to 29.88) | 2.34 (0.54 to 4.57) | -1.47 (-2.21 to -0.73) |
| Bulgaria | 15758.57 (8323.73 to 23819.80) | 175.84 (91.81 to 266.08) | 14.36 (7.47 to 21.88) | 10031.41 (7662.72 to 12860.97) | 71.15 (54.56 to 91.05) | 5.84 (4.46 to 7.36) | -3.07 (-3.32 to -2.82) |
| Burkina Faso | 2622.47 (1278.00 to 4293.96) | 36.50 (19.22 to 59.87) | 1.93 (0.99 to 3.08) | 3722.57 (2014.12 to 6162.72) | 30.91 (16.86 to 50.88) | 1.94 (1.08 to 3.10) | 0.32 (0.01 to 0.64) |
| Burundi | 904.16 (437.73 to 1679.21) | 28.57 (13.98 to 51.59) | 1.26 (0.62 to 2.29) | 765.17 (396.47 to 1330.95) | 14.77 (7.39 to 25.88) | 0.97 (0.50 to 1.62) | -2.52 (-2.78 to -2.26) |
| Cabo Verde | 74.93 (43.67 to 113.14) | 27.84 (16.46 to 42.31) | 3.28 (1.94 to 4.95) | 360.11 (220.47 to 515.46) | 86.64 (52.99 to 123.96) | 10.32 (6.66 to 14.41) | 3.82 (3.17 to 4.47) |
| Cambodia | 1955.88 (683.02 to 4025.63) | 30.69 (11.42 to 60.52) | 1.86 (0.73 to 3.68) | 3458.98 (1696.51 to 6432.96) | 34.17 (16.91 to 63.11) | 2.92 (1.44 to 5.15) | 0.41 (0.14 to 0.68) |
| Cameroon | 3073.29 (1429.33 to 5245.56) | 49.99 (24.40 to 86.09) | 3.33 (1.61 to 5.44) | 5897.66 (3027.59 to 10349.25) | 44.57 (22.78 to 79.16) | 2.71 (1.41 to 4.71) | 0.34 (0.06 to 0.63) |
| Canada | 8292.34 (2534.40 to 15391.15) | 26.05 (8.03 to 48.20) | 4.28 (1.32 to 7.94) | 3261.42 (1239.29 to 5739.34) | 4.16 (1.64 to 7.23) | 0.99 (0.39 to 1.74) | -6.19 (-6.63 to -5.74) |
| Central African Republic | 570.21 (285.72 to 986.32) | 35.89 (19.52 to 60.07) | 1.45 (0.81 to 2.42) | 679.29 (364.99 to 1076.54) | 28.42 (15.82 to 44.46) | 1.12 (0.62 to 1.73) | -0.57 (-0.72 to -0.41) |
| Chad | 1821.90 (922.95 to 3003.11) | 38.34 (20.60 to 61.84) | 2.20 (1.20 to 3.63) | 3981.19 (1981.64 to 6879.74) | 43.75 (23.58 to 73.44) | 2.60 (1.48 to 4.17) | 0.99 (0.70 to 1.28) |
| Chile | 4086.85 (1439.05 to 7823.90) | 45.28 (15.87 to 87.33) | 5.77 (2.03 to 11.14) | 6654.77 (4317.57 to 8838.74) | 25.83 (16.86 to 33.99) | 4.88 (3.20 to 6.42) | -1.90 (-2.19 to -1.61) |
| China | 449466.87 (216120.31 to 792725.15) | 74.83 (35.44 to 131.25) | 6.25 (3.00 to 10.87) | 1857360.27 (1300499.81 to 2293490.62) | 102.34 (71.92 to 126.30) | 15.88 (11.13 to 18.72) | 1.54 (1.12 to 1.97) |
| Colombia | 10347.62 (4752.51 to 16883.27) | 58.74 (27.40 to 95.12) | 7.33 (3.42 to 11.91) | 12762.89 (8351.23 to 17375.97) | 22.94 (15.01 to 31.20) | 3.57 (2.33 to 4.82) | -3.38 (-3.54 to -3.22) |
| Comoros | 38.11 (19.76 to 66.50) | 13.28 (6.66 to 23.71) | 0.85 (0.43 to 1.49) | 40.28 (20.85 to 70.67) | 8.86 (4.45 to 15.31) | 0.72 (0.38 to 1.27) | -1.21 (-1.36 to -1.06) |
| Congo | 570.62 (233.64 to 1148.62) | 52.92 (21.41 to 102.00) | 2.62 (1.03 to 5.11) | 1548.85 (642.43 to 2688.34) | 69.53 (29.07 to 118.68) | 4.12 (1.75 to 6.76) | 1.23 (0.96 to 1.50) |
| Cook Islands | 3.18 (0.36 to 8.02) | 28.16 (2.20 to 71.68) | 2.55 (0.20 to 6.43) | 3.54 (0.01 to 8.14) | 14.18 (0.09 to 32.49) | 2.11 (0.02 to 4.86) | -2.62 (-3.01 to -2.23) |
| Costa Rica | 667.47 (290.68 to 1103.62) | 38.29 (16.81 to 63.72) | 6.43 (2.80 to 10.82) | 930.32 (618.46 to 1274.27) | 16.91 (11.39 to 23.10) | 3.00 (2.02 to 4.10) | -2.75 (-3.11 to -2.39) |
| Côte d'Ivoire | 4128.37 (2006.27 to 6770.55) | 64.53 (33.01 to 104.33) | 3.89 (2.05 to 6.24) | 6697.20 (3197.60 to 11376.75) | 53.91 (26.51 to 92.45) | 3.79 (1.92 to 6.29) | 0.02 (-0.37 to 0.42) |
| Croatia | 6077.86 (3189.44 to 9219.02) | 116.93 (61.14 to 177.51) | 11.66 (6.04 to 17.67) | 3723.80 (2814.86 to 4744.49) | 38.24 (29.07 to 48.63) | 5.70 (4.40 to 7.27) | -3.51 (-3.77 to -3.25) |
| Cuba | 6416.73 (2228.32 to 12227.44) | 67.05 (23.43 to 127.66) | 9.24 (3.23 to 17.64) | 9167.69 (4478.31 to 14705.24) | 44.37 (21.78 to 71.21) | 5.37 (2.65 to 8.83) | -1.25 (-1.54 to -0.95) |
| Cyprus | 578.22 (277.81 to 917.49) | 110.69 (53.32 to 176.90) | 9.27 (4.45 to 14.84) | 502.09 (374.03 to 645.58) | 29.18 (21.65 to 37.29) | 5.50 (4.17 to 7.01) | -4.70 (-4.92 to -4.49) |
| Czechia | 17137.07 (9266.60 to 25550.31) | 128.42 (69.95 to 190.67) | 13.05 (7.10 to 19.37) | 7212.19 (5414.42 to 9135.89) | 31.19 (23.48 to 39.49) | 4.96 (3.74 to 6.27) | -4.19 (-4.48 to -3.89) |
| Democratic People's Republic of Korea | 6831.29 (2882.07 to 12813.71) | 53.79 (22.65 to 100.21) | 5.31 (2.30 to 9.62) | 8810.20 (5591.04 to 13247.32) | 29.74 (18.87 to 44.73) | 3.62 (2.34 to 5.28) | -1.41 (-1.69 to -1.12) |
| Democratic Republic of the Congo | 5049.21 (2512.37 to 9014.20) | 25.43 (13.30 to 43.99) | 1.42 (0.74 to 2.39) | 6337.00 (3683.78 to 9825.83) | 18.60 (10.84 to 29.36) | 1.13 (0.71 to 1.77) | -0.80 (-1.04 to -0.55) |
| Denmark | 5589.59 (2692.08 to 9060.91) | 65.61 (31.89 to 105.96) | 8.91 (4.32 to 14.39) | 1531.53 (950.92 to 2148.13) | 11.54 (7.22 to 16.13) | 2.59 (1.62 to 3.62) | -5.91 (-6.17 to -5.65) |
| Djibouti | 108.59 (49.27 to 182.47) | 61.07 (28.69 to 100.58) | 4.42 (2.08 to 7.25) | 407.74 (211.82 to 684.68) | 75.57 (39.12 to 127.98) | 5.21 (2.73 to 8.42) | 0.93 (0.65 to 1.21) |
| Dominica | 28.78 (7.71 to 60.90) | 49.02 (13.10 to 104.00) | 5.31 (1.37 to 11.26) | 41.27 (17.30 to 67.83) | 55.30 (23.81 to 90.69) | 5.44 (2.36 to 8.92) | 0.28 (0.07 to 0.50) |
| Dominican Republic | 1273.57 (347.80 to 2863.80) | 32.18 (8.75 to 70.37) | 4.07 (1.11 to 8.52) | 5018.60 (2117.37 to 8507.08) | 51.32 (21.53 to 87.07) | 7.02 (3.05 to 11.75) | 2.14 (1.81 to 2.48) |
| Ecuador | 4193.97 (2026.05 to 6394.38) | 76.55 (35.66 to 116.86) | 9.52 (4.43 to 14.53) | 4803.12 (2876.66 to 7114.57) | 32.25 (19.27 to 47.69) | 4.06 (2.50 to 5.82) | -3.04 (-3.51 to -2.55) |
| Egypt | 57524.68 (38299.66 to 76639.97) | 231.82 (154.41 to 308.65) | 14.32 (9.60 to 19.03) | 115654.44 (90067.65 to 144272.08) | 251.38 (196.60 to 310.18) | 17.69 (14.30 to 21.04) | 0.58 (0.26 to 0.89) |
| El Salvador | 1018.58 (418.41 to 1915.35) | 30.20 (12.34 to 57.83) | 3.64 (1.49 to 6.93) | 2112.10 (1056.93 to 3169.08) | 31.88 (15.95 to 47.88) | 4.01 (2.13 to 6.07) | 0.25 (-0.04 to 0.53) |
| Equatorial Guinea | 104.44 (48.55 to 187.33) | 41.95 (20.44 to 73.32) | 1.98 (0.97 to 3.45) | 524.93 (299.71 to 816.29) | 109.76 (59.92 to 169.08) | 6.79 (4.00 to 9.74) | 4.11 (3.24 to 4.99) |
| Eritrea | 624.33 (303.09 to 1091.86) | 38.92 (19.44 to 66.75) | 1.56 (0.79 to 2.68) | 895.70 (442.73 to 1542.06) | 33.99 (17.25 to 58.44) | 2.02 (1.06 to 3.34) | -0.54 (-0.88 to -0.21) |
| Estonia | 1378.43 (549.87 to 2439.31) | 71.38 (28.56 to 126.26) | 7.02 (2.76 to 12.48) | 213.59 (69.50 to 379.34) | 6.85 (2.26 to 12.16) | 1.06 (0.35 to 1.88) | -8.54 (-9.14 to -7.95) |
| Eswatini | 175.71 (77.57 to 323.48) | 51.59 (23.41 to 92.02) | 3.40 (1.53 to 6.18) | 397.53 (155.78 to 694.97) | 75.00 (30.19 to 130.29) | 2.55 (1.02 to 4.23) | 1.42 (0.90 to 1.94) |
| Ethiopia | 6605.58 (3911.35 to 9979.34) | 20.36 (12.58 to 30.28) | 0.81 (0.51 to 1.22) | 7495.71 (4596.46 to 11195.39) | 15.30 (9.37 to 22.93) | 1.11 (0.69 to 1.64) | -0.55 (-0.73 to -0.37) |
| Fiji | 121.28 (27.85 to 336.33) | 37.74 (8.85 to 104.21) | 2.95 (0.73 to 7.82) | 458.54 (138.74 to 955.93) | 71.22 (22.21 to 147.76) | 4.93 (1.58 to 10.21) | 1.74 (1.29 to 2.18) |
| Finland | 1849.16 (335.03 to 3782.97) | 25.88 (4.77 to 52.79) | 3.61 (0.67 to 7.40) | 436.41 (56.93 to 931.22) | 2.88 (0.43 to 6.08) | 0.71 (0.11 to 1.49) | -7.51 (-7.99 to -7.04) |
| France | 31211.25 (14786.38 to 50629.37) | 35.88 (17.07 to 58.05) | 5.67 (2.69 to 9.19) | 13393.99 (9056.08 to 18530.72) | 7.67 (5.24 to 10.56) | 1.95 (1.33 to 2.68) | -4.97 (-5.27 to -4.66) |
| Gabon | 695.42 (316.62 to 1019.24) | 114.62 (53.21 to 168.41) | 7.34 (3.51 to 10.68) | 887.01 (547.72 to 1320.03) | 98.92 (61.11 to 147.16) | 6.45 (4.07 to 9.06) | -0.35 (-0.48 to -0.23) |
| Gambia | 292.87 (149.20 to 495.93) | 54.46 (28.62 to 89.56) | 3.72 (1.95 to 5.95) | 471.12 (267.75 to 787.84) | 46.90 (26.09 to 78.12) | 3.07 (1.83 to 4.83) | -0.33 (-0.52 to -0.13) |
| Georgia | 4739.31 (1682.46 to 8874.80) | 84.38 (30.00 to 157.45) | 8.67 (3.07 to 16.26) | 3141.45 (1775.64 to 4589.59) | 50.14 (28.47 to 72.95) | 5.12 (2.91 to 7.45) | -0.25 (-1.28 to 0.79) |
| Germany | 94298.25 (49793.86 to 146712.21) | 71.77 (38.33 to 111.39) | 9.86 (5.28 to 15.30) | 29057.43 (20310.90 to 38616.60) | 12.87 (9.07 to 17.02) | 2.76 (1.95 to 3.65) | -5.43 (-5.64 to -5.22) |
| Ghana | 4387.93 (2486.48 to 6732.89) | 57.06 (33.31 to 85.67) | 3.87 (2.30 to 5.88) | 11037.50 (5580.54 to 18425.74) | 70.76 (35.41 to 119.31) | 5.07 (2.61 to 8.04) | 1.49 (1.09 to 1.89) |
| Greece | 10781.80 (5878.61 to 16625.78) | 75.37 (41.38 to 116.09) | 11.25 (6.18 to 17.32) | 7545.86 (5765.98 to 9504.31) | 25.38 (19.64 to 31.75) | 4.99 (3.84 to 6.24) | -3.79 (-4.23 to -3.36) |
| Greenland | 8.03 (0.82 to 20.73) | 29.42 (1.76 to 77.55) | 2.17 (0.13 to 5.69) | 5.24 (0.27 to 13.30) | 9.18 (0.53 to 23.22) | 1.21 (0.07 to 3.00) | -4.25 (-4.69 to -3.81) |
| Grenada | 52.01 (15.45 to 103.96) | 65.43 (19.29 to 130.69) | 6.94 (2.06 to 14.40) | 65.17 (30.04 to 104.45) | 66.63 (30.92 to 106.81) | 6.10 (2.93 to 9.99) | -0.20 (-0.45 to 0.06) |
| Guam | 17.41 (0.69 to 45.23) | 29.69 (0.38 to 79.19) | 3.57 (0.05 to 9.52) | 39.14 (19.29 to 59.47) | 18.65 (9.28 to 28.11) | 3.32 (1.67 to 5.02) | -0.68 (-1.48 to 0.12) |
| Guatemala | 2849.76 (1185.49 to 5244.29) | 65.91 (27.55 to 120.25) | 4.74 (1.97 to 8.61) | 3443.14 (1295.20 to 5957.45) | 34.54 (13.03 to 58.40) | 3.32 (1.25 to 5.46) | -2.28 (-2.61 to -1.95) |
| Guinea | 2463.55 (1250.97 to 4051.29) | 46.22 (24.59 to 75.44) | 2.60 (1.39 to 4.25) | 2474.10 (1341.30 to 4098.67) | 37.07 (19.79 to 61.68) | 2.24 (1.29 to 3.55) | -0.10 (-0.34 to 0.14) |
| Guinea-Bissau | 389.21 (196.50 to 683.25) | 67.70 (36.74 to 113.50) | 3.02 (1.64 to 5.10) | 367.34 (202.96 to 603.46) | 48.85 (27.55 to 79.40) | 2.44 (1.37 to 3.94) | -0.79 (-0.97 to -0.62) |
| Guyana | 303.87 (83.05 to 679.87) | 75.69 (19.76 to 176.35) | 5.57 (1.43 to 13.13) | 485.43 (219.06 to 791.84) | 84.79 (38.39 to 137.23) | 5.94 (2.63 to 10.07) | 0.65 (0.43 to 0.87) |
| Haiti | 1045.71 (336.04 to 2268.43) | 29.60 (9.64 to 64.36) | 1.59 (0.51 to 3.39) | 1508.20 (617.14 to 2863.21) | 23.35 (9.56 to 43.99) | 1.35 (0.58 to 2.61) | -0.35 (-0.54 to -0.17) |
| Honduras | 695.13 (324.34 to 1241.90) | 29.65 (13.48 to 52.81) | 3.27 (1.46 to 5.95) | 1925.31 (847.66 to 3247.99) | 36.18 (16.16 to 60.89) | 2.91 (1.33 to 4.82) | 1.06 (0.70 to 1.42) |
| Hungary | 15465.34 (7612.12 to 23589.47) | 114.63 (56.89 to 173.91) | 10.53 (5.22 to 16.00) | 8149.40 (6035.02 to 10337.16) | 39.00 (28.95 to 49.64) | 5.01 (3.72 to 6.39) | -3.32 (-3.61 to -3.04) |
| Iceland | 46.85 (3.78 to 105.70) | 15.50 (1.31 to 34.87) | 2.64 (0.22 to 5.99) | 19.07 (3.58 to 41.50) | 2.86 (0.57 to 6.19) | 0.79 (0.16 to 1.72) | -5.73 (-6.18 to -5.28) |
| India | 227944.54 (125551.71 to 367099.79) | 46.41 (25.20 to 73.10) | 3.34 (1.85 to 5.20) | 947583.68 (620124.11 to 1256544.07) | 90.95 (59.30 to 120.09) | 8.38 (5.48 to 10.94) | 2.66 (2.20 to 3.13) |
| Indonesia | 38884.30 (16641.02 to 72566.52) | 40.02 (16.87 to 74.48) | 3.28 (1.37 to 6.14) | 129281.10 (79057.59 to 180926.89) | 67.99 (40.96 to 95.03) | 6.10 (3.56 to 8.38) | 1.43 (1.29 to 1.57) |
| Iran (Islamic Republic of) | 29167.85 (23102.33 to 34846.86) | 122.77 (95.67 to 146.61) | 12.27 (9.51 to 14.57) | 49495.23 (40074.94 to 58218.89) | 74.05 (59.81 to 87.35) | 9.14 (7.37 to 10.81) | -1.63 (-1.76 to -1.49) |
| Iraq | 15495.78 (10403.78 to 20038.05) | 162.47 (106.35 to 213.61) | 15.91 (11.00 to 20.22) | 28831.40 (21246.70 to 37619.89) | 150.59 (112.04 to 193.25) | 13.79 (10.62 to 16.83) | -0.06 (-0.26 to 0.14) |
| Ireland | 2262.16 (932.40 to 3923.64) | 57.48 (23.73 to 99.73) | 7.13 (2.96 to 12.31) | 595.70 (313.73 to 920.09) | 7.11 (3.75 to 11.01) | 1.79 (0.94 to 2.77) | -6.81 (-7.13 to -6.48) |
| Israel | 3190.92 (1871.80 to 4668.49) | 70.24 (40.99 to 103.30) | 10.94 (6.41 to 16.06) | 2647.78 (2069.64 to 3218.47) | 19.29 (15.21 to 23.34) | 5.12 (4.03 to 6.17) | -4.52 (-4.69 to -4.35) |
| Italy | 55256.96 (32655.50 to 80060.15) | 63.77 (38.02 to 92.35) | 9.91 (5.91 to 14.31) | 29386.27 (22056.43 to 37100.23) | 15.72 (11.97 to 19.56) | 3.92 (3.00 to 4.88) | -4.60 (-4.81 to -4.39) |
| Jamaica | 504.57 (162.25 to 1035.33) | 26.39 (8.60 to 54.17) | 3.95 (1.30 to 8.03) | 1101.58 (584.73 to 1733.58) | 34.64 (18.29 to 54.49) | 4.60 (2.52 to 7.00) | 0.87 (0.58 to 1.16) |
| Japan | 38489.64 (8141.15 to 82171.46) | 25.09 (5.37 to 53.53) | 4.74 (1.02 to 10.12) | 49305.72 (26184.23 to 75288.88) | 9.90 (5.41 to 14.84) | 3.18 (1.73 to 4.77) | -3.05 (-3.36 to -2.73) |
| Jordan | 1340.64 (1031.34 to 1661.91) | 97.54 (75.41 to 120.74) | 11.40 (9.00 to 13.66) | 3565.48 (2652.65 to 4592.99) | 61.61 (46.44 to 78.66) | 8.58 (6.71 to 10.59) | -1.91 (-2.26 to -1.57) |
| Kazakhstan | 10858.91 (4281.13 to 19361.42) | 93.72 (36.92 to 169.71) | 8.95 (3.48 to 16.16) | 12694.24 (8886.79 to 16911.21) | 89.43 (63.38 to 119.07) | 7.68 (5.40 to 10.21) | -0.38 (-0.70 to -0.06) |
| Kenya | 1479.23 (808.74 to 2340.91) | 11.71 (6.71 to 18.46) | 0.92 (0.52 to 1.44) | 3571.40 (1854.34 to 5886.96) | 17.08 (8.83 to 28.06) | 1.14 (0.59 to 1.84) | 2.04 (1.69 to 2.38) |
| Kiribati | 5.03 (1.48 to 12.71) | 13.52 (3.90 to 34.08) | 0.80 (0.24 to 1.97) | 11.03 (3.71 to 24.26) | 17.53 (6.04 to 38.24) | 1.24 (0.45 to 2.73) | 0.62 (0.44 to 0.79) |
| Kuwait | 629.29 (512.39 to 746.85) | 115.38 (92.37 to 136.31) | 17.13 (13.74 to 20.16) | 1764.86 (1318.91 to 2276.06) | 70.95 (52.33 to 91.77) | 15.31 (12.19 to 18.36) | -1.11 (-1.67 to -0.55) |
| Kyrgyzstan | 2093.65 (673.98 to 4309.20) | 70.35 (23.09 to 144.04) | 6.53 (2.14 to 13.18) | 2234.01 (1134.69 to 3507.13) | 55.91 (28.23 to 88.27) | 6.46 (3.34 to 10.18) | -0.40 (-1.11 to 0.32) |
| Lao People's Democratic Republic | 1102.64 (413.47 to 2136.68) | 42.04 (17.82 to 81.14) | 2.15 (0.89 to 4.14) | 2361.14 (958.47 to 4195.59) | 58.79 (23.70 to 103.12) | 5.15 (1.99 to 8.75) | 1.06 (0.71 to 1.40) |
| Latvia | 4461.73 (2194.39 to 6960.16) | 129.27 (63.41 to 201.55) | 12.68 (6.20 to 19.71) | 1490.73 (1017.32 to 2025.43) | 33.02 (22.63 to 44.75) | 3.93 (2.67 to 5.34) | -4.70 (-5.03 to -4.36) |
| Lebanon | 1424.23 (899.12 to 2039.28) | 72.39 (44.84 to 105.46) | 7.27 (4.69 to 9.99) | 2838.46 (1865.68 to 3977.49) | 43.73 (28.88 to 60.90) | 5.63 (3.80 to 7.63) | -1.54 (-1.79 to -1.28) |
| Lesotho | 220.37 (130.51 to 332.29) | 22.22 (13.41 to 32.96) | 1.60 (0.98 to 2.42) | 379.34 (197.44 to 632.87) | 36.35 (19.02 to 60.61) | 1.09 (0.58 to 1.83) | 2.63 (2.28 to 2.98) |
| Liberia | 683.55 (368.20 to 1050.82) | 35.39 (20.15 to 54.16) | 1.82 (1.07 to 2.76) | 535.76 (323.49 to 823.90) | 24.70 (15.16 to 37.24) | 1.72 (1.10 to 2.50) | -0.05 (-0.48 to 0.39) |
| Libya | 1789.27 (1322.69 to 2351.49) | 88.68 (64.74 to 117.64) | 11.71 (8.80 to 14.83) | 4440.73 (3178.07 to 6100.09) | 98.57 (70.88 to 133.88) | 10.20 (7.74 to 13.01) | 0.88 (0.48 to 1.29) |
| Lithuania | 4607.87 (2113.51 to 7447.24) | 105.23 (48.44 to 170.01) | 11.52 (5.31 to 18.57) | 1706.80 (1063.86 to 2441.14) | 25.81 (16.10 to 36.90) | 3.25 (2.03 to 4.64) | -4.85 (-5.17 to -4.52) |
| Luxembourg | 295.28 (133.29 to 479.85) | 55.96 (25.51 to 90.72) | 7.49 (3.45 to 12.04) | 87.04 (49.35 to 124.99) | 7.39 (4.28 to 10.63) | 1.86 (1.10 to 2.68) | -6.19 (-6.54 to -5.85) |
| Madagascar | 681.98 (384.22 to 1093.18) | 10.35 (5.97 to 16.56) | 0.60 (0.35 to 0.95) | 1240.72 (759.68 to 1987.20) | 11.44 (6.99 to 18.60) | 0.71 (0.44 to 1.15) | 1.27 (0.93 to 1.61) |
| Malawi | 1058.66 (576.83 to 1726.73) | 15.77 (9.03 to 26.06) | 0.77 (0.45 to 1.28) | 1072.98 (623.98 to 1774.79) | 13.82 (8.17 to 22.43) | 0.69 (0.42 to 1.10) | -0.11 (-0.37 to 0.16) |
| Malaysia | 9627.56 (4630.45 to 14724.56) | 108.46 (49.70 to 168.84) | 12.88 (5.91 to 20.11) | 15196.57 (10415.68 to 20422.91) | 61.26 (41.57 to 82.62) | 6.98 (4.65 to 9.39) | -1.99 (-2.20 to -1.77) |
| Maldives | 53.28 (17.36 to 108.73) | 58.01 (19.53 to 116.24) | 4.92 (1.64 to 9.91) | 69.15 (41.81 to 102.44) | 24.60 (14.72 to 36.21) | 4.74 (2.80 to 6.88) | -3.73 (-4.12 to -3.35) |
| Mali | 2770.34 (1522.42 to 4338.12) | 41.46 (23.22 to 65.27) | 2.15 (1.18 to 3.32) | 4804.36 (2698.89 to 8063.90) | 36.39 (20.72 to 58.94) | 2.11 (1.22 to 3.36) | 0.33 (0.07 to 0.60) |
| Malta | 250.74 (114.56 to 396.58) | 63.22 (29.06 to 99.89) | 9.16 (4.26 to 14.49) | 180.40 (127.64 to 239.71) | 16.65 (11.98 to 21.92) | 4.24 (2.99 to 5.56) | -4.39 (-4.77 to -4.01) |
| Marshall Islands | 5.38 (1.50 to 13.51) | 34.43 (9.56 to 86.04) | 2.18 (0.60 to 5.34) | 10.89 (3.61 to 24.71) | 38.03 (12.88 to 85.32) | 2.74 (0.93 to 5.95) | 0.07 (-0.17 to 0.31) |
| Mauritania | 1007.24 (546.58 to 1659.55) | 81.84 (44.74 to 131.75) | 5.51 (3.02 to 8.74) | 1730.25 (970.30 to 2653.52) | 81.04 (45.56 to 123.53) | 7.55 (4.36 to 11.14) | -0.09 (-0.34 to 0.16) |
| Mauritius | 232.67 (108.56 to 409.48) | 36.05 (16.70 to 63.16) | 3.59 (1.65 to 6.25) | 413.90 (140.86 to 723.25) | 24.26 (8.61 to 42.02) | 3.08 (1.12 to 5.32) | -1.53 (-1.98 to -1.08) |
| Mexico | 34304.71 (19548.59 to 48826.76) | 81.57 (46.56 to 117.66) | 9.02 (5.14 to 12.98) | 41689.71 (28225.25 to 55373.63) | 36.02 (24.44 to 47.74) | 3.91 (2.66 to 5.19) | -2.79 (-3.03 to -2.55) |
| Micronesia (Federated States of) | 14.18 (3.97 to 38.65) | 30.08 (8.50 to 83.30) | 1.99 (0.55 to 5.31) | 23.61 (7.75 to 56.68) | 38.01 (12.47 to 89.71) | 3.06 (0.99 to 6.96) | 0.76 (0.68 to 0.84) |
| Monaco | 17.75 (3.02 to 35.89) | 22.68 (3.95 to 45.80) | 3.81 (0.68 to 7.63) | 14.30 (7.26 to 22.23) | 12.58 (6.48 to 19.74) | 2.26 (1.18 to 3.47) | -1.64 (-2.60 to -0.68) |
| Mongolia | 531.35 (190.58 to 1125.54) | 44.54 (16.42 to 92.53) | 3.28 (1.21 to 6.82) | 1711.25 (833.78 to 2341.53) | 94.07 (44.76 to 127.59) | 9.29 (4.38 to 12.62) | 3.58 (3.02 to 4.14) |
| Montenegro | 533.56 (239.48 to 837.29) | 92.05 (41.17 to 144.52) | 12.72 (5.75 to 19.60) | 675.04 (417.50 to 866.48) | 78.61 (48.22 to 100.99) | 6.85 (4.23 to 8.67) | 0.03 (-0.17 to 0.23) |
| Morocco | 10114.01 (6002.66 to 14995.33) | 67.34 (39.88 to 101.24) | 6.50 (3.91 to 9.48) | 25964.61 (17714.54 to 33910.27) | 88.13 (60.34 to 115.20) | 9.27 (6.66 to 12.00) | 1.16 (0.79 to 1.54) |
| Mozambique | 1160.22 (708.46 to 1767.54) | 12.68 (8.10 to 18.64) | 0.67 (0.43 to 0.98) | 1839.46 (1115.56 to 2796.89) | 14.58 (9.08 to 22.38) | 0.69 (0.44 to 1.04) | 1.31 (1.02 to 1.60) |
| Myanmar | 11682.93 (5112.99 to 21327.83) | 50.21 (22.48 to 89.86) | 2.79 (1.31 to 5.09) | 31202.17 (14703.33 to 51071.16) | 74.72 (35.59 to 121.88) | 6.49 (3.00 to 10.49) | 1.32 (1.03 to 1.61) |
| Namibia | 347.50 (126.94 to 620.34) | 51.56 (19.46 to 92.17) | 3.61 (1.40 to 6.53) | 853.03 (343.33 to 1392.95) | 69.31 (28.66 to 111.36) | 3.53 (1.47 to 5.35) | 1.56 (1.33 to 1.80) |
| Nauru | 1.47 (0.08 to 5.03) | 32.87 (0.51 to 120.43) | 1.89 (0.03 to 6.72) | 1.82 (0.34 to 4.11) | 34.18 (6.04 to 76.41) | 2.12 (0.40 to 4.61) | -0.95 (-1.46 to -0.43) |
| Nepal | 6254.57 (3037.75 to 11360.20) | 51.47 (25.46 to 91.57) | 3.34 (1.71 to 5.84) | 12679.68 (7971.81 to 18987.52) | 63.19 (40.01 to 93.82) | 5.29 (3.40 to 7.55) | 0.84 (0.30 to 1.38) |
| Netherlands | 12902.17 (6926.55 to 19750.42) | 63.74 (34.32 to 97.06) | 9.82 (5.29 to 14.97) | 5143.50 (3691.80 to 6756.40) | 13.25 (9.53 to 17.28) | 2.92 (2.11 to 3.81) | -5.44 (-5.72 to -5.16) |
| New Zealand | 589.48 (20.96 to 1719.58) | 15.44 (0.65 to 45.03) | 2.20 (0.09 to 6.38) | 612.85 (261.59 to 1001.64) | 6.80 (2.95 to 11.08) | 1.70 (0.74 to 2.77) | -3.15 (-3.52 to -2.77) |
| Nicaragua | 382.70 (157.12 to 721.61) | 17.88 (7.63 to 33.60) | 2.71 (1.16 to 5.09) | 607.94 (271.97 to 1092.03) | 13.92 (6.24 to 24.99) | 1.71 (0.78 to 3.10) | 0.19 (-0.18 to 0.56) |
| Niger | 3770.67 (1576.70 to 6665.39) | 54.94 (27.91 to 90.70) | 2.72 (1.36 to 4.42) | 3944.32 (1975.86 to 6809.70) | 33.14 (18.07 to 55.03) | 2.28 (1.26 to 3.77) | -1.07 (-1.33 to -0.81) |
| Nigeria | 47562.08 (23511.68 to 74818.32) | 66.84 (36.75 to 102.34) | 4.10 (2.27 to 6.33) | 74956.62 (37503.81 to 127182.19) | 62.24 (32.77 to 99.06) | 4.71 (2.47 to 7.52) | 0.71 (0.18 to 1.25) |
| Niue | 0.73 (0.25 to 1.51) | 31.15 (10.73 to 64.14) | 2.82 (1.00 to 5.99) | 0.53 (0.08 to 1.13) | 26.78 (4.17 to 56.62) | 2.40 (0.36 to 4.90) | -1.27 (-1.60 to -0.94) |
| North Macedonia | 2371.03 (1044.69 to 3565.85) | 144.31 (63.37 to 217.41) | 14.55 (6.47 to 21.69) | 2842.76 (1892.67 to 3635.81) | 115.62 (76.64 to 145.10) | 9.30 (6.22 to 11.30) | -0.63 (-1.12 to -0.14) |
| Northern Mariana Islands | 5.09 (0.27 to 13.75) | 35.29 (0.55 to 96.31) | 3.71 (0.05 to 9.40) | 13.62 (6.84 to 20.04) | 32.70 (16.52 to 48.20) | 3.49 (1.73 to 5.28) | -0.25 (-0.79 to 0.30) |
| Norway | 2420.55 (770.92 to 4342.79) | 32.71 (10.53 to 58.38) | 5.05 (1.62 to 9.06) | 457.84 (174.40 to 816.20) | 3.94 (1.55 to 6.89) | 1.05 (0.41 to 1.84) | -7.26 (-7.58 to -6.94) |
| Oman | 1257.29 (876.50 to 1674.15) | 180.38 (122.12 to 243.73) | 17.65 (13.19 to 22.55) | 1593.35 (1173.42 to 2048.31) | 109.49 (79.95 to 138.50) | 11.65 (8.99 to 14.14) | -1.06 (-1.27 to -0.85) |
| Pakistan | 37679.38 (18271.73 to 66160.93) | 52.45 (25.21 to 90.03) | 4.20 (2.02 to 7.08) | 102996.47 (53461.90 to 158515.31) | 86.70 (45.34 to 132.17) | 6.57 (3.43 to 9.88) | 1.70 (1.37 to 2.04) |
| Palau | 3.13 (0.13 to 7.91) | 35.83 (0.94 to 92.61) | 2.79 (0.08 to 7.00) | 5.79 (0.62 to 11.96) | 33.03 (3.69 to 68.12) | 2.94 (0.33 to 5.84) | 0.29 (-0.58 to 1.16) |
| Palestine | 844.32 (546.84 to 1192.05) | 97.78 (62.89 to 139.22) | 8.73 (5.77 to 12.07) | 1862.97 (1338.32 to 2395.09) | 96.80 (67.74 to 124.71) | 10.80 (7.61 to 13.68) | -0.32 (-0.77 to 0.13) |
| Panama | 492.64 (199.08 to 864.94) | 33.49 (13.61 to 59.12) | 5.62 (2.28 to 9.91) | 758.09 (415.48 to 1173.94) | 16.85 (9.29 to 25.97) | 3.14 (1.83 to 4.66) | -2.08 (-2.43 to -1.72) |
| Papua New Guinea | 555.97 (146.09 to 1481.37) | 31.12 (7.94 to 80.52) | 2.14 (0.57 to 5.49) | 1521.02 (447.03 to 3615.22) | 33.86 (10.05 to 78.61) | 2.28 (0.72 to 5.31) | 0.18 (-0.02 to 0.38) |
| Paraguay | 506.59 (123.18 to 1243.24) | 21.40 (5.30 to 51.05) | 3.34 (0.83 to 8.09) | 1187.74 (426.52 to 2297.80) | 21.58 (7.77 to 41.68) | 2.42 (0.88 to 4.62) | 0.28 (0.11 to 0.45) |
| Peru | 10589.73 (4509.12 to 17927.44) | 73.04 (32.56 to 120.99) | 8.73 (3.87 to 14.30) | 13166.76 (7362.27 to 19896.48) | 39.20 (22.00 to 59.26) | 4.14 (2.38 to 6.02) | -2.39 (-2.73 to -2.06) |
| Philippines | 16032.24 (7128.69 to 27113.52) | 56.67 (25.79 to 96.81) | 5.59 (2.58 to 9.69) | 43157.94 (24384.34 to 61047.58) | 59.21 (33.10 to 83.97) | 5.11 (2.99 to 7.09) | 0.00 (-0.23 to 0.23) |
| Poland | 47622.77 (23976.05 to 71773.91) | 116.85 (59.03 to 176.58) | 12.23 (6.16 to 18.46) | 32314.85 (25097.54 to 39742.78) | 42.34 (33.11 to 51.91) | 5.93 (4.65 to 7.27) | -3.30 (-3.54 to -3.07) |
| Portugal | 6031.79 (2351.51 to 10399.27) | 48.52 (19.24 to 83.43) | 5.86 (2.32 to 10.08) | 2276.00 (1241.67 to 3450.56) | 7.62 (4.26 to 11.48) | 1.73 (0.96 to 2.61) | -6.24 (-6.73 to -5.74) |
| Puerto Rico | 497.87 (10.28 to 1518.58) | 14.85 (0.33 to 45.31) | 1.96 (0.04 to 5.95) | 478.82 (125.79 to 848.27) | 5.98 (1.67 to 10.52) | 1.30 (0.35 to 2.30) | -3.28 (-3.70 to -2.87) |
| Qatar | 213.42 (170.71 to 257.64) | 296.16 (240.44 to 354.07) | 23.79 (19.97 to 27.08) | 562.12 (408.92 to 735.29) | 108.69 (79.87 to 136.71) | 16.08 (13.27 to 18.68) | -3.77 (-4.52 to -3.01) |
| Republic of Korea | 17171.58 (7447.13 to 28281.65) | 79.31 (34.05 to 131.63) | 8.40 (3.61 to 13.96) | 25477.95 (17512.32 to 33486.23) | 28.13 (19.31 to 36.90) | 7.51 (5.16 to 9.88) | -3.66 (-3.90 to -3.41) |
| Republic of Moldova | 2813.49 (976.16 to 5369.65) | 78.88 (27.10 to 150.64) | 6.99 (2.40 to 13.35) | 2331.51 (1107.20 to 3871.54) | 39.24 (18.70 to 64.92) | 4.51 (2.14 to 7.41) | -1.58 (-2.21 to -0.95) |
| Romania | 25345.59 (11188.98 to 40163.08) | 111.35 (48.70 to 175.73) | 10.41 (4.55 to 16.41) | 18186.97 (13530.67 to 23096.28) | 46.00 (34.12 to 58.06) | 5.19 (3.84 to 6.55) | -2.95 (-3.25 to -2.65) |
| Russian Federation | 204681.34 (98493.47 to 311578.94) | 131.33 (63.72 to 200.03) | 12.74 (6.18 to 19.42) | 91532.87 (56088.61 to 140748.31) | 38.31 (23.55 to 58.81) | 3.65 (2.25 to 5.62) | -4.66 (-5.15 to -4.17) |
| Rwanda | 1294.00 (625.90 to 2297.58) | 32.26 (16.19 to 57.03) | 1.43 (0.72 to 2.51) | 765.91 (341.91 to 1382.67) | 13.24 (6.12 to 24.52) | 0.95 (0.46 to 1.70) | -3.45 (-3.76 to -3.14) |
| Saint Kitts and Nevis | 15.76 (4.00 to 32.73) | 43.22 (10.81 to 90.18) | 3.56 (0.90 to 7.41) | 9.30 (3.51 to 15.76) | 17.28 (6.77 to 29.09) | 1.82 (0.71 to 3.10) | -2.90 (-3.30 to -2.50) |
| Saint Lucia | 61.25 (18.98 to 123.07) | 78.89 (24.01 to 158.44) | 7.20 (2.15 to 14.44) | 107.68 (49.01 to 170.48) | 48.85 (22.40 to 76.79) | 5.64 (2.58 to 8.91) | -2.21 (-2.55 to -1.85) |
| Saint Vincent and the Grenadines | 44.54 (13.09 to 90.81) | 65.02 (19.12 to 134.17) | 6.55 (1.92 to 13.50) | 76.23 (32.10 to 122.55) | 60.45 (25.87 to 96.71) | 6.70 (2.92 to 10.91) | -0.45 (-0.65 to -0.25) |
| Samoa | 21.52 (5.48 to 63.86) | 27.50 (6.99 to 80.33) | 2.43 (0.63 to 6.63) | 49.30 (14.32 to 110.24) | 38.24 (11.04 to 85.70) | 3.87 (1.06 to 8.55) | 1.32 (1.21 to 1.43) |
| San Marino | 10.12 (3.96 to 17.42) | 27.61 (11.08 to 47.27) | 5.22 (2.12 to 8.97) | 5.75 (2.98 to 9.66) | 5.92 (3.04 to 9.86) | 2.08 (1.16 to 3.11) | -4.45 (-4.96 to -3.94) |
| Sao Tome and Principe | 17.64 (8.86 to 28.77) | 23.05 (12.22 to 36.86) | 1.88 (1.03 to 2.94) | 36.08 (19.04 to 60.58) | 38.08 (20.10 to 64.83) | 3.61 (1.92 to 6.10) | 2.82 (2.46 to 3.20) |
| Saudi Arabia | 9992.91 (7413.29 to 13168.09) | 169.03 (123.11 to 223.21) | 15.36 (12.18 to 18.54) | 21491.07 (15993.95 to 27494.53) | 135.60 (105.68 to 167.37) | 15.80 (12.92 to 18.70) | -0.69 (-0.92 to -0.46) |
| Senegal | 3110.56 (1794.98 to 4961.02) | 67.18 (40.27 to 104.82) | 4.48 (2.65 to 6.88) | 2602.55 (1303.51 to 4555.77) | 34.31 (17.15 to 60.50) | 2.57 (1.31 to 4.31) | -2.61 (-3.35 to -1.86) |
| Serbia | 14093.70 (6189.74 to 21341.78) | 169.02 (73.66 to 256.86) | 14.14 (6.18 to 21.42) | 13607.68 (9125.17 to 17334.41) | 79.48 (53.19 to 101.17) | 8.89 (5.97 to 11.28) | -2.32 (-2.55 to -2.10) |
| Seychelles | 15.21 (5.05 to 26.89) | 26.62 (8.80 to 47.22) | 2.81 (0.93 to 5.05) | 20.86 (7.48 to 37.52) | 20.72 (7.58 to 37.20) | 2.49 (0.92 to 4.40) | -1.24 (-1.56 to -0.92) |
| Sierra Leone | 1388.04 (678.50 to 2302.76) | 41.46 (21.66 to 67.00) | 2.27 (1.20 to 3.64) | 1228.90 (672.80 to 1978.02) | 29.04 (16.47 to 45.90) | 1.95 (1.13 to 3.04) | -0.36 (-0.68 to -0.04) |
| Singapore | 2001.29 (831.24 to 3192.45) | 105.68 (44.28 to 169.69) | 15.21 (6.40 to 24.43) | 1547.23 (782.35 to 2362.64) | 18.80 (9.51 to 28.70) | 6.43 (3.26 to 9.70) | -5.15 (-5.53 to -4.76) |
| Slovakia | 7469.28 (4120.26 to 10996.91) | 131.15 (72.26 to 193.05) | 13.55 (7.45 to 19.86) | 4229.04 (3205.79 to 5388.19) | 44.85 (33.98 to 57.06) | 5.71 (4.34 to 7.27) | -3.22 (-3.48 to -2.96) |
| Slovenia | 1661.01 (883.62 to 2430.95) | 68.99 (36.87 to 100.60) | 9.08 (4.86 to 13.36) | 816.56 (596.82 to 1048.45) | 15.89 (11.62 to 20.42) | 3.37 (2.52 to 4.29) | -4.91 (-5.14 to -4.68) |
| Solomon Islands | 26.85 (7.32 to 72.65) | 21.33 (6.00 to 55.40) | 1.30 (0.38 to 3.35) | 68.39 (22.86 to 153.81) | 23.06 (7.75 to 51.82) | 1.75 (0.59 to 3.87) | 0.10 (-0.13 to 0.33) |
| Somalia | 777.50 (446.17 to 1233.33) | 18.60 (11.47 to 28.13) | 0.80 (0.51 to 1.19) | 1296.85 (765.12 to 2070.59) | 14.27 (8.59 to 22.21) | 0.56 (0.35 to 0.88) | 0.07 (-0.40 to 0.54) |
| South Africa | 14115.03 (8621.56 to 19660.32) | 59.76 (38.01 to 80.94) | 5.58 (3.64 to 7.57) | 27105.07 (19298.44 to 34785.39) | 65.75 (46.87 to 84.90) | 4.02 (2.85 to 5.19) | 0.71 (0.33 to 1.09) |
| South Sudan | 2384.21 (969.89 to 4296.69) | 61.55 (25.30 to 106.77) | 3.28 (1.34 to 5.63) | 1480.42 (763.56 to 2683.00) | 28.69 (15.01 to 50.89) | 1.45 (0.80 to 2.49) | -1.86 (-2.30 to -1.43) |
| Spain | 21682.41 (9294.51 to 36233.25) | 41.20 (17.96 to 68.70) | 6.38 (2.79 to 10.64) | 9910.86 (6443.02 to 13824.55) | 8.15 (5.35 to 11.20) | 2.11 (1.38 to 2.89) | -5.15 (-5.51 to -4.78) |
| Sri Lanka | 2952.20 (1096.70 to 5974.44) | 34.13 (12.70 to 69.26) | 3.39 (1.25 to 7.00) | 11377.63 (4589.89 to 17706.75) | 47.87 (19.40 to 74.20) | 7.28 (2.99 to 10.75) | 1.66 (1.32 to 2.00) |
| Sudan | 7877.41 (4606.42 to 12795.45) | 67.75 (39.60 to 106.86) | 4.58 (2.72 to 7.22) | 17315.00 (9769.49 to 27309.53) | 95.40 (52.68 to 149.81) | 8.22 (4.61 to 12.73) | 1.96 (1.70 to 2.22) |
| Suriname | 219.89 (72.31 to 386.22) | 86.26 (28.05 to 155.82) | 9.17 (2.95 to 16.53) | 393.40 (198.10 to 621.09) | 66.41 (33.91 to 104.46) | 7.39 (3.59 to 11.26) | -0.78 (-1.07 to -0.49) |
| Sweden | 4674.41 (1274.44 to 8809.29) | 28.23 (7.79 to 52.97) | 4.68 (1.30 to 8.78) | 910.82 (281.02 to 1703.40) | 3.45 (1.08 to 6.35) | 0.93 (0.29 to 1.73) | -7.07 (-7.49 to -6.64) |
| Switzerland | 5454.97 (2716.00 to 8767.67) | 49.00 (24.40 to 78.27) | 8.28 (4.12 to 13.26) | 1580.47 (1014.16 to 2188.46) | 7.05 (4.56 to 9.62) | 2.14 (1.40 to 2.94) | -6.16 (-6.40 to -5.93) |
| Syrian Arab Republic | 6671.55 (4566.55 to 9201.73) | 123.55 (85.29 to 172.89) | 11.96 (8.54 to 15.78) | 12777.00 (8811.72 to 17426.23) | 127.32 (89.16 to 171.38) | 12.65 (9.49 to 16.24) | -0.08 (-0.28 to 0.13) |
| Taiwan (Province of China) | 7287.80 (3203.14 to 12336.31) | 57.71 (25.17 to 97.70) | 7.74 (3.39 to 13.20) | 10450.84 (7871.96 to 13236.29) | 24.00 (18.16 to 30.30) | 5.45 (4.12 to 6.89) | -2.63 (-2.97 to -2.29) |
| Tajikistan | 2283.82 (731.61 to 4541.65) | 69.19 (21.81 to 137.41) | 6.36 (1.94 to 12.59) | 3277.85 (1876.43 to 5004.63) | 67.38 (38.42 to 104.45) | 6.51 (3.71 to 9.90) | 1.12 (0.28 to 1.96) |
| Thailand | 19071.60 (7705.03 to 33531.42) | 62.01 (25.17 to 109.73) | 7.22 (2.90 to 12.77) | 53355.78 (36744.41 to 73127.86) | 50.39 (34.67 to 69.03) | 8.12 (5.77 to 10.26) | -1.60 (-2.07 to -1.13) |
| Timor-Leste | 110.94 (32.68 to 253.72) | 24.91 (8.48 to 57.37) | 1.78 (0.61 to 3.94) | 361.72 (123.48 to 717.35) | 46.08 (15.72 to 88.05) | 4.13 (1.39 to 7.99) | 2.84 (2.37 to 3.30) |
| Togo | 922.05 (471.10 to 1530.15) | 48.11 (24.99 to 78.21) | 3.15 (1.72 to 5.10) | 1399.57 (780.25 to 2321.69) | 38.65 (22.19 to 64.57) | 2.65 (1.60 to 4.18) | -0.25 (-0.64 to 0.15) |
| Tokelau | 0.34 (0.02 to 1.12) | 26.63 (1.56 to 89.28) | 2.27 (0.14 to 7.53) | 0.29 (0.01 to 0.70) | 20.27 (1.19 to 48.41) | 1.98 (0.11 to 4.67) | -1.82 (-2.22 to -1.42) |
| Tonga | 10.11 (2.73 to 26.87) | 19.74 (5.43 to 51.86) | 2.13 (0.62 to 5.71) | 24.97 (7.96 to 54.08) | 31.98 (10.22 to 69.22) | 3.86 (1.27 to 8.27) | 1.41 (1.14 to 1.68) |
| Trinidad and Tobago | 823.66 (215.31 to 1508.32) | 111.51 (26.11 to 206.17) | 10.50 (2.48 to 19.46) | 1165.21 (498.81 to 1933.39) | 63.19 (27.43 to 104.07) | 6.86 (3.05 to 11.13) | -2.10 (-2.36 to -1.83) |
| Tunisia | 3286.50 (2117.04 to 4515.35) | 73.24 (46.01 to 102.10) | 8.94 (5.56 to 12.12) | 7760.37 (5139.64 to 10937.08) | 66.24 (44.57 to 93.22) | 7.62 (5.26 to 10.06) | -0.57 (-0.97 to -0.17) |
| Türkiye | 33273.79 (19338.55 to 43314.33) | 98.36 (59.55 to 125.25) | 9.43 (5.67 to 11.92) | 57000.06 (43349.71 to 71884.74) | 67.87 (51.79 to 85.22) | 8.78 (6.94 to 10.62) | -0.94 (-1.32 to -0.56) |
| Turkmenistan | 2433.59 (819.92 to 4480.48) | 116.42 (35.91 to 220.14) | 10.42 (3.22 to 19.63) | 3643.70 (2196.21 to 5587.97) | 104.41 (62.81 to 160.41) | 9.09 (5.58 to 13.37) | -0.82 (-1.13 to -0.51) |
| Tuvalu | 0.52 (0.17 to 1.28) | 8.26 (2.63 to 20.01) | 0.50 (0.16 to 1.19) | 1.14 (0.44 to 2.34) | 12.48 (4.80 to 25.56) | 1.08 (0.42 to 2.18) | 0.86 (0.50 to 1.22) |
| Uganda | 1915.90 (1161.78 to 2935.11) | 19.35 (11.89 to 29.08) | 0.91 (0.57 to 1.38) | 3385.36 (1905.05 to 5377.43) | 19.54 (10.72 to 31.16) | 1.22 (0.70 to 1.95) | -0.14 (-0.43 to 0.16) |
| Ukraine | 102221.77 (50898.76 to 155229.75) | 159.24 (79.87 to 242.59) | 16.01 (8.01 to 24.39) | 56360.75 (31612.73 to 90068.91) | 71.59 (40.37 to 114.12) | 7.13 (4.07 to 11.03) | -3.28 (-3.64 to -2.92) |
| United Arab Emirates | 697.46 (525.80 to 890.70) | 181.26 (135.95 to 232.96) | 17.52 (13.92 to 20.94) | 1891.74 (1385.55 to 2436.25) | 114.23 (85.20 to 145.03) | 13.93 (11.16 to 17.19) | 0.35 (-0.32 to 1.02) |
| United Kingdom | 60981.03 (29634.16 to 97963.47) | 65.26 (32.02 to 104.48) | 9.12 (4.47 to 14.61) | 16550.79 (10757.00 to 22841.32) | 11.30 (7.42 to 15.50) | 2.30 (1.51 to 3.14) | -6.20 (-6.47 to -5.93) |
| United Republic of Tanzania | 2293.25 (1249.99 to 3534.92) | 13.23 (8.23 to 20.19) | 0.79 (0.49 to 1.19) | 4705.65 (2557.40 to 8059.80) | 17.21 (9.30 to 28.63) | 1.19 (0.66 to 1.96) | 1.60 (1.39 to 1.81) |
| United States of America | 136164.96 (58508.09 to 229142.71) | 41.60 (18.07 to 69.71) | 6.17 (2.68 to 10.34) | 50064.11 (24664.35 to 78690.17) | 8.20 (4.12 to 12.77) | 1.35 (0.68 to 2.11) | -5.72 (-6.17 to -5.26) |
| United States Virgin Islands | 15.93 (1.42 to 41.10) | 23.12 (1.58 to 60.87) | 2.73 (0.19 to 7.20) | 18.38 (8.38 to 29.78) | 10.84 (4.96 to 17.40) | 1.77 (0.79 to 2.87) | -2.37 (-2.72 to -2.01) |
| Uruguay | 1581.46 (621.54 to 2980.37) | 41.60 (16.40 to 78.08) | 5.18 (2.05 to 9.75) | 1046.68 (409.60 to 1812.23) | 16.94 (6.69 to 29.20) | 2.45 (0.98 to 4.20) | -3.30 (-3.55 to -3.05) |
| Uzbekistan | 10894.35 (4034.70 to 20430.59) | 84.82 (30.45 to 161.76) | 9.32 (3.36 to 17.81) | 27099.46 (16998.94 to 36518.48) | 127.66 (79.67 to 171.89) | 15.01 (9.40 to 20.43) | 1.81 (1.38 to 2.24) |
| Vanuatu | 11.35 (2.79 to 31.59) | 19.71 (4.93 to 55.91) | 1.34 (0.34 to 3.58) | 38.57 (13.10 to 90.23) | 25.73 (8.79 to 58.94) | 1.87 (0.63 to 3.98) | 1.59 (0.90 to 2.29) |
| Venezuela (Bolivarian Republic of) | 7116.83 (4167.57 to 10605.79) | 71.83 (39.55 to 110.99) | 9.14 (5.02 to 14.15) | 11697.33 (7005.54 to 18164.21) | 41.64 (25.11 to 64.61) | 4.23 (2.59 to 6.03) | -1.87 (-2.02 to -1.71) |
| Viet Nam | 5807.74 (2475.60 to 11360.45) | 14.33 (6.04 to 28.10) | 1.47 (0.62 to 2.85) | 41478.82 (21205.35 to 61975.38) | 48.91 (24.74 to 73.30) | 6.12 (2.97 to 8.98) | 5.13 (4.77 to 5.49) |
| Yemen | 5352.75 (2667.04 to 8568.08) | 77.54 (40.51 to 121.59) | 5.21 (2.70 to 8.02) | 12486.38 (6914.58 to 19332.90) | 94.95 (51.95 to 147.55) | 7.06 (3.90 to 10.79) | 0.83 (0.64 to 1.02) |
| Zambia | 1158.89 (585.13 to 1971.84) | 25.70 (14.69 to 41.55) | 1.29 (0.70 to 2.08) | 2235.74 (1089.62 to 4191.95) | 31.67 (15.54 to 57.28) | 1.48 (0.74 to 2.69) | 0.56 (-0.25 to 1.38) |
| Zimbabwe | 1107.04 (517.30 to 1941.00) | 23.25 (11.50 to 39.87) | 1.69 (0.87 to 2.94) | 1516.44 (795.00 to 2538.08) | 21.60 (11.82 to 36.35) | 0.83 (0.47 to 1.35) | 0.18 (-0.34 to 0.70) |

Table S15. Number and age-standardised rates of deaths attributable to household particulate matter pollution in 1990 and 2021, and estimated annual percentage changes from 1990 to 2021, by country.

| **Cause of deaths** | **1990** |  |  | **2021** |  |  | **1990–2021** |
| --- | --- | --- | --- | --- | --- | --- | --- |
|  | **Number of cases** | **Age-standardised rates per 100 000 people** | **Age-standardised PAF (%)** | **Number of cases** | **Age-standardised rates per 100 000 people** | **Age-standardised PAF (%)** | **EAPC in age-standardised rates (%)** |
| Afghanistan | 28006.87 (19991.35 to 35899.35) | 358.90 (270.13 to 453.34) | 17.94 (14.05 to 21.47) | 25968.16 (20141.63 to 32655.97) | 244.60 (186.80 to 302.48) | 12.04 (9.79 to 14.20) | -1.44 (-1.69 to -1.19) |
| Albania | 3085.76 (2068.57 to 3993.99) | 168.01 (111.60 to 215.00) | 19.71 (13.10 to 25.20) | 717.78 (54.16 to 2614.19) | 17.92 (1.36 to 64.89) | 2.37 (0.18 to 8.48) | -7.89 (-8.60 to -7.18) |
| Algeria | 826.99 (69.32 to 3487.42) | 8.39 (0.70 to 35.26) | 0.80 (0.07 to 3.34) | 40.71 (1.34 to 230.39) | 0.16 (0.01 to 0.88) | 0.02 (0.00 to 0.09) | -14.06 (-14.90 to -13.21) |
| American Samoa | 1.11 (0.00 to 11.12) | 5.72 (0.00 to 57.69) | 0.56 (0.00 to 5.71) | 2.58 (0.04 to 15.06) | 6.29 (0.09 to 36.70) | 0.65 (0.01 to 3.78) | 0.17 (-0.04 to 0.39) |
| Andorra | 0.00 (0.00 to 0.01) | 0.01 (0.00 to 0.03) | 0.00 (0.00 to 0.01) | 0.00 (0.00 to 0.00) | 0.00 (0.00 to 0.00) | 0.00 (0.00 to 0.00) | -7.47 (-7.90 to -7.03) |
| Angola | 15455.80 (9911.77 to 21062.28) | 238.45 (164.90 to 318.10) | 11.20 (8.13 to 14.07) | 8500.01 (3157.97 to 15359.96) | 68.57 (24.45 to 124.00) | 4.04 (1.46 to 7.11) | -4.74 (-5.18 to -4.29) |
| Antigua and Barbuda | 1.35 (0.04 to 7.56) | 2.37 (0.07 to 13.24) | 0.31 (0.01 to 1.71) | 0.15 (0.00 to 1.24) | 0.16 (0.00 to 1.36) | 0.02 (0.00 to 0.18) | -8.23 (-8.53 to -7.93) |
| Argentina | 3012.15 (281.03 to 11605.79) | 10.10 (0.95 to 38.93) | 1.22 (0.11 to 4.67) | 274.52 (1.76 to 2440.46) | 0.48 (0.00 to 4.31) | 0.07 (0.00 to 0.63) | -9.43 (-9.79 to -9.07) |
| Armenia | 1506.77 (638.89 to 2615.99) | 62.07 (26.61 to 106.78) | 6.85 (2.92 to 11.81) | 173.74 (18.66 to 622.98) | 4.11 (0.44 to 14.74) | 0.55 (0.06 to 1.97) | -11.27 (-12.82 to -9.69) |
| Australia | 54.72 (0.01 to 592.86) | 0.29 (0.00 to 3.19) | 0.05 (0.00 to 0.50) | 3.25 (0.00 to 19.59) | 0.01 (0.00 to 0.04) | 0.00 (0.00 to 0.01) | -12.62 (-13.38 to -11.86) |
| Austria | 12.81 (0.01 to 123.21) | 0.10 (0.00 to 1.01) | 0.01 (0.00 to 0.14) | 0.93 (0.00 to 4.94) | 0.00 (0.00 to 0.02) | 0.00 (0.00 to 0.01) | -9.88 (-10.05 to -9.70) |
| Azerbaijan | 4375.21 (924.23 to 9445.77) | 88.92 (18.22 to 192.10) | 8.45 (1.75 to 18.25) | 334.74 (4.88 to 2198.55) | 4.30 (0.06 to 28.38) | 0.41 (0.01 to 2.62) | -12.33 (-14.14 to -10.47) |
| Bahamas | 0.74 (0.00 to 8.22) | 0.49 (0.00 to 5.50) | 0.06 (0.00 to 0.65) | 0.33 (0.00 to 3.05) | 0.09 (0.00 to 0.86) | 0.01 (0.00 to 0.08) | -5.86 (-6.48 to -5.23) |
| Bahrain | 1.63 (0.00 to 16.51) | 1.35 (0.00 to 13.92) | 0.10 (0.00 to 1.02) | 0.14 (0.00 to 0.92) | 0.03 (0.00 to 0.19) | 0.00 (0.00 to 0.02) | -12.98 (-13.35 to -12.61) |
| Bangladesh | 199516.12 (153543.19 to 241708.99) | 280.51 (229.98 to 332.84) | 19.45 (16.10 to 22.86) | 189732.41 (140001.30 to 242132.47) | 156.37 (116.31 to 198.93) | 17.78 (14.14 to 21.02) | -1.95 (-2.28 to -1.61) |
| Barbados | 0.48 (0.01 to 2.82) | 0.16 (0.00 to 0.97) | 0.02 (0.00 to 0.12) | 0.16 (0.00 to 1.15) | 0.03 (0.00 to 0.24) | 0.00 (0.00 to 0.03) | -4.74 (-5.43 to -4.04) |
| Belarus | 711.59 (58.28 to 2954.98) | 5.89 (0.48 to 24.33) | 0.64 (0.05 to 2.67) | 65.14 (0.44 to 567.82) | 0.40 (0.00 to 3.48) | 0.04 (0.00 to 0.33) | -11.31 (-13.13 to -9.46) |
| Belgium | 15.67 (0.01 to 139.19) | 0.10 (0.00 to 0.90) | 0.01 (0.00 to 0.13) | 0.69 (0.00 to 5.10) | 0.00 (0.00 to 0.02) | 0.00 (0.00 to 0.00) | -11.56 (-11.84 to -11.27) |
| Belize | 51.93 (25.69 to 80.40) | 44.67 (23.02 to 68.95) | 6.40 (3.26 to 9.79) | 32.23 (5.49 to 89.78) | 11.74 (2.02 to 32.82) | 1.50 (0.26 to 4.20) | -4.45 (-4.84 to -4.05) |
| Benin | 7648.57 (4871.87 to 10053.02) | 215.72 (150.96 to 268.64) | 13.42 (9.61 to 16.51) | 10622.22 (7738.45 to 13878.07) | 156.35 (118.07 to 197.76) | 11.21 (8.72 to 13.68) | -1.08 (-1.14 to -1.02) |
| Bermuda | 0.25 (0.00 to 2.46) | 0.45 (0.00 to 4.40) | 0.05 (0.00 to 0.52) | 0.02 (0.00 to 0.10) | 0.01 (0.00 to 0.06) | 0.00 (0.00 to 0.01) | -11.43 (-12.17 to -10.69) |
| Bhutan | 706.67 (503.83 to 907.42) | 202.98 (152.15 to 256.83) | 14.73 (11.60 to 17.83) | 139.31 (27.40 to 380.42) | 25.26 (4.95 to 69.01) | 3.28 (0.63 to 8.65) | -7.29 (-7.96 to -6.60) |
| Bolivia (Plurinational State of) | 5024.77 (2766.48 to 7719.49) | 127.08 (73.27 to 185.30) | 9.47 (5.45 to 13.78) | 2125.96 (664.20 to 4182.24) | 27.02 (8.39 to 53.13) | 1.87 (0.56 to 3.70) | -4.65 (-4.97 to -4.32) |
| Bosnia and Herzegovina | 5990.66 (5038.42 to 6934.38) | 176.88 (148.37 to 204.82) | 22.23 (18.66 to 25.58) | 1511.11 (129.80 to 5076.48) | 23.90 (2.06 to 80.02) | 3.16 (0.27 to 10.49) | -7.49 (-7.87 to -7.11) |
| Botswana | 1009.55 (551.32 to 1531.77) | 167.26 (84.79 to 253.45) | 11.32 (5.66 to 16.45) | 187.27 (3.94 to 987.20) | 14.79 (0.31 to 78.78) | 0.78 (0.02 to 4.36) | -7.76 (-8.03 to -7.48) |
| Brazil | 46303.43 (25617.52 to 76976.66) | 54.93 (30.20 to 92.91) | 5.93 (3.27 to 10.00) | 10591.27 (2681.53 to 29999.57) | 4.44 (1.15 to 12.51) | 0.60 (0.15 to 1.69) | -8.07 (-8.34 to -7.81) |
| Brunei Darussalam | 0.17 (0.00 to 0.14) | 0.20 (0.00 to 0.15) | 0.02 (0.00 to 0.02) | 0.08 (0.00 to 0.12) | 0.03 (0.00 to 0.05) | 0.00 (0.00 to 0.01) | -6.65 (-7.39 to -5.91) |
| Bulgaria | 3512.06 (303.70 to 12494.23) | 39.47 (3.43 to 141.37) | 3.22 (0.28 to 11.61) | 797.32 (5.38 to 6854.33) | 5.64 (0.04 to 48.39) | 0.46 (0.00 to 4.00) | -7.61 (-8.75 to -6.45) |
| Burkina Faso | 15379.43 (9141.60 to 20849.30) | 200.05 (130.30 to 259.32) | 10.57 (6.97 to 13.31) | 19187.00 (13580.64 to 25176.55) | 150.79 (110.28 to 193.07) | 9.46 (7.08 to 11.74) | -0.82 (-0.89 to -0.75) |
| Burundi | 10232.29 (7096.18 to 13129.84) | 309.30 (234.42 to 383.83) | 13.66 (10.56 to 16.59) | 10405.15 (7825.24 to 13117.88) | 191.93 (146.30 to 249.39) | 12.61 (9.98 to 14.99) | -1.92 (-2.09 to -1.76) |
| Cabo Verde | 313.17 (239.51 to 391.94) | 113.95 (87.72 to 142.83) | 13.44 (10.59 to 16.29) | 128.75 (36.79 to 273.07) | 31.09 (8.91 to 66.03) | 3.71 (1.08 to 8.00) | -5.05 (-5.43 to -4.67) |
| Cambodia | 20044.06 (12568.77 to 26845.96) | 306.97 (223.25 to 381.73) | 18.59 (13.72 to 22.49) | 18356.69 (13415.99 to 23825.42) | 183.24 (132.95 to 234.55) | 15.66 (11.58 to 19.53) | -1.75 (-1.83 to -1.67) |
| Cameroon | 11219.80 (7328.70 to 15535.52) | 172.44 (117.47 to 230.50) | 11.47 (8.05 to 15.08) | 19336.17 (12870.28 to 27002.55) | 142.01 (94.00 to 199.57) | 8.64 (6.13 to 11.26) | -0.69 (-1.05 to -0.32) |
| Canada | 27.40 (0.00 to 231.79) | 0.09 (0.00 to 0.73) | 0.01 (0.00 to 0.12) | 1.16 (0.00 to 7.02) | 0.00 (0.00 to 0.01) | 0.00 (0.00 to 0.00) | -14.82 (-15.78 to -13.84) |
| Central African Republic | 5801.85 (3822.78 to 7641.21) | 349.57 (256.16 to 438.93) | 14.17 (10.61 to 17.23) | 7042.94 (4871.32 to 9337.72) | 289.01 (212.98 to 373.76) | 11.33 (8.66 to 14.12) | -0.64 (-0.70 to -0.58) |
| Chad | 10779.59 (7025.98 to 14172.79) | 215.74 (148.55 to 274.19) | 12.39 (8.78 to 15.55) | 18818.61 (12318.88 to 25742.98) | 192.83 (138.61 to 254.87) | 11.48 (8.75 to 14.20) | -0.49 (-0.66 to -0.31) |
| Chile | 5248.44 (1921.77 to 8811.93) | 58.51 (21.41 to 98.59) | 7.46 (2.72 to 12.59) | 257.57 (0.43 to 2587.34) | 1.00 (0.00 to 10.11) | 0.19 (0.00 to 1.91) | -12.97 (-13.20 to -12.73) |
| China | 1787388.45 (1397900.25 to 2137741.71) | 292.73 (231.22 to 351.53) | 24.43 (19.85 to 28.46) | 415467.84 (63588.70 to 1346129.38) | 22.76 (3.52 to 73.91) | 3.52 (0.52 to 11.37) | -8.69 (-9.56 to -7.81) |
| Colombia | 7720.19 (2295.31 to 14756.03) | 43.39 (12.68 to 82.78) | 5.42 (1.58 to 10.32) | 1522.92 (60.05 to 7862.38) | 2.73 (0.11 to 14.16) | 0.43 (0.02 to 2.21) | -8.96 (-9.64 to -8.27) |
| Comoros | 726.01 (528.35 to 962.84) | 238.22 (181.60 to 302.48) | 15.19 (11.62 to 18.55) | 664.62 (509.84 to 858.14) | 144.08 (109.41 to 188.75) | 11.76 (9.34 to 14.04) | -1.86 (-2.05 to -1.68) |
| Congo | 2815.12 (1907.15 to 3738.72) | 257.07 (174.99 to 334.39) | 12.70 (8.81 to 16.22) | 2532.68 (1313.20 to 3914.53) | 113.52 (57.57 to 177.04) | 6.72 (3.41 to 10.19) | -3.11 (-3.42 to -2.80) |
| Cook Islands | 1.36 (0.02 to 7.85) | 12.27 (0.12 to 72.08) | 1.11 (0.01 to 6.49) | 0.14 (0.00 to 1.51) | 0.54 (0.00 to 6.08) | 0.08 (0.00 to 0.91) | -9.42 (-10.01 to -8.82) |
| Costa Rica | 475.11 (121.71 to 984.59) | 27.08 (6.75 to 56.24) | 4.55 (1.13 to 9.43) | 66.22 (1.37 to 435.44) | 1.20 (0.02 to 7.92) | 0.21 (0.00 to 1.42) | -10.10 (-10.26 to -9.94) |
| Côte d'Ivoire | 13168.45 (8702.51 to 18458.85) | 188.97 (133.21 to 250.58) | 11.38 (7.86 to 14.88) | 18401.68 (12464.44 to 25587.86) | 141.61 (95.41 to 195.54) | 9.97 (6.91 to 12.99) | -1.06 (-1.34 to -0.79) |
| Croatia | 659.82 (16.45 to 3785.19) | 12.82 (0.32 to 72.79) | 1.28 (0.03 to 7.24) | 71.79 (0.10 to 703.49) | 0.73 (0.00 to 7.18) | 0.11 (0.00 to 1.07) | -11.02 (-11.70 to -10.33) |
| Cuba | 1034.39 (205.12 to 2800.45) | 10.84 (2.15 to 29.45) | 1.49 (0.30 to 4.08) | 368.03 (58.20 to 1360.93) | 1.78 (0.28 to 6.57) | 0.21 (0.03 to 0.79) | -6.95 (-7.57 to -6.33) |
| Cyprus | 3.56 (0.02 to 31.02) | 0.69 (0.00 to 5.95) | 0.06 (0.00 to 0.50) | 0.16 (0.00 to 1.39) | 0.01 (0.00 to 0.08) | 0.00 (0.00 to 0.02) | -13.61 (-14.61 to -12.60) |
| Czechia | 165.78 (0.62 to 1507.12) | 1.25 (0.00 to 11.32) | 0.13 (0.00 to 1.15) | 17.62 (0.00 to 162.51) | 0.08 (0.00 to 0.70) | 0.01 (0.00 to 0.11) | -9.73 (-10.21 to -9.25) |
| Democratic People's Republic of Korea | 32351.65 (23186.34 to 42042.08) | 258.62 (188.81 to 327.27) | 25.54 (19.78 to 30.27) | 63401.09 (49541.61 to 77586.80) | 217.39 (171.79 to 267.21) | 26.45 (22.54 to 30.16) | -0.57 (-0.75 to -0.40) |
| Democratic Republic of the Congo | 53078.05 (35479.35 to 69142.45) | 252.07 (182.39 to 318.57) | 14.02 (10.29 to 17.12) | 72051.30 (53005.91 to 93189.76) | 208.91 (152.95 to 271.89) | 12.72 (9.75 to 15.36) | -0.66 (-0.72 to -0.59) |
| Denmark | 3.69 (0.00 to 31.80) | 0.04 (0.00 to 0.37) | 0.01 (0.00 to 0.05) | 0.26 (0.00 to 1.29) | 0.00 (0.00 to 0.01) | 0.00 (0.00 to 0.00) | -10.09 (-10.37 to -9.81) |
| Djibouti | 209.06 (121.17 to 309.22) | 111.61 (66.86 to 162.82) | 8.07 (5.10 to 11.33) | 255.82 (121.63 to 458.81) | 46.98 (22.89 to 83.46) | 3.24 (1.57 to 5.38) | -3.10 (-3.38 to -2.82) |
| Dominica | 44.77 (17.43 to 71.77) | 76.38 (29.65 to 122.26) | 8.28 (3.19 to 13.22) | 5.17 (0.50 to 18.84) | 7.04 (0.71 to 25.42) | 0.69 (0.07 to 2.49) | -7.75 (-8.11 to -7.38) |
| Dominican Republic | 3302.66 (1742.59 to 4709.96) | 81.20 (42.57 to 116.97) | 10.24 (5.40 to 14.33) | 334.59 (8.49 to 1996.42) | 3.42 (0.09 to 20.43) | 0.47 (0.01 to 2.88) | -9.75 (-10.05 to -9.45) |
| Ecuador | 2009.57 (655.82 to 3919.46) | 36.01 (11.57 to 71.28) | 4.48 (1.43 to 8.86) | 469.26 (40.01 to 1811.33) | 3.17 (0.27 to 12.23) | 0.40 (0.03 to 1.55) | -7.93 (-8.27 to -7.59) |
| Egypt | 21373.73 (8760.55 to 39932.90) | 84.62 (35.99 to 152.76) | 5.23 (2.23 to 9.45) | 177.47 (11.33 to 858.89) | 0.39 (0.02 to 1.87) | 0.03 (0.00 to 0.13) | -17.81 (-18.43 to -17.19) |
| El Salvador | 3132.91 (2192.74 to 4047.74) | 90.90 (64.99 to 115.55) | 10.96 (7.88 to 13.90) | 906.33 (217.56 to 2164.26) | 13.64 (3.29 to 32.68) | 1.72 (0.43 to 3.97) | -6.19 (-6.54 to -5.83) |
| Equatorial Guinea | 731.83 (501.40 to 980.23) | 282.03 (202.78 to 364.35) | 13.27 (10.25 to 16.26) | 14.86 (0.00 to 169.23) | 3.10 (0.00 to 36.60) | 0.19 (0.00 to 2.11) | -18.38 (-20.43 to -16.28) |
| Eritrea | 4484.12 (2751.29 to 5992.55) | 273.05 (192.32 to 355.73) | 10.96 (7.82 to 13.98) | 4694.94 (3152.30 to 6413.04) | 178.32 (126.62 to 236.81) | 10.58 (7.77 to 13.42) | -1.22 (-1.36 to -1.08) |
| Estonia | 497.47 (41.27 to 1700.87) | 25.91 (2.19 to 87.95) | 2.55 (0.22 to 8.62) | 12.78 (0.00 to 138.07) | 0.40 (0.00 to 4.29) | 0.06 (0.00 to 0.66) | -15.22 (-16.23 to -14.19) |
| Eswatini | 634.23 (381.77 to 904.05) | 182.49 (107.00 to 249.74) | 12.00 (7.37 to 15.97) | 432.92 (92.80 to 968.77) | 83.78 (17.17 to 187.79) | 2.85 (0.60 to 5.96) | -1.85 (-2.51 to -1.19) |
| Ethiopia | 102518.95 (67610.96 to 133088.99) | 287.52 (195.84 to 361.33) | 11.50 (7.79 to 14.30) | 68497.20 (53384.59 to 84337.48) | 124.49 (96.20 to 152.36) | 9.05 (7.03 to 10.95) | -3.12 (-3.27 to -2.98) |
| Fiji | 616.00 (343.61 to 856.69) | 193.46 (106.82 to 265.77) | 15.16 (8.56 to 20.22) | 362.55 (56.82 to 883.72) | 57.62 (9.01 to 140.32) | 3.98 (0.63 to 9.67) | -3.93 (-4.30 to -3.55) |
| Finland | 4.74 (0.00 to 42.13) | 0.07 (0.00 to 0.59) | 0.01 (0.00 to 0.08) | 0.24 (0.00 to 1.47) | 0.00 (0.00 to 0.01) | 0.00 (0.00 to 0.00) | -12.71 (-13.14 to -12.27) |
| France | 69.07 (0.03 to 627.77) | 0.08 (0.00 to 0.72) | 0.01 (0.00 to 0.11) | 4.65 (0.00 to 32.37) | 0.00 (0.00 to 0.02) | 0.00 (0.00 to 0.00) | -10.92 (-11.23 to -10.61) |
| Gabon | 183.42 (6.19 to 793.19) | 30.45 (0.97 to 136.16) | 1.94 (0.06 to 8.67) | 46.45 (0.97 to 305.03) | 5.22 (0.11 to 35.06) | 0.34 (0.01 to 2.25) | -6.19 (-6.58 to -5.81) |
| Gambia | 1202.33 (799.27 to 1615.54) | 209.69 (148.23 to 278.11) | 14.30 (10.35 to 17.81) | 2052.33 (1519.32 to 2608.05) | 199.10 (143.68 to 258.69) | 13.04 (10.04 to 16.15) | -0.20 (-0.30 to -0.09) |
| Georgia | 3800.50 (490.05 to 9466.43) | 68.16 (8.91 to 166.93) | 7.01 (0.91 to 17.20) | 1194.84 (135.02 to 3698.76) | 18.93 (2.14 to 58.84) | 1.93 (0.22 to 6.04) | -6.15 (-7.85 to -4.42) |
| Germany | 39.41 (0.01 to 368.82) | 0.03 (0.00 to 0.28) | 0.00 (0.00 to 0.04) | 4.06 (0.00 to 18.48) | 0.00 (0.00 to 0.01) | 0.00 (0.00 to 0.00) | -8.77 (-9.10 to -8.43) |
| Ghana | 16259.44 (11928.13 to 21060.08) | 201.42 (147.70 to 256.11) | 13.65 (10.43 to 16.70) | 18347.27 (10356.23 to 27111.06) | 116.41 (63.71 to 173.83) | 8.34 (4.78 to 12.06) | -1.86 (-2.20 to -1.52) |
| Greece | 174.85 (1.40 to 1295.10) | 1.23 (0.01 to 9.14) | 0.18 (0.00 to 1.36) | 13.43 (0.01 to 138.02) | 0.04 (0.00 to 0.46) | 0.01 (0.00 to 0.09) | -11.08 (-11.96 to -10.19) |
| Greenland | 0.01 (0.00 to 0.05) | 0.04 (0.00 to 0.20) | 0.00 (0.00 to 0.01) | 0.00 (0.00 to 0.00) | 0.00 (0.00 to 0.00) | 0.00 (0.00 to 0.00) | -10.90 (-12.07 to -9.72) |
| Grenada | 47.57 (19.42 to 80.72) | 59.32 (23.95 to 100.14) | 6.28 (2.52 to 10.56) | 1.14 (0.04 to 6.72) | 1.20 (0.05 to 7.07) | 0.11 (0.00 to 0.65) | -12.52 (-13.02 to -12.01) |
| Guam | 0.18 (0.00 to 1.69) | 0.31 (0.00 to 2.99) | 0.04 (0.00 to 0.36) | 0.30 (0.00 to 1.82) | 0.14 (0.00 to 0.88) | 0.02 (0.00 to 0.16) | -1.92 (-2.25 to -1.59) |
| Guatemala | 6479.21 (3656.19 to 9695.13) | 144.00 (82.11 to 212.04) | 10.36 (5.90 to 15.24) | 5317.79 (1844.93 to 9436.70) | 53.68 (18.46 to 94.84) | 5.17 (1.75 to 9.04) | -3.24 (-3.44 to -3.04) |
| Guinea | 12872.80 (8421.89 to 17398.46) | 230.50 (158.20 to 301.80) | 12.97 (8.98 to 16.55) | 13394.94 (9561.88 to 18242.33) | 192.66 (141.28 to 252.58) | 11.66 (8.87 to 14.24) | -0.36 (-0.49 to -0.23) |
| Guinea-Bissau | 1865.65 (1271.62 to 2443.72) | 306.77 (221.14 to 393.60) | 13.66 (10.18 to 16.83) | 1914.73 (1424.78 to 2445.26) | 247.15 (187.16 to 309.10) | 12.34 (9.66 to 15.06) | -0.55 (-0.64 to -0.46) |
| Guyana | 429.17 (260.95 to 597.02) | 103.05 (64.17 to 144.69) | 7.58 (4.63 to 10.61) | 68.57 (13.39 to 178.28) | 12.06 (2.38 to 31.22) | 0.84 (0.16 to 2.32) | -6.74 (-7.36 to -6.11) |
| Haiti | 12402.35 (9352.88 to 15295.86) | 343.39 (270.00 to 415.67) | 18.46 (15.11 to 21.51) | 16145.56 (11899.62 to 20814.37) | 245.89 (184.75 to 318.31) | 14.27 (10.50 to 17.64) | -0.92 (-0.98 to -0.85) |
| Honduras | 2941.58 (2125.48 to 3754.27) | 121.34 (90.38 to 151.55) | 13.40 (10.24 to 16.41) | 5974.18 (3789.29 to 7920.08) | 112.19 (70.32 to 149.16) | 9.05 (5.60 to 12.08) | -0.07 (-0.33 to 0.19) |
| Hungary | 3105.24 (99.57 to 16629.26) | 23.17 (0.76 to 124.01) | 2.13 (0.07 to 11.34) | 409.97 (0.13 to 4739.66) | 1.95 (0.00 to 22.36) | 0.25 (0.00 to 2.89) | -8.67 (-9.06 to -8.28) |
| Iceland | 0.07 (0.00 to 0.80) | 0.02 (0.00 to 0.26) | 0.00 (0.00 to 0.05) | 0.01 (0.00 to 0.03) | 0.00 (0.00 to 0.00) | 0.00 (0.00 to 0.00) | -10.42 (-10.70 to -10.13) |
| India | 1080924.27 (853596.39 to 1284203.25) | 211.24 (170.55 to 247.97) | 15.19 (12.17 to 17.93) | 1047672.89 (665747.32 to 1538527.90) | 100.37 (63.40 to 148.89) | 9.25 (5.91 to 13.68) | -2.29 (-2.53 to -2.04) |
| Indonesia | 156769.04 (120587.56 to 189683.95) | 163.77 (125.83 to 202.89) | 13.41 (10.22 to 16.41) | 88747.22 (31381.90 to 184276.95) | 47.68 (16.85 to 97.90) | 4.28 (1.46 to 8.99) | -3.69 (-4.39 to -2.98) |
| Iran (Islamic Republic of) | 4169.34 (1448.22 to 9432.11) | 16.40 (5.51 to 37.14) | 1.64 (0.55 to 3.70) | 72.96 (3.55 to 426.53) | 0.11 (0.01 to 0.63) | 0.01 (0.00 to 0.08) | -16.83 (-17.59 to -16.05) |
| Iraq | 2142.74 (283.36 to 7380.58) | 21.69 (2.90 to 72.81) | 2.12 (0.28 to 7.01) | 149.85 (2.65 to 994.52) | 0.79 (0.01 to 5.26) | 0.07 (0.00 to 0.48) | -13.24 (-15.12 to -11.31) |
| Ireland | 17.67 (0.16 to 131.28) | 0.45 (0.00 to 3.35) | 0.06 (0.00 to 0.41) | 0.15 (0.00 to 0.34) | 0.00 (0.00 to 0.00) | 0.00 (0.00 to 0.00) | -17.12 (-18.04 to -16.19) |
| Israel | 7.13 (0.03 to 61.22) | 0.16 (0.00 to 1.35) | 0.02 (0.00 to 0.21) | 0.71 (0.00 to 6.10) | 0.01 (0.00 to 0.04) | 0.00 (0.00 to 0.01) | -10.71 (-11.02 to -10.40) |
| Italy | 274.39 (2.28 to 2064.46) | 0.32 (0.00 to 2.40) | 0.05 (0.00 to 0.37) | 27.63 (0.02 to 231.03) | 0.02 (0.00 to 0.13) | 0.00 (0.00 to 0.03) | -9.50 (-10.15 to -8.85) |
| Jamaica | 1291.61 (661.33 to 1864.40) | 67.14 (33.91 to 97.02) | 10.05 (5.08 to 14.61) | 331.39 (67.64 to 899.77) | 10.41 (2.13 to 27.98) | 1.38 (0.28 to 3.60) | -5.35 (-6.11 to -4.58) |
| Japan | 269.55 (0.87 to 2591.55) | 0.18 (0.00 to 1.70) | 0.03 (0.00 to 0.32) | 30.47 (0.00 to 241.54) | 0.01 (0.00 to 0.05) | 0.00 (0.00 to 0.02) | -10.14 (-10.90 to -9.38) |
| Jordan | 25.06 (5.78 to 73.47) | 1.78 (0.42 to 4.98) | 0.21 (0.05 to 0.59) | 2.46 (0.26 to 10.19) | 0.04 (0.00 to 0.18) | 0.01 (0.00 to 0.02) | -13.00 (-13.70 to -12.29) |
| Kazakhstan | 4078.32 (319.56 to 14734.86) | 35.58 (2.76 to 127.02) | 3.40 (0.26 to 12.23) | 238.89 (0.21 to 2409.63) | 1.71 (0.00 to 17.34) | 0.15 (0.00 to 1.47) | -12.78 (-14.91 to -10.61) |
| Kenya | 18675.09 (12626.54 to 24176.54) | 141.98 (94.34 to 183.49) | 11.12 (7.78 to 14.13) | 26272.77 (19781.78 to 33444.37) | 129.17 (94.45 to 167.05) | 8.62 (6.41 to 10.70) | -0.04 (-0.23 to 0.16) |
| Kiribati | 117.22 (95.07 to 143.13) | 312.22 (254.07 to 375.95) | 18.43 (15.42 to 21.41) | 124.37 (95.90 to 160.61) | 201.51 (159.08 to 254.66) | 14.20 (11.84 to 16.92) | -1.49 (-1.71 to -1.27) |
| Kuwait | 0.43 (0.00 to 2.95) | 0.08 (0.00 to 0.53) | 0.01 (0.00 to 0.08) | 0.07 (0.00 to 0.15) | 0.00 (0.00 to 0.01) | 0.00 (0.00 to 0.00) | -10.99 (-11.49 to -10.49) |
| Kyrgyzstan | 4881.53 (2748.94 to 6971.86) | 163.94 (92.55 to 235.13) | 15.22 (8.79 to 21.33) | 3141.66 (1901.92 to 4442.61) | 79.65 (47.78 to 111.58) | 9.19 (5.50 to 12.94) | -2.59 (-3.40 to -1.76) |
| Lao People's Democratic Republic | 10614.91 (7288.07 to 13874.73) | 395.36 (303.10 to 489.82) | 20.21 (16.28 to 23.78) | 6810.05 (3242.86 to 10210.17) | 169.86 (79.81 to 253.90) | 14.84 (7.03 to 21.30) | -2.84 (-3.02 to -2.66) |
| Latvia | 1057.52 (70.04 to 4187.60) | 30.73 (2.04 to 121.10) | 3.01 (0.20 to 11.84) | 79.60 (0.06 to 853.82) | 1.74 (0.00 to 18.61) | 0.21 (0.00 to 2.24) | -11.82 (-13.37 to -10.24) |
| Lebanon | 185.43 (24.94 to 578.84) | 9.41 (1.25 to 29.73) | 0.94 (0.13 to 2.99) | 11.45 (0.30 to 68.75) | 0.18 (0.00 to 1.06) | 0.02 (0.00 to 0.14) | -13.81 (-14.31 to -13.32) |
| Lesotho | 1818.27 (1292.54 to 2317.35) | 179.62 (132.08 to 228.16) | 12.93 (9.97 to 15.57) | 2074.61 (1487.72 to 2774.47) | 202.12 (144.06 to 271.02) | 6.05 (4.51 to 7.89) | 1.26 (0.75 to 1.77) |
| Liberia | 4847.45 (3113.33 to 6534.99) | 237.37 (174.28 to 298.26) | 12.23 (9.20 to 14.85) | 4056.92 (2989.17 to 5434.70) | 179.93 (136.61 to 233.74) | 12.52 (10.10 to 14.96) | -1.08 (-1.19 to -0.98) |
| Libya | 14.57 (0.00 to 141.56) | 0.70 (0.00 to 6.76) | 0.09 (0.00 to 0.93) | 14.00 (0.42 to 91.61) | 0.31 (0.01 to 2.03) | 0.03 (0.00 to 0.20) | -3.06 (-4.85 to -1.24) |
| Lithuania | 498.79 (27.91 to 2280.92) | 11.42 (0.64 to 52.07) | 1.25 (0.07 to 5.68) | 21.40 (0.00 to 217.94) | 0.32 (0.00 to 3.24) | 0.04 (0.00 to 0.40) | -13.43 (-14.56 to -12.28) |
| Luxembourg | 0.24 (0.00 to 1.78) | 0.05 (0.00 to 0.34) | 0.01 (0.00 to 0.05) | 0.01 (0.00 to 0.00) | 0.00 (0.00 to 0.00) | 0.00 (0.00 to 0.00) | -11.39 (-11.58 to -11.20) |
| Madagascar | 20400.24 (14121.92 to 25333.75) | 292.34 (227.98 to 347.99) | 17.05 (13.52 to 20.06) | 26359.25 (20207.45 to 32929.89) | 236.50 (178.21 to 297.37) | 14.70 (11.85 to 17.29) | -0.81 (-0.87 to -0.75) |
| Malawi | 16532.55 (10398.13 to 21885.63) | 231.73 (161.72 to 289.31) | 11.36 (8.05 to 13.99) | 14802.43 (11363.42 to 18734.26) | 186.85 (145.83 to 230.27) | 9.31 (7.43 to 11.07) | -0.92 (-1.10 to -0.73) |
| Malaysia | 728.31 (99.87 to 2431.54) | 8.15 (1.10 to 27.14) | 0.97 (0.13 to 3.21) | 40.36 (0.02 to 443.63) | 0.16 (0.00 to 1.82) | 0.02 (0.00 to 0.21) | -11.77 (-12.17 to -11.36) |
| Maldives | 198.33 (126.68 to 264.48) | 202.81 (121.36 to 269.00) | 17.23 (10.28 to 22.61) | 7.78 (0.13 to 56.37) | 2.80 (0.05 to 20.29) | 0.54 (0.01 to 3.66) | -14.69 (-15.31 to -14.06) |
| Mali | 14841.11 (10956.66 to 18533.48) | 207.43 (159.82 to 251.26) | 10.74 (8.40 to 12.96) | 21419.49 (16056.91 to 27367.41) | 150.15 (115.28 to 188.81) | 8.72 (6.75 to 10.68) | -0.99 (-1.04 to -0.94) |
| Malta | 6.22 (0.20 to 38.24) | 1.58 (0.05 to 9.78) | 0.23 (0.01 to 1.42) | 0.11 (0.00 to 0.93) | 0.01 (0.00 to 0.09) | 0.00 (0.00 to 0.02) | -15.54 (-15.85 to -15.24) |
| Marshall Islands | 31.12 (22.58 to 40.72) | 200.02 (144.96 to 259.37) | 12.71 (9.24 to 16.51) | 41.07 (27.06 to 59.66) | 144.67 (98.40 to 207.36) | 10.40 (7.33 to 13.88) | -0.61 (-0.85 to -0.38) |
| Mauritania | 2363.96 (1627.28 to 3136.85) | 185.54 (127.43 to 245.34) | 12.50 (8.79 to 16.24) | 1951.80 (1143.11 to 2931.30) | 88.97 (51.73 to 136.63) | 8.29 (4.91 to 12.09) | -2.54 (-2.63 to -2.44) |
| Mauritius | 114.61 (21.62 to 305.06) | 18.06 (3.27 to 48.00) | 1.80 (0.33 to 4.79) | 4.81 (0.02 to 45.41) | 0.28 (0.00 to 2.67) | 0.04 (0.00 to 0.34) | -12.63 (-12.95 to -12.32) |
| Mexico | 9224.36 (1693.62 to 23837.16) | 20.68 (3.61 to 56.62) | 2.29 (0.40 to 6.24) | 6327.86 (596.91 to 25352.04) | 5.56 (0.53 to 22.25) | 0.60 (0.06 to 2.41) | -4.51 (-4.65 to -4.37) |
| Micronesia (Federated States of) | 143.67 (110.53 to 182.91) | 305.00 (241.14 to 377.46) | 20.17 (16.33 to 23.59) | 85.10 (55.00 to 124.51) | 140.37 (92.77 to 202.73) | 11.27 (7.90 to 14.96) | -2.59 (-2.64 to -2.54) |
| Monaco | 0.00 (0.00 to 0.00) | 0.01 (0.00 to 0.00) | 0.00 (0.00 to 0.00) | 0.00 (0.00 to 0.00) | 0.00 (0.00 to 0.00) | 0.00 (0.00 to 0.00) | -3.93 (-4.22 to -3.63) |
| Mongolia | 3140.41 (2198.42 to 4118.56) | 261.28 (195.11 to 323.45) | 19.27 (14.59 to 23.37) | 642.80 (52.48 to 2128.20) | 36.07 (3.04 to 118.81) | 3.55 (0.30 to 11.72) | -7.23 (-8.28 to -6.16) |
| Montenegro | 196.73 (12.84 to 695.40) | 34.13 (2.25 to 120.18) | 4.71 (0.31 to 16.62) | 146.73 (2.17 to 903.94) | 17.22 (0.26 to 106.11) | 1.50 (0.02 to 9.38) | -3.96 (-4.82 to -3.09) |
| Morocco | 11870.72 (6614.98 to 17896.57) | 77.27 (41.71 to 115.68) | 7.47 (4.01 to 11.21) | 1829.05 (298.00 to 5679.88) | 6.22 (1.01 to 19.34) | 0.65 (0.11 to 2.03) | -8.33 (-9.00 to -7.65) |
| Mozambique | 22201.02 (15524.67 to 29010.29) | 228.12 (171.36 to 280.09) | 12.03 (9.13 to 14.52) | 26681.46 (20090.11 to 33163.20) | 204.41 (155.38 to 259.16) | 9.65 (7.74 to 11.59) | 0.08 (-0.08 to 0.24) |
| Myanmar | 89444.99 (66248.09 to 113008.79) | 380.29 (297.09 to 471.39) | 21.14 (17.40 to 24.57) | 69213.40 (35982.36 to 101497.92) | 167.10 (87.01 to 244.31) | 14.48 (7.69 to 20.62) | -2.89 (-3.17 to -2.60) |
| Namibia | 1197.79 (660.10 to 1705.87) | 175.61 (90.48 to 250.96) | 12.26 (6.65 to 17.41) | 639.29 (85.22 to 1745.23) | 53.00 (6.80 to 143.88) | 2.70 (0.35 to 7.35) | -4.27 (-4.92 to -3.62) |
| Nauru | 0.44 (0.00 to 3.57) | 10.23 (0.01 to 84.48) | 0.59 (0.00 to 4.75) | 0.45 (0.02 to 2.01) | 8.58 (0.33 to 39.05) | 0.53 (0.02 to 2.30) | -2.07 (-4.76 to 0.71) |
| Nepal | 35815.21 (26171.59 to 45846.77) | 280.41 (215.11 to 350.92) | 18.21 (14.51 to 21.62) | 33873.12 (25664.47 to 43385.53) | 168.27 (127.71 to 213.47) | 14.09 (11.19 to 16.86) | -1.49 (-1.63 to -1.35) |
| Netherlands | 13.59 (0.00 to 142.42) | 0.07 (0.00 to 0.70) | 0.01 (0.00 to 0.11) | 0.71 (0.00 to 3.37) | 0.00 (0.00 to 0.01) | 0.00 (0.00 to 0.00) | -11.82 (-12.36 to -11.28) |
| New Zealand | 6.82 (0.02 to 69.49) | 0.18 (0.00 to 1.82) | 0.03 (0.00 to 0.26) | 0.67 (0.00 to 5.05) | 0.01 (0.00 to 0.06) | 0.00 (0.00 to 0.01) | -10.54 (-11.02 to -10.05) |
| Nicaragua | 2001.81 (1452.58 to 2545.91) | 89.82 (68.57 to 109.40) | 13.61 (10.39 to 16.54) | 1876.39 (1155.63 to 2594.58) | 43.19 (26.62 to 59.38) | 5.32 (3.18 to 7.19) | -2.15 (-2.47 to -1.84) |
| Niger | 16478.61 (7744.29 to 23983.88) | 228.74 (137.92 to 299.34) | 11.35 (6.89 to 14.70) | 20678.38 (13658.32 to 28016.19) | 162.80 (116.60 to 212.88) | 11.18 (8.39 to 13.74) | -1.21 (-1.34 to -1.09) |
| Nigeria | 129846.10 (81748.29 to 177060.06) | 177.86 (122.03 to 235.76) | 10.90 (7.64 to 14.22) | 130995.81 (77466.36 to 195684.17) | 94.38 (54.48 to 139.60) | 7.16 (4.02 to 10.50) | -2.54 (-2.83 to -2.24) |
| Niue | 1.89 (0.47 to 3.53) | 79.87 (19.83 to 150.22) | 7.22 (1.75 to 13.49) | 0.11 (0.00 to 0.58) | 5.63 (0.16 to 29.35) | 0.50 (0.01 to 2.53) | -10.14 (-10.83 to -9.43) |
| North Macedonia | 1266.44 (235.79 to 2911.46) | 77.44 (14.43 to 177.99) | 7.81 (1.45 to 18.20) | 407.17 (16.89 to 2117.93) | 16.93 (0.71 to 87.00) | 1.36 (0.06 to 6.95) | -6.02 (-7.21 to -4.82) |
| Northern Mariana Islands | 0.10 (0.00 to 0.80) | 0.72 (0.00 to 5.40) | 0.08 (0.00 to 0.57) | 0.39 (0.00 to 3.96) | 0.95 (0.00 to 9.81) | 0.10 (0.00 to 0.95) | 3.23 (1.88 to 4.61) |
| Norway | 1.65 (0.00 to 13.98) | 0.02 (0.00 to 0.19) | 0.00 (0.00 to 0.03) | 0.06 (0.00 to 0.11) | 0.00 (0.00 to 0.00) | 0.00 (0.00 to 0.00) | -12.24 (-12.59 to -11.90) |
| Oman | 29.60 (0.07 to 280.59) | 4.17 (0.01 to 39.54) | 0.41 (0.00 to 3.80) | 0.45 (0.00 to 2.91) | 0.03 (0.00 to 0.20) | 0.00 (0.00 to 0.02) | -15.93 (-16.78 to -15.08) |
| Pakistan | 136403.70 (101382.69 to 169133.40) | 180.70 (139.02 to 220.61) | 14.47 (10.99 to 17.59) | 147846.11 (88384.61 to 211248.98) | 119.76 (69.71 to 171.73) | 9.09 (5.35 to 12.84) | -1.56 (-1.86 to -1.26) |
| Palau | 0.02 (0.00 to 0.08) | 0.19 (0.00 to 0.89) | 0.01 (0.00 to 0.07) | 0.01 (0.00 to 0.06) | 0.05 (0.00 to 0.37) | 0.00 (0.00 to 0.03) | -2.95 (-3.50 to -2.40) |
| Palestine | 492.24 (285.24 to 755.66) | 55.69 (32.66 to 85.95) | 4.97 (3.03 to 7.26) | 114.98 (47.45 to 246.60) | 6.07 (2.48 to 12.95) | 0.68 (0.27 to 1.43) | -6.75 (-7.02 to -6.48) |
| Panama | 495.54 (154.59 to 909.49) | 33.48 (9.97 to 62.57) | 5.62 (1.65 to 10.42) | 37.25 (0.08 to 346.03) | 0.83 (0.00 to 7.65) | 0.15 (0.00 to 1.48) | -12.20 (-13.16 to -11.24) |
| Papua New Guinea | 6289.74 (4507.11 to 8209.51) | 344.69 (255.11 to 442.24) | 23.75 (19.30 to 27.71) | 12263.39 (9140.17 to 15463.15) | 268.81 (202.65 to 337.96) | 18.12 (13.20 to 23.07) | -0.70 (-0.77 to -0.63) |
| Paraguay | 2069.46 (1145.74 to 2860.99) | 85.96 (45.21 to 119.71) | 13.42 (7.11 to 18.49) | 1102.19 (128.34 to 3065.39) | 20.09 (2.32 to 55.85) | 2.26 (0.25 to 6.05) | -4.46 (-4.99 to -3.92) |
| Peru | 8994.53 (3698.37 to 15683.93) | 60.50 (25.26 to 103.02) | 7.23 (2.99 to 12.41) | 1948.49 (119.30 to 7983.97) | 5.80 (0.36 to 23.77) | 0.61 (0.04 to 2.59) | -7.48 (-8.07 to -6.89) |
| Philippines | 45964.58 (30995.98 to 59390.86) | 156.71 (111.01 to 199.18) | 15.44 (11.04 to 19.47) | 54954.72 (28393.42 to 88823.77) | 77.58 (39.80 to 125.80) | 6.69 (3.47 to 10.67) | -1.74 (-2.05 to -1.43) |
| Poland | 19882.38 (3889.96 to 49015.79) | 49.63 (9.87 to 121.91) | 5.20 (1.03 to 12.78) | 1078.03 (2.51 to 10342.27) | 1.40 (0.00 to 13.45) | 0.20 (0.00 to 1.89) | -12.16 (-12.54 to -11.78) |
| Portugal | 383.42 (7.69 to 2498.06) | 3.12 (0.06 to 20.17) | 0.38 (0.01 to 2.43) | 7.43 (0.00 to 64.95) | 0.02 (0.00 to 0.22) | 0.01 (0.00 to 0.05) | -15.02 (-15.52 to -14.51) |
| Puerto Rico | 1.07 (0.00 to 8.85) | 0.03 (0.00 to 0.27) | 0.00 (0.00 to 0.04) | 0.19 (0.00 to 1.41) | 0.00 (0.00 to 0.02) | 0.00 (0.00 to 0.00) | -8.35 (-8.96 to -7.74) |
| Qatar | 0.02 (0.00 to 0.04) | 0.02 (0.00 to 0.05) | 0.00 (0.00 to 0.00) | 0.00 (0.00 to 0.00) | 0.00 (0.00 to 0.00) | 0.00 (0.00 to 0.00) | -12.27 (-13.09 to -11.44) |
| Republic of Korea | 676.90 (68.96 to 2864.59) | 3.22 (0.33 to 13.56) | 0.34 (0.03 to 1.43) | 4.24 (0.00 to 33.99) | 0.00 (0.00 to 0.04) | 0.00 (0.00 to 0.01) | -19.55 (-20.62 to -18.47) |
| Republic of Moldova | 6810.87 (4200.86 to 9115.36) | 193.81 (119.61 to 259.15) | 17.16 (10.56 to 22.76) | 1474.23 (518.06 to 2646.46) | 24.84 (8.80 to 44.59) | 2.85 (0.99 to 5.18) | -8.21 (-9.04 to -7.37) |
| Romania | 14635.06 (1889.13 to 38994.67) | 64.93 (8.44 to 172.17) | 6.07 (0.79 to 16.03) | 939.25 (1.71 to 9536.61) | 2.36 (0.00 to 23.88) | 0.27 (0.00 to 2.70) | -12.48 (-13.61 to -11.34) |
| Russian Federation | 8116.40 (702.13 to 45870.58) | 5.33 (0.49 to 29.90) | 0.52 (0.05 to 2.90) | 1278.32 (7.73 to 10734.42) | 0.53 (0.00 to 4.47) | 0.05 (0.00 to 0.43) | -10.35 (-12.45 to -8.19) |
| Rwanda | 14029.63 (9330.18 to 18540.97) | 337.64 (252.98 to 420.21) | 15.01 (11.42 to 18.63) | 9487.18 (6900.04 to 12294.18) | 162.61 (121.79 to 210.80) | 11.62 (9.18 to 14.03) | -3.31 (-3.69 to -2.93) |
| Saint Kitts and Nevis | 4.35 (0.23 to 16.58) | 12.00 (0.64 to 45.89) | 0.99 (0.05 to 3.85) | 0.11 (0.00 to 1.19) | 0.21 (0.00 to 2.28) | 0.02 (0.00 to 0.25) | -12.53 (-13.01 to -12.05) |
| Saint Lucia | 45.09 (12.30 to 88.09) | 58.34 (15.56 to 114.54) | 5.32 (1.41 to 10.60) | 4.21 (0.25 to 21.24) | 1.94 (0.11 to 9.60) | 0.22 (0.01 to 1.11) | -11.47 (-11.84 to -11.08) |
| Saint Vincent and the Grenadines | 40.46 (17.76 to 64.50) | 59.29 (26.22 to 95.00) | 5.97 (2.68 to 9.58) | 3.20 (0.22 to 14.92) | 2.57 (0.18 to 11.92) | 0.28 (0.02 to 1.29) | -10.36 (-10.74 to -9.98) |
| Samoa | 194.96 (145.42 to 235.49) | 250.41 (188.48 to 299.11) | 22.23 (17.15 to 26.13) | 202.56 (107.49 to 285.25) | 158.03 (82.91 to 223.52) | 16.00 (8.47 to 21.96) | -1.47 (-1.54 to -1.40) |
| San Marino | 0.01 (0.00 to 0.03) | 0.01 (0.00 to 0.08) | 0.00 (0.00 to 0.02) | 0.00 (0.00 to 0.00) | 0.00 (0.00 to 0.00) | 0.00 (0.00 to 0.00) | -7.47 (-8.23 to -6.71) |
| Sao Tome and Principe | 162.69 (114.12 to 208.95) | 209.45 (157.93 to 259.10) | 17.06 (12.67 to 20.69) | 107.64 (75.38 to 141.14) | 113.61 (79.22 to 147.60) | 10.77 (7.55 to 13.86) | -2.07 (-2.42 to -1.72) |
| Saudi Arabia | 72.99 (0.00 to 676.69) | 1.19 (0.00 to 11.13) | 0.11 (0.00 to 1.05) | 2.58 (0.00 to 13.79) | 0.02 (0.00 to 0.09) | 0.00 (0.00 to 0.01) | -14.42 (-15.01 to -13.83) |
| Senegal | 8547.49 (6066.83 to 11106.83) | 174.37 (128.26 to 221.69) | 11.63 (8.58 to 14.42) | 12604.67 (9525.53 to 16337.14) | 162.14 (120.88 to 212.96) | 12.12 (9.54 to 14.82) | -0.11 (-0.26 to 0.05) |
| Serbia | 6843.55 (709.94 to 19981.46) | 83.45 (8.80 to 242.13) | 6.98 (0.74 to 20.27) | 2061.80 (51.77 to 10760.07) | 12.02 (0.30 to 62.79) | 1.35 (0.03 to 7.00) | -8.58 (-9.72 to -7.43) |
| Seychelles | 2.02 (0.07 to 10.30) | 3.53 (0.13 to 18.11) | 0.37 (0.01 to 1.92) | 0.07 (0.00 to 0.66) | 0.07 (0.00 to 0.66) | 0.01 (0.00 to 0.08) | -11.98 (-12.29 to -11.66) |
| Sierra Leone | 9038.18 (6002.01 to 12131.53) | 254.08 (188.69 to 318.07) | 13.87 (10.06 to 16.94) | 8679.09 (6293.28 to 11249.33) | 197.38 (148.01 to 251.64) | 13.29 (10.40 to 15.96) | -0.75 (-0.93 to -0.58) |
| Singapore | 32.67 (0.04 to 355.00) | 1.75 (0.00 to 18.82) | 0.25 (0.00 to 2.70) | 0.37 (0.00 to 0.24) | 0.00 (0.00 to 0.00) | 0.00 (0.00 to 0.00) | -18.23 (-19.08 to -17.37) |
| Slovakia | 121.85 (2.75 to 793.30) | 2.15 (0.05 to 14.00) | 0.22 (0.00 to 1.44) | 9.04 (0.00 to 86.25) | 0.10 (0.00 to 0.91) | 0.01 (0.00 to 0.12) | -11.39 (-12.16 to -10.60) |
| Slovenia | 109.66 (0.58 to 934.33) | 4.56 (0.02 to 38.86) | 0.60 (0.00 to 5.11) | 12.90 (0.00 to 123.53) | 0.25 (0.00 to 2.38) | 0.05 (0.00 to 0.51) | -10.64 (-11.15 to -10.13) |
| Solomon Islands | 532.17 (373.70 to 707.08) | 413.89 (316.59 to 525.88) | 25.26 (19.88 to 29.90) | 1000.03 (750.79 to 1301.36) | 337.76 (261.89 to 434.21) | 25.56 (21.41 to 29.42) | -0.63 (-0.70 to -0.56) |
| Somalia | 13035.05 (8423.22 to 17962.57) | 289.48 (204.40 to 371.36) | 12.41 (8.82 to 15.38) | 20444.14 (13887.14 to 27253.36) | 212.64 (155.16 to 275.96) | 8.34 (6.06 to 10.55) | -0.96 (-1.01 to -0.90) |
| South Africa | 12063.11 (5613.73 to 21168.12) | 47.12 (21.33 to 83.02) | 4.39 (1.97 to 7.75) | 6372.16 (1280.70 to 15261.53) | 16.20 (3.28 to 38.19) | 0.99 (0.20 to 2.33) | -4.00 (-4.91 to -3.08) |
| South Sudan | 7308.59 (4012.45 to 10933.46) | 177.97 (95.83 to 261.78) | 9.45 (5.05 to 13.26) | 10541.02 (7150.85 to 14479.71) | 188.68 (134.61 to 254.51) | 9.53 (7.28 to 11.69) | -0.08 (-0.48 to 0.31) |
| Spain | 690.09 (5.01 to 5786.93) | 1.32 (0.01 to 11.05) | 0.20 (0.00 to 1.71) | 20.84 (0.00 to 183.97) | 0.02 (0.00 to 0.15) | 0.00 (0.00 to 0.04) | -13.49 (-14.17 to -12.81) |
| Sri Lanka | 16906.90 (13267.67 to 19944.04) | 195.34 (152.14 to 230.44) | 19.39 (15.14 to 22.65) | 8476.90 (867.71 to 26389.47) | 36.03 (3.68 to 111.04) | 5.45 (0.49 to 16.30) | -4.96 (-5.72 to -4.18) |
| Sudan | 32170.63 (22739.60 to 41152.19) | 265.36 (204.36 to 334.71) | 17.92 (14.06 to 21.33) | 17400.90 (9487.68 to 26133.89) | 93.96 (50.91 to 141.78) | 8.08 (4.64 to 11.79) | -3.74 (-3.97 to -3.51) |
| Suriname | 99.40 (16.83 to 253.97) | 38.68 (6.47 to 100.21) | 4.11 (0.68 to 10.69) | 19.29 (0.57 to 115.07) | 3.29 (0.10 to 19.45) | 0.36 (0.01 to 2.25) | -8.96 (-9.58 to -8.33) |
| Sweden | 6.10 (0.00 to 53.35) | 0.04 (0.00 to 0.32) | 0.01 (0.00 to 0.05) | 0.26 (0.00 to 1.79) | 0.00 (0.00 to 0.01) | 0.00 (0.00 to 0.00) | -12.11 (-12.39 to -11.83) |
| Switzerland | 0.91 (0.00 to 6.84) | 0.01 (0.00 to 0.06) | 0.00 (0.00 to 0.01) | 0.13 (0.00 to 0.30) | 0.00 (0.00 to 0.00) | 0.00 (0.00 to 0.00) | -8.46 (-8.85 to -8.08) |
| Syrian Arab Republic | 1377.55 (622.56 to 2576.27) | 24.91 (11.01 to 47.58) | 2.41 (1.15 to 4.60) | 58.83 (19.33 to 142.68) | 0.58 (0.20 to 1.39) | 0.06 (0.02 to 0.14) | -12.75 (-13.28 to -12.22) |
| Taiwan (Province of China) | 1963.70 (55.32 to 10607.25) | 15.82 (0.44 to 84.94) | 2.12 (0.06 to 11.39) | 70.12 (0.00 to 491.79) | 0.16 (0.00 to 1.14) | 0.04 (0.00 to 0.26) | -13.84 (-14.71 to -12.97) |
| Tajikistan | 6848.60 (4305.51 to 9610.52) | 203.58 (131.49 to 271.45) | 18.70 (12.28 to 24.78) | 5239.24 (3543.66 to 7127.27) | 106.51 (71.81 to 143.23) | 10.28 (7.02 to 13.57) | -2.90 (-3.51 to -2.28) |
| Thailand | 27769.45 (14257.94 to 41351.88) | 90.64 (46.67 to 134.38) | 10.57 (5.47 to 15.67) | 3590.05 (61.99 to 20849.35) | 3.39 (0.06 to 19.75) | 0.54 (0.01 to 3.20) | -10.80 (-11.27 to -10.34) |
| Timor-Leste | 1159.65 (711.83 to 1583.65) | 250.10 (187.24 to 310.53) | 17.84 (13.66 to 21.56) | 1162.83 (528.69 to 1768.58) | 146.94 (63.37 to 225.72) | 13.16 (5.95 to 19.64) | -2.15 (-2.46 to -1.84) |
| Togo | 4391.41 (2983.06 to 5749.29) | 215.42 (160.84 to 269.47) | 14.10 (10.52 to 16.99) | 6835.85 (4935.88 to 9019.39) | 186.61 (140.08 to 242.48) | 12.80 (10.06 to 15.36) | -0.58 (-0.77 to -0.39) |
| Tokelau | 0.02 (0.00 to 0.06) | 1.62 (0.14 to 4.82) | 0.14 (0.01 to 0.41) | 0.00 (0.00 to 0.01) | 0.12 (0.01 to 0.46) | 0.01 (0.00 to 0.04) | -9.37 (-9.72 to -9.03) |
| Tonga | 83.05 (60.20 to 105.24) | 161.28 (117.34 to 201.81) | 17.38 (13.03 to 21.24) | 61.22 (28.56 to 92.34) | 78.51 (36.24 to 118.28) | 9.46 (4.66 to 14.39) | -2.09 (-2.27 to -1.91) |
| Trinidad and Tobago | 7.61 (0.30 to 37.62) | 1.03 (0.04 to 5.07) | 0.10 (0.00 to 0.48) | 0.23 (0.00 to 1.77) | 0.01 (0.00 to 0.10) | 0.00 (0.00 to 0.01) | -16.84 (-18.02 to -15.64) |
| Tunisia | 1346.96 (486.05 to 2613.86) | 29.76 (10.80 to 57.61) | 3.63 (1.29 to 6.95) | 25.14 (1.71 to 123.21) | 0.22 (0.01 to 1.06) | 0.02 (0.00 to 0.12) | -16.57 (-17.09 to -16.04) |
| Türkiye | 9711.95 (1118.73 to 31569.52) | 28.74 (3.29 to 93.79) | 2.76 (0.32 to 9.19) | 276.98 (0.28 to 2406.24) | 0.33 (0.00 to 2.89) | 0.04 (0.00 to 0.39) | -14.05 (-14.63 to -13.47) |
| Turkmenistan | 44.35 (6.97 to 155.07) | 2.09 (0.33 to 7.34) | 0.19 (0.03 to 0.66) | 3.20 (0.01 to 26.59) | 0.09 (0.00 to 0.77) | 0.01 (0.00 to 0.06) | -12.44 (-14.56 to -10.26) |
| Tuvalu | 18.35 (13.52 to 23.43) | 291.48 (223.39 to 361.95) | 17.59 (13.82 to 21.22) | 4.65 (2.55 to 7.39) | 51.44 (28.19 to 81.20) | 4.46 (2.49 to 6.94) | -5.94 (-6.08 to -5.79) |
| Uganda | 22076.63 (15460.89 to 28562.92) | 206.52 (153.32 to 256.89) | 9.68 (7.33 to 12.12) | 25474.36 (18792.61 to 33023.77) | 140.46 (106.38 to 177.67) | 8.76 (6.76 to 10.88) | -1.71 (-1.95 to -1.47) |
| Ukraine | 9571.52 (824.75 to 39407.40) | 15.05 (1.30 to 61.78) | 1.51 (0.13 to 6.23) | 5198.21 (597.14 to 18582.44) | 6.60 (0.76 to 23.57) | 0.65 (0.08 to 2.37) | -5.01 (-6.84 to -3.14) |
| United Arab Emirates | 0.05 (0.00 to 0.00) | 0.01 (0.00 to 0.00) | 0.00 (0.00 to 0.00) | 0.01 (0.00 to 0.03) | 0.00 (0.00 to 0.00) | 0.00 (0.00 to 0.00) | -7.14 (-7.67 to -6.60) |
| United Kingdom | 40.90 (0.30 to 335.92) | 0.04 (0.00 to 0.36) | 0.01 (0.00 to 0.05) | 2.32 (0.00 to 21.35) | 0.00 (0.00 to 0.01) | 0.00 (0.00 to 0.00) | -10.90 (-11.35 to -10.46) |
| United Republic of Tanzania | 38515.45 (22529.29 to 51343.21) | 209.74 (141.60 to 263.97) | 12.54 (8.64 to 15.71) | 42079.15 (32150.38 to 53159.25) | 148.25 (115.83 to 186.76) | 10.24 (8.24 to 12.28) | -1.32 (-1.41 to -1.24) |
| United States of America | 77.66 (0.01 to 647.16) | 0.02 (0.00 to 0.20) | 0.00 (0.00 to 0.03) | 16.40 (0.00 to 97.61) | 0.00 (0.00 to 0.02) | 0.00 (0.00 to 0.00) | -7.31 (-7.56 to -7.06) |
| United States Virgin Islands | 0.21 (0.00 to 2.03) | 0.31 (0.00 to 3.00) | 0.04 (0.00 to 0.36) | 0.02 (0.00 to 0.11) | 0.01 (0.00 to 0.06) | 0.00 (0.00 to 0.01) | -9.30 (-10.46 to -8.13) |
| Uruguay | 432.21 (49.22 to 1329.49) | 11.42 (1.32 to 34.88) | 1.42 (0.16 to 4.32) | 21.64 (0.07 to 194.09) | 0.35 (0.00 to 3.14) | 0.05 (0.00 to 0.45) | -10.58 (-11.22 to -9.93) |
| Uzbekistan | 13132.28 (7433.86 to 19911.46) | 101.29 (58.50 to 150.37) | 11.13 (6.43 to 16.51) | 5276.24 (971.96 to 14034.97) | 25.07 (4.61 to 66.73) | 2.94 (0.53 to 7.76) | -4.93 (-5.91 to -3.94) |
| Vanuatu | 220.02 (174.88 to 272.68) | 376.56 (307.20 to 449.61) | 25.78 (21.94 to 29.54) | 438.79 (347.05 to 540.71) | 295.44 (238.05 to 357.19) | 21.47 (17.15 to 25.45) | -0.91 (-1.00 to -0.83) |
| Venezuela (Bolivarian Republic of) | 157.57 (6.82 to 856.76) | 1.56 (0.07 to 8.35) | 0.20 (0.01 to 1.06) | 179.04 (6.80 to 1030.69) | 0.64 (0.02 to 3.70) | 0.07 (0.00 to 0.36) | -3.90 (-4.61 to -3.18) |
| Viet Nam | 88634.08 (70677.49 to 109379.21) | 217.09 (172.79 to 268.16) | 22.18 (18.80 to 25.69) | 57192.08 (22971.66 to 95916.36) | 68.37 (27.74 to 114.09) | 8.54 (3.34 to 14.09) | -3.83 (-4.27 to -3.40) |
| Yemen | 16724.09 (12066.05 to 22574.02) | 231.40 (168.47 to 299.80) | 15.52 (12.15 to 18.87) | 13261.38 (8671.76 to 18952.67) | 98.40 (63.20 to 143.59) | 7.30 (4.90 to 10.10) | -3.14 (-3.28 to -3.01) |
| Zambia | 9982.27 (6142.62 to 13519.47) | 207.41 (150.91 to 263.56) | 10.40 (7.83 to 12.89) | 11386.40 (7782.64 to 15488.82) | 157.10 (107.15 to 208.39) | 7.34 (5.25 to 9.38) | -1.02 (-1.29 to -0.75) |
| Zimbabwe | 7473.41 (4942.63 to 9861.69) | 153.12 (108.18 to 200.56) | 11.10 (7.94 to 14.01) | 13524.00 (9634.24 to 18055.26) | 190.50 (141.85 to 245.58) | 7.35 (5.54 to 9.14) | 1.66 (1.11 to 2.22) |

Table S16. Number and age-standardised rates of DALYs attributable to household particulate matter pollution in 1990 and 2021, and estimated annual percentage changes from 1990 to 2021, by country.

| **Cause of DALYs** | **1990** |  |  | **2021** |  |  | **1990–2021** |
| --- | --- | --- | --- | --- | --- | --- | --- |
|  | **Number of cases** | **Age-standardised rates per 100 000 people** | **Age-standardised PAF (%)** | **Number of cases** | **Age-standardised rates per 100 000 people** | **Age-standardised PAF (%)** | **EAPC in age-standardised rates (%)** |
| Afghanistan | 1331244.19 (845479.76 to 1832311.03) | 11489.18 (8176.93 to 14837.06) | 12.57 (8.96 to 15.79) | 1198258.25 (887153.34 to 1543839.21) | 6450.94 (5068.19 to 8021.69) | 8.77 (7.19 to 10.38) | -2.02 (-2.29 to -1.75) |
| Albania | 97013.12 (61916.84 to 131227.42) | 3945.70 (2643.05 to 5178.11) | 11.65 (7.65 to 15.57) | 13292.26 (1162.97 to 47895.64) | 345.90 (30.29 to 1228.81) | 1.30 (0.12 to 4.55) | -8.59 (-9.28 to -7.89) |
| Algeria | 36115.37 (3881.13 to 150682.21) | 208.42 (22.46 to 836.75) | 0.50 (0.05 to 2.02) | 1294.51 (85.27 to 6413.10) | 3.82 (0.26 to 18.82) | 0.01 (0.00 to 0.06) | -14.13 (-14.94 to -13.32) |
| American Samoa | 45.83 (0.85 to 390.78) | 168.01 (3.15 to 1405.55) | 0.46 (0.01 to 3.91) | 83.47 (3.50 to 422.50) | 178.98 (8.01 to 905.27) | 0.50 (0.02 to 2.51) | 0.12 (-0.04 to 0.29) |
| Andorra | 0.21 (-0.01 to 0.58) | 0.40 (-0.02 to 1.10) | 0.00 (-0.00 to 0.00) | 0.12 (-0.01 to 0.35) | 0.07 (-0.00 to 0.22) | 0.00 (-0.00 to 0.00) | -5.54 (-5.84 to -5.24) |
| Angola | 1028647.56 (619200.97 to 1467645.55) | 8258.88 (5461.03 to 11115.75) | 8.10 (5.36 to 10.58) | 429608.03 (160362.70 to 783893.55) | 1856.33 (695.35 to 3333.01) | 2.97 (1.11 to 5.21) | -5.49 (-5.94 to -5.04) |
| Antigua and Barbuda | 32.96 (1.66 to 169.72) | 60.12 (3.02 to 310.98) | 0.20 (0.01 to 1.07) | 4.59 (0.08 to 34.45) | 4.72 (0.08 to 35.03) | 0.02 (0.00 to 0.12) | -7.80 (-8.08 to -7.52) |
| Argentina | 80883.87 (8117.78 to 300574.94) | 254.36 (25.59 to 947.96) | 0.75 (0.07 to 2.79) | 6768.35 (204.77 to 52877.42) | 12.93 (0.36 to 103.36) | 0.05 (0.00 to 0.40) | -9.46 (-9.82 to -9.10) |
| Armenia | 45770.30 (19853.05 to 81491.43) | 1585.03 (671.42 to 2769.14) | 4.25 (1.81 to 7.40) | 3628.50 (443.57 to 13045.23) | 91.54 (11.06 to 327.13) | 0.33 (0.04 to 1.16) | -11.55 (-13.01 to -10.06) |
| Australia | 1332.20 (8.60 to 12499.91) | 7.11 (0.05 to 65.72) | 0.03 (0.00 to 0.25) | 141.23 (-5.90 to 462.76) | 0.31 (-0.01 to 1.04) | 0.00 (-0.00 to 0.01) | -10.24 (-10.91 to -9.56) |
| Austria | 272.38 (2.32 to 2281.61) | 2.38 (0.02 to 20.29) | 0.01 (0.00 to 0.07) | 36.43 (-1.52 to 110.94) | 0.19 (-0.01 to 0.58) | 0.00 (-0.00 to 0.00) | -7.89 (-8.18 to -7.60) |
| Azerbaijan | 182247.82 (42252.71 to 407620.79) | 2776.22 (621.27 to 6021.89) | 5.95 (1.34 to 13.03) | 9633.37 (397.35 to 59882.36) | 111.58 (4.53 to 685.51) | 0.30 (0.01 to 1.85) | -13.01 (-14.87 to -11.12) |
| Bahamas | 29.89 (0.18 to 296.24) | 17.14 (0.10 to 156.95) | 0.05 (0.00 to 0.45) | 12.95 (0.04 to 89.79) | 3.43 (0.01 to 23.91) | 0.01 (0.00 to 0.06) | -5.46 (-5.94 to -4.98) |
| Bahrain | 76.83 (0.85 to 593.45) | 41.74 (0.38 to 292.77) | 0.10 (0.00 to 0.74) | 11.77 (-0.51 to 41.44) | 1.58 (-0.07 to 5.76) | 0.00 (-0.00 to 0.02) | -10.92 (-11.13 to -10.70) |
| Bangladesh | 11789456.48 (8016842.89 to 14971642.94) | 10001.07 (7857.52 to 11993.52) | 14.50 (11.24 to 17.32) | 5711983.27 (4345495.19 to 7211607.86) | 4234.79 (3234.85 to 5350.70) | 12.11 (9.53 to 14.79) | -2.73 (-2.95 to -2.50) |
| Barbados | 11.00 (0.44 to 63.28) | 4.09 (0.16 to 23.53) | 0.01 (0.00 to 0.08) | 3.80 (0.15 to 26.79) | 0.88 (0.03 to 6.32) | 0.00 (0.00 to 0.02) | -4.58 (-5.25 to -3.90) |
| Belarus | 15050.89 (1401.19 to 61383.91) | 124.98 (11.67 to 506.90) | 0.35 (0.03 to 1.47) | 1302.07 (30.65 to 10818.75) | 8.29 (0.19 to 68.98) | 0.02 (0.00 to 0.19) | -11.43 (-13.25 to -9.56) |
| Belgium | 343.08 (2.27 to 2649.01) | 2.34 (0.02 to 18.11) | 0.01 (0.00 to 0.07) | 29.06 (-1.12 to 113.72) | 0.12 (-0.00 to 0.53) | 0.00 (-0.00 to 0.00) | -9.38 (-9.78 to -8.99) |
| Belize | 2236.54 (1008.33 to 3708.00) | 1341.68 (672.83 to 2062.42) | 3.92 (1.93 to 6.05) | 1045.79 (195.36 to 2744.53) | 329.77 (63.76 to 872.00) | 1.02 (0.19 to 2.68) | -4.55 (-4.82 to -4.27) |
| Benin | 483576.44 (288242.69 to 661842.09) | 7529.92 (4976.20 to 9757.08) | 9.51 (6.39 to 12.32) | 591584.65 (419084.12 to 799909.57) | 4726.76 (3517.63 to 6173.22) | 8.37 (6.47 to 10.52) | -1.51 (-1.57 to -1.45) |
| Bermuda | 8.14 (-0.14 to 54.69) | 13.76 (-0.24 to 92.02) | 0.05 (-0.00 to 0.32) | 2.12 (-0.18 to 7.07) | 1.50 (-0.12 to 4.90) | 0.01 (-0.00 to 0.02) | -7.30 (-7.77 to -6.82) |
| Bhutan | 44189.92 (30350.65 to 58449.87) | 7092.68 (5202.23 to 8943.52) | 10.86 (8.25 to 13.21) | 3985.54 (848.59 to 10837.63) | 669.36 (142.42 to 1818.01) | 2.08 (0.41 to 5.40) | -8.16 (-8.85 to -7.48) |
| Bolivia (Plurinational State of) | 268509.18 (130482.92 to 433148.02) | 4105.70 (2246.74 to 6323.73) | 6.72 (3.75 to 10.26) | 64954.92 (21908.74 to 126448.93) | 690.28 (232.96 to 1343.90) | 1.44 (0.47 to 2.88) | -5.43 (-5.72 to -5.13) |
| Bosnia and Herzegovina | 154959.25 (131549.55 to 179096.90) | 4071.44 (3461.41 to 4729.43) | 13.06 (10.95 to 15.31) | 30126.04 (2836.96 to 101933.74) | 506.75 (48.03 to 1700.15) | 1.86 (0.18 to 6.04) | -7.74 (-8.10 to -7.38) |
| Botswana | 49501.61 (28231.24 to 73708.77) | 4733.52 (2547.73 to 7185.34) | 7.89 (4.19 to 11.50) | 7490.82 (372.91 to 35630.49) | 444.72 (25.92 to 2180.22) | 0.67 (0.04 to 3.35) | -7.63 (-7.88 to -7.38) |
| Brazil | 1897836.31 (1143041.05 to 2953034.79) | 1666.87 (970.44 to 2643.27) | 3.88 (2.27 to 6.15) | 296174.14 (82594.56 to 804792.59) | 126.10 (36.33 to 339.14) | 0.38 (0.12 to 0.98) | -8.31 (-8.59 to -8.03) |
| Brunei Darussalam | 9.54 (-0.64 to 13.97) | 9.07 (-0.67 to 14.17) | 0.03 (-0.00 to 0.04) | 4.74 (-0.19 to 9.27) | 1.46 (-0.07 to 3.11) | 0.01 (-0.00 to 0.01) | -6.40 (-6.92 to -5.89) |
| Bulgaria | 77838.88 (6869.70 to 275360.47) | 760.78 (66.61 to 2695.62) | 1.96 (0.17 to 7.04) | 15582.33 (268.81 to 131992.03) | 117.91 (1.90 to 995.85) | 0.31 (0.01 to 2.60) | -7.41 (-8.53 to -6.27) |
| Burkina Faso | 1038443.72 (584003.98 to 1462854.80) | 7622.64 (4712.29 to 10223.23) | 7.97 (4.86 to 10.57) | 1098942.42 (721643.61 to 1490836.23) | 4700.48 (3419.95 to 6132.09) | 7.29 (5.49 to 9.10) | -1.42 (-1.49 to -1.36) |
| Burundi | 567237.02 (364931.05 to 757905.32) | 9721.52 (6981.06 to 12319.13) | 9.54 (6.89 to 12.07) | 516845.95 (363251.19 to 665226.02) | 5301.75 (4085.01 to 6667.67) | 9.11 (7.00 to 10.65) | -2.22 (-2.33 to -2.11) |
| Cabo Verde | 11660.98 (8700.56 to 15286.51) | 3368.60 (2584.97 to 4200.38) | 8.12 (6.24 to 10.02) | 3216.19 (967.17 to 6701.43) | 737.39 (221.30 to 1524.30) | 2.32 (0.71 to 4.95) | -5.69 (-6.09 to -5.28) |
| Cambodia | 1224300.82 (666241.27 to 1743480.57) | 10629.30 (6920.61 to 13981.68) | 14.66 (9.60 to 18.73) | 581701.47 (415065.90 to 769891.82) | 4539.17 (3281.67 to 5882.63) | 10.85 (8.24 to 13.41) | -2.94 (-3.07 to -2.81) |
| Cameroon | 678437.62 (415813.89 to 989615.58) | 5614.32 (3742.28 to 7699.74) | 7.93 (5.36 to 10.69) | 937554.22 (621855.19 to 1348036.83) | 3985.53 (2683.40 to 5509.01) | 6.41 (4.49 to 8.37) | -1.15 (-1.48 to -0.82) |
| Canada | 668.11 (2.42 to 4758.22) | 2.13 (0.01 to 15.36) | 0.01 (0.00 to 0.06) | 53.05 (-1.98 to 192.60) | 0.08 (-0.00 to 0.26) | 0.00 (-0.00 to 0.00) | -12.62 (-13.53 to -11.71) |
| Central African Republic | 360877.34 (218334.69 to 499135.70) | 11656.32 (7951.92 to 15128.67) | 10.41 (7.19 to 13.39) | 377513.75 (247153.81 to 510314.29) | 8596.53 (6104.80 to 11208.46) | 8.77 (6.73 to 10.74) | -0.98 (-1.04 to -0.92) |
| Chad | 690461.14 (424070.17 to 950281.72) | 7899.12 (5140.27 to 10282.27) | 9.00 (5.92 to 11.59) | 1200675.81 (763798.68 to 1675213.63) | 6160.82 (4273.12 to 8311.89) | 8.75 (6.45 to 10.83) | -0.90 (-1.07 to -0.73) |
| Chile | 134800.18 (51085.53 to 223160.78) | 1291.22 (484.88 to 2146.81) | 4.03 (1.52 to 6.90) | 6836.60 (98.33 to 57412.77) | 28.40 (0.41 to 242.17) | 0.12 (0.00 to 1.06) | -12.24 (-12.45 to -12.03) |
| China | 53590416.91 (40745947.58 to 64919086.60) | 6551.36 (5073.76 to 7895.68) | 15.21 (11.91 to 18.06) | 8858337.18 (1494828.12 to 27820522.32) | 466.10 (81.56 to 1455.82) | 2.05 (0.35 to 6.45) | -8.98 (-9.78 to -8.18) |
| Colombia | 300242.34 (93073.30 to 561223.26) | 1209.67 (364.12 to 2287.40) | 3.26 (1.00 to 6.11) | 40140.23 (2997.81 to 185058.77) | 76.16 (5.69 to 346.74) | 0.27 (0.02 to 1.26) | -8.87 (-9.52 to -8.21) |
| Comoros | 45959.19 (31602.06 to 63397.03) | 8428.15 (6244.67 to 11143.46) | 12.00 (8.73 to 15.45) | 26808.82 (20786.75 to 34601.11) | 4419.81 (3507.03 to 5639.27) | 9.46 (7.40 to 11.43) | -2.28 (-2.40 to -2.15) |
| Congo | 127652.89 (84112.05 to 174505.65) | 6950.64 (4701.54 to 9192.56) | 8.39 (5.80 to 10.87) | 92964.45 (50461.63 to 144686.10) | 2833.08 (1479.79 to 4307.28) | 4.71 (2.52 to 7.05) | -3.38 (-3.71 to -3.04) |
| Cook Islands | 47.30 (2.69 to 238.02) | 347.74 (18.46 to 1800.02) | 0.87 (0.05 to 4.49) | 6.85 (-0.17 to 43.03) | 27.47 (-0.62 to 176.61) | 0.10 (-0.00 to 0.65) | -7.62 (-8.29 to -6.96) |
| Costa Rica | 14865.29 (4356.88 to 30045.21) | 682.27 (193.26 to 1379.44) | 2.57 (0.74 to 5.00) | 2076.54 (123.20 to 11402.34) | 39.85 (2.46 to 219.04) | 0.15 (0.01 to 0.87) | -9.24 (-9.37 to -9.11) |
| Côte d'Ivoire | 899756.41 (575272.13 to 1299790.66) | 6358.53 (4314.37 to 8645.93) | 7.98 (5.36 to 10.78) | 996350.13 (657617.10 to 1398998.52) | 4260.32 (2931.31 to 5889.56) | 7.52 (5.18 to 9.93) | -1.39 (-1.68 to -1.11) |
| Croatia | 13774.49 (491.56 to 76920.13) | 253.97 (9.25 to 1410.57) | 0.76 (0.03 to 4.29) | 1404.65 (24.25 to 12500.26) | 15.88 (0.26 to 140.28) | 0.07 (0.00 to 0.57) | -10.71 (-11.38 to -10.03) |
| Cuba | 24186.55 (5030.55 to 66031.09) | 244.49 (50.65 to 665.51) | 0.84 (0.18 to 2.23) | 7830.07 (1308.07 to 28208.17) | 41.46 (6.88 to 147.55) | 0.14 (0.02 to 0.49) | -6.79 (-7.38 to -6.19) |
| Cyprus | 75.07 (1.42 to 627.49) | 11.76 (0.21 to 98.37) | 0.04 (0.00 to 0.30) | 4.48 (-0.01 to 27.66) | 0.24 (-0.00 to 1.52) | 0.00 (-0.00 to 0.01) | -12.45 (-13.36 to -11.54) |
| Czechia | 3540.77 (61.85 to 30950.32) | 27.18 (0.47 to 236.16) | 0.08 (0.00 to 0.70) | 432.77 (0.39 to 2944.71) | 2.02 (0.00 to 13.88) | 0.01 (0.00 to 0.06) | -9.11 (-9.47 to -8.75) |
| Democratic People's Republic of Korea | 947144.00 (672219.09 to 1251521.11) | 5810.95 (4121.91 to 7583.05) | 15.64 (12.03 to 18.63) | 1468642.58 (1146597.53 to 1801341.57) | 4745.61 (3726.23 to 5813.08) | 16.03 (13.66 to 18.41) | -0.67 (-0.85 to -0.49) |
| Democratic Republic of the Congo | 3176248.15 (1887330.62 to 4420265.42) | 7712.44 (5330.17 to 9823.99) | 8.94 (6.16 to 11.40) | 3116539.77 (2282836.99 to 4117538.58) | 5369.44 (4017.87 to 6856.77) | 8.80 (6.93 to 10.72) | -1.17 (-1.27 to -1.06) |
| Denmark | 79.52 (0.49 to 594.35) | 1.01 (0.01 to 7.60) | 0.00 (0.00 to 0.03) | 10.36 (-0.46 to 31.36) | 0.09 (-0.00 to 0.29) | 0.00 (-0.00 to 0.00) | -7.97 (-8.31 to -7.63) |
| Djibouti | 13000.67 (7072.33 to 19167.88) | 3536.39 (2111.39 to 5126.24) | 5.80 (3.50 to 8.15) | 10828.87 (5167.40 to 18697.24) | 1283.38 (619.58 to 2230.18) | 2.48 (1.21 to 4.02) | -3.57 (-3.82 to -3.33) |
| Dominica | 1097.69 (455.32 to 1694.00) | 1727.34 (698.16 to 2725.96) | 5.16 (2.14 to 8.05) | 134.27 (16.39 to 468.39) | 195.38 (22.54 to 681.22) | 0.51 (0.06 to 1.78) | -7.04 (-7.41 to -6.67) |
| Dominican Republic | 157171.65 (82578.15 to 225964.59) | 2439.03 (1290.60 to 3473.66) | 6.20 (3.37 to 8.62) | 10917.65 (524.52 to 64275.53) | 107.70 (5.24 to 632.55) | 0.33 (0.02 to 1.94) | -9.78 (-10.00 to -9.55) |
| Ecuador | 83566.96 (26286.49 to 165423.12) | 999.79 (326.03 to 1929.52) | 2.75 (0.91 to 5.35) | 13457.03 (1530.02 to 47462.01) | 84.36 (9.60 to 298.90) | 0.27 (0.03 to 1.00) | -8.16 (-8.56 to -7.75) |
| Egypt | 978885.67 (379740.94 to 1879557.87) | 2181.47 (903.91 to 4057.93) | 3.76 (1.54 to 6.89) | 5245.10 (389.38 to 24626.06) | 8.43 (0.62 to 39.66) | 0.02 (0.00 to 0.09) | -18.37 (-18.99 to -17.73) |
| El Salvador | 128073.18 (84155.45 to 171959.27) | 2694.22 (1884.37 to 3486.77) | 6.21 (4.27 to 8.06) | 22195.11 (5691.34 to 51100.46) | 355.61 (91.26 to 819.25) | 1.06 (0.27 to 2.39) | -6.55 (-6.89 to -6.22) |
| Equatorial Guinea | 41878.86 (27093.63 to 58386.97) | 9002.31 (6324.04 to 11839.08) | 9.43 (6.66 to 12.01) | 775.85 (8.82 to 6537.44) | 108.18 (1.60 to 909.10) | 0.18 (0.00 to 1.34) | -17.48 (-19.27 to -15.66) |
| Eritrea | 280896.39 (160581.74 to 388819.98) | 8817.10 (5875.62 to 11569.50) | 7.73 (5.30 to 10.04) | 213858.75 (137577.96 to 301069.86) | 4840.78 (3367.30 to 6538.59) | 8.05 (5.93 to 10.11) | -1.74 (-1.90 to -1.58) |
| Estonia | 9987.91 (937.12 to 34058.62) | 519.36 (50.50 to 1763.27) | 1.37 (0.14 to 4.69) | 321.11 (1.46 to 2527.94) | 11.56 (0.05 to 92.59) | 0.05 (0.00 to 0.37) | -14.23 (-15.16 to -13.29) |
| Eswatini | 32959.75 (19349.91 to 49209.10) | 5142.42 (3079.72 to 7207.84) | 8.36 (5.17 to 11.43) | 16757.38 (4061.62 to 37544.27) | 2295.63 (517.18 to 5090.88) | 2.31 (0.53 to 4.79) | -2.00 (-2.65 to -1.34) |
| Ethiopia | 6922962.81 (4341298.56 to 9240907.69) | 10823.11 (7163.67 to 13875.18) | 9.55 (6.31 to 12.10) | 3736340.45 (2941170.59 to 4685840.16) | 4033.52 (3267.26 to 4921.70) | 7.77 (6.27 to 9.21) | -3.55 (-3.68 to -3.42) |
| Fiji | 21940.57 (12884.69 to 30486.41) | 5002.15 (2838.22 to 6882.81) | 10.94 (6.33 to 14.56) | 11278.71 (1916.82 to 26485.82) | 1457.17 (252.66 to 3405.29) | 3.08 (0.56 to 7.03) | -3.89 (-4.24 to -3.54) |
| Finland | 106.11 (0.80 to 870.51) | 1.54 (0.01 to 12.94) | 0.01 (0.00 to 0.04) | 11.42 (-0.45 to 39.59) | 0.09 (-0.00 to 0.30) | 0.00 (-0.00 to 0.00) | -9.75 (-10.27 to -9.23) |
| France | 1549.25 (17.15 to 11358.00) | 1.92 (0.02 to 14.06) | 0.01 (0.00 to 0.06) | 201.01 (-7.05 to 792.97) | 0.14 (-0.00 to 0.56) | 0.00 (-0.00 to 0.00) | -8.52 (-8.89 to -8.14) |
| Gabon | 7666.86 (350.15 to 30954.22) | 867.56 (46.26 to 3681.49) | 1.34 (0.07 to 5.70) | 1694.12 (59.64 to 10712.26) | 138.21 (5.40 to 884.51) | 0.25 (0.01 to 1.56) | -6.31 (-6.68 to -5.94) |
| Gambia | 76723.25 (49582.83 to 103775.63) | 6918.38 (4599.05 to 9295.54) | 10.19 (7.23 to 13.07) | 87625.72 (64476.87 to 112996.15) | 5289.89 (3958.00 to 6690.75) | 9.49 (7.50 to 11.53) | -0.94 (-1.06 to -0.82) |
| Georgia | 96813.73 (14453.16 to 236716.02) | 1741.29 (269.36 to 4107.74) | 4.47 (0.71 to 10.74) | 25169.15 (3639.07 to 76582.26) | 445.83 (64.45 to 1351.91) | 1.31 (0.19 to 3.93) | -6.55 (-8.30 to -4.78) |
| Germany | 827.07 (5.09 to 6622.42) | 0.67 (0.00 to 5.47) | 0.00 (0.00 to 0.02) | 148.41 (-5.84 to 449.00) | 0.07 (-0.00 to 0.22) | 0.00 (-0.00 to 0.00) | -6.84 (-7.18 to -6.51) |
| Ghana | 909768.57 (634156.33 to 1236767.26) | 6263.03 (4622.22 to 8026.05) | 9.27 (6.99 to 11.69) | 756140.52 (440162.70 to 1109452.30) | 3156.01 (1809.24 to 4611.75) | 6.15 (3.66 to 8.75) | -2.21 (-2.55 to -1.88) |
| Greece | 3561.71 (94.43 to 25136.95) | 25.46 (0.68 to 183.11) | 0.10 (0.00 to 0.72) | 294.04 (3.07 to 2447.55) | 1.23 (0.01 to 10.85) | 0.01 (0.00 to 0.05) | -10.07 (-10.97 to -9.17) |
| Greenland | 0.47 (-0.01 to 2.14) | 1.31 (-0.02 to 5.27) | 0.00 (-0.00 to 0.01) | 0.10 (-0.00 to 0.23) | 0.17 (-0.01 to 0.40) | 0.00 (-0.00 to 0.00) | -8.19 (-8.87 to -7.51) |
| Grenada | 1236.03 (513.79 to 2079.74) | 1555.11 (650.99 to 2613.36) | 4.10 (1.74 to 6.81) | 32.63 (1.58 to 187.28) | 31.71 (1.56 to 184.61) | 0.08 (0.00 to 0.47) | -12.42 (-12.99 to -11.84) |
| Guam | 14.86 (-0.95 to 65.09) | 20.94 (-1.51 to 84.24) | 0.07 (-0.01 to 0.28) | 34.74 (-2.91 to 97.78) | 16.53 (-1.35 to 46.47) | 0.06 (-0.00 to 0.17) | -0.32 (-0.50 to -0.13) |
| Guatemala | 385505.86 (216487.08 to 578824.95) | 4336.12 (2466.98 to 6398.92) | 7.34 (4.22 to 10.92) | 172605.86 (65164.86 to 293514.29) | 1457.88 (544.73 to 2468.47) | 3.56 (1.33 to 6.09) | -3.51 (-3.65 to -3.37) |
| Guinea | 833701.61 (489736.18 to 1182451.06) | 9250.76 (5972.59 to 12528.46) | 10.05 (6.49 to 13.41) | 677290.11 (450055.40 to 956863.72) | 5723.12 (4089.66 to 7684.89) | 8.82 (6.83 to 11.03) | -1.34 (-1.44 to -1.23) |
| Guinea-Bissau | 113726.75 (74385.55 to 153916.20) | 10313.93 (7101.62 to 13408.46) | 9.97 (7.01 to 12.71) | 94103.25 (69598.10 to 121426.04) | 6738.06 (5096.34 to 8487.95) | 9.41 (7.48 to 11.49) | -1.28 (-1.40 to -1.17) |
| Guyana | 18240.71 (10312.70 to 26656.82) | 2965.30 (1777.50 to 4126.31) | 5.37 (3.31 to 7.52) | 2300.37 (461.56 to 5932.52) | 348.06 (71.10 to 901.62) | 0.67 (0.14 to 1.79) | -6.72 (-7.28 to -6.16) |
| Haiti | 610463.70 (417270.69 to 795652.61) | 10096.34 (7660.60 to 12430.82) | 11.78 (9.12 to 14.13) | 631020.18 (464312.41 to 799356.72) | 6630.60 (4926.38 to 8502.34) | 10.04 (7.91 to 12.09) | -1.14 (-1.23 to -1.05) |
| Honduras | 144038.90 (96598.71 to 194207.14) | 3489.94 (2565.18 to 4354.57) | 7.99 (5.79 to 10.15) | 161946.49 (105591.71 to 213141.28) | 2467.72 (1593.48 to 3270.17) | 5.93 (3.87 to 7.72) | -0.94 (-1.11 to -0.77) |
| Hungary | 70184.65 (3066.20 to 365704.46) | 528.88 (22.58 to 2746.36) | 1.35 (0.06 to 6.99) | 9347.51 (102.90 to 90086.47) | 48.77 (0.51 to 469.06) | 0.17 (0.00 to 1.67) | -8.45 (-8.79 to -8.10) |
| Iceland | 1.84 (0.00 to 15.92) | 0.64 (0.00 to 5.63) | 0.00 (0.00 to 0.02) | 0.45 (-0.02 to 1.45) | 0.08 (-0.00 to 0.25) | 0.00 (-0.00 to 0.00) | -7.26 (-7.57 to -6.94) |
| India | 56268402.48 (42437799.87 to 69208616.91) | 7261.66 (5740.88 to 8585.29) | 11.36 (8.83 to 13.45) | 33523359.44 (22090870.22 to 48262489.85) | 2966.21 (1971.31 to 4256.39) | 7.03 (4.66 to 9.85) | -2.82 (-3.05 to -2.58) |
| Indonesia | 6790722.12 (5125611.85 to 8462140.05) | 4952.02 (3844.42 to 6012.87) | 9.37 (7.22 to 11.44) | 2694763.50 (1052360.50 to 5441299.49) | 1184.72 (462.53 to 2363.92) | 3.10 (1.16 to 6.04) | -4.32 (-4.96 to -3.67) |
| Iran (Islamic Republic of) | 199576.07 (73246.31 to 450937.29) | 468.45 (168.08 to 1040.53) | 0.99 (0.36 to 2.20) | 2154.54 (185.45 to 11515.90) | 2.94 (0.26 to 15.51) | 0.01 (0.00 to 0.05) | -16.61 (-17.23 to -15.99) |
| Iraq | 99210.01 (14072.92 to 337252.77) | 655.40 (99.40 to 2181.45) | 1.39 (0.21 to 4.70) | 4936.89 (184.82 to 31185.53) | 19.79 (0.78 to 121.75) | 0.05 (0.00 to 0.33) | -13.57 (-15.45 to -11.66) |
| Ireland | 359.75 (8.20 to 2539.47) | 9.14 (0.21 to 64.21) | 0.03 (0.00 to 0.23) | 8.76 (-0.51 to 24.42) | 0.11 (-0.01 to 0.31) | 0.00 (-0.00 to 0.00) | -14.00 (-15.10 to -12.90) |
| Israel | 167.55 (3.35 to 1303.11) | 3.52 (0.07 to 27.41) | 0.01 (0.00 to 0.11) | 25.62 (-0.46 to 137.46) | 0.20 (-0.00 to 1.10) | 0.00 (-0.00 to 0.01) | -8.99 (-9.41 to -8.57) |
| Italy | 6914.98 (247.82 to 41816.93) | 8.73 (0.30 to 53.68) | 0.03 (0.00 to 0.22) | 918.94 (4.60 to 4433.46) | 0.62 (0.00 to 3.10) | 0.00 (0.00 to 0.02) | -8.24 (-8.83 to -7.64) |
| Jamaica | 38397.77 (20642.19 to 55543.92) | 1846.88 (978.89 to 2632.75) | 6.27 (3.31 to 8.93) | 8128.57 (1751.88 to 21408.90) | 289.61 (61.72 to 776.37) | 0.95 (0.20 to 2.50) | -5.38 (-6.09 to -4.67) |
| Japan | 6251.20 (126.52 to 53902.63) | 4.00 (0.09 to 34.64) | 0.02 (0.00 to 0.16) | 946.37 (-7.46 to 4862.63) | 0.25 (-0.00 to 1.35) | 0.00 (-0.00 to 0.01) | -8.38 (-9.08 to -7.67) |
| Jordan | 1174.38 (262.00 to 3456.44) | 47.46 (11.40 to 134.59) | 0.13 (0.03 to 0.39) | 90.15 (10.67 to 360.81) | 1.17 (0.14 to 4.64) | 0.00 (0.00 to 0.02) | -12.82 (-13.44 to -12.19) |
| Kazakhstan | 122055.27 (10925.72 to 440206.13) | 905.85 (83.62 to 3267.12) | 2.12 (0.19 to 7.66) | 6778.76 (78.30 to 56325.38) | 41.29 (0.49 to 342.47) | 0.11 (0.00 to 0.90) | -12.93 (-15.01 to -10.80) |
| Kenya | 1170819.11 (762760.34 to 1585959.81) | 4664.43 (3214.88 to 5967.77) | 7.89 (5.56 to 10.01) | 1078962.63 (837312.08 to 1328049.53) | 3412.41 (2604.56 to 4268.20) | 6.42 (4.96 to 7.78) | -0.70 (-0.92 to -0.48) |
| Kiribati | 5141.04 (3941.92 to 6456.80) | 9016.51 (7330.07 to 10972.50) | 13.32 (11.10 to 15.41) | 4523.60 (3476.25 to 5976.66) | 5365.66 (4178.76 to 6928.30) | 10.27 (8.46 to 12.16) | -1.74 (-1.95 to -1.54) |
| Kuwait | 39.89 (-1.90 to 141.33) | 6.70 (-0.39 to 22.33) | 0.02 (-0.00 to 0.07) | 11.23 (-0.80 to 36.63) | 0.42 (-0.03 to 1.44) | 0.00 (-0.00 to 0.01) | -8.87 (-9.02 to -8.71) |
| Kyrgyzstan | 173987.77 (90940.67 to 268744.95) | 4435.17 (2445.73 to 6509.79) | 9.62 (5.44 to 13.68) | 87392.03 (52176.89 to 124332.74) | 1773.23 (1080.57 to 2500.01) | 5.38 (3.35 to 7.59) | -3.35 (-4.17 to -2.53) |
| Lao People's Democratic Republic | 589491.64 (356762.40 to 834035.97) | 13181.08 (9216.87 to 17069.99) | 14.97 (10.96 to 18.74) | 234224.68 (117246.53 to 350830.51) | 4349.26 (2131.30 to 6485.13) | 10.24 (5.10 to 14.51) | -3.68 (-3.88 to -3.48) |
| Latvia | 21468.22 (1590.22 to 83635.12) | 630.28 (46.21 to 2444.38) | 1.63 (0.12 to 6.47) | 1632.57 (19.70 to 15293.66) | 40.70 (0.46 to 385.48) | 0.13 (0.00 to 1.27) | -11.58 (-13.10 to -10.03) |
| Lebanon | 6341.05 (949.35 to 18747.34) | 253.72 (38.77 to 754.86) | 0.61 (0.09 to 1.87) | 305.73 (19.52 to 1614.99) | 5.12 (0.32 to 27.11) | 0.02 (0.00 to 0.10) | -13.51 (-13.98 to -13.04) |
| Lesotho | 90038.26 (61220.81 to 118795.46) | 5793.13 (4131.30 to 7359.29) | 9.56 (7.02 to 11.64) | 80693.00 (57436.32 to 109160.17) | 5942.06 (4299.59 to 7952.88) | 5.03 (3.71 to 6.50) | 0.75 (0.33 to 1.16) |
| Liberia | 323615.62 (186798.70 to 458186.79) | 9304.63 (6120.98 to 12369.68) | 8.63 (5.84 to 11.42) | 194947.94 (140821.72 to 267451.55) | 5059.07 (3774.20 to 6642.87) | 8.62 (6.91 to 10.44) | -2.15 (-2.37 to -1.93) |
| Libya | 1002.87 (-6.11 to 6115.66) | 42.94 (-1.13 to 205.25) | 0.12 (-0.00 to 0.58) | 461.72 (31.38 to 2811.90) | 8.91 (0.61 to 54.41) | 0.02 (0.00 to 0.14) | -5.53 (-7.03 to -4.01) |
| Lithuania | 10133.86 (696.34 to 45591.06) | 234.38 (16.26 to 1048.58) | 0.66 (0.05 to 2.97) | 471.88 (1.94 to 3819.51) | 7.97 (0.04 to 65.08) | 0.03 (0.00 to 0.22) | -12.92 (-13.96 to -11.87) |
| Luxembourg | 6.45 (-0.09 to 34.73) | 1.23 (-0.02 to 6.59) | 0.00 (-0.00 to 0.02) | 1.08 (-0.08 to 3.18) | 0.10 (-0.01 to 0.28) | 0.00 (-0.00 to 0.00) | -8.10 (-8.36 to -7.84) |
| Madagascar | 1144214.80 (731986.18 to 1500153.67) | 9311.75 (6646.05 to 11417.70) | 11.98 (8.58 to 14.44) | 1233923.85 (928141.80 to 1554733.17) | 6499.25 (5014.49 to 8002.51) | 11.10 (8.88 to 13.04) | -1.20 (-1.25 to -1.15) |
| Malawi | 1131388.68 (664390.89 to 1553470.02) | 8478.14 (5491.23 to 11005.46) | 8.31 (5.30 to 10.67) | 688324.01 (504270.30 to 897661.87) | 5241.97 (4096.50 to 6581.61) | 7.50 (5.99 to 8.97) | -1.77 (-1.92 to -1.61) |
| Malaysia | 24407.44 (3875.22 to 79231.87) | 220.80 (35.81 to 710.90) | 0.66 (0.10 to 2.09) | 1750.01 (7.19 to 11660.13) | 6.47 (0.03 to 42.23) | 0.02 (0.00 to 0.14) | -10.64 (-11.23 to -10.05) |
| Maldives | 9905.94 (6178.50 to 13536.95) | 5680.83 (3556.32 to 7504.86) | 11.64 (7.34 to 15.41) | 239.71 (10.41 to 1563.59) | 72.40 (3.15 to 463.76) | 0.33 (0.02 to 2.07) | -14.86 (-15.41 to -14.30) |
| Mali | 1019295.03 (718757.00 to 1310004.96) | 7939.50 (5938.83 to 9786.02) | 7.99 (6.04 to 9.86) | 1384749.27 (988366.81 to 1799533.84) | 5129.31 (3936.53 to 6473.92) | 7.52 (5.92 to 9.18) | -1.41 (-1.47 to -1.36) |
| Malta | 132.06 (5.63 to 806.86) | 32.90 (1.43 to 201.46) | 0.13 (0.01 to 0.75) | 3.54 (-0.07 to 19.41) | 0.38 (-0.01 to 2.13) | 0.00 (-0.00 to 0.01) | -13.99 (-14.47 to -13.50) |
| Marshall Islands | 1197.09 (850.24 to 1605.77) | 5104.91 (3773.04 to 6640.08) | 9.20 (6.79 to 11.82) | 1472.91 (977.74 to 2182.28) | 3762.44 (2535.10 to 5513.02) | 7.60 (5.45 to 10.19) | -0.58 (-0.81 to -0.35) |
| Mauritania | 122363.63 (83568.61 to 165001.14) | 5679.78 (3975.32 to 7494.05) | 8.92 (6.37 to 11.60) | 78644.94 (47233.66 to 112110.61) | 2366.77 (1424.47 to 3523.79) | 5.92 (3.65 to 8.29) | -2.93 (-3.02 to -2.83) |
| Mauritius | 3588.06 (754.53 to 9018.64) | 463.48 (96.36 to 1175.40) | 1.24 (0.26 to 3.10) | 171.67 (3.56 to 1223.22) | 10.29 (0.23 to 75.16) | 0.03 (0.00 to 0.24) | -11.62 (-11.91 to -11.34) |
| Mexico | 431845.21 (90466.82 to 1052946.80) | 611.98 (122.46 to 1519.09) | 1.56 (0.30 to 4.02) | 178285.71 (25506.15 to 640729.10) | 149.39 (21.75 to 533.13) | 0.41 (0.06 to 1.46) | -4.81 (-4.93 to -4.69) |
| Micronesia (Federated States of) | 5233.97 (3776.50 to 6858.08) | 8020.58 (6188.00 to 10208.61) | 14.54 (11.59 to 17.32) | 2736.54 (1791.16 to 3990.60) | 3542.90 (2351.23 to 5112.68) | 8.03 (5.62 to 10.66) | -2.71 (-2.76 to -2.66) |
| Monaco | 0.17 (-0.01 to 0.29) | 0.24 (-0.01 to 0.39) | 0.00 (-0.00 to 0.00) | 0.07 (-0.00 to 0.13) | 0.07 (-0.00 to 0.13) | 0.00 (-0.00 to 0.00) | -3.74 (-3.91 to -3.58) |
| Mongolia | 145711.10 (78061.32 to 210017.30) | 7601.34 (5289.20 to 9983.53) | 13.42 (9.34 to 17.65) | 19552.73 (1844.46 to 63591.03) | 820.62 (79.89 to 2648.97) | 2.19 (0.21 to 7.13) | -7.92 (-8.91 to -6.92) |
| Montenegro | 4711.78 (348.77 to 16166.73) | 793.95 (59.46 to 2700.20) | 2.79 (0.21 to 9.74) | 2843.58 (77.20 to 17006.11) | 312.92 (8.46 to 1864.74) | 0.96 (0.03 to 5.85) | -4.81 (-5.75 to -3.86) |
| Morocco | 500581.75 (278455.92 to 790610.53) | 2278.14 (1286.78 to 3439.90) | 4.81 (2.74 to 7.36) | 50590.05 (8713.61 to 158119.98) | 152.49 (26.52 to 475.46) | 0.44 (0.08 to 1.35) | -8.91 (-9.59 to -8.23) |
| Mozambique | 1444379.17 (946928.31 to 1977489.93) | 8390.29 (5896.66 to 10922.91) | 8.67 (6.16 to 11.05) | 1351711.43 (982251.69 to 1747976.13) | 5913.88 (4591.84 to 7366.49) | 7.51 (6.12 to 8.94) | -0.73 (-0.88 to -0.59) |
| Myanmar | 4007418.57 (2537143.24 to 5381366.24) | 11913.01 (8444.34 to 15136.78) | 14.98 (11.22 to 18.46) | 2005565.99 (1079834.89 to 2903484.36) | 4203.00 (2257.58 to 6069.73) | 9.71 (5.39 to 13.67) | -3.59 (-3.90 to -3.27) |
| Namibia | 56546.57 (31848.81 to 80735.44) | 4906.31 (2680.94 to 6979.15) | 8.46 (4.84 to 11.78) | 22511.24 (3391.60 to 58173.18) | 1378.80 (199.05 to 3612.00) | 2.09 (0.31 to 5.48) | -4.41 (-5.01 to -3.80) |
| Nauru | 18.60 (0.35 to 137.05) | 301.03 (6.35 to 2247.47) | 0.49 (0.01 to 3.66) | 16.68 (1.00 to 70.44) | 237.53 (15.78 to 1022.60) | 0.42 (0.03 to 1.79) | -2.19 (-4.85 to 0.54) |
| Nepal | 2130539.15 (1411486.60 to 2849520.94) | 9665.49 (7223.92 to 12193.46) | 13.34 (9.81 to 16.42) | 1016029.35 (785616.11 to 1281485.06) | 4207.71 (3278.09 to 5298.65) | 9.79 (7.72 to 11.92) | -2.55 (-2.66 to -2.44) |
| Netherlands | 305.16 (2.11 to 2868.53) | 1.59 (0.01 to 14.75) | 0.01 (0.00 to 0.06) | 26.61 (-1.23 to 78.75) | 0.08 (-0.00 to 0.23) | 0.00 (-0.00 to 0.00) | -9.91 (-10.45 to -9.36) |
| New Zealand | 182.16 (4.09 to 1543.18) | 4.94 (0.12 to 42.14) | 0.02 (0.00 to 0.14) | 24.75 (-0.35 to 124.85) | 0.31 (-0.00 to 1.56) | 0.00 (-0.00 to 0.01) | -9.19 (-9.74 to -8.64) |
| Nicaragua | 115608.90 (76797.48 to 154548.08) | 2983.05 (2190.49 to 3728.54) | 8.07 (5.94 to 10.12) | 59640.96 (37726.81 to 80196.86) | 1170.09 (736.69 to 1567.95) | 3.64 (2.32 to 4.90) | -2.90 (-3.11 to -2.69) |
| Niger | 1260628.58 (548341.90 to 1886456.97) | 9786.09 (5012.59 to 13876.48) | 9.01 (4.58 to 12.67) | 1299280.95 (830208.46 to 1774985.08) | 4986.82 (3500.54 to 6604.69) | 8.38 (6.17 to 10.57) | -2.45 (-2.65 to -2.24) |
| Nigeria | 8477701.03 (5071533.35 to 12292640.31) | 6929.22 (4362.79 to 9482.67) | 8.18 (5.26 to 11.24) | 8434472.51 (5020966.94 to 12788854.49) | 3426.03 (2058.90 to 5050.15) | 5.94 (3.54 to 8.59) | -2.64 (-2.91 to -2.37) |
| Niue | 44.21 (12.05 to 83.03) | 1981.09 (537.68 to 3725.86) | 4.97 (1.33 to 9.17) | 3.19 (0.20 to 15.61) | 169.02 (10.96 to 815.61) | 0.37 (0.02 to 1.80) | -9.72 (-10.44 to -8.99) |
| North Macedonia | 31890.81 (6198.69 to 72814.23) | 1813.57 (353.13 to 4131.23) | 4.86 (0.94 to 11.10) | 8490.33 (436.84 to 43902.14) | 302.20 (15.24 to 1539.92) | 0.86 (0.04 to 4.17) | -6.68 (-7.69 to -5.67) |
| Northern Mariana Islands | 7.13 (-0.41 to 37.14) | 41.06 (-3.16 to 157.77) | 0.12 (-0.01 to 0.47) | 17.23 (0.02 to 118.75) | 35.81 (-0.04 to 237.77) | 0.11 (-0.00 to 0.71) | 1.34 (0.38 to 2.30) |
| Norway | 37.84 (0.11 to 257.14) | 0.57 (0.00 to 3.93) | 0.00 (0.00 to 0.02) | 4.62 (-0.30 to 15.00) | 0.04 (-0.00 to 0.14) | 0.00 (-0.00 to 0.00) | -8.27 (-8.74 to -7.79) |
| Oman | 1573.85 (40.77 to 11820.44) | 157.78 (4.53 to 1022.19) | 0.39 (0.01 to 2.48) | 45.24 (-2.08 to 168.90) | 2.38 (-0.12 to 8.83) | 0.01 (-0.00 to 0.03) | -13.54 (-13.98 to -13.10) |
| Pakistan | 7645120.03 (5437610.89 to 9907484.45) | 6343.94 (4772.55 to 7781.96) | 10.71 (8.08 to 13.12) | 6921589.71 (4201413.75 to 9894371.64) | 3775.44 (2293.14 to 5291.28) | 7.31 (4.54 to 10.20) | -1.82 (-2.08 to -1.56) |
| Palau | 0.60 (0.04 to 2.62) | 5.48 (0.38 to 23.78) | 0.01 (0.00 to 0.05) | 0.31 (0.01 to 1.99) | 1.53 (0.06 to 9.94) | 0.00 (0.00 to 0.03) | -3.04 (-3.55 to -2.52) |
| Palestine | 18762.03 (10780.30 to 29956.62) | 1279.35 (754.09 to 1976.07) | 3.10 (1.89 to 4.66) | 3437.32 (1421.47 to 7319.20) | 129.32 (53.98 to 271.48) | 0.41 (0.17 to 0.84) | -6.96 (-7.20 to -6.72) |
| Panama | 16361.87 (5740.31 to 28766.60) | 902.96 (315.76 to 1604.95) | 3.13 (1.06 to 5.56) | 1552.82 (36.17 to 9464.44) | 35.67 (0.82 to 219.64) | 0.14 (0.00 to 0.92) | -10.83 (-11.57 to -10.08) |
| Papua New Guinea | 306159.17 (198830.49 to 418690.30) | 9635.74 (7068.56 to 12415.99) | 15.98 (12.08 to 19.33) | 515430.12 (374911.21 to 671356.00) | 7029.25 (5293.18 to 8784.70) | 12.94 (9.83 to 15.91) | -0.86 (-0.95 to -0.77) |
| Paraguay | 79600.97 (47457.19 to 114357.99) | 2368.61 (1360.16 to 3237.99) | 7.65 (4.47 to 10.43) | 30550.68 (4697.85 to 79778.25) | 520.78 (79.71 to 1364.15) | 1.51 (0.22 to 3.74) | -4.82 (-5.41 to -4.24) |
| Peru | 457927.60 (186716.77 to 818103.26) | 2150.57 (906.28 to 3733.70) | 4.98 (2.15 to 8.59) | 58288.65 (6685.93 to 212455.26) | 173.02 (19.92 to 628.61) | 0.51 (0.06 to 1.86) | -7.83 (-8.36 to -7.29) |
| Philippines | 2202302.57 (1424136.33 to 2972877.13) | 4366.92 (2989.26 to 5607.29) | 10.05 (7.08 to 13.02) | 1747159.89 (973124.56 to 2726081.29) | 2020.72 (1119.91 to 3166.30) | 4.92 (2.75 to 7.71) | -2.05 (-2.32 to -1.78) |
| Poland | 445112.67 (88128.19 to 1086869.91) | 1092.00 (219.41 to 2646.90) | 3.05 (0.64 to 7.59) | 23357.51 (422.84 to 199206.42) | 33.30 (0.65 to 283.44) | 0.12 (0.00 to 1.11) | -11.88 (-12.20 to -11.56) |
| Portugal | 7755.22 (276.68 to 48747.48) | 62.39 (2.20 to 388.58) | 0.20 (0.01 to 1.24) | 203.40 (0.86 to 1295.26) | 0.82 (0.00 to 5.57) | 0.00 (0.00 to 0.03) | -13.45 (-14.05 to -12.85) |
| Puerto Rico | 33.96 (0.71 to 275.44) | 0.99 (0.02 to 8.03) | 0.00 (0.00 to 0.03) | 7.44 (-0.18 to 35.71) | 0.11 (-0.00 to 0.58) | 0.00 (-0.00 to 0.00) | -6.74 (-7.31 to -6.16) |
| Qatar | 1.88 (-0.11 to 5.39) | 1.77 (-0.13 to 5.69) | 0.00 (-0.00 to 0.01) | 0.63 (-0.05 to 1.75) | 0.07 (-0.01 to 0.22) | 0.00 (-0.00 to 0.00) | -10.55 (-10.78 to -10.32) |
| Republic of Korea | 18742.57 (1899.29 to 80168.11) | 68.19 (6.93 to 286.39) | 0.20 (0.02 to 0.87) | 156.89 (-2.71 to 873.42) | 0.18 (-0.00 to 0.98) | 0.00 (-0.00 to 0.01) | -17.85 (-19.10 to -16.59) |
| Republic of Moldova | 166675.90 (101720.66 to 226636.90) | 4138.27 (2537.29 to 5619.37) | 9.83 (6.21 to 13.26) | 31852.17 (11193.50 to 58139.98) | 575.14 (209.33 to 1066.27) | 1.75 (0.66 to 3.18) | -7.87 (-8.74 to -7.00) |
| Romania | 340313.14 (45744.88 to 904426.32) | 1434.58 (195.85 to 3779.90) | 3.65 (0.51 to 9.67) | 18968.19 (277.26 to 179527.42) | 53.80 (0.80 to 505.93) | 0.17 (0.00 to 1.64) | -12.39 (-13.53 to -11.25) |
| Russian Federation | 186516.09 (19821.14 to 1025685.96) | 117.51 (13.31 to 639.57) | 0.30 (0.03 to 1.60) | 28009.06 (734.57 to 217919.76) | 12.22 (0.32 to 94.47) | 0.03 (0.00 to 0.26) | -10.24 (-12.32 to -8.12) |
| Rwanda | 838721.39 (490941.74 to 1163761.46) | 11071.57 (7547.10 to 14326.81) | 11.21 (7.74 to 14.36) | 389799.75 (276998.48 to 498118.12) | 4268.68 (3145.20 to 5447.34) | 8.42 (6.47 to 10.08) | -4.01 (-4.36 to -3.66) |
| Saint Kitts and Nevis | 110.15 (9.05 to 395.21) | 290.38 (22.50 to 1048.00) | 0.66 (0.05 to 2.43) | 3.88 (0.04 to 34.57) | 6.62 (0.07 to 58.72) | 0.02 (0.00 to 0.18) | -11.80 (-12.34 to -11.25) |
| Saint Lucia | 1282.16 (361.26 to 2451.32) | 1310.49 (370.18 to 2522.53) | 3.37 (0.94 to 6.52) | 114.65 (8.81 to 554.15) | 57.49 (4.20 to 268.92) | 0.17 (0.01 to 0.86) | -10.27 (-10.53 to -10.02) |
| Saint Vincent and the Grenadines | 1154.91 (491.92 to 1870.03) | 1430.12 (623.32 to 2270.09) | 3.72 (1.66 to 5.88) | 85.02 (6.63 to 363.84) | 67.59 (5.43 to 291.89) | 0.20 (0.02 to 0.88) | -10.14 (-10.48 to -9.80) |
| Samoa | 6617.83 (4866.39 to 8581.55) | 6131.34 (4579.98 to 7426.56) | 14.88 (11.51 to 17.67) | 5958.18 (3286.28 to 8493.26) | 3846.16 (2082.05 to 5412.93) | 10.72 (5.92 to 14.47) | -1.46 (-1.53 to -1.38) |
| San Marino | 0.18 (-0.01 to 0.56) | 0.52 (-0.01 to 1.64) | 0.00 (-0.00 to 0.01) | 0.07 (-0.00 to 0.26) | 0.09 (-0.01 to 0.31) | 0.00 (-0.00 to 0.00) | -5.50 (-5.94 to -5.07) |
| Sao Tome and Principe | 7644.42 (4809.57 to 10400.62) | 6336.79 (4422.78 to 8149.09) | 11.29 (7.77 to 14.38) | 3411.25 (2368.77 to 4542.75) | 2696.71 (1909.22 to 3522.62) | 7.35 (5.38 to 9.37) | -2.97 (-3.31 to -2.62) |
| Saudi Arabia | 6524.41 (-200.04 to 31580.58) | 96.10 (-5.32 to 376.33) | 0.22 (-0.01 to 0.85) | 326.08 (-19.01 to 1045.28) | 1.90 (-0.13 to 6.49) | 0.01 (-0.00 to 0.02) | -12.90 (-13.18 to -12.63) |
| Senegal | 512270.27 (337659.54 to 692479.66) | 5796.62 (4168.78 to 7447.38) | 8.22 (5.88 to 10.53) | 518363.79 (392944.99 to 670674.79) | 4385.50 (3393.33 to 5596.67) | 9.03 (7.18 to 11.07) | -0.73 (-0.94 to -0.52) |
| Serbia | 153707.93 (17435.48 to 442724.11) | 1671.97 (194.49 to 4791.59) | 4.37 (0.51 to 12.50) | 38510.74 (1449.92 to 194531.80) | 239.18 (9.10 to 1212.12) | 0.83 (0.03 to 4.41) | -8.47 (-9.58 to -7.34) |
| Seychelles | 61.31 (4.33 to 270.65) | 101.79 (7.13 to 459.39) | 0.29 (0.02 to 1.27) | 3.14 (0.02 to 21.13) | 2.95 (0.01 to 19.81) | 0.01 (0.00 to 0.07) | -10.81 (-11.10 to -10.51) |
| Sierra Leone | 584006.66 (362741.74 to 823346.84) | 9698.46 (6392.39 to 13051.93) | 10.13 (6.73 to 13.44) | 427837.40 (301287.63 to 564313.04) | 5789.87 (4239.09 to 7441.90) | 9.17 (7.09 to 11.31) | -1.72 (-1.85 to -1.59) |
| Singapore | 921.38 (12.08 to 9445.94) | 42.53 (0.57 to 433.02) | 0.16 (0.00 to 1.67) | 27.15 (-1.55 to 71.47) | 0.32 (-0.02 to 0.86) | 0.00 (-0.00 to 0.01) | -15.04 (-16.05 to -14.02) |
| Slovakia | 2746.39 (93.23 to 17740.75) | 48.66 (1.63 to 314.76) | 0.14 (0.00 to 0.90) | 208.07 (1.19 to 1714.97) | 2.28 (0.01 to 18.90) | 0.01 (0.00 to 0.07) | -11.25 (-11.98 to -10.53) |
| Slovenia | 2445.91 (58.02 to 19699.27) | 104.92 (2.54 to 849.66) | 0.35 (0.01 to 3.00) | 352.41 (-0.51 to 2438.61) | 7.84 (0.00 to 56.05) | 0.04 (0.00 to 0.30) | -9.58 (-10.05 to -9.12) |
| Solomon Islands | 22899.19 (15043.64 to 31134.94) | 10571.34 (7702.29 to 13790.82) | 17.80 (13.43 to 21.96) | 34305.75 (25416.69 to 44539.93) | 8254.25 (6282.69 to 10570.60) | 17.65 (14.43 to 20.60) | -0.75 (-0.84 to -0.67) |
| Somalia | 897476.00 (542926.98 to 1280414.12) | 9934.45 (6775.01 to 13361.61) | 9.43 (6.38 to 12.26) | 1304923.18 (838532.96 to 1762852.01) | 6648.02 (4845.79 to 8631.95) | 7.37 (5.41 to 9.16) | -1.25 (-1.33 to -1.17) |
| South Africa | 669845.47 (325603.46 to 1140541.74) | 1866.15 (896.36 to 3191.04) | 3.40 (1.65 to 5.89) | 217543.79 (49174.94 to 527708.13) | 473.25 (108.26 to 1131.57) | 0.75 (0.17 to 1.76) | -4.96 (-5.70 to -4.22) |
| South Sudan | 455173.81 (254706.94 to 703571.41) | 6486.65 (3572.36 to 9626.93) | 7.01 (3.88 to 10.25) | 649647.20 (425260.44 to 908154.06) | 6451.65 (4548.57 to 8654.79) | 7.84 (5.88 to 9.86) | -0.15 (-0.56 to 0.26) |
| Spain | 15641.74 (639.58 to 117741.37) | 31.05 (1.26 to 235.16) | 0.12 (0.01 to 0.88) | 1188.28 (-43.86 to 4517.38) | 1.16 (-0.04 to 4.64) | 0.01 (-0.00 to 0.03) | -10.25 (-10.97 to -9.52) |
| Sri Lanka | 495571.02 (392100.72 to 591213.30) | 4411.80 (3482.84 to 5183.05) | 11.29 (8.88 to 13.31) | 212606.93 (29315.19 to 616990.67) | 843.54 (118.23 to 2411.96) | 3.22 (0.40 to 8.97) | -5.11 (-5.82 to -4.40) |
| Sudan | 1715566.15 (1085292.61 to 2341180.59) | 8293.97 (5985.94 to 10665.24) | 11.46 (8.45 to 14.08) | 644253.48 (345671.29 to 984539.92) | 2402.94 (1332.46 to 3553.14) | 5.48 (3.16 to 7.90) | -4.28 (-4.51 to -4.05) |
| Suriname | 3662.10 (655.72 to 9016.27) | 1165.79 (206.90 to 2821.82) | 2.72 (0.50 to 6.69) | 640.22 (36.72 to 3590.03) | 109.53 (6.13 to 611.63) | 0.29 (0.02 to 1.56) | -8.72 (-9.32 to -8.11) |
| Sweden | 119.39 (0.84 to 940.02) | 0.80 (0.01 to 6.19) | 0.00 (0.00 to 0.03) | 11.30 (-0.42 to 47.09) | 0.05 (-0.00 to 0.21) | 0.00 (-0.00 to 0.00) | -9.33 (-9.61 to -9.04) |
| Switzerland | 24.05 (-0.49 to 124.58) | 0.23 (-0.00 to 1.23) | 0.00 (-0.00 to 0.01) | 8.49 (-0.52 to 28.75) | 0.04 (-0.00 to 0.14) | 0.00 (-0.00 to 0.00) | -5.42 (-5.59 to -5.25) |
| Syrian Arab Republic | 56581.39 (25877.39 to 105126.44) | 643.94 (295.57 to 1203.54) | 1.55 (0.72 to 2.91) | 1546.01 (537.78 to 3627.13) | 12.61 (4.37 to 29.39) | 0.04 (0.01 to 0.08) | -13.10 (-13.65 to -12.55) |
| Taiwan (Province of China) | 51243.99 (1863.46 to 266990.61) | 345.03 (12.92 to 1801.34) | 1.25 (0.05 to 6.47) | 2345.91 (-72.37 to 11818.28) | 5.72 (-0.16 to 30.49) | 0.03 (-0.00 to 0.16) | -12.44 (-13.38 to -11.48) |
| Tajikistan | 320928.56 (173090.72 to 499829.75) | 6028.66 (3727.65 to 8508.81) | 12.55 (7.89 to 17.88) | 198649.89 (128859.17 to 281572.37) | 2654.74 (1828.90 to 3628.79) | 6.79 (4.63 to 9.14) | -3.42 (-3.96 to -2.89) |
| Thailand | 922755.20 (492740.96 to 1326991.67) | 2419.27 (1286.91 to 3517.29) | 6.57 (3.58 to 9.57) | 110394.77 (5983.11 to 533703.89) | 109.63 (6.15 to 540.41) | 0.40 (0.02 to 2.04) | -10.14 (-10.48 to -9.81) |
| Timor-Leste | 77615.12 (42757.77 to 112693.15) | 8652.01 (5930.11 to 11516.55) | 12.75 (8.64 to 16.50) | 39826.01 (20156.15 to 60068.68) | 3729.39 (1770.16 to 5573.31) | 9.02 (4.41 to 13.36) | -3.20 (-3.55 to -2.84) |
| Togo | 281283.46 (182323.81 to 384542.88) | 7076.75 (5002.51 to 9025.79) | 9.80 (7.05 to 12.23) | 305242.48 (212231.26 to 411716.48) | 5116.75 (3749.47 to 6677.66) | 9.18 (7.12 to 11.22) | -1.11 (-1.31 to -0.92) |
| Tokelau | 0.63 (0.09 to 1.73) | 45.15 (6.35 to 125.38) | 0.11 (0.02 to 0.29) | 0.05 (0.00 to 0.19) | 4.12 (0.44 to 14.66) | 0.01 (0.00 to 0.03) | -9.11 (-9.61 to -8.61) |
| Tonga | 2811.49 (1982.85 to 3604.41) | 4081.74 (2993.90 to 5136.99) | 11.43 (8.56 to 14.00) | 1657.78 (819.26 to 2480.40) | 1938.50 (942.72 to 2886.34) | 6.21 (3.15 to 9.31) | -2.21 (-2.38 to -2.05) |
| Trinidad and Tobago | 235.35 (11.19 to 1164.64) | 26.75 (1.32 to 131.51) | 0.07 (0.00 to 0.34) | 7.59 (0.01 to 47.79) | 0.43 (0.00 to 2.79) | 0.00 (0.00 to 0.01) | -15.93 (-17.07 to -14.76) |
| Tunisia | 53583.98 (20985.28 to 101887.46) | 815.13 (318.39 to 1543.93) | 2.24 (0.88 to 4.23) | 667.80 (59.57 to 3075.80) | 5.40 (0.48 to 24.87) | 0.02 (0.00 to 0.08) | -16.73 (-17.25 to -16.21) |
| Türkiye | 385094.40 (50896.89 to 1165390.85) | 835.52 (111.27 to 2628.97) | 1.85 (0.24 to 5.69) | 8139.95 (104.76 to 58660.38) | 9.29 (0.12 to 67.24) | 0.03 (0.00 to 0.24) | -14.32 (-14.89 to -13.74) |
| Turkmenistan | 2080.22 (335.89 to 7965.73) | 61.78 (10.53 to 216.13) | 0.13 (0.02 to 0.46) | 104.31 (2.43 to 773.63) | 2.55 (0.06 to 18.56) | 0.01 (0.00 to 0.04) | -12.43 (-14.50 to -10.31) |
| Tuvalu | 748.67 (484.63 to 1043.87) | 8306.65 (5957.36 to 10804.77) | 13.08 (9.81 to 16.33) | 135.82 (76.04 to 216.70) | 1291.34 (715.26 to 2059.30) | 3.14 (1.80 to 4.90) | -6.27 (-6.39 to -6.15) |
| Uganda | 1383430.90 (915711.34 to 1860302.29) | 6545.47 (4719.06 to 8323.01) | 6.29 (4.60 to 8.06) | 1348220.85 (958027.96 to 1788690.12) | 4015.21 (3051.13 to 5059.17) | 6.56 (5.13 to 8.16) | -1.93 (-2.15 to -1.71) |
| Ukraine | 197955.23 (17962.27 to 807699.42) | 303.90 (28.07 to 1248.05) | 0.81 (0.08 to 3.25) | 96963.70 (11947.22 to 346174.26) | 129.92 (16.05 to 457.92) | 0.36 (0.05 to 1.29) | -5.01 (-6.78 to -3.20) |
| United Arab Emirates | 5.73 (-0.34 to 13.55) | 1.29 (-0.09 to 3.46) | 0.00 (-0.00 to 0.01) | 1.89 (-0.10 to 5.77) | 0.05 (-0.00 to 0.16) | 0.00 (-0.00 to 0.00) | -9.74 (-10.16 to -9.33) |
| United Kingdom | 839.68 (22.53 to 6476.10) | 0.97 (0.03 to 7.48) | 0.00 (0.00 to 0.03) | 72.91 (0.24 to 433.28) | 0.06 (0.00 to 0.37) | 0.00 (0.00 to 0.00) | -9.33 (-9.81 to -8.84) |
| United Republic of Tanzania | 2510654.41 (1302346.10 to 3497981.60) | 7603.19 (4719.57 to 9916.03) | 9.31 (5.71 to 12.25) | 2002838.53 (1415858.66 to 2652786.48) | 4296.79 (3335.22 to 5389.88) | 7.86 (6.30 to 9.53) | -1.95 (-2.02 to -1.88) |
| United States of America | 1956.74 (5.17 to 13769.44) | 0.64 (0.00 to 4.57) | 0.00 (0.00 to 0.02) | 777.19 (-30.61 to 2996.08) | 0.14 (-0.00 to 0.54) | 0.00 (-0.00 to 0.00) | -5.14 (-5.29 to -5.00) |
| United States Virgin Islands | 7.81 (0.11 to 66.58) | 9.15 (0.14 to 73.69) | 0.03 (0.00 to 0.24) | 1.38 (-0.07 to 4.27) | 0.80 (-0.03 to 2.47) | 0.00 (-0.00 to 0.01) | -6.96 (-7.76 to -6.15) |
| Uruguay | 9804.22 (1223.06 to 29683.40) | 270.46 (34.52 to 817.22) | 0.84 (0.11 to 2.55) | 461.37 (8.45 to 3851.16) | 8.77 (0.15 to 73.40) | 0.03 (0.00 to 0.28) | -10.44 (-11.03 to -9.85) |
| Uzbekistan | 537098.34 (271921.07 to 883156.15) | 2872.72 (1604.25 to 4396.40) | 7.08 (3.93 to 10.88) | 157808.68 (31106.49 to 410978.84) | 589.46 (117.03 to 1534.77) | 1.79 (0.34 to 4.67) | -5.53 (-6.49 to -4.56) |
| Vanuatu | 9030.08 (6899.58 to 11518.25) | 9546.32 (7644.78 to 11733.11) | 18.12 (15.18 to 21.03) | 15033.49 (11872.69 to 18674.93) | 7396.42 (5916.15 to 9060.64) | 15.67 (12.78 to 18.22) | -0.98 (-1.08 to -0.88) |
| Venezuela (Bolivarian Republic of) | 6379.02 (327.95 to 33803.90) | 45.54 (2.81 to 238.06) | 0.13 (0.01 to 0.68) | 5161.10 (301.11 to 29456.63) | 18.30 (1.06 to 105.75) | 0.05 (0.00 to 0.27) | -3.78 (-4.43 to -3.13) |
| Viet Nam | 3212386.23 (2455231.85 to 3950561.00) | 5849.26 (4649.09 to 7143.09) | 14.79 (12.13 to 17.17) | 1416096.12 (590136.94 to 2373459.71) | 1532.25 (645.93 to 2571.01) | 5.36 (2.23 to 8.76) | -4.40 (-4.75 to -4.05) |
| Yemen | 998107.62 (657775.25 to 1417645.50) | 7049.62 (5246.98 to 9385.78) | 9.99 (7.61 to 12.70) | 531800.22 (348102.42 to 756496.96) | 2514.59 (1665.62 to 3541.15) | 4.88 (3.36 to 6.55) | -3.66 (-3.78 to -3.55) |
| Zambia | 645994.01 (352630.55 to 922151.63) | 6989.63 (4575.18 to 9096.82) | 7.51 (4.83 to 9.89) | 531909.47 (350561.45 to 744522.72) | 4257.36 (2940.42 to 5699.28) | 5.83 (4.26 to 7.40) | -1.68 (-1.99 to -1.36) |
| Zimbabwe | 399775.74 (251178.28 to 542177.51) | 4437.48 (3026.83 to 5801.43) | 7.44 (5.09 to 9.64) | 623761.58 (417555.12 to 857692.75) | 5514.92 (4019.77 to 7310.57) | 6.39 (4.70 to 8.03) | 1.59 (1.08 to 2.10) |

Table S17. Number and age-standardised rates of deaths attributable to particulate matter pollution in 1990 and 2021, and estimated annual percentage changes from 1990 to 2021, by country.

| **Cause of deaths** | **1990** |  |  | **2021** |  |  | **1990–2021** |
| --- | --- | --- | --- | --- | --- | --- | --- |
|  | **Number of cases** | **Age-standardised rates per 100 000 people** | **Age-standardised PAF (%)** | **Number of cases** | **Age-standardised rates per 100 000 people** | **Age-standardised PAF (%)** | **EAPC in age-standardised rates (%)** |
| Afghanistan | 33050.05 (24682.35 to 41186.88) | 425.79 (331.09 to 525.36) | 21.29 (17.64 to 24.64) | 30471.61 (23818.33 to 37713.89) | 288.53 (226.67 to 352.95) | 14.20 (11.66 to 16.37) | -1.38 (-1.56 to -1.20) |
| Albania | 3914.56 (3113.44 to 4655.42) | 213.18 (171.31 to 249.72) | 25.01 (20.20 to 29.25) | 2645.12 (1725.68 to 3970.84) | 65.81 (42.75 to 98.38) | 8.74 (5.61 to 13.07) | -3.89 (-4.18 to -3.60) |
| Algeria | 11392.59 (8150.03 to 14629.67) | 119.66 (85.33 to 156.54) | 11.44 (8.20 to 14.78) | 21599.63 (14946.98 to 28902.91) | 84.38 (58.49 to 112.67) | 8.82 (6.31 to 11.37) | -1.19 (-1.37 to -1.01) |
| American Samoa | 5.58 (0.20 to 17.81) | 27.58 (0.25 to 91.90) | 2.72 (0.02 to 9.12) | 11.84 (1.47 to 25.74) | 28.53 (3.57 to 61.98) | 2.95 (0.39 to 6.29) | -0.14 (-0.37 to 0.09) |
| Andorra | 16.21 (6.67 to 28.19) | 32.62 (13.48 to 56.52) | 5.90 (2.74 to 9.76) | 12.68 (6.93 to 19.91) | 7.42 (4.03 to 11.67) | 1.93 (1.10 to 2.91) | -4.44 (-4.81 to -4.07) |
| Angola | 17927.72 (12055.19 to 23632.16) | 279.33 (211.92 to 359.03) | 13.12 (10.24 to 15.90) | 16757.26 (11531.55 to 22676.67) | 136.81 (93.13 to 186.49) | 8.05 (5.69 to 10.19) | -2.73 (-2.90 to -2.55) |
| Antigua and Barbuda | 34.55 (9.54 to 67.89) | 60.68 (16.74 to 119.05) | 7.83 (2.14 to 15.22) | 38.22 (15.78 to 63.62) | 41.24 (17.31 to 67.75) | 5.40 (2.28 to 8.88) | -1.35 (-1.58 to -1.13) |
| Argentina | 21308.89 (10801.94 to 32597.98) | 71.04 (35.82 to 109.03) | 8.55 (4.32 to 13.11) | 15547.84 (7957.20 to 24216.68) | 27.34 (14.10 to 42.29) | 4.04 (2.09 to 6.20) | -2.82 (-3.08 to -2.56) |
| Armenia | 3925.96 (2421.32 to 5463.86) | 161.24 (98.24 to 227.11) | 17.79 (10.78 to 25.06) | 4158.03 (2968.81 to 5484.62) | 98.04 (70.12 to 129.04) | 13.07 (9.38 to 16.86) | -2.11 (-2.37 to -1.84) |
| Australia | 3107.08 (149.18 to 8233.33) | 16.58 (0.95 to 43.78) | 2.58 (0.15 to 6.80) | 4250.45 (2579.31 to 6147.94) | 8.32 (5.10 to 11.94) | 2.29 (1.40 to 3.27) | -2.80 (-3.38 to -2.21) |
| Austria | 7976.74 (4360.55 to 12235.99) | 65.36 (35.88 to 99.81) | 9.19 (5.02 to 14.02) | 2922.53 (2099.23 to 3829.59) | 13.60 (9.78 to 17.75) | 3.12 (2.26 to 4.06) | -5.25 (-5.46 to -5.04) |
| Azerbaijan | 8926.52 (4916.95 to 13082.61) | 180.65 (95.23 to 264.64) | 17.17 (9.11 to 25.14) | 7936.36 (4461.10 to 11806.64) | 100.43 (56.99 to 149.11) | 9.59 (5.56 to 14.22) | -2.17 (-2.46 to -1.89) |
| Bahamas | 82.78 (19.07 to 154.80) | 55.09 (11.35 to 104.82) | 6.53 (1.34 to 12.69) | 127.34 (47.07 to 227.32) | 35.36 (13.33 to 62.62) | 3.43 (1.32 to 5.93) | -1.50 (-1.69 to -1.31) |
| Bahrain | 387.53 (318.27 to 446.02) | 317.81 (257.78 to 368.39) | 23.51 (19.33 to 27.08) | 746.92 (597.30 to 906.25) | 150.65 (121.40 to 178.26) | 14.73 (12.28 to 17.07) | -2.78 (-3.20 to -2.36) |
| Bangladesh | 222195.63 (170553.97 to 268036.41) | 314.69 (263.60 to 365.60) | 21.82 (18.41 to 25.11) | 231601.82 (184107.90 to 286874.27) | 190.91 (154.00 to 234.90) | 21.71 (18.59 to 24.64) | -1.67 (-1.95 to -1.40) |
| Barbados | 206.93 (64.33 to 371.67) | 69.55 (22.45 to 124.10) | 8.88 (2.86 to 15.63) | 235.00 (109.24 to 364.62) | 46.84 (22.90 to 72.31) | 6.91 (3.40 to 10.64) | -1.26 (-1.44 to -1.07) |
| Belarus | 18883.94 (10614.43 to 27002.12) | 155.36 (87.67 to 222.71) | 17.03 (9.70 to 24.43) | 11201.27 (7796.87 to 15479.20) | 68.78 (47.90 to 94.95) | 6.67 (4.84 to 9.02) | -3.33 (-3.75 to -2.90) |
| Belgium | 10270.48 (5460.93 to 15738.93) | 66.03 (35.52 to 101.06) | 9.46 (5.08 to 14.50) | 3305.56 (2290.65 to 4396.55) | 12.04 (8.53 to 15.82) | 2.83 (2.01 to 3.71) | -5.38 (-5.69 to -5.06) |
| Belize | 103.52 (68.69 to 145.91) | 90.80 (56.62 to 128.95) | 13.01 (8.10 to 18.50) | 158.67 (88.55 to 236.11) | 57.91 (31.99 to 86.69) | 7.39 (4.29 to 10.81) | -1.73 (-2.16 to -1.31) |
| Benin | 9242.61 (6071.55 to 11890.99) | 263.33 (189.48 to 322.18) | 16.38 (12.14 to 19.62) | 12802.01 (9672.56 to 16272.76) | 190.04 (148.18 to 233.31) | 13.62 (10.93 to 16.08) | -0.96 (-1.05 to -0.86) |
| Bermuda | 16.82 (0.14 to 43.71) | 29.58 (0.33 to 76.74) | 3.47 (0.04 to 8.89) | 10.24 (1.94 to 19.40) | 6.88 (1.33 to 12.91) | 1.31 (0.27 to 2.42) | -5.36 (-5.76 to -4.95) |
| Bhutan | 809.85 (592.00 to 1031.95) | 234.44 (180.57 to 293.65) | 17.02 (13.67 to 20.25) | 568.70 (421.27 to 725.21) | 102.98 (76.08 to 130.38) | 13.40 (10.30 to 16.39) | -2.95 (-3.15 to -2.75) |
| Bolivia (Plurinational State of) | 9017.75 (5717.40 to 12301.93) | 230.38 (163.00 to 300.08) | 17.16 (12.20 to 22.00) | 6002.42 (4013.96 to 8658.51) | 76.15 (51.40 to 108.98) | 5.26 (3.61 to 7.20) | -3.59 (-3.69 to -3.49) |
| Bosnia and Herzegovina | 7142.24 (6245.52 to 8008.17) | 210.29 (182.95 to 237.12) | 26.43 (23.10 to 29.64) | 5753.40 (4219.06 to 7810.39) | 90.99 (66.89 to 122.94) | 12.03 (9.03 to 15.99) | -3.06 (-3.24 to -2.87) |
| Botswana | 1334.70 (935.09 to 1782.10) | 221.91 (150.98 to 297.74) | 15.02 (10.64 to 19.08) | 987.14 (609.74 to 1486.29) | 76.57 (45.61 to 119.19) | 4.04 (2.44 to 6.18) | -3.12 (-3.39 to -2.86) |
| Brazil | 85227.36 (53704.98 to 118915.98) | 101.52 (62.33 to 143.99) | 10.96 (6.72 to 15.49) | 63603.29 (36871.11 to 90408.48) | 26.45 (15.37 to 37.50) | 3.56 (2.08 to 5.06) | -4.30 (-4.42 to -4.18) |
| Brunei Darussalam | 26.96 (2.65 to 64.93) | 30.45 (2.29 to 74.75) | 3.03 (0.23 to 7.57) | 40.29 (8.74 to 75.66) | 15.72 (3.42 to 29.91) | 2.34 (0.54 to 4.59) | -1.49 (-2.23 to -0.75) |
| Bulgaria | 19275.20 (12187.44 to 27165.94) | 215.36 (135.99 to 302.64) | 17.59 (11.05 to 24.62) | 10831.66 (8242.73 to 15679.84) | 76.82 (58.22 to 110.90) | 6.31 (4.83 to 8.98) | -3.73 (-4.11 to -3.34) |
| Burkina Faso | 18003.80 (10958.12 to 24244.58) | 236.58 (153.84 to 300.75) | 12.50 (8.40 to 15.59) | 22912.13 (16184.18 to 29848.82) | 181.71 (136.41 to 226.36) | 11.40 (8.80 to 13.76) | -0.62 (-0.71 to -0.54) |
| Burundi | 11137.06 (7748.66 to 14044.09) | 337.89 (258.95 to 419.09) | 14.93 (11.74 to 17.87) | 11170.69 (8469.88 to 13999.57) | 206.70 (156.88 to 264.95) | 13.58 (10.66 to 16.09) | -1.97 (-2.14 to -1.80) |
| Cabo Verde | 388.12 (304.79 to 473.47) | 141.80 (111.84 to 172.63) | 16.73 (13.57 to 19.71) | 489.00 (350.00 to 619.79) | 117.76 (84.51 to 149.18) | 14.04 (10.61 to 17.45) | -1.03 (-1.55 to -0.52) |
| Cambodia | 22000.34 (13582.11 to 29418.86) | 337.67 (246.45 to 412.35) | 20.46 (15.13 to 24.29) | 21818.70 (16758.93 to 27535.10) | 217.45 (168.30 to 269.66) | 18.58 (14.45 to 22.33) | -1.50 (-1.57 to -1.43) |
| Cameroon | 14295.80 (10118.92 to 19056.32) | 222.48 (164.86 to 280.35) | 14.80 (11.23 to 18.21) | 25238.29 (18809.41 to 33431.74) | 186.62 (141.73 to 246.33) | 11.35 (8.94 to 13.87) | -0.46 (-0.75 to -0.17) |
| Canada | 8321.64 (2535.67 to 15403.19) | 26.14 (8.05 to 48.20) | 4.30 (1.32 to 7.94) | 3262.75 (1239.40 to 5735.05) | 4.16 (1.64 to 7.23) | 0.99 (0.39 to 1.74) | -6.20 (-6.64 to -5.76) |
| Central African Republic | 6372.48 (4309.31 to 8491.19) | 385.47 (286.17 to 477.11) | 15.62 (11.84 to 18.91) | 7722.31 (5328.75 to 10104.97) | 317.43 (233.68 to 405.23) | 12.45 (9.46 to 15.36) | -0.64 (-0.70 to -0.58) |
| Chad | 12603.16 (8274.39 to 16302.90) | 254.11 (178.96 to 318.18) | 14.59 (10.44 to 18.20) | 22802.63 (14928.68 to 30599.33) | 236.59 (171.99 to 303.05) | 14.09 (11.09 to 16.80) | -0.23 (-0.39 to -0.07) |
| Chile | 9338.55 (6552.08 to 12084.88) | 103.82 (71.93 to 134.92) | 13.23 (9.19 to 17.21) | 6915.89 (4703.56 to 9110.39) | 26.84 (18.39 to 35.35) | 5.07 (3.49 to 6.60) | -4.13 (-4.31 to -3.96) |
| China | 2236939.42 (1919875.64 to 2568404.39) | 367.58 (317.05 to 421.27) | 30.68 (26.99 to 33.95) | 2273437.80 (1771096.98 to 2892736.93) | 125.13 (97.59 to 157.87) | 19.40 (15.89 to 23.57) | -3.62 (-3.92 to -3.31) |
| Colombia | 18075.58 (13062.44 to 22519.33) | 102.17 (71.80 to 129.78) | 12.75 (8.93 to 16.27) | 14289.32 (9151.00 to 20795.88) | 25.68 (16.62 to 37.21) | 4.00 (2.58 to 5.65) | -4.73 (-4.89 to -4.56) |
| Comoros | 764.15 (561.49 to 1003.08) | 251.50 (193.80 to 319.33) | 16.03 (12.44 to 19.52) | 704.95 (544.98 to 909.66) | 152.95 (117.59 to 199.12) | 12.49 (9.94 to 14.81) | -1.83 (-2.00 to -1.65) |
| Congo | 3386.17 (2547.23 to 4308.16) | 310.02 (241.03 to 386.13) | 15.32 (12.24 to 18.55) | 4082.76 (3007.20 to 5337.51) | 183.10 (135.30 to 232.02) | 10.85 (8.22 to 13.24) | -2.02 (-2.19 to -1.84) |
| Cook Islands | 4.54 (0.41 to 11.19) | 40.48 (2.50 to 100.81) | 3.66 (0.23 to 9.08) | 3.68 (0.01 to 9.08) | 14.74 (0.10 to 36.14) | 2.20 (0.02 to 5.42) | -3.45 (-3.91 to -2.98) |
| Costa Rica | 1143.12 (701.78 to 1516.66) | 65.40 (39.66 to 87.38) | 10.99 (6.60 to 14.78) | 997.00 (671.21 to 1382.16) | 18.12 (12.33 to 24.91) | 3.22 (2.19 to 4.46) | -4.11 (-4.35 to -3.87) |
| Côte d'Ivoire | 17298.51 (12029.86 to 22675.36) | 253.52 (197.09 to 313.51) | 15.27 (11.90 to 18.53) | 25102.67 (18801.43 to 32172.03) | 195.54 (151.81 to 251.52) | 13.77 (10.91 to 16.58) | -0.79 (-0.98 to -0.60) |
| Croatia | 6739.64 (3835.08 to 9931.06) | 129.78 (74.52 to 190.92) | 12.94 (7.35 to 19.10) | 3796.89 (2915.23 to 4871.71) | 38.99 (29.97 to 49.92) | 5.81 (4.47 to 7.44) | -3.93 (-4.16 to -3.71) |
| Cuba | 7453.78 (3232.50 to 13248.15) | 77.92 (33.96 to 138.23) | 10.74 (4.66 to 19.06) | 9536.76 (4756.56 to 15072.18) | 46.15 (23.10 to 72.92) | 5.58 (2.80 to 8.97) | -1.79 (-2.06 to -1.53) |
| Cyprus | 581.87 (278.02 to 923.13) | 111.40 (53.56 to 177.65) | 9.32 (4.47 to 14.90) | 502.26 (374.41 to 645.78) | 29.19 (21.78 to 37.30) | 5.51 (4.17 to 7.02) | -4.72 (-4.93 to -4.50) |
| Czechia | 17305.73 (9415.14 to 25693.08) | 129.69 (70.81 to 192.23) | 13.18 (7.20 to 19.49) | 7231.14 (5415.89 to 9140.72) | 31.27 (23.51 to 39.56) | 4.98 (3.75 to 6.28) | -4.22 (-4.51 to -3.93) |
| Democratic People's Republic of Korea | 39184.87 (31037.58 to 49019.38) | 312.43 (249.94 to 383.02) | 30.85 (26.72 to 35.00) | 72214.14 (57770.60 to 87870.35) | 247.15 (199.46 to 300.47) | 30.08 (26.22 to 33.87) | -0.69 (-0.86 to -0.52) |
| Democratic Republic of the Congo | 58126.54 (39748.89 to 75519.33) | 277.50 (205.68 to 347.63) | 15.44 (11.66 to 18.53) | 78387.87 (57310.87 to 101923.21) | 227.51 (166.27 to 294.86) | 13.86 (10.74 to 16.62) | -0.67 (-0.71 to -0.63) |
| Denmark | 5593.48 (2690.72 to 9064.43) | 65.65 (31.88 to 106.00) | 8.92 (4.31 to 14.39) | 1531.81 (951.00 to 2148.52) | 11.54 (7.22 to 16.13) | 2.59 (1.62 to 3.62) | -5.91 (-6.17 to -5.65) |
| Djibouti | 317.73 (215.67 to 423.79) | 172.71 (124.49 to 228.35) | 12.49 (9.21 to 15.48) | 663.73 (438.92 to 953.74) | 122.58 (81.66 to 172.00) | 8.45 (5.88 to 11.14) | -1.14 (-1.24 to -1.03) |
| Dominica | 73.58 (48.09 to 99.40) | 125.45 (80.87 to 169.98) | 13.60 (9.01 to 18.48) | 46.46 (21.98 to 73.20) | 62.37 (30.33 to 96.78) | 6.13 (2.89 to 9.56) | -2.09 (-2.46 to -1.71) |
| Dominican Republic | 4577.97 (3333.10 to 5803.04) | 113.42 (80.53 to 144.26) | 14.32 (10.38 to 18.00) | 5354.97 (2304.05 to 8883.65) | 54.77 (23.43 to 91.06) | 7.49 (3.26 to 12.09) | -1.66 (-1.98 to -1.35) |
| Ecuador | 6205.89 (4366.99 to 7931.27) | 112.60 (77.80 to 143.26) | 14.00 (9.68 to 17.77) | 5273.94 (3299.72 to 7559.66) | 35.43 (22.43 to 50.53) | 4.46 (2.92 to 6.14) | -3.91 (-4.33 to -3.49) |
| Egypt | 78912.44 (62649.62 to 97178.37) | 316.48 (256.23 to 377.41) | 19.55 (15.83 to 23.22) | 115832.12 (90096.13 to 144717.99) | 251.77 (196.73 to 311.01) | 17.71 (14.30 to 21.06) | -0.31 (-0.55 to -0.06) |
| El Salvador | 4152.46 (3297.12 to 4944.67) | 121.14 (98.01 to 142.40) | 14.61 (11.92 to 17.04) | 3019.02 (1898.46 to 4151.00) | 45.53 (28.70 to 62.69) | 5.73 (3.75 to 7.77) | -3.15 (-3.32 to -2.98) |
| Equatorial Guinea | 836.35 (574.20 to 1117.28) | 324.00 (238.04 to 409.78) | 15.25 (11.83 to 18.25) | 540.06 (318.92 to 845.94) | 112.91 (64.87 to 174.50) | 6.98 (4.45 to 10.12) | -4.02 (-4.73 to -3.30) |
| Eritrea | 5108.55 (3196.76 to 6734.93) | 311.97 (226.21 to 398.40) | 12.52 (9.08 to 15.63) | 5591.32 (3874.11 to 7576.75) | 212.33 (152.89 to 282.04) | 12.60 (9.41 to 15.61) | -1.12 (-1.20 to -1.04) |
| Estonia | 1877.08 (789.01 to 3066.12) | 97.36 (41.47 to 158.44) | 9.58 (4.11 to 15.57) | 226.59 (70.00 to 435.16) | 7.26 (2.27 to 13.88) | 1.12 (0.35 to 2.18) | -9.57 (-10.18 to -8.96) |
| Eswatini | 810.13 (568.73 to 1060.23) | 234.12 (174.38 to 296.56) | 15.41 (11.78 to 18.59) | 830.83 (485.61 to 1286.44) | 158.84 (92.56 to 239.79) | 5.40 (3.33 to 7.91) | -0.70 (-1.26 to -0.14) |
| Ethiopia | 109126.53 (72082.31 to 141585.49) | 307.88 (208.93 to 384.92) | 12.31 (8.34 to 15.23) | 75995.80 (60382.47 to 92951.52) | 139.80 (109.01 to 168.49) | 10.16 (7.85 to 12.17) | -2.92 (-3.05 to -2.78) |
| Fiji | 737.52 (483.79 to 974.64) | 231.27 (153.09 to 304.67) | 18.12 (12.14 to 23.27) | 821.60 (314.33 to 1381.02) | 128.92 (48.72 to 213.74) | 8.92 (3.51 to 14.47) | -2.04 (-2.27 to -1.82) |
| Finland | 1854.16 (334.04 to 3785.46) | 25.95 (4.77 to 52.82) | 3.62 (0.66 to 7.40) | 436.67 (56.92 to 930.77) | 2.88 (0.43 to 6.07) | 0.71 (0.11 to 1.49) | -7.52 (-7.99 to -7.04) |
| France | 31283.25 (15070.53 to 50632.63) | 35.97 (17.48 to 58.08) | 5.68 (2.76 to 9.19) | 13398.91 (9056.37 to 18546.43) | 7.67 (5.24 to 10.59) | 1.95 (1.33 to 2.69) | -4.97 (-5.28 to -4.67) |
| Gabon | 879.50 (568.15 to 1233.77) | 145.17 (89.45 to 209.27) | 9.29 (5.83 to 13.11) | 933.80 (592.74 to 1381.41) | 104.18 (66.44 to 151.51) | 6.79 (4.55 to 9.26) | -1.02 (-1.15 to -0.89) |
| Gambia | 1495.35 (1057.53 to 1914.58) | 264.18 (197.81 to 330.27) | 18.01 (13.79 to 21.34) | 2523.72 (1907.58 to 3139.38) | 246.02 (184.34 to 304.79) | 16.11 (12.98 to 19.02) | -0.22 (-0.31 to -0.13) |
| Georgia | 8543.91 (4138.16 to 13136.78) | 152.61 (74.06 to 233.00) | 15.69 (7.61 to 23.94) | 4337.66 (2717.97 to 6162.98) | 69.09 (43.52 to 98.10) | 7.06 (4.41 to 10.00) | -3.40 (-4.09 to -2.69) |
| Germany | 94338.32 (49801.02 to 146727.93) | 71.80 (38.30 to 111.40) | 9.87 (5.27 to 15.31) | 29061.71 (20290.67 to 38634.86) | 12.87 (9.07 to 17.03) | 2.76 (1.95 to 3.65) | -5.43 (-5.64 to -5.23) |
| Ghana | 20649.33 (15522.77 to 25896.27) | 258.50 (203.39 to 321.49) | 17.52 (14.16 to 20.27) | 29390.94 (22420.02 to 36728.19) | 187.21 (142.02 to 235.51) | 13.42 (10.75 to 16.12) | -0.90 (-1.06 to -0.74) |
| Greece | 10959.08 (6138.21 to 16803.15) | 76.61 (43.19 to 117.28) | 11.43 (6.44 to 17.46) | 7559.71 (5764.31 to 9503.57) | 25.42 (19.64 to 31.76) | 5.00 (3.84 to 6.27) | -3.83 (-4.26 to -3.40) |
| Greenland | 8.04 (0.81 to 20.74) | 29.47 (1.76 to 77.56) | 2.17 (0.13 to 5.69) | 5.25 (0.27 to 13.35) | 9.18 (0.53 to 23.58) | 1.21 (0.07 to 3.08) | -4.26 (-4.70 to -3.81) |
| Grenada | 99.62 (59.92 to 146.35) | 124.80 (74.63 to 184.28) | 13.23 (7.96 to 19.44) | 66.32 (32.14 to 106.91) | 67.84 (33.24 to 108.50) | 6.21 (3.10 to 10.04) | -1.85 (-2.03 to -1.67) |
| Guam | 17.61 (0.69 to 45.38) | 30.05 (0.38 to 79.35) | 3.62 (0.05 to 9.58) | 39.46 (19.49 to 59.52) | 18.80 (9.41 to 28.27) | 3.35 (1.71 to 5.08) | -0.70 (-1.49 to 0.10) |
| Guatemala | 9331.58 (5920.70 to 11972.27) | 209.97 (142.60 to 263.95) | 15.11 (10.31 to 18.97) | 8762.60 (5755.10 to 11619.65) | 88.24 (58.33 to 116.40) | 8.49 (5.65 to 10.89) | -2.90 (-3.11 to -2.69) |
| Guinea | 15338.04 (9771.25 to 20476.19) | 276.75 (196.35 to 351.97) | 15.58 (10.99 to 19.34) | 15870.50 (11400.71 to 20838.59) | 229.76 (173.52 to 296.17) | 13.90 (10.96 to 16.50) | -0.32 (-0.43 to -0.20) |
| Guinea-Bissau | 2255.10 (1588.84 to 2949.19) | 374.49 (283.13 to 470.05) | 16.68 (12.91 to 19.85) | 2282.39 (1727.68 to 2826.25) | 296.04 (229.62 to 361.61) | 14.78 (12.11 to 17.77) | -0.59 (-0.66 to -0.52) |
| Guyana | 733.28 (478.22 to 1032.14) | 178.78 (110.18 to 264.59) | 13.16 (8.19 to 19.51) | 554.13 (294.26 to 854.92) | 96.87 (50.72 to 150.19) | 6.79 (3.42 to 10.79) | -1.77 (-1.94 to -1.60) |
| Haiti | 13449.20 (10519.88 to 16670.09) | 373.01 (305.20 to 444.83) | 20.05 (17.01 to 23.12) | 17654.56 (13201.71 to 22594.63) | 269.25 (205.42 to 346.11) | 15.62 (11.64 to 19.17) | -0.87 (-0.94 to -0.81) |
| Honduras | 3637.16 (2914.75 to 4357.47) | 151.01 (124.89 to 176.48) | 16.68 (14.00 to 19.14) | 7900.54 (6139.48 to 9910.95) | 148.39 (114.94 to 185.08) | 11.97 (9.36 to 14.52) | 0.18 (-0.10 to 0.45) |
| Hungary | 18581.37 (10439.47 to 28377.93) | 137.87 (78.25 to 208.97) | 12.66 (7.22 to 19.20) | 8562.88 (6285.95 to 12909.91) | 40.97 (30.16 to 61.93) | 5.26 (3.89 to 7.92) | -3.87 (-4.11 to -3.62) |
| Iceland | 46.93 (3.83 to 105.75) | 15.53 (1.33 to 34.88) | 2.64 (0.23 to 5.99) | 19.08 (3.58 to 41.50) | 2.86 (0.57 to 6.19) | 0.79 (0.16 to 1.72) | -5.74 (-6.18 to -5.29) |
| India | 1308971.09 (1067722.87 to 1518275.50) | 257.67 (215.35 to 296.11) | 18.53 (15.55 to 21.16) | 1995558.23 (1679989.50 to 2304833.94) | 191.35 (161.02 to 220.14) | 17.64 (14.94 to 19.98) | -0.79 (-0.97 to -0.62) |
| Indonesia | 195678.56 (158617.72 to 230428.40) | 203.82 (162.16 to 240.79) | 16.69 (13.43 to 19.77) | 218085.86 (154064.66 to 293111.13) | 115.69 (81.82 to 154.07) | 10.38 (7.46 to 13.72) | -1.81 (-2.15 to -1.46) |
| Iran (Islamic Republic of) | 33344.62 (27453.50 to 39123.11) | 139.20 (112.55 to 163.62) | 13.91 (11.36 to 16.20) | 49569.01 (40092.53 to 58243.52) | 74.16 (59.84 to 87.38) | 9.16 (7.38 to 10.83) | -1.96 (-2.05 to -1.86) |
| Iraq | 17644.98 (13711.24 to 21611.50) | 184.23 (141.31 to 233.04) | 18.04 (14.35 to 21.67) | 28982.75 (21285.97 to 37642.70) | 151.39 (112.44 to 193.01) | 13.86 (10.85 to 16.93) | -0.92 (-1.07 to -0.77) |
| Ireland | 2280.01 (935.82 to 3912.39) | 57.93 (23.82 to 99.59) | 7.18 (2.97 to 12.29) | 595.86 (313.73 to 920.78) | 7.12 (3.75 to 11.01) | 1.79 (0.94 to 2.77) | -6.83 (-7.15 to -6.51) |
| Israel | 3198.14 (1880.44 to 4672.82) | 70.40 (41.30 to 103.32) | 10.96 (6.41 to 16.09) | 2648.55 (2070.27 to 3218.85) | 19.30 (15.22 to 23.34) | 5.12 (4.03 to 6.17) | -4.53 (-4.70 to -4.36) |
| Italy | 55539.51 (32741.17 to 80640.52) | 64.10 (38.15 to 92.82) | 9.96 (5.93 to 14.39) | 29415.57 (22060.41 to 37154.70) | 15.73 (11.97 to 19.59) | 3.92 (3.00 to 4.89) | -4.61 (-4.82 to -4.41) |
| Jamaica | 1796.77 (1289.24 to 2282.04) | 93.56 (66.35 to 119.00) | 14.01 (9.97 to 17.95) | 1433.36 (845.02 to 2095.72) | 45.06 (27.13 to 65.72) | 5.98 (3.74 to 8.54) | -1.90 (-2.40 to -1.40) |
| Japan | 38767.11 (8232.38 to 82252.13) | 25.27 (5.43 to 53.59) | 4.78 (1.03 to 10.13) | 49339.58 (26188.03 to 75321.35) | 9.91 (5.41 to 14.85) | 3.19 (1.73 to 4.77) | -3.06 (-3.38 to -2.75) |
| Jordan | 1365.80 (1047.82 to 1710.50) | 99.33 (76.54 to 123.20) | 11.61 (9.15 to 13.95) | 3567.97 (2653.59 to 4594.11) | 61.66 (46.45 to 78.65) | 8.59 (6.72 to 10.60) | -1.97 (-2.30 to -1.63) |
| Kazakhstan | 14946.15 (6726.51 to 24795.43) | 129.37 (58.65 to 213.95) | 12.35 (5.52 to 20.35) | 12936.13 (9258.64 to 17209.07) | 91.16 (64.96 to 121.81) | 7.82 (5.52 to 10.44) | -2.11 (-2.58 to -1.63) |
| Kenya | 20154.92 (13646.19 to 26004.75) | 153.69 (104.95 to 196.30) | 12.03 (8.38 to 15.13) | 29845.68 (22868.61 to 36861.44) | 146.26 (107.49 to 185.07) | 9.76 (7.40 to 11.91) | 0.17 (-0.02 to 0.36) |
| Kiribati | 122.27 (98.95 to 147.39) | 325.78 (265.00 to 388.61) | 19.23 (16.26 to 22.28) | 135.41 (102.43 to 178.05) | 219.05 (170.88 to 279.63) | 15.44 (12.63 to 18.67) | -1.37 (-1.58 to -1.17) |
| Kuwait | 629.77 (514.30 to 746.66) | 115.47 (92.51 to 136.27) | 17.14 (13.78 to 20.17) | 1764.95 (1318.94 to 2276.29) | 70.95 (52.33 to 91.77) | 15.31 (12.19 to 18.36) | -1.11 (-1.67 to -0.55) |
| Kyrgyzstan | 6976.88 (4996.48 to 9010.29) | 234.35 (171.98 to 295.56) | 21.75 (15.79 to 27.08) | 5376.61 (4100.63 to 6687.92) | 135.59 (103.25 to 168.29) | 15.65 (12.31 to 19.15) | -1.91 (-2.37 to -1.45) |
| Lao People's Democratic Republic | 11717.97 (8291.72 to 15425.08) | 437.42 (345.15 to 539.39) | 22.37 (18.47 to 25.94) | 9172.81 (6400.59 to 11800.74) | 228.68 (157.34 to 291.54) | 19.99 (14.32 to 24.66) | -2.25 (-2.34 to -2.15) |
| Latvia | 5521.80 (3161.91 to 7837.44) | 160.07 (92.07 to 226.98) | 15.70 (9.05 to 22.31) | 1571.26 (1049.48 to 2299.53) | 34.78 (23.24 to 50.71) | 4.14 (2.77 to 6.00) | -5.82 (-6.27 to -5.36) |
| Lebanon | 1610.13 (1090.10 to 2263.79) | 81.82 (54.62 to 116.06) | 8.21 (5.58 to 11.12) | 2850.02 (1863.36 to 3976.28) | 43.91 (28.94 to 61.16) | 5.65 (3.82 to 7.65) | -1.92 (-2.19 to -1.66) |
| Lesotho | 2038.75 (1483.42 to 2541.10) | 201.85 (150.25 to 254.56) | 14.53 (11.19 to 17.57) | 2454.03 (1756.17 to 3191.66) | 238.48 (175.85 to 308.26) | 7.14 (5.67 to 8.98) | 1.43 (0.95 to 1.92) |
| Liberia | 5531.53 (3631.93 to 7396.64) | 272.78 (202.51 to 339.91) | 14.05 (10.64 to 16.89) | 4592.74 (3432.96 to 6029.83) | 204.63 (158.63 to 265.33) | 14.24 (11.72 to 16.90) | -0.96 (-1.06 to -0.86) |
| Libya | 1804.38 (1359.63 to 2368.08) | 89.40 (65.21 to 117.95) | 11.81 (8.97 to 14.97) | 4455.05 (3214.42 to 6110.36) | 98.89 (71.64 to 134.04) | 10.24 (7.74 to 13.05) | 0.87 (0.47 to 1.27) |
| Lithuania | 5109.00 (2646.63 to 7705.71) | 116.70 (60.69 to 175.65) | 12.78 (6.63 to 19.28) | 1728.74 (1066.33 to 2449.08) | 26.14 (16.21 to 36.94) | 3.29 (2.04 to 4.66) | -5.31 (-5.64 to -4.98) |
| Luxembourg | 295.56 (132.93 to 479.86) | 56.01 (25.44 to 90.72) | 7.50 (3.45 to 12.04) | 87.06 (49.43 to 125.01) | 7.40 (4.28 to 10.63) | 1.86 (1.10 to 2.68) | -6.19 (-6.54 to -5.85) |
| Madagascar | 21082.59 (14686.03 to 26022.04) | 302.70 (236.10 to 358.82) | 17.65 (14.00 to 20.85) | 27599.84 (20948.56 to 34309.30) | 247.94 (188.33 to 311.29) | 15.41 (12.51 to 17.99) | -0.73 (-0.78 to -0.67) |
| Malawi | 17591.83 (11146.25 to 23171.85) | 247.52 (176.43 to 309.14) | 12.14 (8.63 to 14.84) | 15876.10 (12061.88 to 19914.07) | 200.68 (155.99 to 246.83) | 10.00 (7.95 to 11.86) | -0.86 (-1.04 to -0.68) |
| Malaysia | 10358.61 (5447.15 to 15391.38) | 116.64 (58.63 to 176.38) | 13.85 (6.97 to 20.94) | 15239.26 (10461.14 to 20436.67) | 61.44 (41.89 to 82.63) | 7.00 (4.69 to 9.41) | -2.16 (-2.37 to -1.95) |
| Maldives | 251.66 (197.97 to 300.77) | 260.86 (198.44 to 310.30) | 22.16 (17.05 to 25.99) | 76.98 (44.26 to 118.16) | 27.41 (15.72 to 42.19) | 5.27 (3.02 to 7.98) | -8.33 (-8.74 to -7.93) |
| Mali | 17613.98 (13322.59 to 21666.62) | 248.92 (195.49 to 297.92) | 12.89 (10.27 to 15.06) | 26226.61 (20225.09 to 32433.29) | 186.55 (146.86 to 229.92) | 10.84 (8.96 to 12.76) | -0.75 (-0.82 to -0.68) |
| Malta | 257.01 (117.93 to 405.26) | 64.82 (29.91 to 101.31) | 9.39 (4.34 to 14.74) | 180.53 (127.60 to 240.22) | 16.66 (11.97 to 21.95) | 4.24 (2.99 to 5.57) | -4.45 (-4.82 to -4.08) |
| Marshall Islands | 36.51 (26.86 to 49.42) | 234.53 (170.39 to 313.81) | 14.90 (10.91 to 19.42) | 51.97 (34.44 to 77.13) | 182.75 (126.53 to 262.10) | 13.14 (9.43 to 17.84) | -0.49 (-0.67 to -0.32) |
| Mauritania | 3371.55 (2562.94 to 4161.80) | 267.40 (208.47 to 327.55) | 18.02 (14.28 to 21.41) | 3682.61 (2851.77 to 4652.01) | 170.03 (129.83 to 215.43) | 15.84 (12.76 to 18.61) | -1.58 (-1.72 to -1.44) |
| Mauritius | 347.39 (169.88 to 536.72) | 54.13 (25.09 to 84.18) | 5.39 (2.51 to 8.45) | 418.84 (151.56 to 729.23) | 24.55 (9.30 to 42.37) | 3.12 (1.20 to 5.37) | -2.52 (-2.88 to -2.16) |
| Mexico | 43546.23 (30821.44 to 55731.58) | 102.28 (68.89 to 132.52) | 11.31 (7.72 to 14.65) | 48036.93 (33317.87 to 67962.48) | 41.60 (28.74 to 58.65) | 4.52 (3.11 to 6.35) | -3.07 (-3.28 to -2.86) |
| Micronesia (Federated States of) | 157.88 (121.99 to 195.93) | 335.14 (264.56 to 409.42) | 22.16 (18.10 to 26.15) | 108.73 (71.31 to 158.03) | 178.41 (119.81 to 256.44) | 14.32 (9.76 to 19.39) | -2.11 (-2.14 to -2.07) |
| Monaco | 17.75 (3.02 to 35.89) | 22.69 (3.95 to 45.80) | 3.81 (0.68 to 7.63) | 14.30 (7.26 to 22.23) | 12.58 (6.48 to 19.74) | 2.26 (1.18 to 3.47) | -1.64 (-2.60 to -0.68) |
| Mongolia | 3672.04 (2727.49 to 4612.83) | 305.84 (250.83 to 366.09) | 22.55 (18.78 to 26.68) | 2355.03 (1705.79 to 3144.75) | 130.20 (92.09 to 173.79) | 12.84 (9.13 to 17.08) | -3.26 (-3.67 to -2.85) |
| Montenegro | 730.48 (435.65 to 1031.76) | 126.21 (75.02 to 177.64) | 17.44 (10.42 to 24.50) | 822.13 (571.17 to 1376.72) | 95.87 (66.45 to 160.64) | 8.35 (5.81 to 13.86) | -1.13 (-1.40 to -0.85) |
| Morocco | 21989.41 (17076.57 to 27511.67) | 144.64 (111.28 to 178.39) | 13.97 (11.00 to 17.12) | 27798.47 (19775.12 to 36093.94) | 94.37 (66.63 to 121.18) | 9.92 (7.39 to 12.38) | -1.18 (-1.40 to -0.97) |
| Mozambique | 23361.58 (16318.12 to 30546.34) | 240.81 (181.44 to 295.24) | 12.70 (9.74 to 15.33) | 28521.44 (21345.26 to 35767.25) | 218.99 (166.19 to 276.45) | 10.34 (8.41 to 12.38) | 0.16 (-0.01 to 0.32) |
| Myanmar | 101129.91 (76295.51 to 124948.44) | 430.50 (344.49 to 524.73) | 23.93 (20.26 to 27.12) | 100430.14 (76013.42 to 127275.95) | 241.86 (185.46 to 303.41) | 20.97 (16.42 to 25.43) | -2.12 (-2.29 to -1.94) |
| Namibia | 1545.66 (1094.28 to 1977.01) | 227.21 (158.77 to 295.26) | 15.87 (11.23 to 19.68) | 1493.08 (884.87 to 2322.07) | 122.37 (71.59 to 190.79) | 6.23 (3.69 to 9.52) | -2.13 (-2.52 to -1.73) |
| Nauru | 1.91 (0.08 to 6.32) | 43.20 (0.53 to 150.58) | 2.48 (0.03 to 8.65) | 2.27 (0.37 to 4.97) | 42.79 (6.59 to 92.99) | 2.65 (0.42 to 5.81) | -1.24 (-2.57 to 0.11) |
| Nepal | 42073.07 (30662.68 to 52869.22) | 331.91 (261.47 to 406.50) | 21.55 (17.49 to 25.15) | 46555.73 (38060.47 to 57038.43) | 231.47 (189.75 to 282.80) | 19.38 (17.02 to 21.80) | -1.00 (-1.18 to -0.82) |
| Netherlands | 12916.49 (6875.24 to 19752.40) | 63.81 (34.07 to 97.07) | 9.83 (5.25 to 14.97) | 5144.31 (3692.13 to 6755.13) | 13.26 (9.53 to 17.29) | 2.92 (2.11 to 3.81) | -5.44 (-5.72 to -5.17) |
| New Zealand | 596.48 (21.35 to 1746.19) | 15.63 (0.66 to 45.54) | 2.23 (0.09 to 6.51) | 613.61 (261.42 to 1001.34) | 6.80 (2.95 to 11.08) | 1.71 (0.74 to 2.77) | -3.18 (-3.55 to -2.80) |
| Nicaragua | 2385.01 (1844.90 to 2895.94) | 107.72 (87.73 to 123.77) | 16.32 (13.42 to 18.71) | 2484.76 (1912.17 to 3113.94) | 57.12 (43.72 to 71.64) | 7.04 (5.38 to 8.67) | -1.70 (-2.00 to -1.40) |
| Niger | 20251.23 (9722.09 to 29234.86) | 283.70 (179.80 to 364.60) | 14.08 (9.00 to 17.70) | 24624.49 (16350.75 to 33260.85) | 195.95 (143.60 to 253.83) | 13.46 (10.10 to 16.13) | -1.18 (-1.30 to -1.06) |
| Nigeria | 177448.97 (119571.96 to 226829.55) | 244.74 (184.90 to 298.52) | 15.00 (11.46 to 17.71) | 206012.29 (156870.47 to 259211.59) | 156.65 (122.16 to 190.80) | 11.87 (9.37 to 14.10) | -1.49 (-1.62 to -1.35) |
| Niue | 2.62 (1.09 to 4.25) | 111.08 (46.14 to 179.51) | 10.04 (4.03 to 15.74) | 0.64 (0.08 to 1.35) | 32.43 (4.39 to 68.10) | 2.90 (0.38 to 6.14) | -5.18 (-5.76 to -4.61) |
| North Macedonia | 3638.55 (2607.12 to 4501.36) | 221.82 (159.10 to 274.64) | 22.37 (15.98 to 27.65) | 3250.64 (2447.91 to 4252.34) | 132.59 (101.37 to 170.70) | 10.66 (8.31 to 13.66) | -2.00 (-2.63 to -1.37) |
| Northern Mariana Islands | 5.20 (0.27 to 13.79) | 36.09 (0.55 to 96.66) | 3.79 (0.05 to 9.60) | 14.02 (6.92 to 21.67) | 33.68 (16.67 to 52.75) | 3.60 (1.75 to 5.78) | -0.14 (-0.68 to 0.41) |
| Norway | 2422.33 (770.32 to 4343.39) | 32.73 (10.52 to 58.42) | 5.06 (1.62 to 9.06) | 457.91 (174.39 to 816.03) | 3.94 (1.55 to 6.89) | 1.05 (0.41 to 1.84) | -7.27 (-7.59 to -6.95) |
| Oman | 1287.34 (909.25 to 1696.83) | 184.61 (128.46 to 247.39) | 18.06 (13.70 to 22.85) | 1593.81 (1173.26 to 2048.40) | 109.53 (80.26 to 138.50) | 11.65 (9.01 to 14.14) | -1.12 (-1.33 to -0.91) |
| Pakistan | 174113.93 (142324.22 to 205800.20) | 233.18 (192.54 to 271.58) | 18.68 (15.48 to 21.57) | 250886.67 (210160.69 to 301849.23) | 206.49 (170.47 to 252.71) | 15.66 (13.59 to 17.76) | -0.53 (-0.73 to -0.33) |
| Palau | 3.15 (0.13 to 7.99) | 36.02 (0.94 to 93.49) | 2.80 (0.08 to 7.04) | 5.80 (0.63 to 11.96) | 33.09 (3.71 to 68.14) | 2.94 (0.33 to 5.84) | 0.28 (-0.59 to 1.15) |
| Palestine | 1336.76 (964.85 to 1712.91) | 153.49 (110.31 to 199.30) | 13.70 (10.16 to 17.06) | 1978.19 (1425.46 to 2532.88) | 102.89 (73.50 to 131.04) | 11.48 (8.32 to 14.30) | -1.34 (-1.68 to -1.00) |
| Panama | 988.57 (613.95 to 1319.00) | 66.99 (40.73 to 90.09) | 11.24 (6.94 to 15.14) | 795.65 (443.63 to 1209.51) | 17.69 (9.97 to 26.73) | 3.30 (1.98 to 4.97) | -4.34 (-4.60 to -4.09) |
| Papua New Guinea | 6846.72 (5126.72 to 8759.54) | 375.86 (291.86 to 473.10) | 25.89 (22.25 to 29.47) | 13785.80 (10445.15 to 17459.28) | 302.69 (230.47 to 381.61) | 20.41 (15.04 to 24.99) | -0.61 (-0.66 to -0.56) |
| Paraguay | 2576.84 (1792.85 to 3227.22) | 107.39 (71.16 to 137.38) | 16.76 (10.98 to 20.90) | 2291.15 (903.85 to 3941.59) | 41.70 (16.28 to 71.58) | 4.68 (1.83 to 7.80) | -2.87 (-3.17 to -2.58) |
| Peru | 19590.00 (13108.09 to 25144.55) | 133.58 (91.86 to 170.22) | 15.96 (11.06 to 19.87) | 15123.15 (9469.81 to 21435.05) | 45.03 (28.28 to 63.76) | 4.75 (3.13 to 6.54) | -3.83 (-4.07 to -3.60) |
| Philippines | 62005.90 (48181.92 to 74466.56) | 213.40 (172.15 to 252.30) | 21.02 (17.14 to 24.60) | 98136.87 (73278.70 to 125520.13) | 136.82 (101.14 to 175.02) | 11.80 (8.92 to 14.77) | -1.14 (-1.33 to -0.96) |
| Poland | 67527.77 (45535.73 to 87829.30) | 166.53 (112.72 to 215.88) | 17.44 (11.80 to 22.57) | 33403.74 (25684.68 to 42671.13) | 43.76 (33.77 to 55.86) | 6.13 (4.73 to 7.79) | -4.43 (-4.60 to -4.25) |
| Portugal | 6418.09 (2565.18 to 10875.29) | 51.66 (21.03 to 87.25) | 6.24 (2.54 to 10.55) | 2283.64 (1243.12 to 3468.47) | 7.65 (4.27 to 11.57) | 1.73 (0.96 to 2.61) | -6.37 (-6.84 to -5.90) |
| Puerto Rico | 498.97 (10.30 to 1519.90) | 14.88 (0.33 to 45.35) | 1.96 (0.04 to 5.95) | 479.05 (124.96 to 847.99) | 5.99 (1.67 to 10.52) | 1.30 (0.35 to 2.30) | -3.29 (-3.70 to -2.87) |
| Qatar | 213.44 (170.71 to 257.63) | 296.19 (240.44 to 354.10) | 23.80 (19.97 to 27.08) | 562.12 (408.93 to 735.29) | 108.69 (79.87 to 136.71) | 16.08 (13.27 to 18.68) | -3.77 (-4.52 to -3.01) |
| Republic of Korea | 17852.24 (7999.04 to 29416.57) | 82.55 (37.21 to 136.62) | 8.75 (3.96 to 14.46) | 25482.92 (17516.45 to 33517.22) | 28.13 (19.31 to 36.92) | 7.52 (5.16 to 9.88) | -3.73 (-3.97 to -3.48) |
| Republic of Moldova | 9626.49 (7633.62 to 11408.05) | 272.74 (216.57 to 323.64) | 24.16 (19.11 to 28.82) | 3806.02 (2466.65 to 5358.69) | 64.09 (41.74 to 89.94) | 7.35 (4.85 to 10.28) | -5.67 (-6.09 to -5.25) |
| Romania | 39993.80 (23462.88 to 56558.56) | 176.34 (103.90 to 249.26) | 16.49 (9.71 to 23.27) | 19134.20 (13950.36 to 26565.70) | 48.37 (35.49 to 66.69) | 5.45 (4.00 to 7.52) | -4.81 (-5.15 to -4.47) |
| Russian Federation | 212852.61 (104434.99 to 323562.56) | 136.69 (67.60 to 207.70) | 13.26 (6.56 to 20.17) | 92834.92 (56185.50 to 142126.89) | 38.85 (23.58 to 59.39) | 3.70 (2.25 to 5.66) | -4.86 (-5.42 to -4.30) |
| Rwanda | 15324.77 (10175.10 to 20207.76) | 369.93 (281.88 to 456.66) | 16.44 (12.76 to 19.97) | 10253.22 (7434.09 to 13095.61) | 175.86 (131.81 to 226.51) | 12.56 (10.04 to 14.88) | -3.32 (-3.69 to -2.95) |
| Saint Kitts and Nevis | 20.12 (5.57 to 37.78) | 55.26 (15.10 to 103.97) | 4.56 (1.22 to 8.63) | 9.41 (3.50 to 15.99) | 17.50 (6.77 to 29.54) | 1.84 (0.71 to 3.10) | -3.51 (-3.95 to -3.06) |
| Saint Lucia | 106.39 (63.13 to 152.89) | 137.29 (79.51 to 199.11) | 12.53 (7.26 to 18.29) | 111.91 (52.61 to 174.70) | 50.79 (24.74 to 79.34) | 5.87 (2.88 to 9.18) | -3.67 (-4.12 to -3.22) |
| Saint Vincent and the Grenadines | 85.02 (52.70 to 122.65) | 124.35 (75.54 to 180.45) | 12.53 (7.64 to 18.10) | 79.44 (34.68 to 126.03) | 63.04 (27.59 to 99.66) | 6.98 (3.19 to 11.19) | -2.18 (-2.46 to -1.90) |
| Samoa | 216.53 (174.00 to 261.26) | 277.98 (225.39 to 331.32) | 24.67 (20.45 to 28.76) | 251.93 (171.32 to 337.83) | 196.33 (132.53 to 260.87) | 19.88 (13.68 to 24.87) | -1.09 (-1.14 to -1.03) |
| San Marino | 10.13 (3.96 to 17.42) | 27.62 (11.09 to 47.28) | 5.23 (2.16 to 8.97) | 5.75 (2.98 to 9.66) | 5.92 (3.04 to 9.87) | 2.08 (1.16 to 3.11) | -4.46 (-4.97 to -3.94) |
| Sao Tome and Principe | 180.34 (127.14 to 229.32) | 232.50 (176.05 to 285.25) | 18.94 (14.36 to 22.63) | 143.73 (109.82 to 180.20) | 151.69 (117.23 to 188.01) | 14.38 (11.23 to 17.56) | -1.33 (-1.57 to -1.09) |
| Saudi Arabia | 10070.15 (7473.79 to 13173.66) | 170.29 (125.68 to 224.13) | 15.47 (12.31 to 18.66) | 21494.23 (15994.57 to 27495.12) | 135.62 (105.69 to 167.38) | 15.80 (12.93 to 18.70) | -0.71 (-0.94 to -0.48) |
| Senegal | 11659.61 (8717.15 to 14413.03) | 241.57 (194.50 to 288.48) | 16.11 (12.98 to 18.81) | 15208.97 (11857.91 to 18694.77) | 196.47 (151.52 to 244.53) | 14.69 (12.22 to 16.93) | -0.63 (-0.69 to -0.57) |
| Serbia | 20944.29 (13348.09 to 27654.44) | 252.55 (160.92 to 334.48) | 21.13 (13.45 to 28.00) | 15673.08 (11665.97 to 21273.95) | 91.52 (68.34 to 124.08) | 10.24 (7.74 to 13.93) | -4.06 (-4.47 to -3.65) |
| Seychelles | 17.23 (5.48 to 30.76) | 30.17 (9.36 to 54.25) | 3.18 (1.00 to 5.72) | 20.93 (7.60 to 37.90) | 20.79 (7.70 to 37.56) | 2.50 (0.92 to 4.40) | -1.51 (-1.79 to -1.23) |
| Sierra Leone | 10427.24 (6915.44 to 13815.44) | 295.57 (221.12 to 367.82) | 16.14 (12.05 to 19.49) | 9908.18 (7364.04 to 12770.75) | 226.43 (172.32 to 286.49) | 15.24 (12.16 to 18.05) | -0.70 (-0.84 to -0.56) |
| Singapore | 2035.02 (880.93 to 3203.11) | 107.49 (46.85 to 170.31) | 15.47 (6.78 to 24.52) | 1547.70 (781.99 to 2367.12) | 18.80 (9.51 to 28.75) | 6.43 (3.26 to 9.71) | -5.19 (-5.57 to -4.80) |
| Slovakia | 7592.93 (4180.87 to 11065.42) | 133.34 (73.38 to 194.23) | 13.77 (7.54 to 20.04) | 4238.72 (3212.40 to 5394.02) | 44.95 (34.03 to 57.12) | 5.72 (4.33 to 7.28) | -3.28 (-3.54 to -3.03) |
| Slovenia | 1771.63 (980.31 to 2620.27) | 73.59 (41.03 to 108.00) | 9.69 (5.43 to 14.19) | 829.96 (601.01 to 1078.10) | 16.15 (11.85 to 20.82) | 3.43 (2.54 to 4.44) | -5.11 (-5.34 to -4.89) |
| Solomon Islands | 559.09 (391.57 to 734.35) | 435.27 (333.35 to 547.96) | 26.56 (21.31 to 31.15) | 1068.50 (821.05 to 1390.83) | 360.85 (285.77 to 459.42) | 27.31 (23.26 to 31.18) | -0.59 (-0.67 to -0.51) |
| Somalia | 13812.67 (9011.34 to 19076.76) | 308.09 (218.68 to 396.02) | 13.21 (9.44 to 16.36) | 21741.44 (14746.48 to 29014.44) | 226.91 (164.76 to 293.69) | 8.90 (6.42 to 11.17) | -0.90 (-0.96 to -0.84) |
| South Africa | 26186.28 (18806.16 to 33887.04) | 106.91 (78.20 to 138.30) | 9.97 (7.31 to 12.87) | 33488.71 (24600.70 to 43241.88) | 81.98 (59.78 to 105.37) | 5.02 (3.68 to 6.40) | -0.84 (-1.33 to -0.35) |
| South Sudan | 9694.57 (6759.14 to 13011.07) | 239.56 (168.25 to 314.36) | 12.73 (9.16 to 15.76) | 12022.73 (8237.71 to 16331.08) | 217.38 (159.87 to 288.44) | 10.98 (8.59 to 13.35) | -0.41 (-0.66 to -0.17) |
| Spain | 22381.04 (10122.34 to 37501.13) | 42.53 (19.35 to 70.87) | 6.58 (2.99 to 10.98) | 9932.51 (6442.53 to 13872.02) | 8.17 (5.36 to 11.24) | 2.11 (1.38 to 2.91) | -5.22 (-5.56 to -4.87) |
| Sri Lanka | 19860.12 (17040.73 to 22559.17) | 229.47 (197.28 to 260.00) | 22.77 (19.90 to 25.50) | 19862.02 (10696.27 to 33071.79) | 83.94 (45.45 to 139.94) | 12.73 (7.39 to 19.93) | -2.88 (-3.35 to -2.41) |
| Sudan | 40051.08 (29989.16 to 49588.21) | 333.14 (269.10 to 401.24) | 22.50 (18.76 to 25.89) | 34719.50 (25726.11 to 45971.57) | 189.38 (142.15 to 246.80) | 16.30 (12.69 to 19.94) | -1.94 (-2.01 to -1.88) |
| Suriname | 319.43 (188.37 to 468.32) | 125.00 (73.36 to 184.98) | 13.28 (7.67 to 19.65) | 412.83 (222.32 to 650.38) | 69.71 (37.93 to 110.16) | 7.75 (4.21 to 11.57) | -2.04 (-2.36 to -1.72) |
| Sweden | 4680.75 (1274.36 to 8820.62) | 28.27 (7.78 to 53.03) | 4.69 (1.30 to 8.82) | 911.15 (280.95 to 1703.41) | 3.45 (1.08 to 6.35) | 0.93 (0.29 to 1.73) | -7.07 (-7.49 to -6.65) |
| Switzerland | 5455.95 (2716.03 to 8767.83) | 49.00 (24.40 to 78.27) | 8.28 (4.12 to 13.26) | 1580.63 (1014.19 to 2188.71) | 7.05 (4.56 to 9.61) | 2.14 (1.40 to 2.94) | -6.16 (-6.40 to -5.93) |
| Syrian Arab Republic | 8050.08 (5850.98 to 10705.01) | 148.47 (105.37 to 195.22) | 14.37 (10.89 to 18.00) | 12836.01 (8882.66 to 17509.03) | 127.90 (89.80 to 172.47) | 12.71 (9.53 to 16.29) | -0.57 (-0.78 to -0.36) |
| Taiwan (Province of China) | 9260.20 (4321.34 to 15660.41) | 73.60 (34.14 to 124.98) | 9.87 (4.55 to 16.73) | 10524.83 (7922.54 to 13392.13) | 24.17 (18.33 to 30.69) | 5.49 (4.15 to 6.98) | -3.15 (-3.42 to -2.87) |
| Tajikistan | 9134.61 (6704.48 to 11567.71) | 272.83 (213.16 to 329.95) | 25.06 (20.03 to 30.16) | 8518.57 (6793.09 to 10689.86) | 173.92 (136.95 to 215.59) | 16.79 (13.49 to 20.46) | -1.85 (-2.20 to -1.50) |
| Thailand | 46851.45 (36012.43 to 56396.12) | 152.69 (115.98 to 184.72) | 17.80 (13.82 to 20.71) | 56974.24 (39744.36 to 76767.24) | 53.81 (37.63 to 72.29) | 8.67 (6.49 to 10.85) | -3.93 (-4.23 to -3.63) |
| Timor-Leste | 1270.72 (805.95 to 1707.01) | 275.03 (213.71 to 336.75) | 19.62 (15.63 to 23.08) | 1524.98 (1007.22 to 2049.82) | 193.07 (122.84 to 262.40) | 17.29 (11.32 to 22.04) | -1.33 (-1.53 to -1.13) |
| Togo | 5314.30 (3711.86 to 6896.57) | 263.56 (201.47 to 322.49) | 17.25 (13.22 to 20.16) | 8235.94 (5918.06 to 10832.35) | 225.27 (168.91 to 291.25) | 15.45 (12.20 to 18.26) | -0.51 (-0.65 to -0.38) |
| Tokelau | 0.36 (0.02 to 1.17) | 28.26 (1.74 to 93.08) | 2.41 (0.15 to 7.82) | 0.29 (0.01 to 0.70) | 20.39 (1.21 to 48.52) | 1.99 (0.12 to 4.69) | -1.99 (-2.39 to -1.58) |
| Tonga | 93.17 (70.19 to 115.00) | 181.06 (137.74 to 220.21) | 19.51 (15.37 to 23.14) | 86.22 (51.26 to 119.89) | 110.54 (65.55 to 153.63) | 13.32 (8.24 to 17.95) | -1.44 (-1.58 to -1.30) |
| Trinidad and Tobago | 831.34 (220.31 to 1513.22) | 112.55 (27.58 to 207.36) | 10.59 (2.63 to 19.59) | 1165.45 (498.23 to 1933.55) | 63.21 (27.45 to 104.08) | 6.86 (3.06 to 11.13) | -2.13 (-2.40 to -1.87) |
| Tunisia | 4634.63 (3416.13 to 5918.47) | 103.02 (75.64 to 131.35) | 12.58 (9.47 to 15.82) | 7785.75 (5166.54 to 10961.15) | 66.46 (44.65 to 93.25) | 7.65 (5.26 to 10.06) | -1.50 (-1.72 to -1.28) |
| Türkiye | 43000.29 (32462.07 to 54931.08) | 127.13 (96.36 to 164.83) | 12.19 (9.28 to 15.62) | 57282.02 (43676.60 to 72185.26) | 68.21 (52.17 to 85.51) | 8.82 (6.97 to 10.60) | -1.71 (-2.14 to -1.28) |
| Turkmenistan | 2478.06 (858.03 to 4521.32) | 118.51 (36.49 to 220.93) | 10.61 (3.27 to 19.69) | 3647.02 (2207.78 to 5589.57) | 104.51 (63.05 to 160.45) | 9.09 (5.65 to 13.37) | -0.92 (-1.22 to -0.61) |
| Tuvalu | 18.88 (13.92 to 24.09) | 299.78 (229.33 to 370.32) | 18.09 (14.34 to 21.69) | 5.78 (3.41 to 8.88) | 63.93 (37.90 to 98.28) | 5.55 (3.29 to 8.49) | -5.35 (-5.50 to -5.21) |
| Uganda | 23993.56 (16788.37 to 31183.31) | 225.88 (170.23 to 279.63) | 10.59 (8.06 to 13.07) | 28860.70 (21434.13 to 36476.33) | 160.00 (123.26 to 200.38) | 9.98 (7.74 to 12.34) | -1.55 (-1.78 to -1.31) |
| Ukraine | 111819.91 (61255.34 to 162178.11) | 174.33 (96.04 to 252.68) | 17.53 (9.64 to 25.38) | 61576.09 (35407.24 to 96211.60) | 78.21 (45.26 to 122.09) | 7.78 (4.57 to 11.57) | -3.51 (-4.07 to -2.94) |
| United Arab Emirates | 697.53 (525.80 to 890.73) | 181.27 (135.94 to 232.95) | 17.52 (13.92 to 20.95) | 1891.75 (1385.56 to 2436.14) | 114.23 (85.20 to 145.03) | 13.93 (11.16 to 17.19) | 0.35 (-0.32 to 1.02) |
| United Kingdom | 61023.30 (29667.37 to 98016.24) | 65.30 (32.06 to 104.53) | 9.13 (4.47 to 14.61) | 16553.36 (10757.59 to 22842.24) | 11.30 (7.42 to 15.50) | 2.30 (1.51 to 3.14) | -6.20 (-6.47 to -5.93) |
| United Republic of Tanzania | 40807.90 (24038.91 to 54203.68) | 222.97 (151.20 to 278.88) | 13.34 (9.32 to 16.69) | 46785.94 (36548.27 to 57483.78) | 165.47 (130.46 to 207.13) | 11.43 (9.36 to 13.53) | -1.09 (-1.16 to -1.02) |
| United States of America | 136249.42 (58536.92 to 229174.43) | 41.63 (18.08 to 69.72) | 6.17 (2.68 to 10.34) | 50084.66 (24665.14 to 78677.80) | 8.20 (4.12 to 12.77) | 1.35 (0.68 to 2.11) | -5.72 (-6.17 to -5.26) |
| United States Virgin Islands | 16.15 (1.43 to 41.25) | 23.44 (1.59 to 61.23) | 2.77 (0.20 to 7.25) | 18.41 (8.39 to 29.76) | 10.86 (4.96 to 17.39) | 1.77 (0.79 to 2.87) | -2.39 (-2.75 to -2.04) |
| Uruguay | 2014.39 (793.98 to 3347.12) | 53.04 (21.17 to 87.74) | 6.61 (2.64 to 10.93) | 1068.71 (412.16 to 1820.97) | 17.30 (6.88 to 29.38) | 2.50 (1.00 to 4.22) | -3.85 (-4.08 to -3.62) |
| Uzbekistan | 24032.05 (16358.96 to 32422.33) | 186.16 (129.65 to 245.95) | 20.46 (14.25 to 26.84) | 32383.28 (24156.34 to 41752.61) | 152.77 (112.84 to 197.48) | 17.95 (13.79 to 22.65) | -0.65 (-0.99 to -0.31) |
| Vanuatu | 231.39 (184.71 to 288.29) | 396.29 (322.34 to 475.74) | 27.13 (23.72 to 30.87) | 477.40 (384.53 to 571.94) | 321.20 (262.18 to 379.08) | 23.34 (19.11 to 27.47) | -0.77 (-0.82 to -0.72) |
| Venezuela (Bolivarian Republic of) | 7275.70 (4303.05 to 10723.48) | 73.40 (40.40 to 112.18) | 9.34 (5.14 to 14.28) | 11877.61 (7069.25 to 18251.19) | 42.28 (25.50 to 64.95) | 4.29 (2.65 to 6.30) | -1.90 (-2.05 to -1.74) |
| Viet Nam | 94444.25 (75709.75 to 115507.17) | 231.42 (186.31 to 284.62) | 23.64 (20.09 to 27.10) | 98692.80 (71006.11 to 126493.52) | 117.31 (84.36 to 149.56) | 14.65 (10.90 to 18.33) | -2.25 (-2.47 to -2.03) |
| Yemen | 22079.03 (16251.90 to 28483.30) | 308.97 (234.66 to 387.78) | 20.72 (17.10 to 24.09) | 25751.98 (19311.89 to 34477.19) | 193.38 (141.09 to 257.92) | 14.36 (11.06 to 18.20) | -1.68 (-1.77 to -1.58) |
| Zambia | 11142.40 (6877.52 to 14988.79) | 233.13 (172.70 to 292.76) | 11.69 (8.70 to 14.34) | 13624.11 (9805.01 to 17718.05) | 188.79 (138.63 to 239.54) | 8.81 (6.95 to 10.81) | -0.81 (-0.99 to -0.64) |
| Zimbabwe | 8580.97 (5886.27 to 10931.80) | 176.39 (127.48 to 222.64) | 12.79 (9.47 to 15.91) | 15041.32 (10753.60 to 19902.16) | 212.11 (159.92 to 271.00) | 8.18 (6.26 to 10.07) | 1.48 (0.95 to 2.02) |

Table S18. Number and age-standardised rates of DALYs attributable to particulate matter pollution in 1990 and 2021, and estimated annual percentage changes from 1990 to 2021, by country.

| **Cause of DALYs** | **1990** |  |  | **2021** |  |  | **1990–2021** |
| --- | --- | --- | --- | --- | --- | --- | --- |
|  | **Number of cases** | **Age-standardised rates per 100 000 people** | **Age-standardised PAF (%)** | **Number of cases** | **Age-standardised rates per 100 000 people** | **Age-standardised PAF (%)** | **EAPC in age-standardised rates (%)** |
| Afghanistan | 1556953.07 (1054586.77 to 2048999.27) | 13517.28 (9934.27 to 16895.39) | 14.79 (11.15 to 18.11) | 1395809.78 (1039199.67 to 1783666.76) | 7566.53 (6042.23 to 9207.11) | 10.29 (8.52 to 12.03) | -1.95 (-2.16 to -1.75) |
| Albania | 122338.32 (87501.20 to 152720.76) | 4992.69 (3866.49 to 5997.47) | 14.75 (11.22 to 17.96) | 49107.12 (32340.80 to 72848.20) | 1277.50 (853.22 to 1842.83) | 4.79 (3.17 to 6.92) | -4.58 (-4.84 to -4.32) |
| Algeria | 458029.60 (317281.93 to 615981.07) | 2706.06 (1960.60 to 3446.19) | 6.52 (4.72 to 8.47) | 585559.20 (426597.90 to 755183.61) | 1740.92 (1262.61 to 2262.83) | 5.40 (3.92 to 6.87) | -1.61 (-1.81 to -1.41) |
| American Samoa | 220.98 (20.25 to 639.84) | 737.48 (46.04 to 2278.27) | 2.02 (0.12 to 6.32) | 374.22 (52.65 to 779.85) | 791.78 (120.93 to 1628.53) | 2.21 (0.37 to 4.55) | -0.01 (-0.23 to 0.21) |
| Andorra | 374.22 (163.24 to 634.36) | 698.65 (317.79 to 1164.55) | 2.99 (1.33 to 4.90) | 264.85 (144.90 to 410.56) | 171.59 (93.44 to 265.44) | 0.91 (0.52 to 1.37) | -4.27 (-4.59 to -3.95) |
| Angola | 1186197.47 (737228.28 to 1642580.71) | 9611.48 (6671.43 to 12389.41) | 9.42 (6.74 to 11.91) | 828832.21 (556035.39 to 1156523.52) | 3662.42 (2569.12 to 4834.08) | 5.86 (4.32 to 7.42) | -3.52 (-3.71 to -3.34) |
| Antigua and Barbuda | 786.09 (257.56 to 1485.09) | 1445.49 (469.23 to 2722.35) | 4.77 (1.51 to 8.67) | 1014.81 (470.21 to 1604.90) | 1027.60 (499.12 to 1614.71) | 3.62 (1.78 to 5.70) | -1.23 (-1.42 to -1.03) |
| Argentina | 559350.61 (322498.53 to 823117.97) | 1754.46 (1001.85 to 2585.35) | 5.19 (2.87 to 7.68) | 345946.16 (193257.13 to 520368.93) | 659.77 (373.26 to 964.98) | 2.42 (1.39 to 3.60) | -3.09 (-3.34 to -2.85) |
| Armenia | 117242.71 (74356.49 to 163972.38) | 4065.90 (2561.52 to 5702.01) | 10.90 (6.83 to 14.96) | 85376.18 (62527.27 to 111181.13) | 2137.51 (1586.27 to 2747.97) | 7.68 (5.72 to 9.80) | -2.46 (-2.66 to -2.25) |
| Australia | 64405.38 (6165.63 to 165792.53) | 343.82 (38.21 to 864.02) | 1.29 (0.14 to 3.22) | 82283.74 (51526.01 to 117573.56) | 188.44 (117.30 to 264.96) | 0.97 (0.60 to 1.40) | -2.42 (-3.01 to -1.81) |
| Austria | 148983.24 (81927.85 to 222793.11) | 1307.68 (728.93 to 1914.05) | 4.79 (2.74 to 7.06) | 52831.91 (38335.54 to 68262.99) | 292.95 (214.96 to 376.67) | 1.49 (1.10 to 1.95) | -5.01 (-5.18 to -4.84) |
| Azerbaijan | 369064.55 (197865.65 to 563024.45) | 5624.37 (3126.38 to 8347.17) | 12.05 (6.55 to 17.79) | 213185.15 (129125.78 to 304253.67) | 2427.19 (1509.07 to 3432.20) | 6.53 (4.09 to 9.35) | -3.16 (-3.47 to -2.85) |
| Bahamas | 2748.89 (831.58 to 4716.38) | 1529.39 (405.22 to 2717.81) | 4.23 (1.12 to 7.52) | 3830.61 (1475.12 to 6596.64) | 984.84 (395.39 to 1692.23) | 2.57 (1.07 to 4.25) | -1.36 (-1.51 to -1.21) |
| Bahrain | 13225.89 (10946.26 to 15325.00) | 6495.56 (5311.99 to 7509.83) | 15.73 (13.06 to 18.16) | 23816.78 (19075.20 to 28763.56) | 2938.15 (2398.59 to 3491.32) | 9.28 (7.64 to 10.88) | -2.98 (-3.28 to -2.68) |
| Bangladesh | 13042339.70 (8941219.51 to 16535320.54) | 11135.33 (8719.71 to 13206.55) | 16.15 (12.75 to 19.14) | 6900920.51 (5432325.27 to 8554048.30) | 5114.68 (4047.16 to 6317.54) | 14.63 (12.25 to 17.10) | -2.46 (-2.63 to -2.30) |
| Barbados | 4495.79 (1694.17 to 7655.96) | 1650.46 (699.97 to 2761.05) | 5.37 (2.20 to 8.85) | 5281.87 (2704.61 to 8021.86) | 1181.85 (653.50 to 1778.53) | 4.31 (2.40 to 6.22) | -1.05 (-1.22 to -0.88) |
| Belarus | 401419.61 (233881.04 to 567247.43) | 3305.19 (1939.73 to 4645.16) | 9.35 (5.55 to 13.39) | 217779.29 (151136.57 to 294832.56) | 1389.70 (967.71 to 1875.18) | 3.96 (2.89 to 5.36) | -3.59 (-4.07 to -3.11) |
| Belgium | 197035.47 (109822.22 to 298251.28) | 1343.32 (746.41 to 2012.57) | 5.00 (2.69 to 7.51) | 64584.12 (46705.85 to 84682.72) | 290.90 (208.13 to 376.74) | 1.43 (1.05 to 1.90) | -4.92 (-5.18 to -4.65) |
| Belize | 4293.36 (2835.30 to 6045.46) | 2653.29 (1775.94 to 3708.80) | 7.76 (5.21 to 10.75) | 4967.91 (2984.35 to 7045.27) | 1570.01 (924.97 to 2242.12) | 4.84 (2.98 to 6.82) | -1.86 (-2.18 to -1.54) |
| Benin | 580326.02 (349631.41 to 781459.30) | 9115.56 (6192.04 to 11625.01) | 11.51 (7.80 to 14.54) | 706463.19 (506537.74 to 940914.50) | 5699.63 (4386.53 to 7158.59) | 10.09 (8.06 to 12.28) | -1.39 (-1.48 to -1.29) |
| Bermuda | 382.86 (14.47 to 949.89) | 641.70 (32.23 to 1574.82) | 2.12 (0.11 to 5.21) | 222.85 (46.13 to 415.72) | 174.99 (39.78 to 321.56) | 0.79 (0.18 to 1.43) | -4.75 (-5.13 to -4.37) |
| Bhutan | 50335.46 (34668.73 to 65809.68) | 8136.60 (6117.88 to 10295.57) | 12.46 (9.71 to 14.85) | 15827.43 (11479.04 to 20283.01) | 2652.36 (1932.76 to 3387.75) | 8.25 (6.53 to 10.18) | -3.96 (-4.17 to -3.75) |
| Bolivia (Plurinational State of) | 474224.39 (256015.01 to 679940.36) | 7320.25 (4621.03 to 10005.62) | 11.97 (7.44 to 15.72) | 178540.83 (122403.60 to 252370.68) | 1896.38 (1306.10 to 2660.34) | 3.96 (2.82 to 5.28) | -4.40 (-4.49 to -4.30) |
| Bosnia and Herzegovina | 184663.10 (162700.84 to 207368.56) | 4837.92 (4262.87 to 5436.71) | 15.52 (13.47 to 17.95) | 115375.09 (85789.09 to 155296.32) | 1941.11 (1462.48 to 2584.21) | 7.12 (5.42 to 9.17) | -3.29 (-3.46 to -3.12) |
| Botswana | 64781.14 (44989.01 to 87226.55) | 6246.96 (4357.73 to 8363.41) | 10.41 (7.46 to 13.38) | 38697.63 (24729.58 to 55623.14) | 2247.00 (1454.72 to 3233.50) | 3.36 (2.25 to 4.74) | -3.04 (-3.27 to -2.81) |
| Brazil | 3273191.52 (2216206.87 to 4448906.59) | 2950.31 (1947.93 to 4017.49) | 6.87 (4.50 to 9.40) | 1693619.34 (1035318.97 to 2352528.23) | 707.86 (439.95 to 969.66) | 2.15 (1.33 to 2.96) | -4.62 (-4.75 to -4.49) |
| Brunei Darussalam | 816.18 (126.73 to 1859.99) | 685.45 (74.42 to 1630.39) | 2.01 (0.22 to 4.95) | 1436.18 (351.38 to 2679.90) | 409.47 (104.21 to 753.15) | 1.61 (0.42 to 3.09) | -1.31 (-2.03 to -0.59) |
| Bulgaria | 430501.92 (271793.81 to 601517.03) | 4179.89 (2621.78 to 5814.56) | 10.77 (6.68 to 15.08) | 212661.56 (161998.17 to 301410.79) | 1624.43 (1242.00 to 2266.89) | 4.29 (3.26 to 5.97) | -3.51 (-3.88 to -3.14) |
| Burkina Faso | 1208454.64 (696317.59 to 1679281.74) | 8938.27 (5558.16 to 11881.75) | 9.34 (5.68 to 12.41) | 1300388.79 (870050.18 to 1727980.81) | 5617.53 (4120.00 to 7132.49) | 8.71 (6.48 to 10.69) | -1.23 (-1.32 to -1.14) |
| Burundi | 615132.62 (397124.24 to 815925.41) | 10593.44 (7731.85 to 13430.84) | 10.40 (7.61 to 12.86) | 552510.14 (390359.81 to 708854.13) | 5696.14 (4379.44 to 7104.28) | 9.79 (7.71 to 11.48) | -2.27 (-2.38 to -2.15) |
| Cabo Verde | 14271.39 (10628.94 to 18060.60) | 4152.15 (3215.48 to 5088.34) | 10.01 (7.80 to 11.95) | 11987.97 (8680.25 to 15390.27) | 2732.83 (1993.10 to 3483.68) | 8.62 (6.53 to 10.57) | -1.74 (-2.14 to -1.35) |
| Cambodia | 1340490.54 (715323.80 to 1890020.58) | 11656.93 (7523.90 to 15230.71) | 16.08 (10.64 to 20.50) | 687811.34 (520029.01 to 895572.10) | 5364.64 (4188.91 to 6821.79) | 12.82 (10.30 to 15.29) | -2.70 (-2.83 to -2.58) |
| Cameroon | 856025.21 (560692.99 to 1206802.70) | 7168.01 (5193.74 to 9352.38) | 10.13 (7.25 to 12.93) | 1209277.34 (846623.74 to 1659491.60) | 5199.13 (3919.80 to 6774.26) | 8.37 (6.45 to 10.29) | -0.92 (-1.18 to -0.65) |
| Canada | 170898.58 (52267.00 to 309388.12) | 544.50 (174.77 to 980.81) | 2.18 (0.70 to 4.01) | 71707.23 (28326.91 to 127708.89) | 109.56 (42.42 to 188.25) | 0.53 (0.21 to 0.91) | -5.43 (-5.80 to -5.06) |
| Central African Republic | 394877.97 (243490.04 to 544715.51) | 12821.75 (8981.36 to 16759.71) | 11.45 (7.98 to 14.70) | 412195.74 (269960.68 to 556176.61) | 9427.06 (6741.52 to 12143.36) | 9.62 (7.23 to 11.75) | -0.97 (-1.03 to -0.91) |
| Chad | 803033.89 (492396.18 to 1091948.64) | 9245.15 (6046.53 to 11832.01) | 10.53 (6.85 to 13.39) | 1443609.03 (900194.88 to 1968402.53) | 7494.18 (5239.26 to 9805.70) | 10.65 (7.82 to 12.89) | -0.66 (-0.81 to -0.50) |
| Chile | 237974.18 (170374.26 to 302408.39) | 2282.39 (1640.84 to 2922.91) | 7.13 (4.90 to 9.31) | 162739.10 (116513.85 to 209023.84) | 678.31 (491.31 to 863.62) | 2.83 (2.04 to 3.62) | -3.69 (-3.90 to -3.49) |
| China | 66320218.91 (53455165.84 to 77459839.36) | 8142.70 (6796.79 to 9441.41) | 18.91 (15.74 to 21.57) | 46676902.76 (36578694.24 to 59744053.28) | 2436.88 (1919.69 to 3069.45) | 10.74 (8.73 to 13.09) | -4.08 (-4.31 to -3.85) |
| Colombia | 685694.80 (498721.14 to 879507.33) | 2787.69 (2051.52 to 3477.16) | 7.50 (5.47 to 9.33) | 348430.81 (230219.31 to 479055.02) | 663.35 (434.83 to 914.46) | 2.39 (1.63 to 3.30) | -4.79 (-4.95 to -4.63) |
| Comoros | 48262.61 (33280.90 to 66229.16) | 8874.52 (6674.81 to 11553.52) | 12.63 (9.34 to 16.13) | 28346.42 (22033.76 to 36186.20) | 4678.50 (3709.35 to 5879.27) | 10.01 (7.98 to 12.05) | -2.24 (-2.36 to -2.13) |
| Congo | 152513.22 (105818.21 to 202529.26) | 8361.19 (6343.28 to 10600.65) | 10.10 (7.69 to 12.36) | 148154.16 (110779.84 to 194265.09) | 4540.89 (3442.42 to 5823.12) | 7.56 (5.85 to 9.35) | -2.30 (-2.50 to -2.10) |
| Cook Islands | 151.67 (26.00 to 355.95) | 1093.27 (165.45 to 2633.44) | 2.73 (0.43 to 6.51) | 102.62 (3.87 to 243.97) | 423.94 (22.52 to 996.76) | 1.59 (0.09 to 3.72) | -3.27 (-3.74 to -2.79) |
| Costa Rica | 34722.37 (23608.94 to 45230.43) | 1603.83 (1030.00 to 2098.90) | 6.04 (3.87 to 7.79) | 27152.67 (18749.74 to 37040.29) | 524.32 (363.14 to 710.53) | 2.00 (1.41 to 2.73) | -3.57 (-3.75 to -3.40) |
| Côte d'Ivoire | 1168975.70 (775107.19 to 1571204.82) | 8419.04 (6130.25 to 10720.97) | 10.57 (7.89 to 13.47) | 1337283.13 (969650.74 to 1785962.46) | 5807.76 (4447.12 to 7341.08) | 10.25 (7.99 to 12.60) | -1.12 (-1.34 to -0.90) |
| Croatia | 140574.08 (81671.27 to 204466.65) | 2562.92 (1519.99 to 3701.68) | 7.71 (4.51 to 11.19) | 68901.77 (52815.27 to 86793.80) | 789.80 (605.47 to 990.29) | 3.26 (2.48 to 4.15) | -3.84 (-4.06 to -3.62) |
| Cuba | 168319.93 (76115.72 to 292077.51) | 1695.66 (776.67 to 2928.00) | 5.83 (2.79 to 9.84) | 197415.63 (103756.37 to 305458.79) | 1043.15 (554.78 to 1600.75) | 3.56 (1.90 to 5.59) | -1.63 (-1.90 to -1.36) |
| Cyprus | 11718.03 (6044.83 to 17885.32) | 1823.59 (932.71 to 2807.88) | 5.71 (2.93 to 8.58) | 10053.39 (7458.35 to 12949.99) | 535.80 (403.30 to 688.15) | 2.70 (2.03 to 3.43) | -4.33 (-4.52 to -4.14) |
| Czechia | 362305.40 (201419.52 to 529158.24) | 2766.05 (1586.61 to 3995.98) | 8.09 (4.68 to 11.80) | 138763.11 (106460.64 to 175537.53) | 657.95 (508.87 to 834.82) | 2.74 (2.08 to 3.49) | -4.27 (-4.51 to -4.04) |
| Democratic People's Republic of Korea | 1146494.96 (875364.28 to 1475310.64) | 7028.53 (5564.95 to 8809.19) | 18.92 (16.04 to 21.62) | 1677311.13 (1336474.87 to 2041583.91) | 5410.71 (4332.05 to 6625.60) | 18.28 (15.95 to 20.73) | -0.78 (-0.95 to -0.60) |
| Democratic Republic of the Congo | 3465427.96 (2114899.96 to 4824588.67) | 8460.85 (5963.44 to 10824.07) | 9.81 (6.87 to 12.46) | 3378502.12 (2481784.92 to 4405195.87) | 5841.77 (4357.63 to 7465.07) | 9.57 (7.52 to 11.61) | -1.17 (-1.26 to -1.09) |
| Denmark | 104070.71 (51044.96 to 168354.55) | 1326.47 (666.06 to 2120.68) | 4.68 (2.28 to 7.24) | 27943.43 (17597.49 to 38731.76) | 245.15 (156.95 to 338.45) | 1.24 (0.79 to 1.77) | -5.71 (-5.93 to -5.49) |
| Djibouti | 19439.24 (12579.31 to 26534.85) | 5388.30 (3810.93 to 7068.33) | 8.85 (6.38 to 11.00) | 27048.60 (18675.13 to 38161.63) | 3261.49 (2236.27 to 4580.88) | 6.31 (4.48 to 8.25) | -1.66 (-1.78 to -1.55) |
| Dominica | 1788.60 (1230.62 to 2367.31) | 2822.54 (1916.40 to 3748.34) | 8.43 (5.83 to 11.08) | 1193.49 (618.71 to 1822.16) | 1692.45 (943.18 to 2519.37) | 4.44 (2.43 to 6.55) | -1.39 (-1.79 to -0.99) |
| Dominican Republic | 214545.42 (150348.03 to 277131.42) | 3367.06 (2456.61 to 4244.74) | 8.57 (6.24 to 10.63) | 164517.05 (88350.83 to 255170.94) | 1619.21 (866.45 to 2518.46) | 4.96 (2.65 to 7.55) | -1.82 (-2.14 to -1.50) |
| Ecuador | 247937.44 (174032.67 to 322065.70) | 3018.73 (2170.64 to 3849.28) | 8.29 (5.87 to 10.62) | 142271.64 (93199.49 to 198958.60) | 888.69 (583.03 to 1233.80) | 2.87 (1.87 to 3.84) | -4.23 (-4.68 to -3.78) |
| Egypt | 3504149.18 (2418726.04 to 4582430.51) | 8020.61 (6368.56 to 9853.28) | 13.84 (10.94 to 16.85) | 3313814.37 (2585145.03 to 4157753.40) | 5287.62 (4151.34 to 6604.02) | 12.41 (9.87 to 15.02) | -1.02 (-1.22 to -0.81) |
| El Salvador | 167540.75 (122872.84 to 211329.12) | 3551.88 (2817.61 to 4236.39) | 8.19 (6.41 to 9.78) | 72623.42 (47968.50 to 99425.40) | 1165.20 (772.53 to 1594.64) | 3.47 (2.35 to 4.67) | -3.55 (-3.70 to -3.41) |
| Equatorial Guinea | 47563.80 (30352.42 to 66698.80) | 10284.84 (7254.22 to 13539.76) | 10.77 (7.83 to 13.50) | 22996.02 (14012.68 to 36008.72) | 2912.21 (1785.48 to 4491.61) | 4.79 (3.12 to 6.78) | -4.78 (-5.50 to -4.06) |
| Eritrea | 318739.81 (185197.55 to 434448.26) | 10054.77 (6670.65 to 13142.62) | 8.82 (6.08 to 11.23) | 253265.66 (168024.98 to 352963.84) | 5753.23 (4104.96 to 7672.29) | 9.57 (7.11 to 12.06) | -1.64 (-1.74 to -1.54) |
| Estonia | 38229.63 (16501.50 to 61623.32) | 1983.32 (892.89 to 3149.70) | 5.22 (2.34 to 8.23) | 4358.18 (1471.27 to 7972.80) | 166.30 (57.42 to 303.44) | 0.65 (0.22 to 1.19) | -9.26 (-9.82 to -8.70) |
| Eswatini | 41733.30 (27756.04 to 57070.71) | 6570.49 (4750.90 to 8440.80) | 10.68 (7.92 to 13.25) | 32024.83 (19785.79 to 49195.05) | 4362.33 (2622.05 to 6648.11) | 4.39 (2.81 to 6.30) | -0.83 (-1.39 to -0.28) |
| Ethiopia | 7342911.48 (4625731.82 to 9751812.39) | 11536.51 (7676.43 to 14721.99) | 10.18 (6.73 to 12.94) | 4085793.86 (3219496.75 to 5106986.50) | 4466.33 (3654.84 to 5364.56) | 8.60 (6.93 to 10.07) | -3.38 (-3.50 to -3.26) |
| Fiji | 26151.55 (17936.62 to 34421.73) | 5966.24 (4014.03 to 7811.90) | 13.05 (9.02 to 16.53) | 25356.32 (10426.40 to 41510.87) | 3247.26 (1336.98 to 5256.35) | 6.87 (2.94 to 10.85) | -2.02 (-2.20 to -1.83) |
| Finland | 37631.23 (6970.48 to 74786.35) | 547.04 (110.57 to 1064.28) | 1.95 (0.39 to 3.87) | 8208.37 (1200.20 to 17326.78) | 69.37 (12.85 to 141.04) | 0.35 (0.07 to 0.70) | -7.06 (-7.47 to -6.66) |
| France | 571619.40 (280626.10 to 915060.46) | 716.73 (358.34 to 1132.33) | 2.74 (1.37 to 4.35) | 245598.74 (168906.37 to 330655.01) | 187.05 (127.77 to 252.48) | 0.94 (0.63 to 1.31) | -4.32 (-4.60 to -4.05) |
| Gabon | 36257.36 (22942.46 to 51117.70) | 4077.62 (2634.66 to 5677.07) | 6.33 (4.17 to 8.65) | 32444.60 (21482.21 to 47587.49) | 2651.77 (1752.01 to 3876.54) | 4.87 (3.39 to 6.61) | -1.24 (-1.35 to -1.12) |
| Gambia | 94509.40 (64002.32 to 125092.99) | 8630.56 (6251.88 to 11007.34) | 12.71 (9.49 to 15.62) | 106558.98 (82576.87 to 133791.54) | 6494.15 (4965.68 to 7997.85) | 11.65 (9.53 to 13.56) | -0.95 (-1.06 to -0.85) |
| Georgia | 218055.30 (110358.15 to 325641.74) | 3910.46 (2021.67 to 5799.45) | 10.04 (5.09 to 14.97) | 90693.31 (57879.40 to 125621.12) | 1616.16 (1054.85 to 2214.43) | 4.76 (3.13 to 6.64) | -3.83 (-4.57 to -3.09) |
| Germany | 1758324.63 (945698.99 to 2675030.36) | 1433.56 (789.11 to 2158.29) | 5.17 (2.75 to 7.75) | 544628.66 (387864.98 to 719875.01) | 295.59 (210.02 to 391.49) | 1.41 (1.02 to 1.89) | -4.99 (-5.16 to -4.82) |
| Ghana | 1143193.98 (821030.35 to 1501116.72) | 7967.95 (6133.55 to 9914.50) | 11.79 (9.28 to 14.17) | 1189051.61 (913159.82 to 1526414.00) | 5018.88 (3938.21 to 6249.64) | 9.78 (7.90 to 11.67) | -1.27 (-1.44 to -1.10) |
| Greece | 212756.21 (120147.35 to 320102.34) | 1509.02 (865.97 to 2235.38) | 5.93 (3.42 to 8.83) | 132545.18 (103037.69 to 164576.95) | 579.31 (454.53 to 707.25) | 2.64 (2.03 to 3.34) | -3.33 (-3.66 to -3.01) |
| Greenland | 266.05 (41.90 to 631.53) | 710.44 (91.13 to 1778.44) | 1.39 (0.17 to 3.43) | 145.40 (11.79 to 368.25) | 225.19 (25.90 to 553.05) | 0.71 (0.08 to 1.69) | -4.17 (-4.59 to -3.75) |
| Grenada | 2542.33 (1617.21 to 3607.83) | 3231.16 (1989.90 to 4600.69) | 8.53 (5.37 to 12.08) | 1818.02 (958.60 to 2770.20) | 1718.42 (944.32 to 2606.98) | 4.54 (2.46 to 6.94) | -1.80 (-2.04 to -1.56) |
| Guam | 604.12 (73.00 to 1467.06) | 706.38 (51.30 to 1768.31) | 2.39 (0.17 to 6.04) | 1274.23 (673.63 to 1884.65) | 649.22 (339.04 to 964.96) | 2.39 (1.27 to 3.53) | 0.38 (-0.29 to 1.06) |
| Guatemala | 548697.34 (340925.70 to 722460.43) | 6242.90 (4013.53 to 7950.27) | 10.56 (6.80 to 13.34) | 281831.42 (188112.53 to 383263.94) | 2377.93 (1595.36 to 3196.73) | 5.81 (3.99 to 7.43) | -3.16 (-3.32 to -2.99) |
| Guinea | 988052.37 (587171.53 to 1389575.27) | 11020.50 (6944.29 to 14782.67) | 11.97 (7.62 to 15.81) | 795577.80 (544476.68 to 1093360.13) | 6777.68 (5010.60 to 8888.47) | 10.45 (8.08 to 12.81) | -1.29 (-1.39 to -1.20) |
| Guinea-Bissau | 136372.13 (90983.04 to 182235.09) | 12501.57 (9071.06 to 16290.10) | 12.09 (8.85 to 15.29) | 111230.95 (83506.67 to 140783.79) | 8039.92 (6189.70 to 9800.33) | 11.23 (9.20 to 13.34) | -1.31 (-1.41 to -1.22) |
| Guyana | 30202.68 (20478.84 to 40589.04) | 5054.35 (3296.70 to 6969.55) | 9.15 (6.05 to 12.91) | 18040.59 (10018.45 to 26694.66) | 2722.75 (1497.75 to 4029.34) | 5.21 (2.97 to 7.71) | -1.78 (-1.89 to -1.67) |
| Haiti | 659986.78 (451068.82 to 860406.96) | 10946.56 (8575.80 to 13522.05) | 12.77 (9.96 to 15.20) | 687551.90 (509699.45 to 868147.59) | 7241.07 (5504.49 to 9123.74) | 10.96 (8.67 to 13.10) | -1.10 (-1.20 to -1.00) |
| Honduras | 176292.27 (125991.03 to 226292.77) | 4311.16 (3487.40 to 5135.06) | 9.87 (7.86 to 11.65) | 212994.18 (166287.40 to 262458.00) | 3252.03 (2569.68 to 3978.34) | 7.81 (6.30 to 9.51) | -0.69 (-0.87 to -0.50) |
| Hungary | 419464.49 (245568.64 to 630966.86) | 3156.98 (1886.14 to 4649.67) | 8.07 (4.79 to 12.05) | 172636.28 (127744.15 to 251683.29) | 917.27 (681.75 to 1326.33) | 3.23 (2.42 to 4.70) | -4.00 (-4.22 to -3.78) |
| Iceland | 907.27 (117.56 to 2030.91) | 318.67 (45.65 to 708.49) | 1.33 (0.19 to 2.99) | 369.54 (75.77 to 781.68) | 65.56 (15.23 to 135.76) | 0.36 (0.08 to 0.76) | -5.46 (-5.82 to -5.09) |
| India | 67348076.79 (50893602.16 to 81004721.91) | 8748.77 (7063.36 to 10170.82) | 13.69 (10.89 to 15.73) | 60941686.62 (51445179.38 to 70020271.07) | 5349.47 (4503.74 to 6173.93) | 12.68 (10.82 to 14.48) | -1.46 (-1.57 to -1.36) |
| Indonesia | 8458988.46 (6716928.63 to 10069282.77) | 6156.45 (5028.97 to 7228.74) | 11.65 (9.41 to 13.58) | 6522734.07 (4799000.89 to 8578958.43) | 2830.28 (2096.15 to 3669.67) | 7.42 (5.58 to 9.48) | -2.48 (-2.77 to -2.20) |
| Iran (Islamic Republic of) | 1455217.56 (1179812.27 to 1780142.69) | 3663.56 (3047.33 to 4296.81) | 7.78 (6.38 to 9.18) | 1200850.98 (994681.70 to 1393247.87) | 1612.37 (1335.78 to 1864.42) | 5.32 (4.38 to 6.30) | -2.39 (-2.52 to -2.27) |
| Iraq | 770698.96 (579656.73 to 990227.42) | 5279.32 (4101.33 to 6440.01) | 11.21 (9.07 to 13.52) | 880910.00 (664002.91 to 1127944.66) | 3509.68 (2639.74 to 4449.24) | 9.01 (7.07 to 10.97) | -1.48 (-1.61 to -1.35) |
| Ireland | 44998.15 (18504.96 to 76063.64) | 1135.22 (469.11 to 1909.59) | 3.98 (1.69 to 6.79) | 11689.61 (6254.59 to 17771.14) | 154.89 (83.41 to 235.12) | 0.80 (0.43 to 1.22) | -6.58 (-6.85 to -6.31) |
| Israel | 68912.81 (41901.46 to 96660.29) | 1448.91 (878.83 to 2038.05) | 5.84 (3.56 to 8.29) | 54331.01 (43823.75 to 66324.50) | 438.99 (351.35 to 538.45) | 2.44 (1.93 to 3.02) | -4.14 (-4.27 to -4.01) |
| Italy | 1090145.83 (659302.94 to 1542684.09) | 1331.71 (842.48 to 1845.47) | 5.13 (3.20 to 7.23) | 490878.52 (379570.21 to 611855.25) | 334.70 (263.16 to 414.51) | 1.78 (1.38 to 2.26) | -4.54 (-4.70 to -4.37) |
| Jamaica | 52819.66 (39180.17 to 66930.71) | 2551.36 (1876.04 to 3218.93) | 8.67 (6.29 to 11.06) | 34657.79 (21628.12 to 49611.36) | 1224.86 (760.16 to 1741.83) | 4.03 (2.65 to 5.61) | -1.98 (-2.42 to -1.54) |
| Japan | 810583.25 (180178.95 to 1683068.31) | 513.07 (122.05 to 1052.14) | 2.40 (0.58 to 4.92) | 931225.37 (527738.28 to 1373171.18) | 268.15 (155.31 to 387.92) | 1.66 (0.94 to 2.49) | -2.22 (-2.51 to -1.92) |
| Jordan | 61151.75 (43088.55 to 81874.84) | 2578.72 (2032.79 to 3173.15) | 7.33 (5.84 to 8.93) | 124113.76 (92241.01 to 157366.90) | 1586.54 (1193.47 to 1999.73) | 5.65 (4.44 to 6.84) | -1.90 (-2.18 to -1.62) |
| Kazakhstan | 446353.08 (230693.48 to 733153.42) | 3288.15 (1626.48 to 5341.97) | 7.71 (3.79 to 12.34) | 312382.24 (226701.44 to 405443.95) | 1862.89 (1347.72 to 2425.57) | 4.85 (3.62 to 6.31) | -2.84 (-3.34 to -2.34) |
| Kenya | 1260243.02 (834378.85 to 1697893.57) | 5033.86 (3527.06 to 6393.84) | 8.52 (6.03 to 10.69) | 1218044.76 (954209.81 to 1479975.04) | 3862.44 (3009.31 to 4680.96) | 7.26 (5.72 to 8.58) | -0.49 (-0.70 to -0.27) |
| Kiribati | 5353.43 (4075.49 to 6605.06) | 9401.90 (7679.49 to 11314.50) | 13.88 (11.57 to 16.04) | 4918.20 (3728.60 to 6525.60) | 5832.43 (4465.12 to 7612.48) | 11.16 (9.03 to 13.43) | -1.63 (-1.81 to -1.44) |
| Kuwait | 23727.47 (18722.81 to 28113.15) | 2907.38 (2386.02 to 3407.40) | 9.40 (7.67 to 11.20) | 61198.42 (46797.69 to 76217.14) | 1851.82 (1425.80 to 2301.71) | 8.48 (6.85 to 10.14) | -0.92 (-1.37 to -0.46) |
| Kyrgyzstan | 247277.37 (163754.57 to 344188.16) | 6329.06 (4558.95 to 8361.70) | 13.72 (9.71 to 17.92) | 148866.06 (114453.04 to 184274.45) | 3016.51 (2321.13 to 3711.22) | 9.16 (7.30 to 10.95) | -2.69 (-3.15 to -2.22) |
| Lao People's Democratic Republic | 648665.51 (388422.73 to 901446.59) | 14553.57 (10475.42 to 19003.73) | 16.54 (12.08 to 20.48) | 313533.79 (224654.33 to 411593.81) | 5834.81 (4130.73 to 7552.19) | 13.74 (10.10 to 16.85) | -3.09 (-3.21 to -2.97) |
| Latvia | 112930.17 (65794.93 to 160151.42) | 3310.80 (1960.94 to 4672.41) | 8.57 (5.08 to 12.17) | 28656.79 (19255.91 to 41262.06) | 738.84 (499.21 to 1044.84) | 2.39 (1.61 to 3.45) | -5.89 (-6.37 to -5.40) |
| Lebanon | 51885.81 (35759.54 to 73221.81) | 2087.40 (1436.57 to 2908.19) | 5.05 (3.54 to 6.86) | 65824.87 (43901.74 to 89530.95) | 1102.44 (739.54 to 1483.87) | 3.72 (2.58 to 4.88) | -1.99 (-2.24 to -1.74) |
| Lesotho | 100422.69 (67884.08 to 131658.59) | 6484.83 (4663.22 to 8102.65) | 10.71 (7.81 to 13.01) | 95088.11 (68424.82 to 123175.47) | 7007.01 (5126.66 to 9041.75) | 5.94 (4.59 to 7.46) | 0.94 (0.55 to 1.32) |
| Liberia | 367654.54 (213975.09 to 515302.09) | 10621.59 (7124.76 to 14129.21) | 9.85 (6.65 to 12.87) | 219237.58 (160548.88 to 297246.15) | 5723.55 (4363.08 to 7407.31) | 9.75 (7.91 to 11.66) | -2.03 (-2.26 to -1.80) |
| Libya | 70590.17 (51607.95 to 92331.94) | 2407.42 (1822.66 to 3111.88) | 6.89 (5.38 to 8.56) | 132052.04 (98393.49 to 176809.50) | 2485.80 (1851.23 to 3297.70) | 6.63 (5.06 to 8.37) | 0.53 (0.25 to 0.83) |
| Lithuania | 103690.03 (55369.01 to 155590.08) | 2391.26 (1294.70 to 3574.95) | 6.74 (3.61 to 10.18) | 30185.35 (18644.15 to 42502.99) | 530.82 (331.44 to 739.76) | 1.77 (1.11 to 2.55) | -5.39 (-5.73 to -5.05) |
| Luxembourg | 5750.94 (2623.97 to 9231.80) | 1105.28 (514.30 to 1767.25) | 3.90 (1.79 to 6.26) | 1747.72 (1044.68 to 2500.83) | 168.97 (102.94 to 242.90) | 0.90 (0.53 to 1.30) | -5.87 (-6.15 to -5.59) |
| Madagascar | 1180437.97 (758686.46 to 1547023.70) | 9626.97 (6853.75 to 11745.28) | 12.38 (8.85 to 14.94) | 1289158.73 (972534.23 to 1618964.41) | 6804.67 (5256.83 to 8379.48) | 11.62 (9.26 to 13.54) | -1.12 (-1.17 to -1.07) |
| Malawi | 1201524.14 (708046.71 to 1649412.13) | 9029.87 (5921.51 to 11592.26) | 8.85 (5.69 to 11.38) | 735611.68 (542525.02 to 950339.45) | 5620.30 (4362.75 to 6972.54) | 8.04 (6.40 to 9.56) | -1.71 (-1.86 to -1.56) |
| Malaysia | 319981.31 (181097.19 to 453663.53) | 2916.20 (1578.38 to 4243.80) | 8.74 (4.61 to 12.66) | 404526.51 (288020.46 to 532452.41) | 1449.76 (1038.48 to 1913.35) | 4.69 (3.23 to 6.26) | -2.31 (-2.52 to -2.09) |
| Maldives | 12417.01 (9202.15 to 15761.00) | 7227.05 (5625.44 to 8524.82) | 14.80 (11.53 to 17.46) | 2279.52 (1376.20 to 3381.49) | 673.06 (410.13 to 1001.70) | 3.05 (1.85 to 4.44) | -8.63 (-9.05 to -8.21) |
| Mali | 1202721.56 (877276.34 to 1503657.46) | 9436.88 (7239.64 to 11467.57) | 9.49 (7.30 to 11.45) | 1680563.45 (1235339.44 to 2166135.22) | 6294.19 (4976.15 to 7695.84) | 9.22 (7.58 to 10.94) | -1.19 (-1.25 to -1.12) |
| Malta | 5314.69 (2525.97 to 8166.89) | 1310.64 (648.11 to 1992.41) | 5.14 (2.54 to 7.81) | 3517.12 (2549.30 to 4630.11) | 391.82 (282.34 to 518.36) | 2.04 (1.47 to 2.68) | -4.01 (-4.27 to -3.75) |
| Marshall Islands | 1397.84 (985.50 to 1926.90) | 5984.82 (4394.81 to 7979.59) | 10.78 (7.97 to 14.19) | 1859.27 (1230.26 to 2753.27) | 4745.10 (3181.71 to 6891.88) | 9.59 (6.82 to 12.89) | -0.46 (-0.65 to -0.28) |
| Mauritania | 172173.79 (126429.43 to 217235.92) | 8092.10 (6170.99 to 9978.29) | 12.71 (9.91 to 15.20) | 144219.25 (111179.10 to 178657.59) | 4416.75 (3479.07 to 5526.86) | 11.04 (8.82 to 12.91) | -2.02 (-2.07 to -1.97) |
| Mauritius | 10692.01 (5710.49 to 16640.27) | 1372.26 (737.74 to 2119.71) | 3.66 (1.88 to 5.72) | 11699.65 (4495.99 to 20475.56) | 697.65 (283.47 to 1164.97) | 2.21 (0.87 to 3.64) | -2.14 (-2.46 to -1.82) |
| Mexico | 1843882.31 (1279520.83 to 2418771.64) | 2766.97 (1979.79 to 3546.84) | 7.04 (5.10 to 9.07) | 1304386.82 (933741.26 to 1796200.07) | 1068.20 (770.55 to 1466.27) | 2.93 (2.09 to 3.98) | -3.23 (-3.40 to -3.06) |
| Micronesia (Federated States of) | 5738.13 (4202.06 to 7443.25) | 8809.91 (6832.00 to 10886.81) | 15.98 (12.73 to 18.88) | 3495.03 (2298.39 to 5094.39) | 4509.39 (2992.34 to 6491.53) | 10.22 (7.01 to 13.67) | -2.23 (-2.26 to -2.19) |
| Monaco | 310.55 (53.95 to 619.32) | 470.86 (90.38 to 916.13) | 1.96 (0.37 to 3.71) | 258.89 (134.87 to 396.78) | 283.60 (153.69 to 432.76) | 1.22 (0.67 to 1.86) | -1.42 (-2.37 to -0.46) |
| Mongolia | 169789.91 (91321.12 to 238005.46) | 8883.35 (6455.19 to 11208.98) | 15.68 (11.41 to 19.87) | 71067.30 (50619.42 to 92886.42) | 2949.22 (2148.69 to 3870.18) | 7.87 (5.74 to 10.44) | -3.99 (-4.34 to -3.63) |
| Montenegro | 17525.67 (11437.20 to 24045.97) | 2938.53 (1935.30 to 4009.61) | 10.35 (6.74 to 14.22) | 16063.15 (11357.41 to 26496.40) | 1762.08 (1254.70 to 2884.59) | 5.41 (3.87 to 8.90) | -1.96 (-2.29 to -1.64) |
| Morocco | 903871.36 (649116.02 to 1204379.94) | 4175.52 (3208.79 to 5265.34) | 8.81 (6.85 to 11.01) | 741588.84 (531361.90 to 958622.49) | 2227.92 (1607.23 to 2858.26) | 6.50 (4.96 to 8.18) | -1.87 (-2.09 to -1.66) |
| Mozambique | 1516485.15 (995656.57 to 2080897.95) | 8832.90 (6267.40 to 11495.79) | 9.13 (6.45 to 11.53) | 1439121.57 (1045227.45 to 1840962.48) | 6324.60 (4886.58 to 7820.42) | 8.03 (6.48 to 9.51) | -0.66 (-0.81 to -0.50) |
| Myanmar | 4508090.62 (2901498.17 to 6025705.58) | 13430.95 (9799.79 to 16871.69) | 16.89 (12.75 to 20.37) | 2882176.36 (2235517.20 to 3592833.67) | 6029.14 (4725.27 to 7458.18) | 13.93 (11.27 to 16.60) | -2.84 (-3.04 to -2.64) |
| Namibia | 72286.26 (50219.95 to 93119.32) | 6316.22 (4517.67 to 8059.92) | 10.90 (7.87 to 13.57) | 52302.14 (32563.63 to 81237.07) | 3175.88 (1959.76 to 4853.47) | 4.81 (3.09 to 7.02) | -2.26 (-2.61 to -1.91) |
| Nauru | 79.52 (6.97 to 245.09) | 1211.25 (64.65 to 3962.00) | 1.98 (0.11 to 6.50) | 83.71 (16.99 to 182.48) | 1173.51 (198.52 to 2596.77) | 2.07 (0.39 to 4.46) | -1.30 (-2.62 to 0.03) |
| Nepal | 2486503.60 (1631815.98 to 3269982.04) | 11355.85 (8349.27 to 14177.73) | 15.67 (11.86 to 18.99) | 1379478.45 (1121460.53 to 1668086.03) | 5726.96 (4707.00 to 6845.63) | 13.33 (11.48 to 15.40) | -2.07 (-2.19 to -1.96) |
| Netherlands | 257860.18 (139168.65 to 388080.50) | 1338.06 (735.04 to 1993.34) | 5.39 (2.99 to 8.10) | 96580.27 (69780.98 to 125688.53) | 288.36 (209.88 to 373.47) | 1.46 (1.08 to 1.95) | -5.26 (-5.51 to -5.00) |
| New Zealand | 12827.75 (1155.37 to 35883.58) | 339.14 (35.10 to 937.19) | 1.16 (0.12 to 3.23) | 12373.90 (5366.90 to 19990.25) | 156.90 (71.88 to 250.06) | 0.77 (0.35 to 1.24) | -2.90 (-3.32 to -2.48) |
| Nicaragua | 136797.60 (93904.59 to 176995.43) | 3548.91 (2826.68 to 4232.57) | 9.60 (7.38 to 11.54) | 78299.09 (61835.32 to 98460.64) | 1536.05 (1207.76 to 1926.80) | 4.78 (3.76 to 5.81) | -2.44 (-2.64 to -2.24) |
| Niger | 1544167.10 (681348.20 to 2287637.00) | 12059.50 (6230.26 to 16949.69) | 11.10 (5.74 to 15.34) | 1536917.40 (978417.12 to 2118014.25) | 5952.05 (4191.27 to 7847.32) | 10.00 (7.47 to 12.43) | -2.43 (-2.62 to -2.23) |
| Nigeria | 11516989.64 (6906385.39 to 15471083.32) | 9455.13 (6393.48 to 12142.24) | 11.16 (7.46 to 14.16) | 12797073.39 (9361248.52 to 16639381.34) | 5371.20 (4145.15 to 6714.04) | 9.32 (7.12 to 11.41) | -1.78 (-1.92 to -1.63) |
| Niue | 61.48 (27.05 to 97.58) | 2760.72 (1212.22 to 4393.42) | 6.93 (3.09 to 10.80) | 18.00 (3.17 to 37.75) | 955.40 (215.14 to 1925.01) | 2.11 (0.46 to 4.24) | -4.84 (-5.45 to -4.22) |
| North Macedonia | 91015.00 (66698.62 to 110822.86) | 5156.02 (3777.15 to 6267.08) | 13.81 (10.30 to 16.85) | 68126.83 (52249.45 to 87381.76) | 2391.46 (1876.91 to 3039.94) | 6.78 (5.30 to 8.70) | -2.64 (-3.09 to -2.19) |
| Northern Mariana Islands | 208.32 (26.66 to 532.67) | 899.21 (67.39 to 2312.22) | 2.68 (0.20 to 6.49) | 435.72 (219.66 to 684.54) | 851.10 (437.74 to 1309.64) | 2.61 (1.34 to 3.95) | -0.01 (-0.55 to 0.54) |
| Norway | 44706.47 (14556.55 to 79125.88) | 675.40 (229.44 to 1177.90) | 2.59 (0.91 to 4.56) | 8671.54 (3445.86 to 15037.99) | 89.00 (38.16 to 151.10) | 0.49 (0.21 to 0.84) | -6.95 (-7.21 to -6.69) |
| Oman | 52238.79 (36940.69 to 69432.72) | 4663.91 (3335.29 to 6100.75) | 11.47 (8.72 to 14.26) | 51435.86 (38948.31 to 65123.86) | 2383.54 (1798.88 to 3020.87) | 7.42 (5.81 to 8.96) | -1.64 (-1.81 to -1.46) |
| Pakistan | 9624371.43 (7249109.21 to 11919086.58) | 8052.58 (6606.84 to 9558.26) | 13.60 (11.09 to 15.81) | 11323036.39 (9263348.57 to 13555533.79) | 6278.18 (5311.62 to 7405.54) | 12.15 (10.43 to 13.88) | -0.86 (-1.04 to -0.69) |
| Palau | 106.42 (8.35 to 255.86) | 974.74 (64.13 to 2363.41) | 2.09 (0.14 to 5.11) | 185.36 (22.34 to 370.76) | 879.88 (140.16 to 1763.23) | 2.16 (0.36 to 4.17) | 0.20 (-0.64 to 1.04) |
| Palestine | 48981.22 (33505.65 to 65036.91) | 3457.49 (2516.14 to 4440.50) | 8.38 (6.34 to 10.54) | 57775.22 (43848.79 to 73578.14) | 2175.54 (1621.26 to 2747.36) | 6.86 (5.09 to 8.60) | -1.51 (-1.78 to -1.23) |
| Panama | 31632.54 (21215.96 to 41644.94) | 1751.87 (1135.32 to 2303.86) | 6.08 (3.96 to 8.01) | 22348.09 (13389.58 to 33870.64) | 521.37 (313.94 to 790.88) | 2.02 (1.26 to 2.96) | -3.95 (-4.16 to -3.74) |
| Papua New Guinea | 332272.31 (212812.06 to 442105.81) | 10482.18 (8055.44 to 13322.15) | 17.38 (13.75 to 20.57) | 576736.95 (443948.03 to 732303.46) | 7890.02 (6079.44 to 9805.55) | 14.52 (11.50 to 17.38) | -0.78 (-0.84 to -0.71) |
| Paraguay | 98086.81 (70447.98 to 128044.42) | 2934.27 (2089.63 to 3709.75) | 9.48 (6.66 to 11.76) | 62848.17 (27539.88 to 101938.83) | 1068.69 (467.87 to 1728.13) | 3.10 (1.38 to 4.90) | -3.25 (-3.59 to -2.90) |
| Peru | 966316.55 (585830.59 to 1304823.89) | 4569.19 (3047.50 to 5908.34) | 10.59 (6.99 to 13.70) | 398816.99 (271825.00 to 559672.05) | 1180.40 (808.86 to 1659.97) | 3.47 (2.40 to 4.64) | -4.46 (-4.63 to -4.30) |
| Philippines | 2891686.11 (2037920.79 to 3624009.96) | 5864.10 (4595.82 to 7031.84) | 13.50 (10.39 to 15.98) | 3077136.01 (2367176.99 to 3832242.95) | 3541.40 (2726.98 to 4450.99) | 8.62 (6.70 to 10.61) | -1.40 (-1.56 to -1.24) |
| Poland | 1533341.68 (1064084.69 to 1983297.09) | 3726.38 (2608.63 to 4769.77) | 10.42 (7.30 to 13.39) | 669165.93 (531475.52 to 841460.66) | 965.51 (771.43 to 1205.38) | 3.60 (2.88 to 4.52) | -4.42 (-4.59 to -4.25) |
| Portugal | 128060.80 (53609.85 to 212294.55) | 1021.15 (448.53 to 1650.12) | 3.27 (1.46 to 5.34) | 42954.83 (24225.19 to 65328.07) | 183.93 (104.27 to 278.40) | 0.89 (0.51 to 1.34) | -5.70 (-6.09 to -5.31) |
| Puerto Rico | 12911.54 (721.81 to 35995.43) | 373.45 (22.37 to 1025.67) | 1.20 (0.07 to 3.21) | 11704.36 (2979.88 to 21309.66) | 195.28 (59.02 to 347.43) | 0.84 (0.26 to 1.47) | -2.35 (-2.72 to -1.97) |
| Qatar | 8137.01 (6476.52 to 9875.30) | 5899.72 (4790.78 to 7043.87) | 15.45 (12.88 to 17.79) | 23966.29 (17859.73 to 30528.85) | 2272.76 (1714.69 to 2829.33) | 9.23 (7.55 to 10.89) | -3.42 (-4.00 to -2.83) |
| Republic of Korea | 491368.96 (237274.23 to 781723.22) | 1753.87 (831.66 to 2815.89) | 5.27 (2.53 to 8.49) | 603458.16 (440102.94 to 762879.84) | 681.66 (502.93 to 862.98) | 3.90 (2.83 to 5.06) | -3.28 (-3.52 to -3.03) |
| Republic of Moldova | 235403.30 (187082.24 to 279741.06) | 5826.68 (4652.50 to 6942.27) | 13.84 (10.92 to 16.57) | 82703.45 (54092.39 to 115196.73) | 1486.70 (981.94 to 2053.75) | 4.53 (3.03 to 6.21) | -5.32 (-5.77 to -4.88) |
| Romania | 936590.39 (564103.51 to 1327792.41) | 3929.86 (2425.03 to 5565.15) | 10.01 (6.15 to 14.12) | 370376.33 (275934.25 to 502183.85) | 1067.43 (788.20 to 1437.14) | 3.44 (2.54 to 4.65) | -4.83 (-5.19 to -4.47) |
| Russian Federation | 4793333.23 (2464511.06 to 7183743.72) | 2924.94 (1546.01 to 4334.22) | 7.38 (3.89 to 11.04) | 1918416.13 (1156545.85 to 2891891.72) | 839.00 (511.86 to 1255.56) | 2.24 (1.34 to 3.34) | -4.89 (-5.47 to -4.31) |
| Rwanda | 913627.61 (543639.99 to 1267854.23) | 12105.49 (8332.28 to 15730.21) | 12.25 (8.48 to 15.68) | 420011.95 (301421.34 to 530367.12) | 4610.00 (3397.23 to 5802.77) | 9.10 (7.02 to 10.86) | -4.02 (-4.37 to -3.68) |
| Saint Kitts and Nevis | 503.53 (171.94 to 889.38) | 1328.47 (426.53 to 2397.80) | 3.02 (1.00 to 5.51) | 278.38 (109.36 to 465.43) | 459.92 (187.21 to 757.13) | 1.34 (0.56 to 2.21) | -3.30 (-3.78 to -2.82) |
| Saint Lucia | 2963.45 (1907.75 to 4181.38) | 3055.63 (1873.84 to 4343.57) | 7.87 (4.83 to 11.00) | 2884.41 (1511.96 to 4314.14) | 1409.13 (774.78 to 2061.21) | 4.22 (2.43 to 6.23) | -2.62 (-2.95 to -2.29) |
| Saint Vincent and the Grenadines | 2378.49 (1571.86 to 3329.35) | 2969.09 (1908.60 to 4260.95) | 7.73 (4.94 to 10.70) | 2031.41 (1040.68 to 3102.29) | 1585.15 (841.22 to 2388.20) | 4.63 (2.45 to 6.81) | -2.04 (-2.26 to -1.82) |
| Samoa | 7330.40 (5547.98 to 9326.57) | 6803.61 (5439.34 to 8223.65) | 16.51 (13.51 to 19.47) | 7399.12 (5143.65 to 9853.28) | 4777.26 (3348.60 to 6364.99) | 13.31 (9.19 to 16.61) | -1.07 (-1.13 to -1.01) |
| San Marino | 188.47 (76.21 to 318.84) | 570.43 (248.11 to 941.31) | 2.56 (1.16 to 4.36) | 114.92 (59.86 to 189.91) | 157.55 (83.49 to 260.24) | 0.92 (0.49 to 1.42) | -3.88 (-4.31 to -3.43) |
| Sao Tome and Principe | 8445.06 (5387.00 to 11326.42) | 7018.73 (4938.87 to 8981.31) | 12.50 (8.74 to 15.73) | 4520.74 (3385.65 to 5695.76) | 3583.27 (2775.98 to 4415.41) | 9.76 (7.53 to 11.92) | -2.24 (-2.48 to -2.01) |
| Saudi Arabia | 411129.09 (291980.20 to 544177.39) | 4309.48 (3176.90 to 5559.98) | 9.72 (7.80 to 11.80) | 764225.24 (563388.72 to 982447.75) | 3177.46 (2460.09 to 3958.21) | 9.95 (8.15 to 11.98) | -0.85 (-1.04 to -0.66) |
| Senegal | 689771.64 (475136.97 to 887703.54) | 7923.49 (6093.58 to 9645.29) | 11.24 (8.72 to 13.62) | 619414.06 (473990.51 to 769415.49) | 5280.08 (4146.31 to 6435.17) | 10.87 (8.88 to 12.65) | -1.23 (-1.32 to -1.14) |
| Serbia | 472966.63 (316103.76 to 612847.84) | 5087.71 (3503.62 to 6568.19) | 13.31 (9.21 to 17.34) | 293094.00 (222269.61 to 389439.17) | 1827.93 (1398.64 to 2416.05) | 6.37 (4.92 to 8.54) | -3.96 (-4.34 to -3.57) |
| Seychelles | 479.93 (199.78 to 821.42) | 795.88 (317.65 to 1364.78) | 2.23 (0.89 to 3.81) | 643.10 (255.49 to 1122.56) | 589.20 (246.60 to 1011.58) | 1.92 (0.82 to 3.17) | -1.30 (-1.56 to -1.04) |
| Sierra Leone | 669906.87 (408668.10 to 933846.35) | 11202.12 (7472.92 to 14873.24) | 11.70 (7.83 to 15.30) | 485243.55 (346171.66 to 641768.46) | 6610.13 (4976.80 to 8442.11) | 10.47 (8.30 to 12.92) | -1.66 (-1.76 to -1.57) |
| Singapore | 53057.68 (24832.03 to 81527.15) | 2417.38 (1124.66 to 3728.21) | 9.32 (4.08 to 14.84) | 39020.36 (21666.03 to 57027.84) | 469.73 (259.71 to 686.85) | 3.15 (1.82 to 4.70) | -4.90 (-5.32 to -4.47) |
| Slovakia | 169936.74 (97755.60 to 244129.56) | 2988.00 (1745.06 to 4282.48) | 8.48 (4.86 to 12.30) | 83690.25 (63611.00 to 104610.70) | 923.84 (709.70 to 1150.76) | 3.30 (2.54 to 4.10) | -3.61 (-3.85 to -3.37) |
| Slovenia | 38555.34 (22142.71 to 56547.23) | 1652.05 (961.74 to 2381.40) | 5.54 (3.25 to 8.05) | 16546.89 (12289.57 to 21570.00) | 380.75 (283.32 to 496.73) | 1.88 (1.41 to 2.46) | -4.92 (-5.11 to -4.73) |
| Solomon Islands | 24003.59 (15934.47 to 32918.55) | 11105.77 (8038.23 to 14428.10) | 18.70 (14.32 to 22.77) | 36607.22 (27091.97 to 47200.18) | 8813.39 (6860.11 to 11307.05) | 18.85 (15.80 to 21.77) | -0.71 (-0.80 to -0.62) |
| Somalia | 949052.97 (574218.21 to 1365736.17) | 10546.16 (7282.26 to 14171.53) | 10.01 (6.78 to 12.94) | 1384132.16 (886764.89 to 1874378.48) | 7081.22 (5114.04 to 9224.97) | 7.85 (5.75 to 9.71) | -1.20 (-1.28 to -1.11) |
| South Africa | 1335057.98 (890627.08 to 1765903.07) | 3897.51 (2742.17 to 5046.39) | 7.11 (5.01 to 9.17) | 1124060.48 (848996.29 to 1447167.10) | 2396.63 (1828.97 to 3078.39) | 3.81 (2.89 to 4.84) | -1.53 (-1.91 to -1.15) |
| South Sudan | 595661.96 (378495.97 to 847692.11) | 8583.53 (6081.85 to 11536.42) | 9.28 (6.41 to 11.94) | 735191.47 (489019.14 to 1019065.69) | 7361.69 (5193.46 to 9853.66) | 8.94 (6.78 to 11.03) | -0.47 (-0.74 to -0.19) |
| Spain | 448131.86 (203892.76 to 726297.33) | 889.61 (426.97 to 1407.78) | 3.44 (1.65 to 5.38) | 200535.98 (131262.03 to 279629.06) | 213.19 (138.76 to 295.55) | 1.14 (0.75 to 1.57) | -4.56 (-4.86 to -4.26) |
| Sri Lanka | 578452.21 (497977.32 to 661605.58) | 5158.56 (4451.35 to 5860.40) | 13.19 (11.32 to 14.95) | 492066.48 (298628.34 to 759708.15) | 1946.47 (1190.80 to 2980.02) | 7.43 (4.81 to 11.01) | -3.04 (-3.46 to -2.63) |
| Sudan | 2115615.86 (1391803.08 to 2820142.56) | 10326.81 (7905.40 to 12722.49) | 14.27 (11.03 to 17.25) | 1250304.53 (900191.79 to 1665433.69) | 4748.13 (3558.76 to 6279.46) | 10.83 (8.48 to 13.03) | -2.55 (-2.61 to -2.48) |
| Suriname | 11366.47 (7312.35 to 15701.07) | 3650.98 (2278.61 to 5163.30) | 8.52 (5.34 to 12.05) | 12617.05 (7504.66 to 18560.23) | 2130.22 (1290.31 to 3064.41) | 5.56 (3.44 to 7.77) | -1.98 (-2.27 to -1.68) |
| Sweden | 83214.48 (23271.51 to 154302.88) | 558.50 (171.31 to 1022.62) | 2.34 (0.74 to 4.31) | 16655.64 (5051.69 to 30736.49) | 78.40 (25.04 to 141.27) | 0.44 (0.15 to 0.78) | -6.60 (-6.95 to -6.25) |
| Switzerland | 99719.29 (50184.47 to 156153.37) | 980.73 (503.90 to 1513.24) | 3.85 (1.97 to 5.88) | 31735.28 (20700.74 to 44264.37) | 182.67 (119.16 to 257.09) | 1.02 (0.68 to 1.41) | -5.41 (-5.60 to -5.22) |
| Syrian Arab Republic | 316111.53 (218908.49 to 425496.44) | 3740.36 (2727.47 to 4935.04) | 9.00 (6.71 to 11.23) | 331226.61 (232186.82 to 455360.54) | 2690.00 (1916.70 to 3628.27) | 7.74 (5.88 to 10.26) | -1.01 (-1.29 to -0.73) |
| Taiwan (Province of China) | 241952.69 (116298.00 to 402651.89) | 1612.09 (769.53 to 2702.16) | 5.86 (2.80 to 9.79) | 241685.18 (190897.17 to 301354.81) | 607.83 (480.33 to 767.62) | 3.12 (2.44 to 3.99) | -2.78 (-3.03 to -2.53) |
| Tajikistan | 424615.05 (256071.82 to 595647.00) | 8033.66 (5814.58 to 10238.02) | 16.74 (12.16 to 21.38) | 318106.02 (236010.63 to 418360.34) | 4289.39 (3442.82 to 5352.37) | 10.98 (8.83 to 13.38) | -2.41 (-2.68 to -2.14) |
| Thailand | 1527847.27 (1208353.67 to 1853327.54) | 4007.33 (3166.89 to 4800.06) | 10.89 (8.60 to 12.83) | 1397292.31 (1049019.05 to 1821193.35) | 1408.80 (1065.25 to 1826.80) | 5.15 (4.00 to 6.45) | -3.85 (-4.11 to -3.59) |
| Timor-Leste | 84872.47 (46347.13 to 120207.56) | 9484.96 (6540.62 to 12272.92) | 13.98 (9.51 to 17.48) | 51819.82 (36566.52 to 67861.06) | 4870.76 (3369.91 to 6427.76) | 11.78 (8.39 to 14.89) | -2.40 (-2.66 to -2.13) |
| Togo | 338018.76 (224327.88 to 454540.79) | 8588.79 (6155.14 to 10862.37) | 11.90 (8.64 to 14.59) | 364802.82 (255933.63 to 481244.15) | 6149.19 (4500.03 to 7964.26) | 11.03 (8.65 to 13.35) | -1.04 (-1.18 to -0.90) |
| Tokelau | 10.35 (0.83 to 31.92) | 744.25 (55.21 to 2328.79) | 1.77 (0.13 to 5.51) | 8.64 (0.81 to 20.29) | 672.00 (88.75 to 1499.07) | 1.48 (0.19 to 3.33) | -1.72 (-2.28 to -1.16) |
| Tonga | 3140.10 (2327.38 to 4036.75) | 4570.91 (3449.94 to 5642.57) | 12.80 (10.07 to 15.56) | 2327.83 (1431.59 to 3257.36) | 2725.75 (1677.18 to 3813.90) | 8.73 (5.46 to 11.76) | -1.55 (-1.66 to -1.44) |
| Trinidad and Tobago | 23758.22 (9084.69 to 41130.38) | 2736.73 (928.72 to 4788.48) | 6.89 (2.29 to 12.08) | 30778.41 (14232.05 to 49145.75) | 1719.63 (825.78 to 2736.94) | 4.69 (2.25 to 7.21) | -1.82 (-2.08 to -1.56) |
| Tunisia | 175251.20 (127617.14 to 230091.94) | 2708.02 (2013.53 to 3445.71) | 7.46 (5.60 to 9.58) | 191130.20 (130835.80 to 265007.96) | 1531.52 (1061.88 to 2109.50) | 4.83 (3.42 to 6.49) | -1.84 (-2.02 to -1.66) |
| Türkiye | 1656328.55 (1220024.41 to 2209386.80) | 3607.88 (2725.49 to 4646.42) | 7.96 (6.04 to 10.21) | 1316361.98 (1027408.52 to 1634604.27) | 1495.28 (1172.94 to 1857.97) | 5.25 (4.25 to 6.38) | -2.71 (-3.04 to -2.37) |
| Turkmenistan | 111160.03 (39793.08 to 211646.22) | 3379.92 (1186.24 to 6155.55) | 7.06 (2.48 to 12.76) | 106184.52 (65858.61 to 156774.64) | 2550.78 (1586.38 to 3759.53) | 6.06 (3.83 to 8.87) | -1.23 (-1.53 to -0.93) |
| Tuvalu | 769.64 (494.22 to 1068.90) | 8541.33 (6136.33 to 11002.25) | 13.45 (10.06 to 16.64) | 168.89 (99.96 to 261.82) | 1604.44 (943.66 to 2457.82) | 3.90 (2.32 to 6.02) | -5.69 (-5.84 to -5.55) |
| Uganda | 1497136.89 (988580.04 to 2007600.56) | 7131.32 (5165.73 to 9015.88) | 6.86 (5.01 to 8.75) | 1516904.60 (1086069.29 to 1990273.19) | 4556.24 (3499.32 to 5660.22) | 7.44 (5.92 to 9.17) | -1.77 (-1.99 to -1.55) |
| Ukraine | 2319356.32 (1305617.28 to 3327888.40) | 3526.91 (2030.96 to 5008.93) | 9.43 (5.46 to 13.59) | 1161823.35 (677950.94 to 1778250.05) | 1559.19 (918.56 to 2368.61) | 4.31 (2.60 to 6.36) | -3.50 (-4.03 to -2.98) |
| United Arab Emirates | 29359.86 (22417.19 to 37936.83) | 4233.30 (3210.32 to 5435.38) | 11.24 (9.05 to 13.58) | 80008.23 (60568.88 to 99799.86) | 2255.67 (1706.89 to 2822.61) | 8.16 (6.54 to 10.14) | -0.67 (-1.19 to -0.15) |
| United Kingdom | 1169978.84 (573625.97 to 1849869.20) | 1349.67 (684.31 to 2115.21) | 4.92 (2.42 to 7.69) | 332998.35 (220754.46 to 451150.47) | 278.50 (190.01 to 373.51) | 1.26 (0.87 to 1.72) | -5.70 (-5.94 to -5.45) |
| United Republic of Tanzania | 2654017.66 (1381339.66 to 3671322.22) | 8059.83 (5019.85 to 10400.55) | 9.87 (6.06 to 12.87) | 2212436.51 (1611753.37 to 2890713.42) | 4772.29 (3746.66 to 5844.29) | 8.73 (7.08 to 10.43) | -1.73 (-1.79 to -1.67) |
| United States of America | 2903557.35 (1293674.17 to 4773752.45) | 947.75 (434.04 to 1537.86) | 3.26 (1.45 to 5.36) | 1201982.15 (608699.27 to 1905698.37) | 224.40 (118.09 to 346.13) | 0.78 (0.42 to 1.22) | -5.06 (-5.45 to -4.66) |
| United States Virgin Islands | 502.16 (80.76 to 1186.51) | 578.26 (83.92 to 1406.08) | 1.72 (0.24 to 4.15) | 505.89 (237.38 to 804.33) | 319.71 (154.71 to 500.18) | 1.13 (0.57 to 1.78) | -1.82 (-2.16 to -1.47) |
| Uruguay | 45662.44 (20146.51 to 73435.80) | 1253.71 (582.21 to 1981.57) | 3.90 (1.77 to 6.15) | 21579.25 (8739.22 to 36884.59) | 415.64 (179.09 to 704.06) | 1.49 (0.63 to 2.53) | -3.84 (-4.09 to -3.58) |
| Uzbekistan | 970240.91 (568147.42 to 1418756.33) | 5242.66 (3501.82 to 7100.51) | 12.94 (8.55 to 17.51) | 948807.13 (724290.03 to 1221185.03) | 3536.77 (2674.31 to 4527.64) | 10.77 (8.35 to 13.33) | -1.31 (-1.63 to -0.99) |
| Vanuatu | 9475.88 (7203.12 to 12091.72) | 10039.77 (8070.67 to 12402.18) | 19.06 (16.10 to 21.88) | 16332.49 (13010.01 to 19900.30) | 8039.26 (6553.84 to 9589.65) | 17.03 (14.20 to 19.69) | -0.83 (-0.90 to -0.76) |
| Venezuela (Bolivarian Republic of) | 271296.42 (174207.66 to 386878.41) | 1975.96 (1202.12 to 2896.60) | 5.64 (3.43 to 8.21) | 320709.62 (198987.41 to 477427.63) | 1123.08 (701.22 to 1674.51) | 2.88 (1.88 to 4.16) | -1.85 (-2.00 to -1.71) |
| Viet Nam | 3413682.65 (2628500.06 to 4170741.65) | 6225.83 (4984.92 to 7504.53) | 15.74 (13.01 to 18.19) | 2435418.99 (1791944.54 to 3131543.63) | 2618.71 (1944.21 to 3343.78) | 9.17 (6.95 to 11.31) | -2.83 (-2.98 to -2.69) |
| Yemen | 1303503.87 (895635.36 to 1799976.73) | 9350.53 (7227.64 to 11862.79) | 13.26 (10.44 to 16.04) | 1002611.44 (774647.85 to 1315689.47) | 4849.65 (3680.37 to 6432.23) | 9.41 (7.40 to 11.61) | -2.26 (-2.34 to -2.17) |
| Zambia | 718106.63 (394758.32 to 1031646.40) | 7814.88 (5154.65 to 10087.70) | 8.39 (5.42 to 10.94) | 630917.68 (437603.45 to 844736.51) | 5092.56 (3738.45 to 6522.51) | 6.98 (5.51 to 8.54) | -1.47 (-1.68 to -1.26) |
| Zimbabwe | 456264.31 (297638.56 to 601488.02) | 5091.93 (3578.78 to 6395.00) | 8.54 (6.01 to 10.77) | 690980.03 (467368.65 to 946020.97) | 6127.39 (4472.58 to 8033.07) | 7.10 (5.35 to 8.76) | 1.41 (0.92 to 1.90) |

Figure S1. Numbers of all-age deaths and DALYs attributable to particulate matter pollution by sex, 1990–2021.

(A) Deaths. (B) DALYs. DALYs, disability-adjusted life years.

Figure S2. Numbers of all-age deaths and DALYs attributable to ambient particulate matter pollution by sex, 1990–2021.

(A) Deaths. (B) DALYs. DALY, disability-adjusted life years.

Figure S3. Numbers of all-age deaths and DALYs attributable to household particulate matter pollution by sex, 1990–2021.

(A) Deaths. (B) DALYs. DALYs, disability-adjusted life years.

Figure S4. Age-standardised death and DALY rates attributable to particulate matter pollution by sex, 1990–2021.

(A) Deaths. (B) DALYs. DALYs, disability-adjusted life years.

Figure S5. Age-standardised death and DALY rates attributable to ambient particulate matter pollution by sex, 1990–2021.

(A) Deaths. (B) DALYs. DALYs, disability-adjusted life years.

Figure S6. Age-standardised death and DALY rates attributable to household particulate matter pollution by sex, 1990–2021.

(A) Deaths. (B) DALYs. DALYs, disability-adjusted life years.

Figure S7. Age-specific numbers and rates of deaths and DALYs attributable to ambient particulate matter pollution by sex, in 2021.

(A) Deaths attributable to ambient particulate matter pollution. (B) DALYs attributable to ambient particulate matter pollution. DALYs, disability-adjusted life years.

Figure S8. Age-specific numbers and rates of deaths and DALYs attributable to household particulate matter pollution by sex, in 2021.

(A) Deaths attributable to household particulate matter pollution. (B) DALYs attributable to household particulate matter pollution. DALYs, disability-adjusted life years.

Figure S9. Age-standardised death rates and their EAPCs attributable to ambient particulate matter pollution by country.

(A) Age-standardised death rates in 2021. (B) EAPCs in age-standardised death rates, 1990–2021. EAPCs, estimated annual percentage changes.

Figure S10. Age-standardised death rates and their EAPCs attributable to household particulate matter pollution by country.

(A) Age-standardised death rates, in 2021. (B) EAPCs in age-standardised death rates, 1990–2021. EAPCs, estimated annual percentage changes.

Figure S11. Age-standardised DALY rates and their EAPCs attributable to household particulate matter pollution by country.

(A) Age-standardised DALY rates, in 2021. (B) EAPCs in age-standardised DALY rates, 1990–2021. DALYs, disability-adjusted life years. EAPCs, estimated annual percentage changes.

Figure S12. Age-standardised death rates and their EAPCs attributable to particulate matter pollution by country.

(A) Age-standardised death rates, in 2021. (B) EAPCs in age-standardised death rates, 1990–2021. EAPCs, estimated annual percentage changes.

Figure S13. Age-standardised DALY rates and their EAPCs attributable to particulate matter pollution by country.

(A) Age-standardised DALY rates, in 2021. (B) EAPCs in age-standardised DALY rates, 1990–2021. DALYs, disability-adjusted life years. EAPCs, estimated annual percentage changes.

Figure S14. Estimated annual percentage changes in age-standardised death rate attributable to ambient particulate matter pollution, 1990–2021, for the leading ten level 3 attributable causes in 2021, by SDI quintile and GBD region.

For each region and SDI quintile, level 3 causes are ranked by attributable death counts in 2021 from left (first) to right (tenth). Causes are coloured by their estimated annual percentage changes in age-standardised death rates attributable to ambient particulate matter pollution, from 1990 to 2021. SDI, socio-demographic index. GBD, Global Burden of Diseases, Injuries, and Risk Factors Study.

Figure S15. Estimated annual percentage changes in age-standardised death rate attributable to household particulate matter pollution, 1990–2021, for the leading ten level 3 attributable causes in 2021, by SDI quintile and GBD region.

For each region and SDI quintile, level 3 causes are ranked by attributable death counts in 2021 from left (first) to right (tenth). Causes are coloured by their estimated annual percentage changes in age-standardised death rates attributable to househould particulate matter pollution, from 1990 to 2021. SDI, socio-demographic index. GBD, Global Burden of Diseases, Injuries, and Risk Factors Study.

Figure S16. Estimated annual percentage changes in age-standardised DALY rate attributable to household particulate matter pollution, 1990–2021, for the leading ten level 3 attributable causes in 2021, by SDI quintile and GBD region.

For each region and SDI quintile, level 3 causes are ranked by attributable DALY counts in 2021 from left (first) to right (tenth). Causes are coloured by their estimated annual percentage changes in age-standardised DALY rates attributable to household particulate matter pollution, from 1990 to 2021. DALYs, disability-adjusted life years. SDI, socio-demographic index. GBD, Global Burden of Diseases, Injuries, and Risk Factors Study.

Figure S17. Estimated annual percentage changes in age-standardised death rate attributable to particulate matter pollution, 1990–2021, for the leading ten level 3 attributable causes in 2021, by SDI quintile and GBD region.

For each region and SDI quintile, level 3 causes are ranked by attributable death counts in 2021 from left (first) to right (tenth). Causes are coloured by their estimated annual percentage changes in age-standardised death rates attributable to particulate matter pollution, from 1990 to 2021. SDI, socio-demographic index. GBD, Global Burden of Diseases, Injuries, and Risk Factors Study.

Figure S18. Estimated annual percentage changes in age-standardised DALY rate attributable to particulate matter pollution, 1990–2021, for the leading ten level 3 attributable causes in 2021, by SDI quintile and GBD region.

For each region and SDI quintile, level 3 causes are ranked by attributable DALY counts in 2021 from left (first) to right (tenth). Causes are coloured by their estimated annual percentage changes in age-standardised DALY rates attributable to particulate matter pollution, from 1990 to 2021. DALYs, disability-adjusted life years. SDI, socio-demographic index. GBD, Global Burden of Diseases, Injuries, and Risk Factors Study.


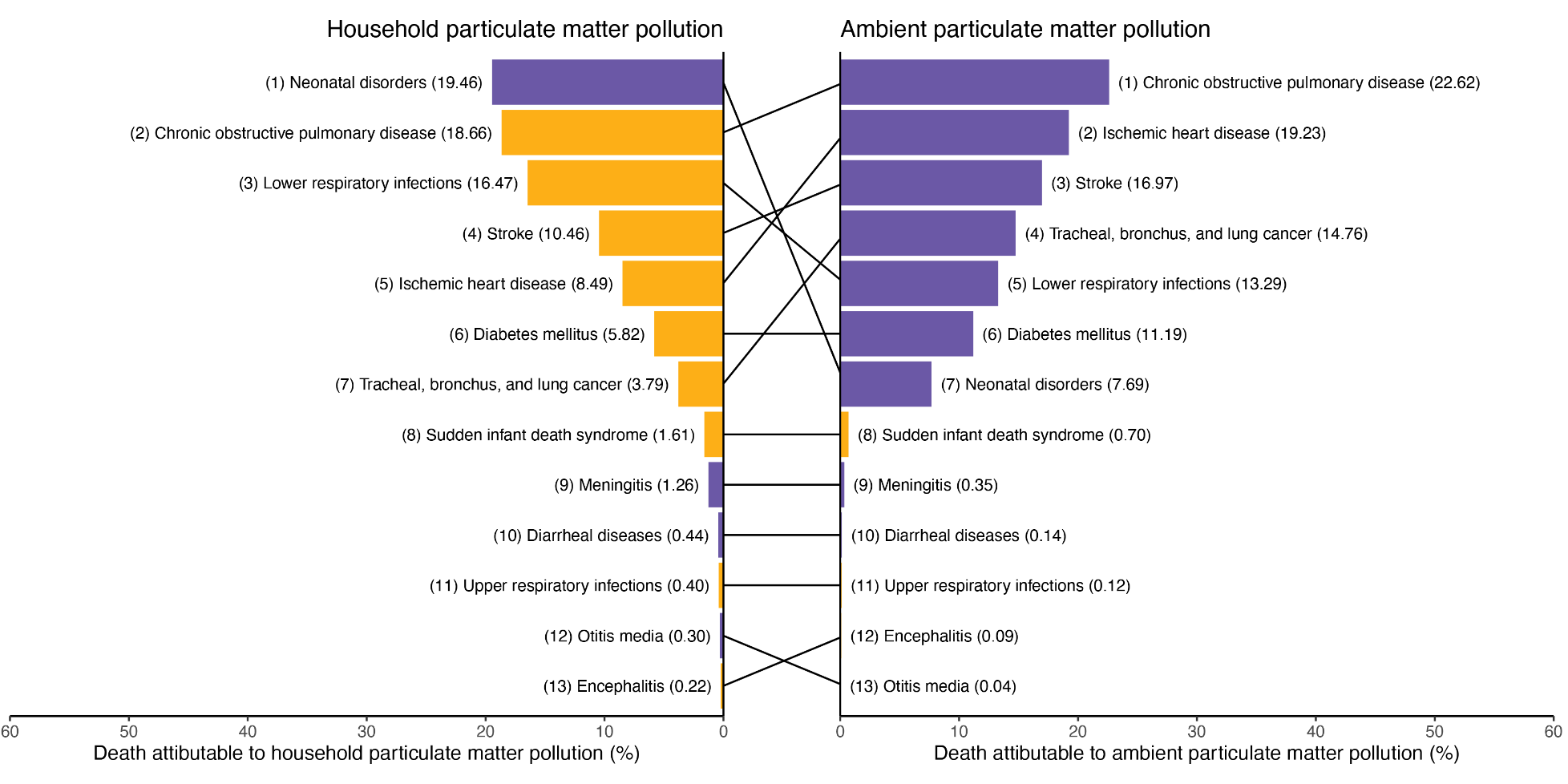


Figure S19. Global rankings of fractions of level 3 causes in age-standardised deaths attributable to ambient and household particulate matter pollution globally in 2021

The list of causes of disease burden represents the level 3 causes of age-standardised deaths (per 100 000 population) attributable to APMP and HPMP observed for both sexes globally in 2021. This same list of health conditions was ranked according to their PAFs for both APMP and HPMP globally in 2021. The colours of the bars and lines denote whether the PAFs are higher for females (yellow) or males (purple) as established by whether the 95% uncertainty interval of the absolute difference in PAFs includes zero. APMP, ambient particulate matter pollution. HPMP, household particulate matter pollution. PAFs, population attributable fractions.

Figure S20. Fraction of lower respiratory infections, chronic obstructive pulmonary disease, and diabetes mellitus in age-standardised DALYs attributable to particulate matter pollution by region and by age group for females and males in 2021.

(A) By region. (B) By age group. The fourth to sixth leading GBD level 3 causes of particulate matter pollution-attributable DALYs are shown. DALYs, disability-adjusted life years. GBD, Global Burden of Diseases, Injuries, and Risk Factors Study.

Figure S21. Fraction of tracheal, bronchus, and lung cancer, blindness and vision loss, and diarrheal diseases in age-standardised DALYs attributable to particulate matter pollution by region and by age group for females and males in 2021.

(A) By region. (B) By age group. DALYs, disability-adjusted life years. GBD, Global Burden of Diseases, Injuries, and Risk Factors Study.

Figure S22. Fraction of meningitis, sudden infant death syndrome, and encephalitis in age-standardised DALYs attributable to particulate matter pollution by region and by age group for females and males in 2021.

(A) By region. (B) By age group. DALYs, disability-adjusted life years. GBD, Global Burden of Diseases, Injuries, and Risk Factors Study.

Figure S23. Fraction of upper respiratory infections and otitis media in age-standardised DALYs attributable to particulate matter pollution by region and by age group for females and males in 2021.

(A) By region. (B) By age group. DALYs, disability-adjusted life years. GBD, Global Burden of Diseases, Injuries, and Risk Factors Study.

Figure S24. Fraction of ischemic heart disease, stroke, and chronic obstructive pulmonary disease in age-standardised DALYs attributable to ambient particulate matter pollution by region and by age group for females and males in 2021.

(A) By region. (B) By age group. The three leading GBD level 3 causes of ambient particulate matter pollution-attributable DALYs are shown. DALYs, disability-adjusted life years. GBD, Global Burden of Diseases, Injuries, and Risk Factors Study.

Figure S25. Fraction of neonatal disorders, lower respiratory infections, and diabetes mellitus in age-standardised DALYs attributable to ambient particulate matter pollution by region and by age group for females and males in 2021.

(A) By region. (B) By age group. The fourth to sixth leading GBD level 3 causes of ambient particulate matter pollution-attributable DALYs are shown. DALYs, disability-adjusted life years. GBD, Global Burden of Diseases, Injuries, and Risk Factors Study.

Figure S26. Fraction of tracheal, bronchus, and lung cancer, diarrheal diseases, and meningitis in age-standardised DALYs attributable to ambient particulate matter pollution by region and by age group for females and males in 2021.

(A) By region. (B) By age group. DALYs, disability-adjusted life years. GBD, Global Burden of Diseases, Injuries, and Risk Factors Study.

Figure S27. Fraction of sudden infant death syndrome, encephalitis, and upper respiratory infections in age-standardised DALYs attributable to ambient particulate matter pollution by region and by age group for females and males in 2021.

(A) By region. (B) By age group. DALYs, disability-adjusted life years. GBD, Global Burden of Diseases, Injuries, and Risk Factors Study.

Figure S28. Fraction of Otitis media in age-standardised DALYs attributable to ambient particulate matter pollution by region and by age group for females and males in 2021.

(A) By region. (B) By age group. DALYs, disability-adjusted life years. GBD, Global Burden of Diseases, Injuries, and Risk Factors Study.

Figure S29. Fraction of neonatal disorders, lower respiratory infections, and ischemic heart disease in age-standardised DALYs attributable to household particulate matter pollution by region and by age group for females and males in 2021.

(A) By region. (B) By age group. The three leading GBD level 3 causes of household particulate matter pollution-attributable DALYs are shown. DALYs, disability-adjusted life years. GBD, Global Burden of Diseases, Injuries, and Risk Factors Study.

Figure S30. Fraction of stroke, chronic obstructive pulmonary disease, and diabetes mellitus in age-standardised DALYs attributable to household particulate matter pollution by region and by age group for females and males in 2021.

(A) By region. (B) By age group. The fourth to sixth leading GBD level 3 causes of household particulate matter pollution-attributable DALYs are shown. DALYs, disability-adjusted life years. GBD, Global Burden of Diseases, Injuries, and Risk Factors Study.

Figure S31. Fraction of blindness and vision loss, tracheal, bronchus, and lung cancer, and diarrheal diseases in age-standardised DALYs attributable to household particulate matter pollution by region and by age group for females and males in 2021.

(A) By region. (B) By age group. DALYs, disability-adjusted life years. GBD, Global Burden of Diseases, Injuries, and Risk Factors Study.

Figure S32. Fraction of meningitis, sudden infant death syndrome, and encephalitis in age-standardised DALYs attributable to household particulate matter pollution by region and by age group for females and males in 2021.

(A) By region. (B) By age group. DALYs, disability-adjusted life years. GBD, Global Burden of Diseases, Injuries, and Risk Factors Study.

Figure S33. Fraction of upper respiratory infections and otitis media in age-standardised DALYs attributable to household particulate matter pollution by region and by age group for females and males in 2021.

(A) By region. (B) By age group. DALYs, disability-adjusted life years. GBD, Global Burden of Diseases, Injuries, and Risk Factors Study.

Fig S34. Age-standardised death and DALY rates attributable to particulate matter pollution across 204 countries and territories by socio-demographic index in 2021.

(A) Age-standardised death rate attributable to ambient particulate matter pollution. (B) Age-standardised DALY rate attributable to ambient particulate matter pollution. DALYs, disability-adjusted life years. SDI, socio-demographic index.

Fig S35. Age-standardised death and DALY rates attributable to household particulate matter pollution across 204 countries and territories by socio-demographic index in 2021.

(A) Age-standardised death rate attributable to ambient particulate matter pollution. (B) Age-standardised DALY rate attributable to ambient particulate matter pollution. DALYs, disability-adjusted life years. SDI, socio-demographic index.

Fig S36. Age-standardised death and DALY rates attributable to ambient particulate matter pollution across 204 countries and territories by socio-demographic index in 2021.

(A) Age-standardised death rate attributable to ambient particulate matter pollution. (B) Age-standardised DALY rate attributable to ambient particulate matter pollution. DALYs, disability-adjusted life years. SDI, socio-demographic index.

Fig S37. Estimated annual percentage changes in age-standardised death and DALY rates attributable to particulate matter pollution across 204 countries and territories by socio-demographic index in 2021.

(A) EAPC in age-standardised death rate attributable to ambient particulate matter pollution. (B) EAPC in age-standardised DALY rate attributable to ambient particulate matter pollution. EAPCs, estimated annual percentage changes. DALYs, disability-adjusted life years. SDI, socio-demographic index.

Fig S38. Estimated annual percentage changes in age-standardised death and DALY rates attributable to household particulate matter pollution across 204 countries and territories by socio-demographic index in 2021.

(A) EAPC in age-standardised death rate attributable to ambient particulate matter pollution. (B) EAPC in age-standardised DALY rate attributable to ambient particulate matter pollution. EAPCs, estimated annual percentage changes. DALYs, disability-adjusted life years. SDI, socio-demographic index.

Fig S39. Estimated annual percentage changes in age-standardised death and DALY rates attributable to ambient particulate matter pollution across 204 countries and territories by socio-demographic index in 2021.

(A) EAPC in age-standardised death rate attributable to ambient particulate matter pollution. (B) EAPC in age-standardised DALY rate attributable to ambient particulate matter pollution. EAPCs, estimated annual percentage changes. DALYs, disability-adjusted life years. SDI, socio-demographic index.
