# Supplementary figures and images for: Global, Regional, and National Burden Attributed to Particulate Matter Pollution, 1990–2021: A Systematic Analysis for the Global Burden of Disease Study 2021
Source: Ann Glob Health. 2026 Feb 24;92(1):22. doi: 10.5334/aogh.4965 (PMC12947824; doi:10.5334/aogh.4965)

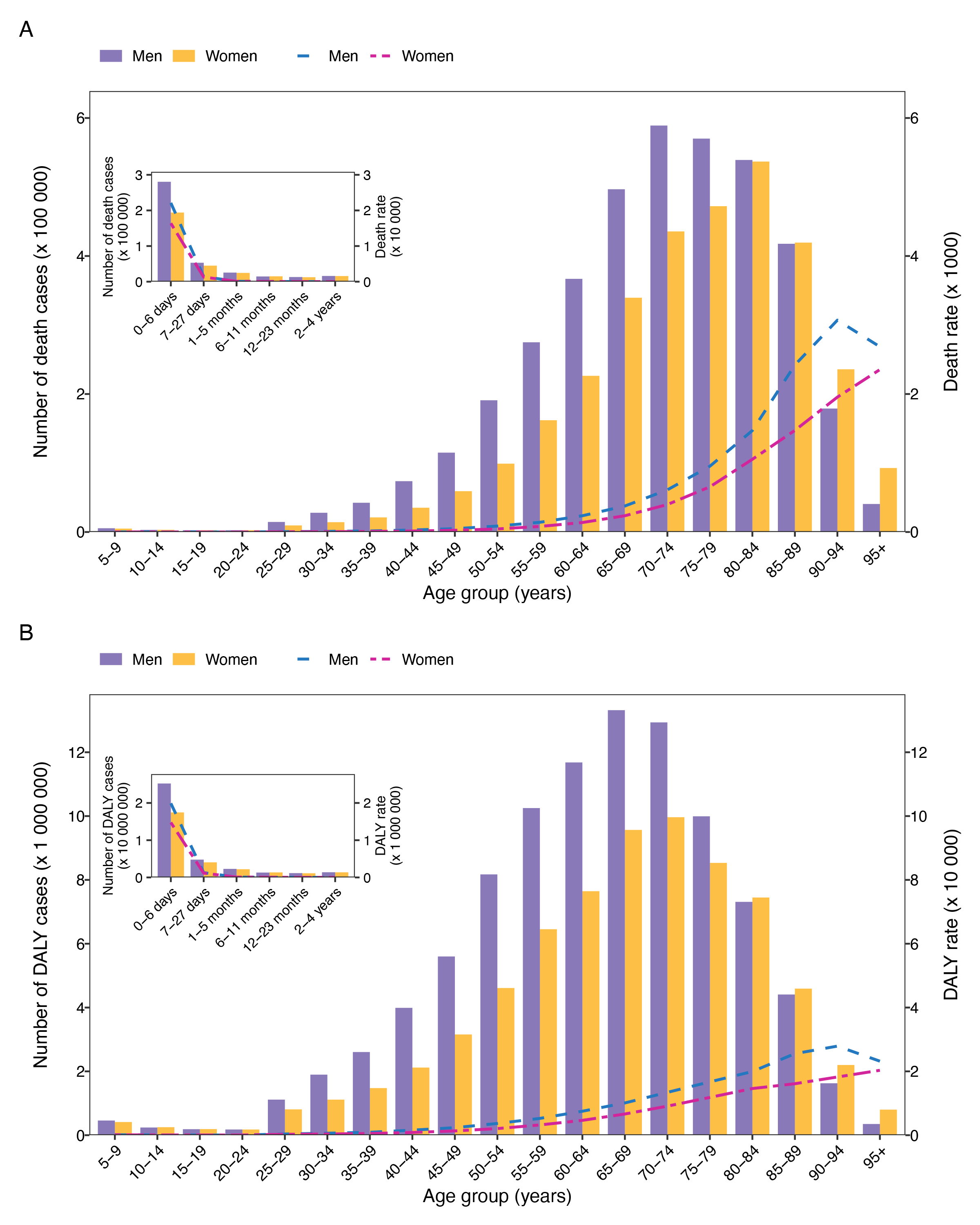

Supplement: Supplementary Materials. — Supplementary appendix. [file agh-92-1-4965-s1.zip › agh-92-1-4965-s1/Figure_1.TIFF]

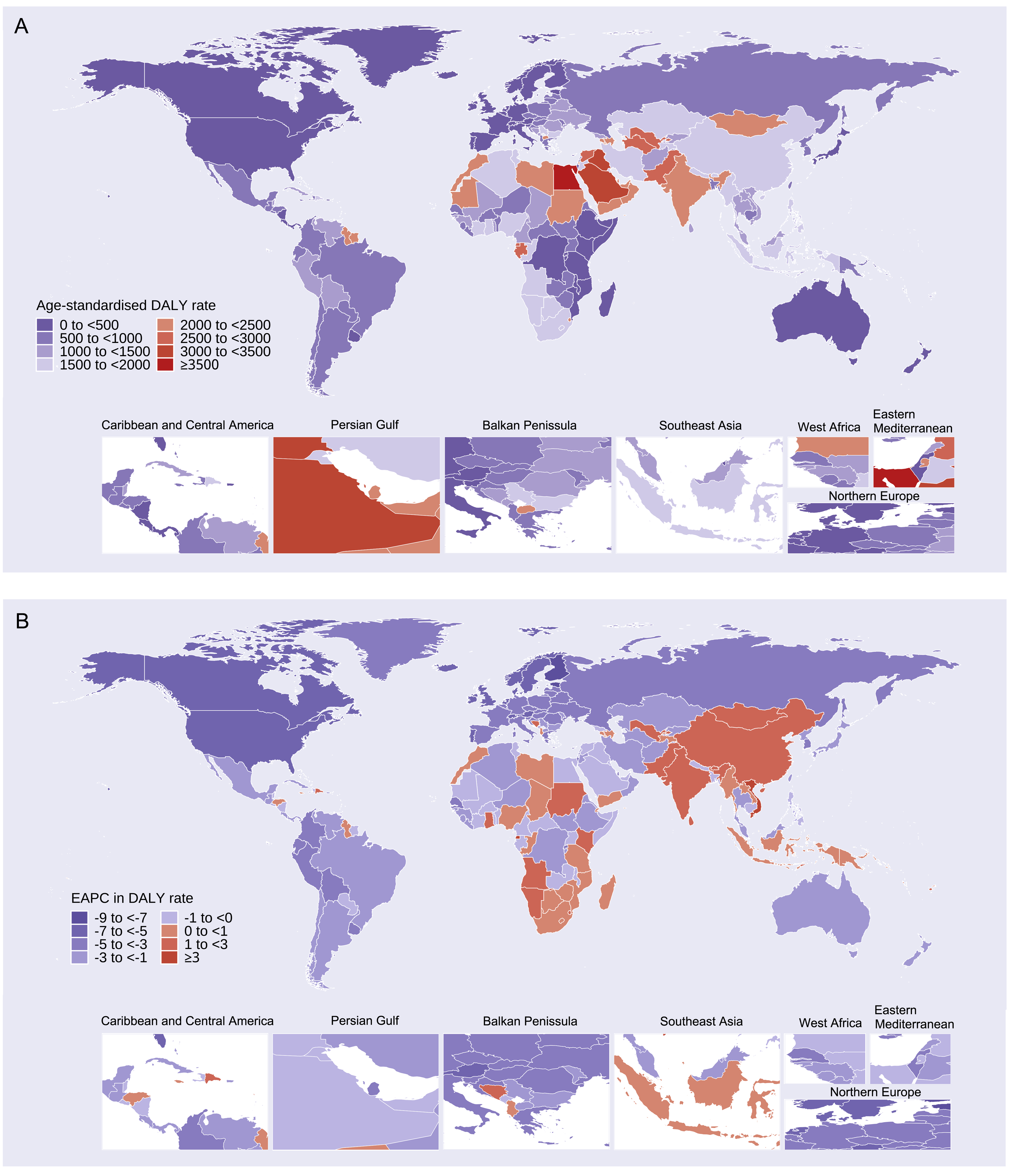

Supplement: Supplementary Materials. — Supplementary appendix. [file agh-92-1-4965-s1.zip › agh-92-1-4965-s1/Figure_2.TIFF]

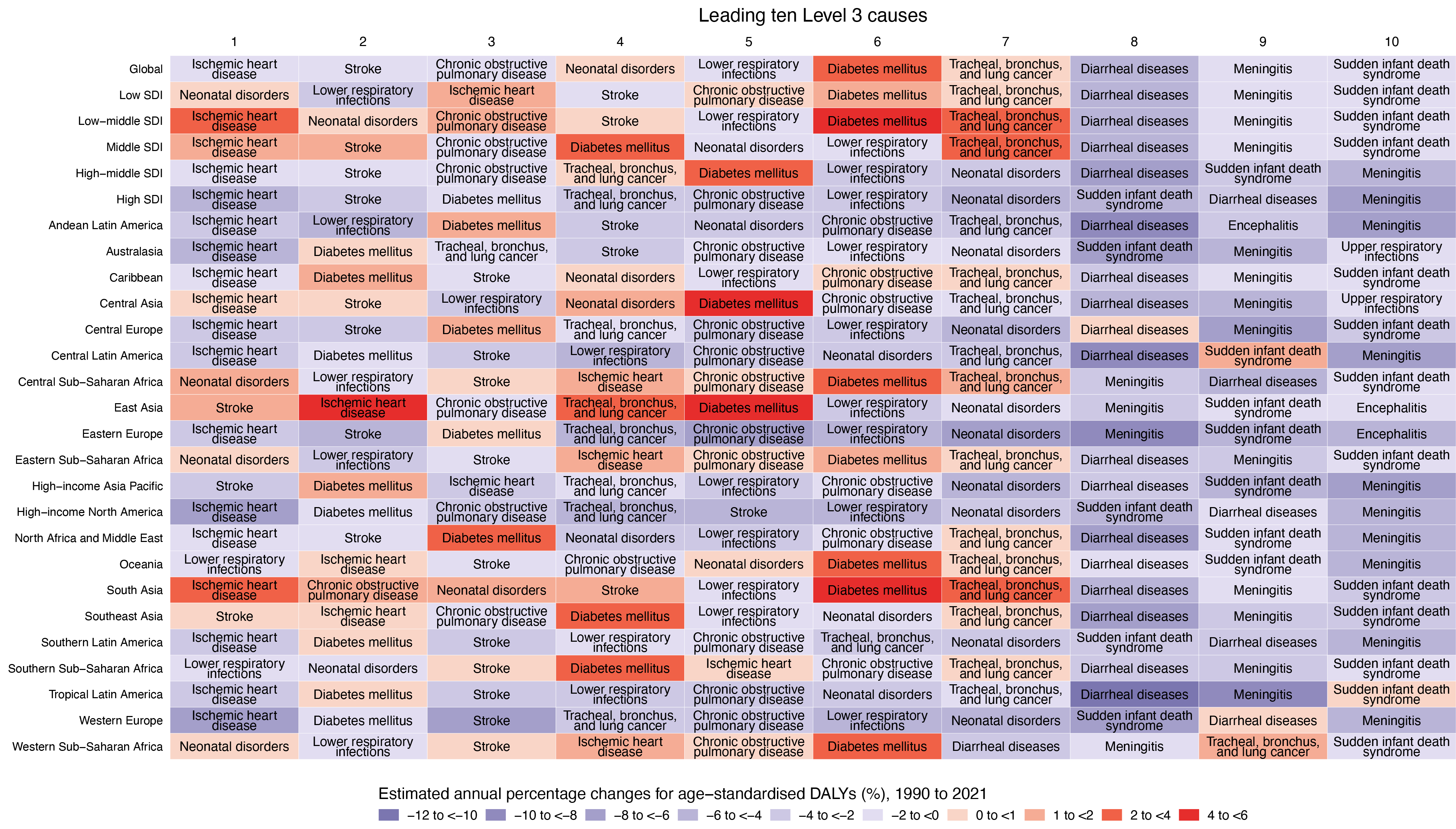

Supplement: Supplementary Materials. — Supplementary appendix. [file agh-92-1-4965-s1.zip › agh-92-1-4965-s1/Figure_3.TIFF]

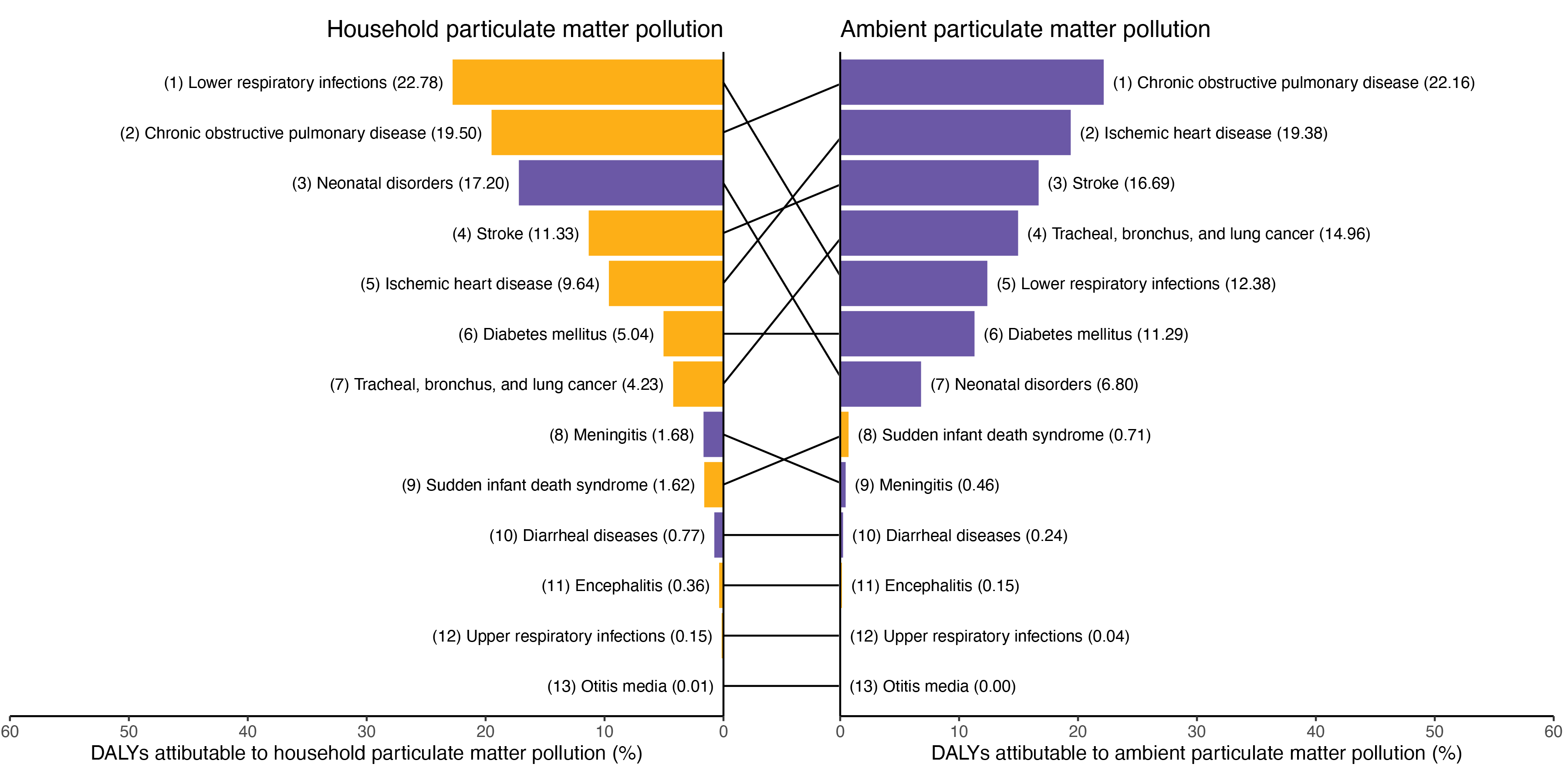

Supplement: Supplementary Materials. — Supplementary appendix. [file agh-92-1-4965-s1.zip › agh-92-1-4965-s1/Figure_4.TIFF]

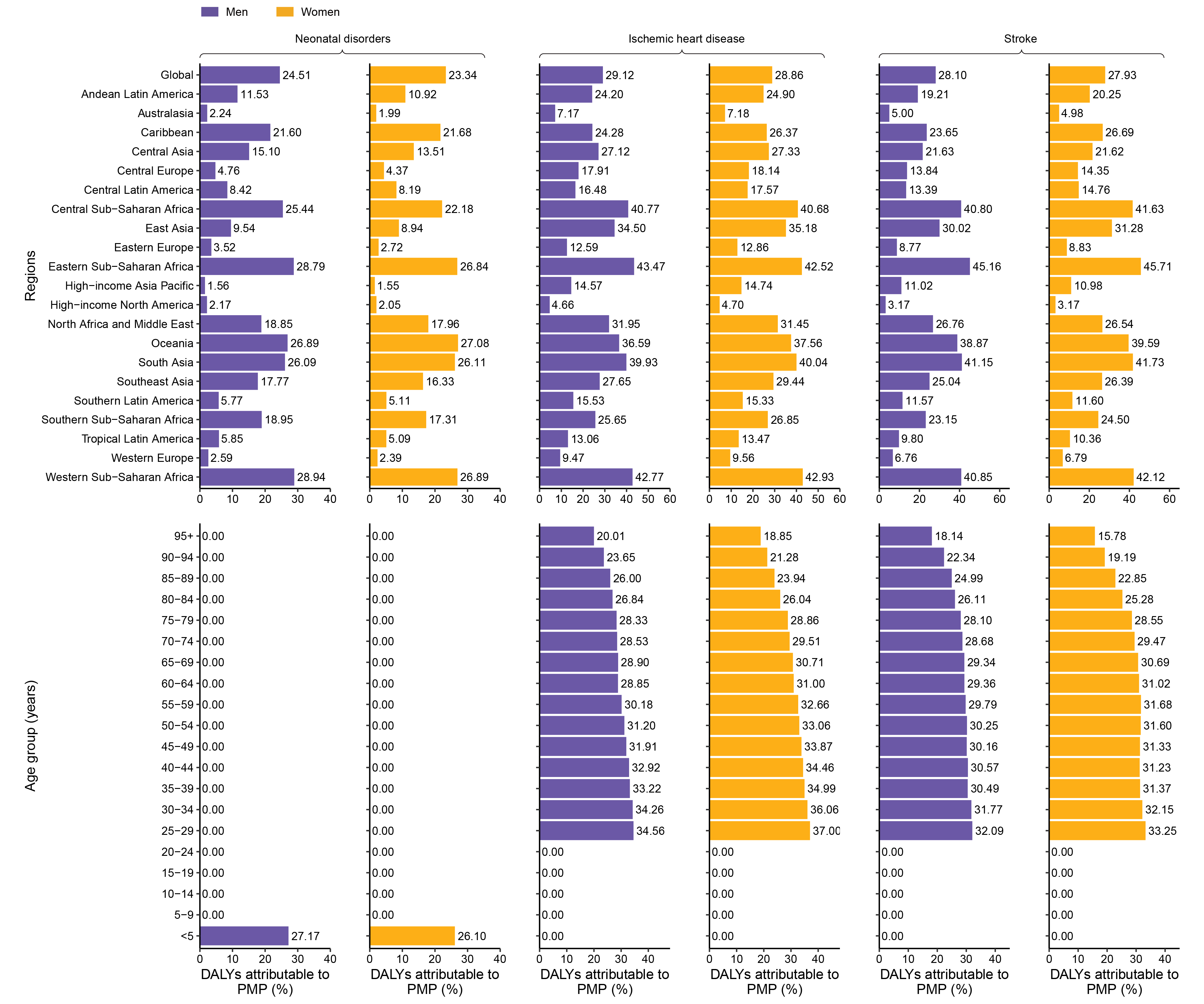

Supplement: Supplementary Materials. — Supplementary appendix. [file agh-92-1-4965-s1.zip › agh-92-1-4965-s1/Figure_5.TIFF]

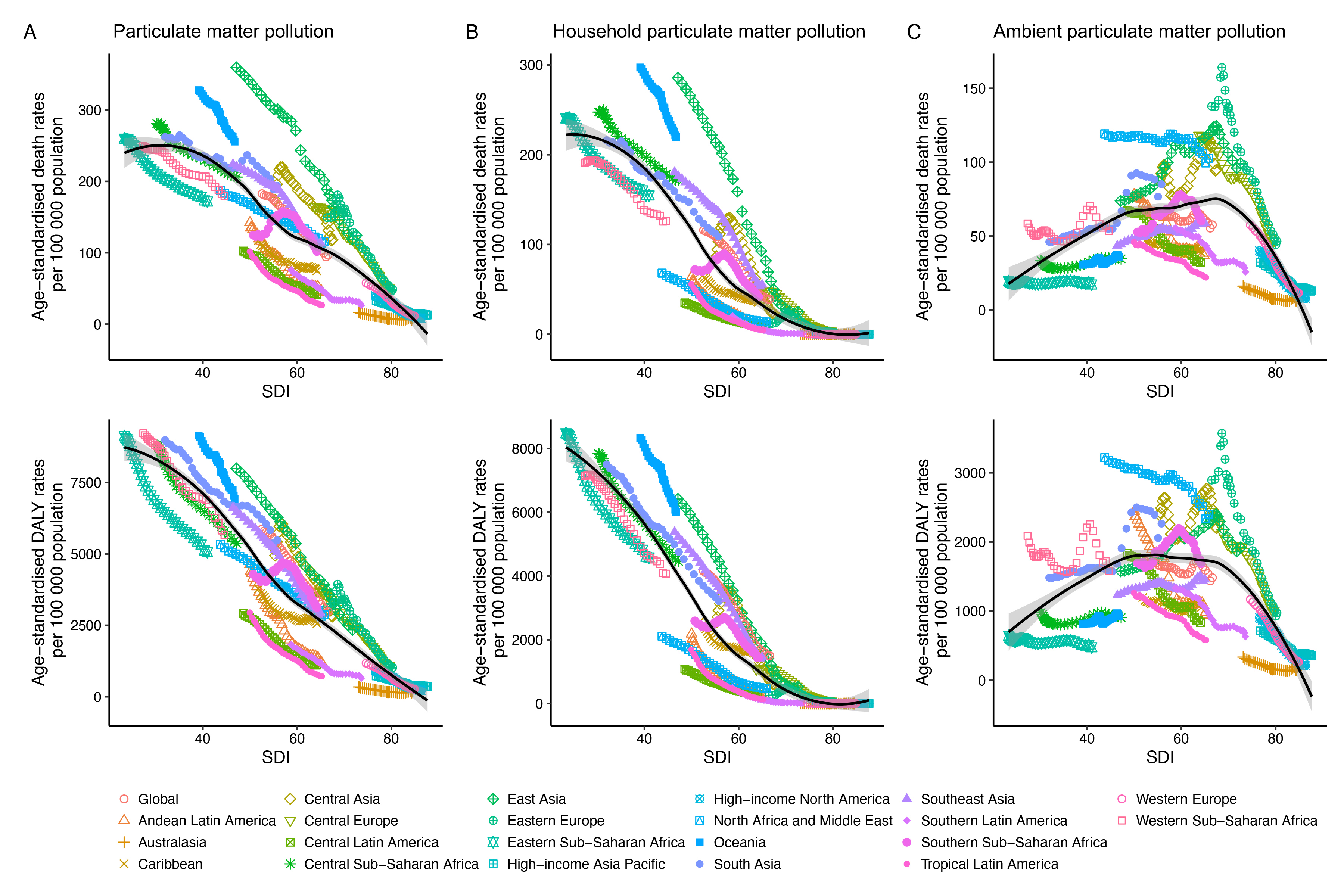

Supplement: Supplementary Materials. — Supplementary appendix. [file agh-92-1-4965-s1.zip › agh-92-1-4965-s1/Figure_6.TIFF]
